# Design, synthesis and insecticidal activity and mechanism research of Chasmanthinine derivatives

**DOI:** 10.1038/s41598-022-19523-8

**Published:** 2022-09-10

**Authors:** Ziyu Song, Xiangyu Li, Ke Xu, Guoqing Sun, Liu Yang, Linyu Huang, Junqi Liu, Pengyuan Yin, Shuai Huang, Feng Gao, Xianli Zhou, Lin Chen

**Affiliations:** 1 School of Life Science and Engineering, Southwest Jiaotong University, Chengdu, 610031 Sichuan People’s Republic of China; 2 Affiliated Hospital of Southwest Jiaotong University & The Third People Hospital of Chengdu, Chengdu, 610031 Sichuan People’s Republic of China

**Keywords:** Organic chemistry, Natural products

## Abstract

Unrestricted reproduction and spread of pest had caused great damage to the quality and yield of crops in recent years. Besides the use of traditional chemical pesticides, natural products also make a huge contribution against pests. Chasmanthinine, a diterpenoid alkaloid isolated from *Aconitum franchetii* var. *villosulum*, shown extremely antifeedant activity against *Spodoptera exigua*. Therefore, a series of novel Chasmanthinine derivatives were synthesized and their biological activity was studied in this work. Compound **33** showed the strongest antifeedant activity (EC_50_ = 0.10 mg/cm^2^) among all the test compounds. The mechanism research of **33** revealed that its antifeedant effect was related to the inhibition of carboxylesterase (CES), and proved the thiophene acyl group could form a strong binding effect with CES by molecular docking. Moreover, compound **10** exhibited the strongest cytotoxicity (IC_50_ = 12.87 μM) against Sf9 cell line and moderate contact toxicity. The mechanism research indicated that compound **10** could induce Sf9 cells apoptosis. In summary, the results lay a foundation for the application of diterpene alkaloids in plant protection.

## Introduction

The unrestricted reproduction and spread of insect pests brought great harm to food security, causing the quality loss and yield reduction of the crop worldwide in recent years^1^. Up to date, although many strategies, including biological control and crossbreeding, have been exploited to protect crop avoiding pest attack. Chemically synthetic insecticides are still considered as one of the most effective strategies of pest control. However, unbounded abuse of existing chemical synthesized pesticides has directly resulted in the drug resistance of pests and the environmental pollution, which has been immensely restricted their application in the agricultural fields^2,3^. The use of plant and plant-derived products to control pests in the developing world is well known and prior to the discovery of synthetic pesticides, plant or plant-based products were the only pest-managing agents available to farmers around the world. Consequently, plant-derived pesticides have gradually become the focus of crop protection in recent years due to their various modes of action, especially the secondary metabolites from plants that possessed various potential pest control activities such as antifeedant, attractant, nematicide, fungicide, insecticide, and insect growth regulation, acting as a promising source for novel pest control agents or biopesticides^4–6^. Among various insecticidal activities from natural products, antifeeding effect plays a significant role, which can protect crop by not killing pests, and has little harm to beneficial insects^7^. Therefore, we purpose to develop the potential novel plant-based lead compounds with excellent antifeedant activity for control pests in the present work.

At present, it has been reported that over 2000 species plants worldwide have insecticidal effects. The genus *Aconitum*, the main branch of Ranunculaceae, as the medicinal plants possess multiple biological activity, which of them were used as crude pesticide to effective prevention of agricultural diseases or control pests in the ancient China^8–12^. Modern pharmacology has been verified that the diterpene alkaloids are the characteristic active ingredients of this genus.

**Figure 1:**
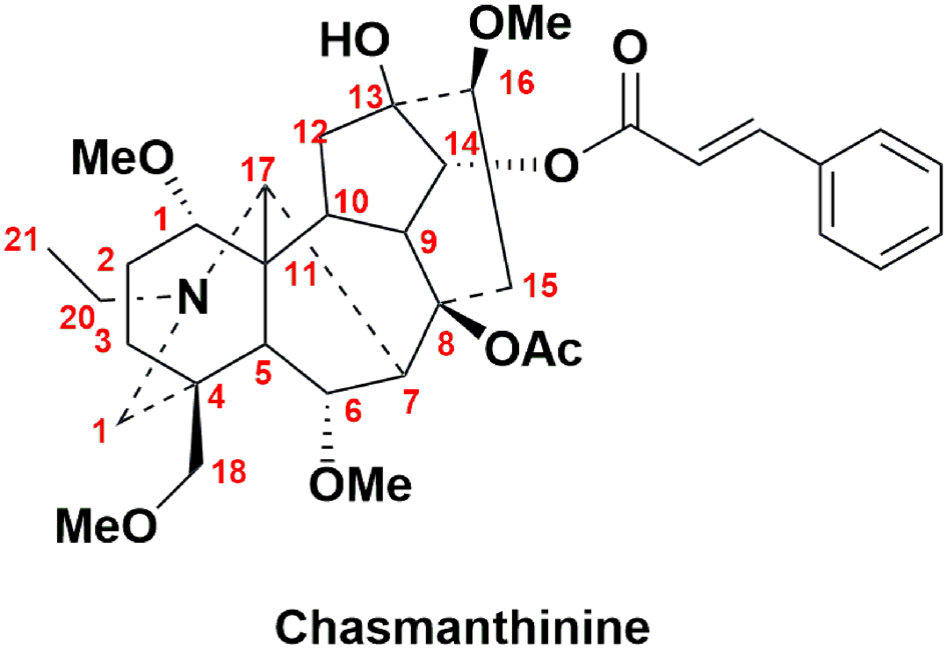
Structure of Chasmanthinine.

Our previous research has proved that Chasmanthinine (Fig. 1), a natural C_19_-aconitine type diterpene alkaloid isolated from *Aconitum franchetii* var. *villosulum*, exhibited the remarkable antifeeding activity against *Spodoptera exigua* (EC_50_ = 0.11 mg/cm^2^) and was roughly equivalent to the commercial pesticide Azadirachtin A (EC_50_ = 0.08 mg/cm^2^). Furthermore, in our continuous studies for the antifeedant effect of Chasmanthinine analogues, many efforts were made to further enhance its activity, revealing the positions of C-13, C-8 and C-14 could be the active sites of Chasmanthinine^13,14^. Therefore, to enrich the structural types of derivatives of Chasmanthinine and search for analogs with excellent antifeedant activity, we first designed and synthesized a series of Chasmanthinine derivatives with characteristic hydroxyl or acetyl at C-8 and hydrogen at C-13 and different ester substituents at C-14. And their bioactivities also were evaluated.

## Results and discussion

### Chemical synthesis

**Figure 2:**
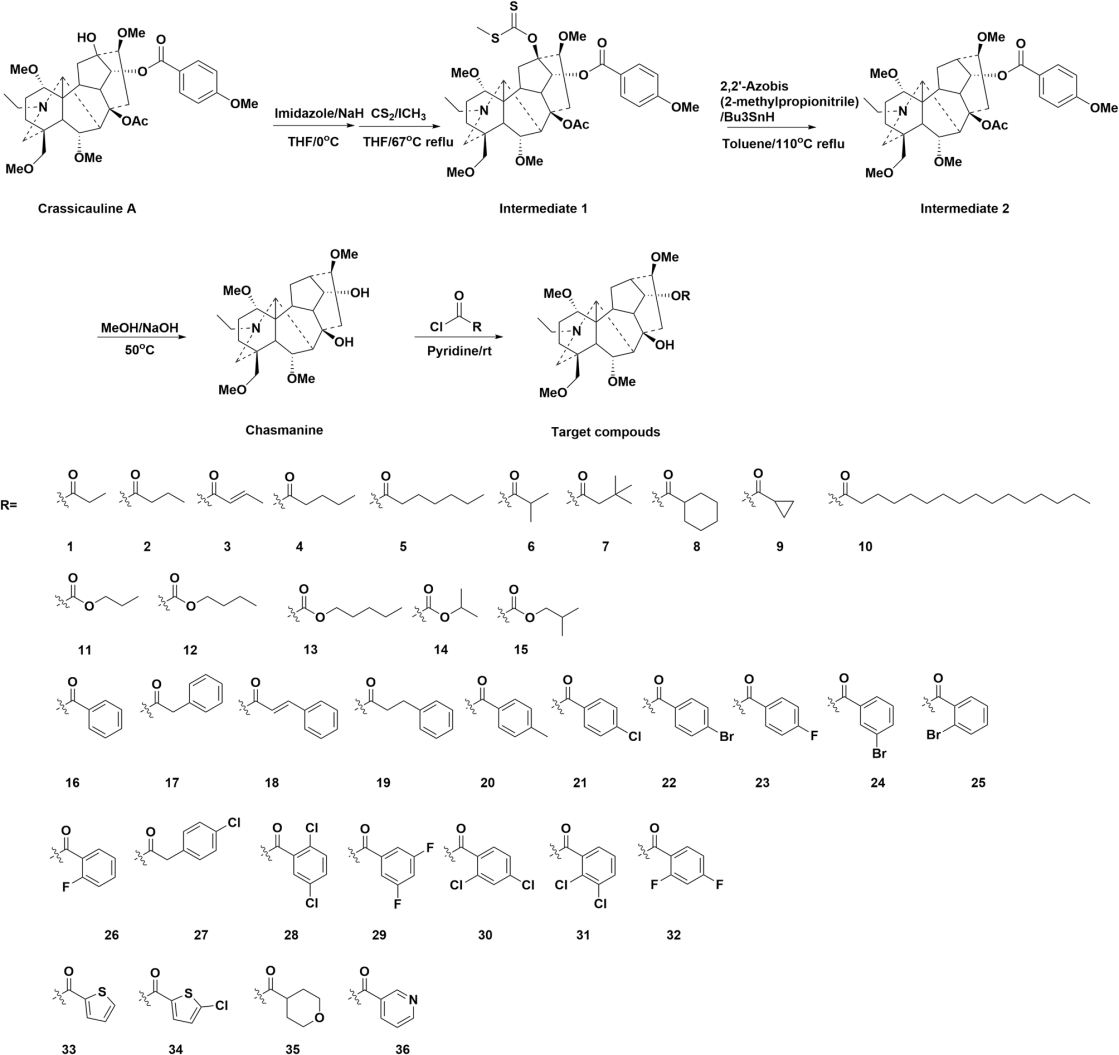
Structures and synthetic route of target compounds.

Thirty-six monoester derivatives with different ester substituents at C-14, including 15 substituted fatty acyl ester derivatives (compounds **1** ~ **15**), 17 benzoyl (compounds **16** ~ **32**) derivatives and 4 heterocyclic derivatives (compounds **33** ~ **36**) were successfully prepared in four steps as shown in Fig. 2. Intermediate **1** was obtained by nucleophilic addition reaction occurred on the 13-hydroxyl group of Crassicauline A. Subsequently, the deoxygenation reduction leaded to the cleavage of the carbon–oxygen bond to generate intermediate **2**. Then, hydrolysis reaction occurred under alkaline conditions to generate key intermediate Chasmanine. The target compounds were obtained by esterification reaction of Chasmanine with different acyl chlorides in good yields. Pyridine was used as acid binding agent in this step. The secondary alcohol (C-14) hydroxyl group of Chasmanine is relatively active than tertiary alcohol (C-8) hydroxyl groups in esterification reaction. Therefore, when preparing the target compounds, the reaction conditions, such as the rate of adding acid chloride, the reaction temperature, and the reaction time should be strictly controlled to ensure the occurrence of the reaction and increase the yield.

In the ^13^C-NMR spectra of the synthetic compounds, the typical ester carbonyl signal at 174 ~ 162 ppm and oxygenated quaternary carbons resonances at 76 ppm strongly indicated that the ester group was substituted at C_14_ and the appearance of hydroxyl groups at C_8_. The chemical shift of C_14_ chain-substituent carbons were reflected at 13 ~ 43 ppm. Carbon chemical shifts of benzoyl and aromatic heterocyclic acyl substituents was around 100 to 165 ppm. This conclusion was also supported by their molecular mass.

**Figure 3:**
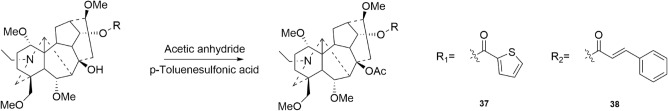
Synthetic route of target compounds.

Moreover, two diester derivatives (**37**) and (**38**) were synthesized by one step reaction as shown in Fig. 3. In ^13^C-NMR spectra, the typical C_14_ ester carbonyl signals were appeared at 174 ~ 169 ppm. The carbon shifts of C_8_ ranged from 74 to 86 ppm compared with that of Chasmanine^15^. And the acetyl carbon spectrum signals were 168 ~ 166 ppm.

All the title compounds and intermediates were identified by ^1^H-NMR, ^13^C-NMR and HR–ESI–MS. The spectral data was given in the supporting information.

### Antifeedant activity

To find potential candidates with antifeedant activity against the larvae of *S. exigua* (Hübner), the choice leaf-disk method was used to evaluate the antifeedant effects of synthetic compounds (**1** ~ **38**), using Chasmanthinine and Azadirachtin A as the reference drugs. Primary screening of all target compounds was conducted at a single concentration of 0.5 mg/mL, and the refusal rate (FR) are shown in Table 1.

**Table 1:** The refusal rate (FR) of target compounds against *S. exigua* at 0.50 mg/mL.

Compd.	R	FR (%) ± SD	Compd.	R	FR (%) ± SD
**1**	Propionyl	< 30	**21**	4-Chlorobenzoyl	44.00 ± 0.19
**2**	Butyryl	36.57 ± 0.19	**22**	4-Bromobenzoyl	71.23 ± 0.03
**3**	2-Butenoyl	41.89 ± 0.10	**23**	4-Fluorobenzoyl	63.17 ± 0.12
**4**	Valeryl	< 30	**24**	3-Bromobenzoyl	56.07 ± 0.01
**5**	Heptanoyl	53.75 ± 0.10	**25**	2-Bromobenzoyl	37.18 ± 0.14
**6**	Isobutyryl	< 30	**26**	2-Fluorobenzoyl	37.31 ± 0.07
**7**	3,3-Dimethylbutyryl	< 30	**27**	4-Chlorophenylacetyl	44.00 ± 0.11
**8**	Cyclohexylformyl	43.76 ± 0.12	**28**	2,5-Dichlorobenzoyl	45.58 ± 0.07
**9**	Cyclopropylformyl	44.14 ± 0.23	**29**	3,5-Difluorobenzoyl	53.05 ± 0.09
**10**	Palmitoyl	< 30	**30**	2,4-Dichlorobenzoyl	< 30
**11**	Propyl formyl	49.85 ± 0.17	**31**	2,3-Dichlorobenzoyl	39.30 ± 0.04
**12**	Butyl formyl	< 30	**32**	2,4-Difluorobenzoyl	38.69 ± 0.15
**13**	n-Pentyl formyl	34.03 ± 0.25	**33**	thienoyl	90.20 ± 0.11
**14**	Isopropyl formyl	38.91 ± 0.22	**34**	2-Chlorothiophene formyl	51.84 ± 0.20
**15**	Isobutyl formyl	43.19 ± 0.13	**35**	tetrahydropyranoyl	< 30
**16**	Benzoyl	46.17 ± 0.04	**36**	Nicotinyl	48.00 ± 0.10
**17**	Phenylacetyl	39.62 ± 0.03	**37**	8-OAc, 14-thienoyl	61.27 ± 0.12
**18**	Cinnamoyl	83.33 ± 0.02	**38**	8-OAc, 14-cinnamoyl	73.12 ± 0.08
**19**	3-Phenylpropionyl	< 30	**Chasmanthinine**		90.07 ± 0.07
**20**	4-Methylbenzoyl	30.38 ± 0.07	**Azadirachtin A**		98.20 ± 1.29

The experiments were repeated three times.

When substituents were aliphatic chains installed at C-14 position (compounds **1** ~ **15**), compound **5** containing heptanoyl was the most active, with an FR of 53.75%. The results shown that the length of the alkyl have significant impact on the antifeedant activity of Chasmanthinine analogues. We observed slightly higher activities for compound **10** containing palmitoyl (FR = 26.02%) than for **1** with propionyl (FR = 23.95%). Introduction of the cycloalkane group improved the antifeedant effects (compound **8** and **9**, substituted with cyclopropyl and cyclohexyl respectively, FR = 43.76% and 44.14%). In conclusion, these derivatives showed moderate antifeedant effect and compounds with great activity still need to be further screened.

Among the derivatives (**16** ~ **32**) of aromatic group-substituted, compound **16** (R = benzoyl) exhibited moderate biological activity (FR = 46.17%). Compounds **19** (R = propyl benzoyl) and **18** (R = cinnamoyl) exhibited distinct activity (FR = 28.61%, 83.33% respectively), which suggested that the activity was increased when the side chain was conjugated with the carbonyl group. The weak activity of compound **17** (R = phenylacetyl, FR = 39.62%) could also prove above conjecture. Halogen atoms often possess a variety of biological activities^16^. When halogen atoms were introduced into the para-position of the C-14 benzoyl group, activity was increased compared to the compound **16** and the activity was as follow: **Br** > **F** > **Cl (**compounds **22**, **23** and **21,** FR = 71.23%, 63.17% and 44.00% respectively). However, when halogen atoms appear at ortho or meta position such as compound **24**, **25**, **26** (C-14 substituted with 3-bromobenzoyl, 2-bromobenzoyl and 2-fluorobenzoyl respectively, FR = 56.07%, 37.18% and 37.31% respectively), antifeedant effect decreased. Multiple halogen atoms introduced into the molecules, such as compound **30** and **32** (FR = 21.45% and 38.69%), also caused the activity decreased. In addition, compound **20** (containing 4-methylbenzoyl) shown weak antifeedant activity (FR = 30.38%). It means that the introduction of electron-donating groups on the benzene ring may cause decrease in activity.

Derivatives with heterocycle (compounds **33 ~ 36**) shown that introduction of thiophene (compound **33**) can greatly improve the activity (FR = 90.20%). When chlorine atom was introduced into thiophene (compound **34**), the activity was drastically reduced (FR = 51.84%).

The number of ester groups in diterpene alkaloids is an important factor affecting the activity^17^. However, after 8-hydroxyacetylation of derivatives **33** and **18** (FR = 90.20% and 83.33%), the activity slightly weakened (compound **37** and **38,** FR = 61.27% and 73.12%). This indicated that C_8_-OAc was not an essential group for antifeedant activity.

In addition, compounds whose FR was greater than 60% (compounds **18**, **22**, **23**, **33**, **37** and **38**) were selected to evaluate antifeedant activities at 1 mg/mL, 0.5 mg/mL, 0.25 mg/mL, 0.125 mg/mL, and 0.0625 mg/mL. The half maximal effective concentration (EC_50_) and 95% Confidence Interval were calculated by Origin 2019b. The result was shown in Table 2.

**Table 2:** EC_50_ values of antifeedant activity of title compounds against *S. exigua* (Hübner).

Compod.	R	EC_50_ (mg/cm^2^)	95% confidence interval
**18**	Cinnamoyl	0.13	(0.12, 0.15)
**22**	4-Bromobenzoyl	0.27	(0.17, 0.46)
**23**	4-Fluorobenzoyl	0.41	(0.30, 0.60)
**33**	thienoyl	0.10	(0.07, 0.15)
**37**	14-Thienoyl, 8-OAc	0.21	(0.10, 0.49)
**38**	14-Cinnamoyl, 8-OAc	0.24	(0.20, 0.29)
**Chasmanthinine**		0.11	(0.07, 0.16)
**Azadirachtin A**		0.08	(0.08, 0.09)

The experiments were repeated three times.

Compound **23** showed lower antifeedant activity (EC_50_ = 0.41 mg/cm^2^). Compounds **18**, **22**, **37** and **38** showed good biological activity (EC_50_ = 0.13, 0.27, 0.21 and 0.24 mg/cm^2^, respectively). However, none of these target compounds are as active as the lead compound, Chasmanthinine. Surprisingly, compound **33** (EC_50_ = 0.10 mg/cm^2^) exhibited enhanced activities superior to the lead compound Chasmanthinine (EC_50_ = 0.11 mg/cm^2^), which was closed to the positive control Azadirachtin A (EC_50_ = 0.08 mg/cm^2^). In general, the results of the EC_50_ value were basically consistent with our preliminary screening results.

### Cytotoxicity of Sf9 cells

During the antifeeding experiment, a small number of test worms died unexpectedly. We suspected that this result was related to the cytotoxicity produced by the compound. In order to verify our supposition, we tested the cytotoxicity of Sf9 cells for all target compounds. Sf9 cells derived from *S. frugiperda* have been widely used as basic models of insecticide cytotoxicology to study pesticide effects due to the in vitro experiments having the advantages of high throughput and high reproducibility^18–21^. Therefore, MTT colorimetry was used to test the cytotoxicity of the target compounds on Sf9 cells. The inhibition rate (IR) was shown in Table 3.

**Table 3:** The Inhibition Rate (IR) of target compounds against Sf9 cells at 100 μM.

Compd.	R	IR (%) ± SD	Compd.	R	IR (%) ± SD
**1**	Propionyl	< 30	**21**	4-Chlorobenzoyl	< 30
**2**	Butyryl	< 30	**22**	4-Bromobenzoyl	< 30
**3**	2-Butenoyl	< 30	**23**	4-Fluorobenzoyl	< 30
**4**	Valeryl	42.40 ± 4.25	**24**	3-Bromobenzoyl	38.33 ± 3.16
**5**	Heptanoyl	< 30	**25**	2-Bromobenzoyl	< 30
**6**	Isobutyryl	< 30	**26**	2-Fluorobenzoyl	< 30
**7**	3,3-Dimethylbutyryl	< 30	**27**	4-Chlorophenylacetyl	< 30
**8**	Cyclohexylformyl	< 30	**28**	2,5-Dichlorobenzoyl	31.43 ± 2.13
**9**	Cyclopropylformyl	< 30	**29**	3,5-Difluorobenzoyl	< 30
**10**	Palmitoyl	94.88 ± 1.79	**30**	2,4-Dichlorobenzoyl	52.79 ± 1.42
**11**	Propyl formyl	< 30	**31**	2,3-Dichlorobenzoyl	30.44 ± 4.60
**12**	Butyl formyl	< 30	**32**	2,4-Difluorobenzoyl	< 30
**13**	*n*-Pentyl formyl	< 30	**33**	thienoyl	< 30
**14**	Isopropyl Formyl	< 30	**34**	2-Chlorothiophene formyl	< 30
**15**	Isobutyl formyl	< 30	**35**	tetrahydropyranoyl	< 30
**16**	Benzoyl	< 30	**36**	Nicotinyl	< 30
**17**	Phenylacetyl	< 30	**37**	8-OAc, 14-thienoyl	< 30
**18**	Cinnamoyl	< 30	**38**	8-OAc, 14-cinnamoyl	< 30
**19**	3-Phenylpropionyl	30.46 ± 4.35	**Azadirachtin A**	**–**	98.02 ± 1.93
**20**	4-Methylbenzoyl	< 30			

The experiments were repeated six times.

Among all title compounds, most compounds exhibited the lower Sf9 cytotoxicity (IR < 30%), compound **30** substituted with 2,4-dichlorobenzoyl at C-14 exhibited moderate cytotoxicity (IR = 52.79%). Compound **10** with C-14 palmitoyl showed the best cytotoxic activity (IR = 94.88%) as well as in accordance with Azadirachtin A (IR = 98.02%).

For further study, the half maximal inhibitory concentration (IC_50_) of compounds with good activity (IR > 50%) was evaluated by set a concentration gradient and the results was shown in Table 4.

**Table 4:** The half maximal inhibitory concentration (IC_50_) of target compounds against Sf9 cells.

Compds.	R	IC_50_ (*μ*M)	95% confidence interval
10	Palmitoyl	12.87	(11.96, 13.85)
30	2,4-Dichlorobenzoyl	126.80	(122.40, 131.30)
Azadirachtin A		4.54	(3.26, 6.38)

The experiments were repeated six times.

Compound **30** exhibited weak IC_50_ value (126.80 μM). While compound **10** showed great activity (IC_50_ = 12.87 μM) and was closed to Azadirachtin A (IC_50_ = 4.54 μM). This suggested that the introduction of long-chain at C-14 in diterpene alkaloids could improve the cytotoxicity of Sf9 cells significantly.

Additionally, compound **10** and **30** were used to evaluate the contact toxicity against *Spodoptera exigua* and the result show in Table 5. Compound **10** containing palmitoyl group exhibited better toxicity than compound **30** with 2,4-dichlorobenzoyl substituted (LC_50_ = 7.90 mg/mL, 11.85 mg/mL) which was consistent with conclusions at the cellular level.

**Table 5:** Lethal concentration 50% (LC_50_) of compound 10 and 30 against *Spodoptera exigua.*

Compds.	LC_50_ (mg/mL)	95% Confidence Interval
**10**	5.79	(5.08, 6.56)
**30**	12.58	(8.44, 28.23)
**Cyhalothrin**	3.92	(2.36, 5.97)

The experiments were repeated three times.

### Study on the mechanism of antifeedant activity

Research on the mechanism is of great significance to the development of insecticides. Acetylcholinesterase (AChE), carboxylesterase (CES), mixed function oxidase (MFO) and glutathione S-transferases (GSTs) are the vital target sites associated with antifeedant activities^22^. Therefore, the inhibitory effect on AChE, CES, MFO and GSTs of *S. exigua* (Hübner) were performed to evaluate the potential target sites of compound **33**. The results are shown in Table 6.

**Table 6:** Inhibition rate (IR) of enzymes of compound **33** in concentration of 0.5 mg/mL.

	IR (%) ± SD			
	AChE	MFO	GST	CES
Compound **33**	3.82 ± 0.01	84.23 ± 0.11	4.22 ± 0.18	90.23 ± 0.01
Azadirachtin A	< 0	< 0	71.24 ± 0.13	88.37 ± 0.01

The experiments were repeated three times.

*AChE* Acetylcholinesterase, *MFO* Mixed function oxidase, *GST* Glutathione S-transferases, *CES* Carboxylesterase.

Compound **33** exhibited great inhibitory activity (IR = 90.18%) on CES at 0.5 mg/mL and moderate activity on MFO (IR = 84.23%), while shown weaker activity to AChE and GST.

**Figure 4:**
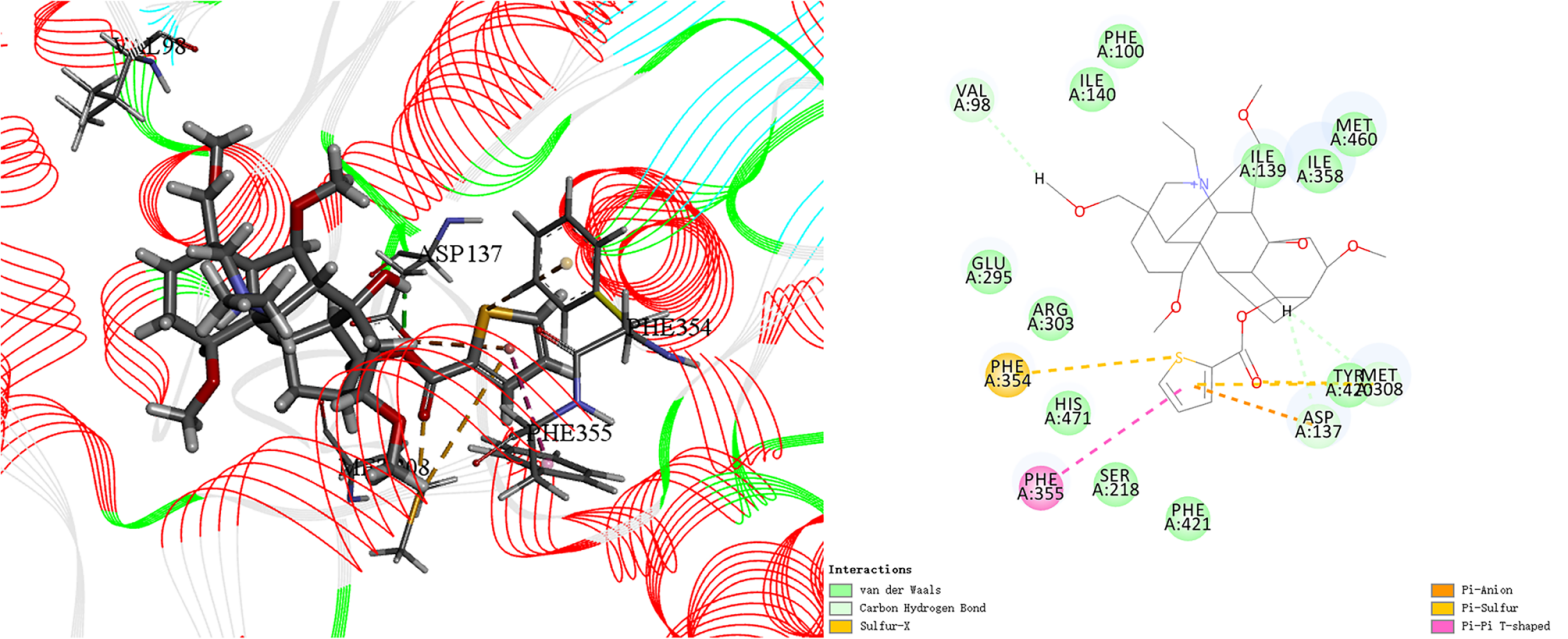
Docking result of receptor-ligand interaction of compound **33** and 5TYJ.

Molecular docking is a powerful tool to predict the interaction between small molecules and biological macromolecules. Based on the in vitro interaction mechanism, we studied the interaction between compound **33** and enzyme. CES (PDB code: 5TYJ) was used as a template to dock with compound **33**. The docking results (Fig. 4) including 3D and 2D graphics were obtained directly generated by Discovery Studio 2019.

Result showed that C_8_-hydroxyl group could interact with Val98 by forming a carbon hydrogen bond. The 14-position hydrogen atom formed the carbon hydrogen interaction with Asp137 and Met308 in the active pocket. In addition, the thienoyl group had a good binding site with the receptor, which can form Pi–Pi T-shaped interaction with Phe355 and can also form a strong Pi-Sulfur interaction with Met308 and Phe354. Meanwhile, it also formed a Pi-Anion with Asp137. Therefore, the role of thiophene and sulfur atoms in the binding is relatively significant, which can effectively improve the binding strength of the target compound to the receptor and provide a rational reference for finding compounds with great activity.

### Study on the mechanism of Sf9 cytotoxicity

#### Sf9 cells morphology observation

**Figure 5:**
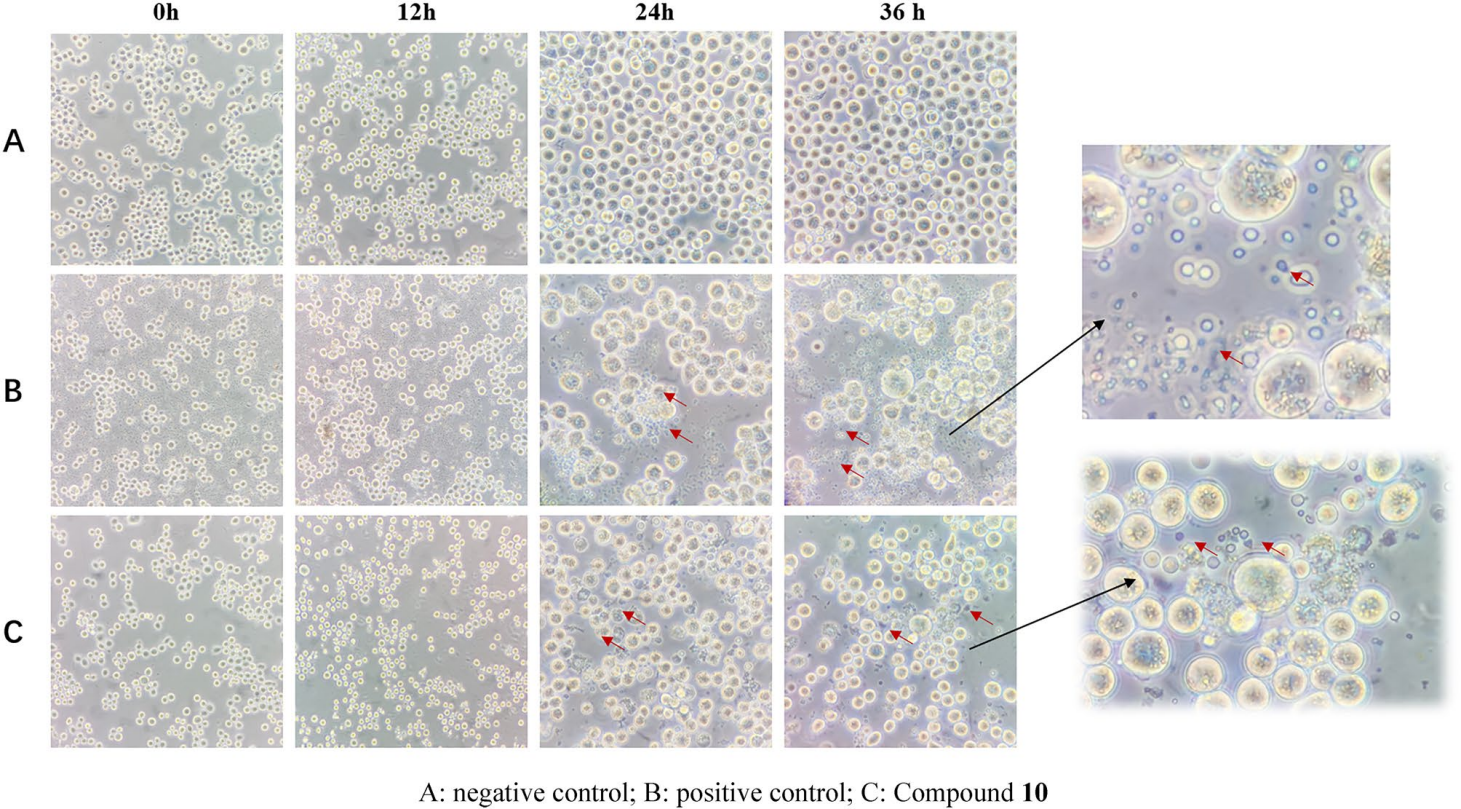
Cell morphology observations.

Morphological changes of cells are considered as the basis evidences to estimate the apoptosis which involved in development, homeostasis and cellular defense of multicellular organism by eliminating unwanted cells. Studies have confirmed that Azadirachtin A can induce apoptosis of Sf9 cells^23^, so it was selected as control. The results shown in the Fig. 5. After 12 h of induction, Sf9 cells were full of outer shape, mostly round in negative control. Comparad to positive control group, there was no significant change in the drug-treated group except for a slight decrease in the number of cells. After 24–36 h, the number of cells in drug-treated group was significantly reduced, and a large number of apoptotic bodies appeared. Likewise, apoptotic bodies also appeared in the positive control while cells in the negative control proliferated normally. A few numbers of cells in the drug-treated group clumped, floated, swelled and death after 36 h of induction with compound **10**. Above results suggested that the anti-proliferative mechanism of compound **10** may be related to the induction of apoptosis, but it needs to be confirmed by further studies.

#### Fluorescence staining

**Figure 6:**
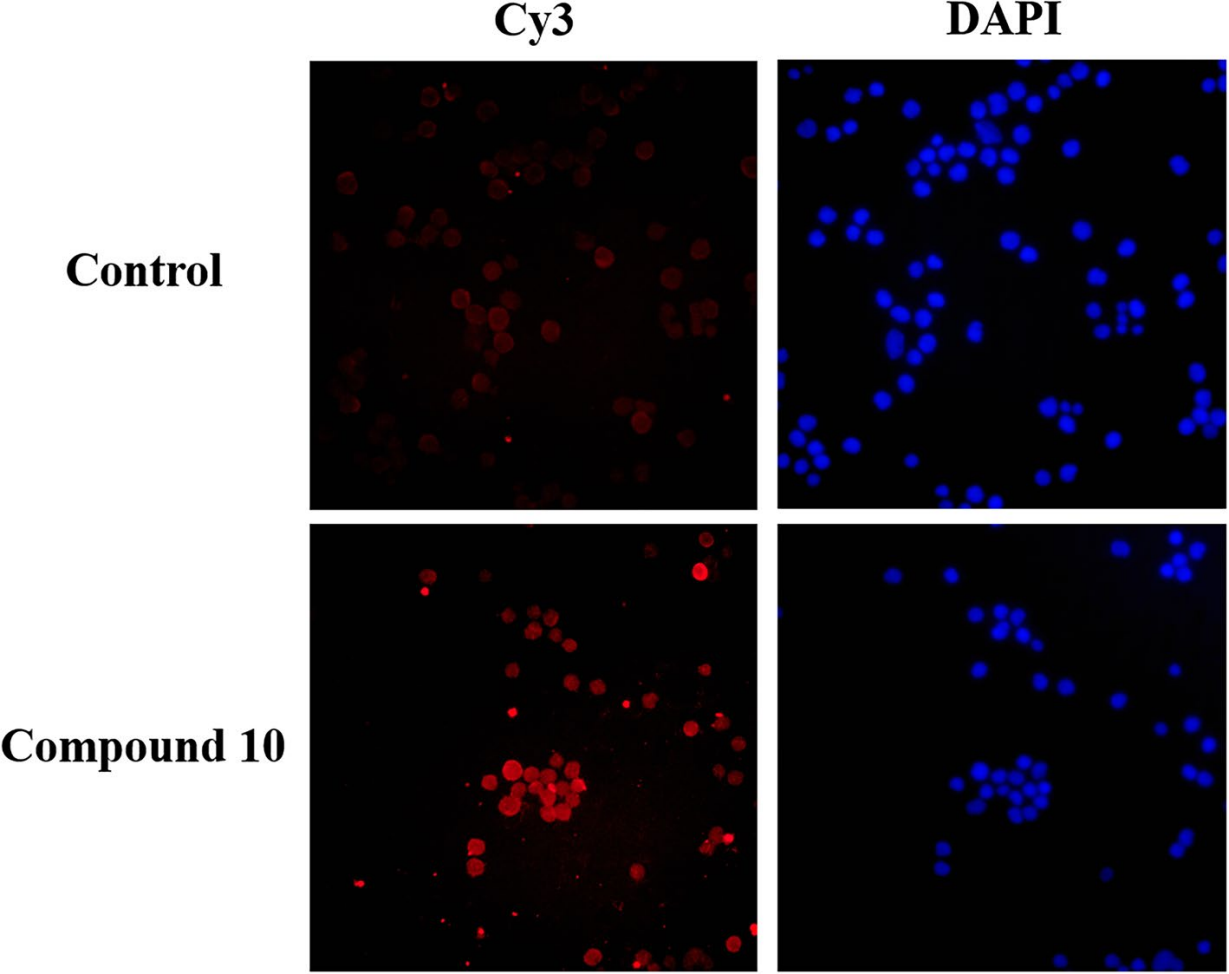
Sf9 cells stained with Cy3-dUTP after treatment compound **10** (15 μM) for 24 h.

During apoptosis, some endonucleases are activated and cut off the genomic DNA between nucleosomes. When genomic DNA is broken, the exposed 3′-OH can be catalyzed by Terminal Deoxynucleotidyl Transferase (TdT) to add dUTP labeled with the red fluorescent probe Cyanine 3 (Cy3), so that it can be detected by fluorescence microscopy. The results were shown in Fig. 6. After 24 h, compared with the blank group, the cells treated with compound **10** (15 μM) showed obvious bright red fluorescence, confirming that it could induce apoptosis of Sf9 cells.

#### DNA ladder

**Figure 7:**
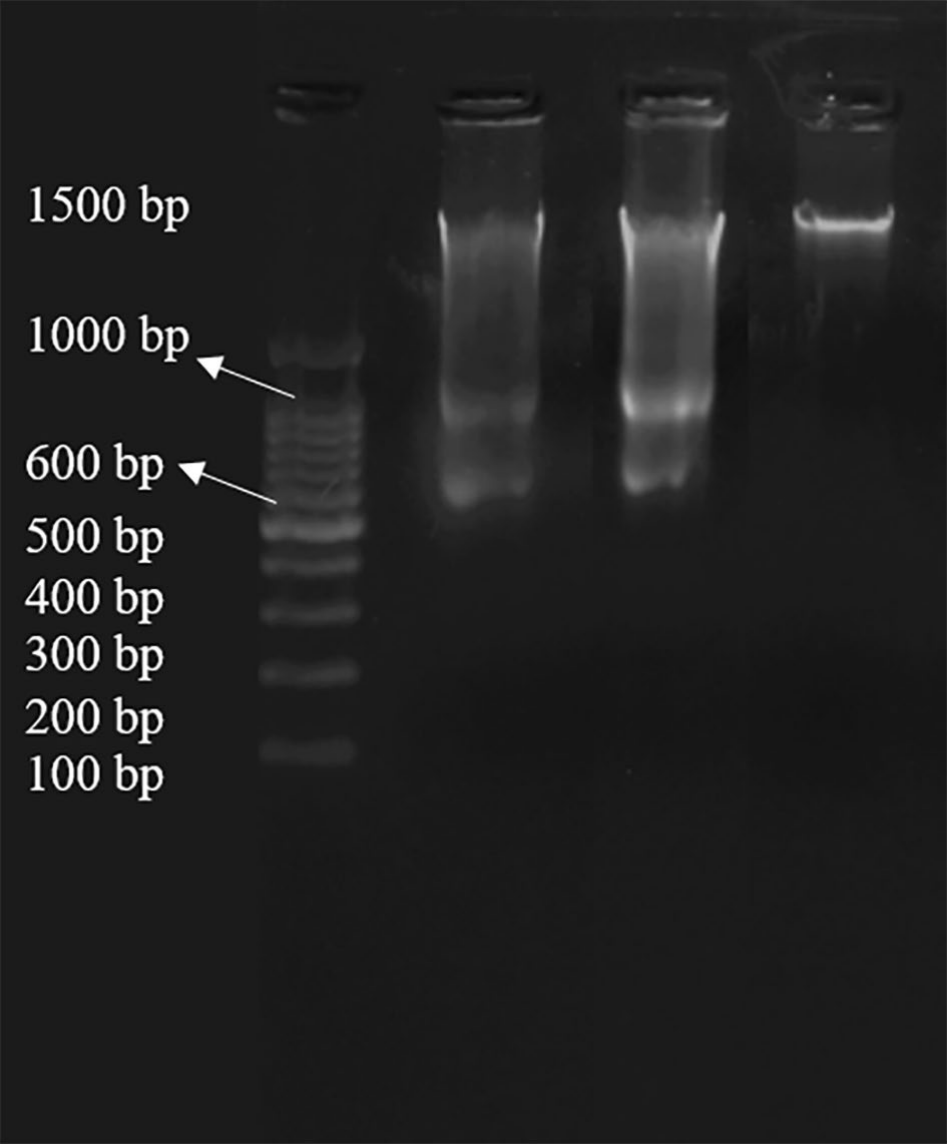
DNA ladder results of Sf9 cells induced by compound **10**. Lanes: 1: 2000 bp marker; 2: positive control (Azadirachtin A); 3: compound **10**; 4: negative control.

In cells undergoing apoptosis (programmed cell death), a fraction of nuclear DNA is fragmented to the size equivalent of DNA in mono-or oligonucleosomes. When such DNA is analyzed by agarose gel electrophoresis it generates the characteristic “ladder” pattern of discontinuous DNA fragments. Such a pattern of DNA degradation generally serves as a marker of the apoptotic mode of cell death^24^. Therefore, DNA gel electrophoresis was used to observe Sf9 cell fragmentation, so as to clarify whether its toxicity is related to apoptosis and the result shown in the Fig. 7.

After treatment of compound **10** for 24 h, characteristic DNA ladder was observed clearly, which was consistent with the results of the positive control Azadirachtin A. Since the previous study had been confirmed that Azadirachtin A induces apoptosis of Sf9 cells^25,26^. Combined with the morphological observations and fluorescence staining of Sf9 cells, it could be speculated that the cytotoxicity of Sf9 cell line is closely related to apoptosis induced by compound **10**.

## Conclusions

Thirty-eight novel derivatives of Chasmanthinine were synthesized and their insecticidal activities were evaluated to discuss their structure–activity relationship. The results illustrated that compound **33** containing a thienyl group at the C_14_ position showed the strongest antifeedant activities (EC_50_ = 0.10 mg/cm^2^) among all tested compounds. Compound **10** with palmitoyl (IR = 94.88% at 100 μM, IC_50_ = 12.87 μM) showed the strongest cytotoxicity to Sf9 cells among all derivatives. These compounds had the good potential for agricultural application and possessed the further research significance.

In addition, the mechanism of antifeedant effect was explored by evaluating in vitro enzyme activity inhibition and molecular docking. Results showed that compound **33** had good binding effect with carboxylesterase. And the mechanism of Sf9 cytotoxicity was explained by morphological observation, fluorescence staining and DNA ladder, which indicated that the mechanism of cytotoxicity may be related to apoptosis.

This work completed the exploration of insecticidal activities and mechanisms of Chasmanthinine derivatives for the first time, which has important guiding significance for the development of plant-derived pesticides.

## Materials and methods

### Materials

#### Chemical materials

Unless otherwise specified, the solvents used herein are all commercially available analytical or chemical grades and used directly without any purification. Crassicauline A was purchased from Chengdu Lemeitian Pharmaceutical Technology Co., Ltd. Chasmanthinine was obtained from *Aconitum franchetii* var. *villosulum* in previous work. Azadirachtin A, Imidazole, NaH, CS_2_, CH_3_I, 2,2′-Azobis(2-methylpropionitrile), T-Bu_3_SnH, and *p-*Toluenesulfonic acid were purchased from Beijing Inokay Technology Co., Ltd. Thin-layer chromatography silica gel GF 254 and column chromatography silica gel G and H (200 ~ 400 mesh) were produced by Qingdao Ocean Chemical Plant.

#### Biological material

*Spodoptera exigua* (Hübner) were purchased from Henan Jiyuan Baiyun Industry Co., Ltd. Sf9 cells (*S. frugiperda* ovary cells), Thiazole blue (MTT) and Dimethyl sulphoxide (DMSO) were purchased from Wuhan Procell Life Technology Co., Ltd. One Step TUNEL Apoptosis Assay Kit and universal genomic DNA purification mini spin kit were purchased from Shanghai Beyotime Institute of Biotechnology.

#### Analytical methods

^1^H and ^13^C nuclear magnetic resonance (NMR) spectra were recorded on a Bruker AV 400 nuclear magnetic resonance instrument (400 MHz). Chemical shifts were recorded in parts per million (ppm) relative to tetramethyl silane as the internal standard. HRMS (ESI) were carried out on a Q-TOF micro mass spectrometer (Waters, USA). Absorbance was recorded on microplate reader (Spectra Max C Max Plus, Molecular Devices, Sunnyvale, CA, USA). The DNA Laddar was recorded by scanning on a multipurpose imager (Invitrogen, Singapore).

### Synthetic procedures

#### General synthetic procedure for intermediate 1, 2 and Chasmanine

##### Intermediate 1

The mixture of Crassicauline A (0.76 mM), imidazole (0.51 mM) and NaH (0.41 mol) was dissolved in dry THF and stirred at 0 °C for 1.5 h under an argon atmosphere. Then the reaction system was transferred to reflux at 67 °C and add CS_2_ and CH_3_I slowly and the reaction was monitored by TLC. At end of the reaction, ice water was slowly added to stop reaction. Then the reaction solution was extracted with EA (20 mL × 5), the organic layers were combined, and concentrated under reduced pressure to obtain the intermediate 1 (yield 95%).

##### Intermediate 2

The mixture of intermediate 1 (0.55 mM) and 2,2′-Azobis(2-methylpropionitrile) (1.22 mM) was dissolved in dry toluene. T-Bu_3_SnH (740 μL) was slowly added dropwise to the solution and reflux at 110 °C for 2 h. After cooling down to room temperature, KF was added to the system and stirred for 1 h and the reaction was monitored by TLC. At end of the reaction, solution was extracted with water and EA, the organic layers were combined and concentrated under reduced pressure to obtain the intermediate 2 (yield 86%).

##### Chasmanine

Intermediate 2 (0.48 mM) was dissolved in a solution of 5% NaOH/MeOH (20 mL). The reaction solution was stirred at 55 °C for 1 h. After cooling down the solution was concentrated under reduced pressure. The residue was dissolved in H_2_O (15 mL) and extracted with DCM (15 mL × 3). The organic layers were combined and concentrated under reduced pressure to obtain the Chasmanine (yield 90%).

#### General synthetic procedure for compounds 1–36

Chasmanine (0.5 mM) was dissolved in pyridine, and the corresponding acyl chloride (2.5 mM) was slowly added dropwise to the solution while stirring at room temperature, and the reaction was monitored by TLC. At the end of the reaction, the solution was concentrated under reduced pressure and the reside was dissolved in H_2_O and the mixture was treated with aq. NaHCO_3_ solution to adjust the pH to 10. The products were extracted with DCM (5 mL × 3). The combined organic layer was dried over anhydrous Na_2_SO_4_ and evaporated to give compounds **1–36** after purification by column chromatography over silica gel (yield 48% ~ 92%).

#### General synthetic procedure for compounds 37 ~ 38

Monoester derivative **18** or **33** was dissolved in acetic anhydride, *p*-toluenesulfonic acid as a catalyst, continuing stirring overnight at room temperature, and the reaction was monitored by TLC. At the end of the reaction, ammonia was used to adjust the pH to 9 ~ 10. Then the reaction solution was extracted with DCM (20 mL × 5), the organic layers were combined and concentrated under reduced pressure to obtain the compound **37** or **38** (yield 90% and 62%).

### Biological activity

#### In vivo antifeedant activity bioassays

The *Spodoptera exigua* (Hübner) were reared on cabbage foliage and maintained at 24 ± 1 °C and > 70% relative humidity with a photoperiod of 16:8 (L:D) in a growth chamber. The newly emerged third-instar larvae of *S. exigua* was used in the antifeedant bioassays. The antifeedant properties of compounds were evaluated using choice leaf-disc method^27^. Azadirachtin A, a commercially insecticide, was used as the positive control. The test compounds were dissolved in 10% acetone–deionized water solution (containing 0.2% Tween − 80) and the concentrations were adjusted to 0.5 mg/mL. Fresh cabbage leaves were cut into leaf discs (5 cm diameter) and treated on the upper surface with 15 μL of the test substance emulsions or deionized water containing acetone and Tween − 80 as a control. After air drying of leaves, two treated leaves and other two control leaves were alternately placed in 15 cm diameter petri dishes. Five healthy and starved 6 h instars were placed in each dish and allowed to feed in a growth chamber. Each treatment was repeated three times, and the areas of feeding were determined by coordinate paper chip method after 24 h. The refusal rate (FR) in each dish was determined using the equation:

$${\text{FR }} = \, \left( {{\text{CK }}{-}{\text{ T}}} \right) \, /{\text{ CK}} \times {1}00\%$$

CK: feeding area of leaves in negative control; T: feeding area of leaves in chemical treatment group.

Compounds with FR > 60% were tested in dose–response experiment to calculate their EC_50_ value (the effective dose for 50%) and 95% confidence interval using Origin 2019b. All the results with significant difference at P < 0.05.

#### In vitro antiproliferative activity on Sf9 cells

Sf9 cells in logarithmic growth phase were collected, and the concentration was adjusted to 8 × 10^4^ cells/mL; 100 μL of cell fluid was taken and added to a 96-well cell culture plate. Discarded the medium after cell adherence and 100 μL of the drug solution (treatment group) or 0.1% DMSO solution (control group) was added for treatment for 48 h. Also, the blank group, without cell culture medium, was set for zero adjustment. Each treatment was repeated 6 times. The cell viability was detected by MTT colorimetry. 20 μL of MMT solution (5 mg/mL) was added to each well and incubated for 4 h. Subsequently, the medium was removed, and the formazan crystals were dissolved with DMSO. The absorbance (OD) was measured at 492 nm and cell lethality was calculated. The cell lethality calculation formula is as follows:

$${\text{Inhibition }}\;{\text{rate }}\left( {{\text{IR }}\% } \right) \, = \, \left( {{\text{OD}}_{{{\text{control }}\;{\text{group}}}} - {\text{ OD}}_{{{\text{treatment}}\;{\text{ group}}}} } \right) \, / \, \left( {{\text{OD}}_{{{\text{control}}\;{\text{ group}}}} - {\text{ OD}}_{{{\text{blank}}\;{\text{ group}}}} } \right) \, \times {1}00\%$$

The median lethal concentration (LC_50_) value of Sf9 cells was obtained by GraphPad. Prism version 8.0. All the results with significant difference at P < 0.05.

#### Contact toxicity bioassay

Contact toxicity (measured as 24 h mortality) of compounds was determined by topical application to third-instar larvae of *Spodoptera exigua* (Hübner). Each larva was soaked in drug solution with concentration gradient for 10 s, and the surface solution was removed and air-dried. DMSO was used as the negative control and Cyhalothrin was used as the positive control. After the compounds were applied, ten larvae were transferred onto a leaf disc in a petri dish with moistened paper towels. Three replicates of ninety larvae each were used per treatment. The number of dead larvae was determined after 24 h. Larvae were considered dead if they did not respond to prodding with forceps.

$${\text{Percent}} \, {\text{mortality}} = \, \left( {{\text{P}}_{{\text{t}}} - {\text{ P}}_{0} } \right) \, / \, \left( {{1 } - {\text{ P}}_{0} } \right) \, \times {1}00 \, \%$$

Pt: deaths of treat group; P_0_: deaths of blank group.

Percent mortality was calculated and the lethal concentration causing 50% mortality (LC_50_ values) determined by Origin 2019b. All the results with significant difference at P < 0.05.

#### In vitro enzymes inhibitory activity

##### Enzyme extraction

Methods refer to the method of Liu et al.^28^. The third instar larvae of similar size of *S. exigua* were weighed after freezing rapidly at − 80 °C and added 5 volumes of normal saline (g: mL = 1: 5). The crude enzyme extract was obtained by homogenization at 0 °C, which fallowed by centrifugation at 12,000 rpm and 4 °C for 20 min. Supernatants were collected and dissolved in 2.5 volume of 0.1 M phosphate buffer (PBS, pH 7.5) as subsequent enzyme solution.

##### Acetylcholinesterase

Acetylcholinesterase (AChE) reaction was determined using the procedure of Shixing Miao et al.^29^. 140 μL phosphate buffer (0.1 M, pH 8.0), 10 μL test compound solution and 10 μL diluted enzyme solution were mixed and incubated 20 min at 30 °C. Then, 10 μL acetylthiocholine iodide (0.01 M) was added and incubated at 30 °C. After 20 min of treatment, 10 μL 5, 5′-dithiobis (2-nitrobenzoic acid) (0.01 M) was added to terminate the reaction. Absorbance was measured at 405 nm.

##### Mixed-functional oxidase

The *Mixed-Functional Oxidase* (MFO) inhibitory assay were performed using Shang’s method, with some modifications^30^. 50 μL diluted subsequent enzyme solution and 50 *μ*L test compound solution was mixed and incubated 20 min at 30 °C. 50 μL paranitroanisole (0.05 M) was added and incubation for 20 min at 30 °C, then measured absorbance at 405 nm.

##### Glutathione S-transferase

Glutathione S-transferase (GST) activity was determined by a modified procedure of Kumrungsee^31^. 50 μL subsequent diluted enzyme solution and 50 μL test compound solution was mixed and incubated 20 min at 30 °C. 50 μL glutathione (0.01 M) and 50 μL test compound solution (0.01 M) was added, and the absorbance was measured at 340 nm.

##### Carboxylesterase

Carboxylesterase (CES) reaction was determined by a modified procedure of Nobsathian^32^. 50 μL diluted enzyme solution and 50 μL test compound solution was mixed and incubated 20 min at 30 °C. Then, 50 μL *α* − naphthylacetate (0.01 M) was added and incubation. After 20 min, 50 μL diazoblue lauryl sulfate solution (5% Sodium dodecyl sulfate/1% Diazo Blue B = 5/2) was mixed. Absorbance was measured at 600 nm.

$${\text{Inhibition}}\;{\text{ rate }}\left( {{\text{IR }}\% } \right) \, = \left( {{\text{OD}}_{{{\text{control}}\;{\text{ group}}}} - {\text{ OD}}_{{{\text{treatment}}\;{\text{ group}}}} } \right) \, / \, \left( {{\text{OD}}_{{{\text{control }}\;{\text{group}}}} - {\text{ OD}}_{{{\text{blank }}\;{\text{group}}}} } \right) \, \times {1}00 \, \%$$

The inhibition rate of each enzyme was measured with Azadirachtin as a control. Each treatment of AChE, MFO, GST, CES were assayed in triplicate.

#### Molecular docking simulation

The ligand–protein docking was performed using Discovery Studio Client 2019 (DS 2019), which predicated the active site of CES and the bonding pose with compound **33**. The 3D model of CES (PDB ID: 5TYJ) was obtained from Protein Date Bank at http://www.rcsb.orgl/, and molecule structure of compounds **33** was constructed by Chemdraw. Protein was protonated as well as defining the site of binding by DS 2019, and the compound **33** was energy minimized by optimization. Finally, the molecular docking was achieved thought function CDOCKER protocol.

#### Sf9 cells morphology observation

Sf9 cells in logarithmic growth phase were collected, and the concentration was adjusted to 1 × 10^5^ cells/mL, 4 mL of cell fluid was taken and added to a 6-well cell culture plate. The medium was aspirated, and 4 mL of the drug solution to be tested (treatment group, 12.87 μM) or Azadirachtin A solution (positive control group, 4.92 μM) or blank solution (negative control group) was added for treatment for 0 h, 12 h, 24 h and 36 h. Than the cell morphology was observed under a high magnification microscope.

#### Fluorescence staining

Sf9 cells were seeded on cover slips before being cultured in 24-well plates for 24 h, and then exposed to compound **10** (15 μM) for 24 h. Discarded the culture media, and the uncovered cells were fixed in 4% paraformaldehyde for 30 min and washed with PBS twice. Finally, using the One Step TUNEL Apoptosis Assay Kit to detect apoptotic cells according to the manufacturer’s instructions.

#### DNA ladder assay

After treated with compounds for 24 h, collecting Sf9 cells by centrifugation at 1000 rpm for 5 min. Then resuspending pellets by PBS and DNA fragmentation was extracted using the universal genomic DNA purification mini spin kit (Shanghai, Beyotime). DNA electrophoresis was performed on 2% (w/v) agarose gels at constant voltage 100 V for 20 min. The image was recorded by scanning on a multipurpose imager.

## Supplementary Information

Supplementary Information 1.

Supplementary Information 2.

## Data Availability

The original data and supporting information of this study are available at https://www.nature.com/srep.

## References

[1] Kushwaha UKS (2022). A cost-efficient and alternative technique of managing fall armyworm *Spodoptera frugiperda* (JE Smith) larvae in maize crop. Sci. Rep..

[2] Horikoshi RM (2022). Afidopyropen, a novel insecticide originating from microbial secondary extracts. Sci. Rep..

[3] Van Den Berg H (2021). Recent trends in global insecticide use for disease vector control and potential implications for resistance management. Sci. Rep..

[4] Medina-Romero YM (2021). Essential oils of *Bursera morelensis* and *Lippia graveolens* for the development of a new biopesticides in postharvest control. Sci. Rep..

[5] Zhao L (2017). Advances and prospects in biogenic substances against plant virus: A review. Pestic. Biochem. Phys..

[6] Souto AL (2021). Plant-derived pesticides as an alternative to pest management and sustainable agricultural production: Prospects, applications and challenges. Molecules.

[7] Gliszczyńska A (2021). Synthesis of novel phytol-derived *γ*-butyrolactones and evaluation of their biological activity. Sci. Rep..

[8] Liu F (2017). Cytotoxicity of *Aconitum alkaloid* and its interaction with calf thymus DNA by multi-spectroscopic techniques. Sci. Rep..

[9] Thawabteh AM (2021). Classification, toxicity and bioactivity of natural diterpenoid alkaloids. Molecules.

[10] Wan LX (2021). Isolation, structure elucidation, semi-synthesis, and structural modification of C_19_-diterpenoid alkaloids from *Aconitum **apetalum* and their neuroprotective activities. J. Nat. Prod..

[11] Zhang C (2018). A comparison of the effects of agricultural pesticide uses on peripheral nerve conduction in China. Sci. Rep..

[12] Liu W (2022). Synthesis and biological activity of novel hydantoin cyclohexyl sulfonamide derivatives as potential antimicrobial agents in agriculture. Pest. Manag. Sci..

[13] Zhang JF (2017). Diterpenoid alkaloids from two *Aconitum* species with antifeedant activity against *Spodoptera exigua*. J. Nat. Prod..

[14] Song ZY (2021). Semi-synthetic chasmanthinine analogues with antifeedant effects against *Spodoptera exigua*. Heterocycles.

[15] Pelletier SW, Djarmati Z, Lajsic S (1974). Structure of neoline, chasmanine, and homochasmanine. J. Am. Chem. Soc..

[16] Laus G (2001). Biological activities of natural halogen compounds. Stud. Nat. Prod. Chem..

[17] Turabekova MA, Rasulev BF (2005). QSAR analysis of the structure: Toxicity relationship of *Aconitum* and *Delphinium* diterpene alkaloids. Chem. Nat. Compds..

[18] Xu Z (2017). Pyrethrum-extract induced autophagy in insect cells: A new target?. Pestic. Biochem. Physiol..

[19] Liang X, Gao Y, Luan S (2018). Two decades of advances in diterpenoid alkaloids with cytotoxicity activities. RSC. Adv..

[20] He H (2020). Synthesis, characterization of two matrine derivatives and their cytotoxic effect on Sf9 cell of *Spodoptera frugiperda*. Sci. Rep..

[21] Mak M (2021). Triangulation of methods using insect cell lines to investigate insecticidal mode-of-action. Pest. Manag. Sci..

[22] Pan L (2016). Antifeedant activity of Ginkgo biloba secondary metabolites against Hyphantria cunea larvae: Mechanisms and applications. PLoS ONE.

[23] Wang Z (2015). Azadirachtin-induced apoptosis involves lysosomal membrane permeabilization and cathepsin L release in *Spodoptera frugiperda* Sf9 cells. Int. J. Biochem. Cell. B..

[24] Gong JP, Traganos F, Darzynkiewicz Z (1994). A selective procedure for DNA extraction from apoptotic cells applicable for gel electrophoresis and flow cytometry. Anal. Biochem..

[25] Yooboon T (2019). Cytotoxic effects of *β*-asarone on Sf9 insect cells. Arch. Insect. Biochem..

[26] Kumarswamy R (2009). Mitochondrial regulation of insect cell apoptosis: evidence for permeability transition pore-independent cytochrome-c release in the Lepidopteran Sf9 cells. Int. J. Biochem. Cell. B..

[27] Gonzalez-Coloma A (1995). Antifeedant and toxic effects of sesquiterpenes from *Senecio palmensis* to colorado potato beetle. J. Chem. Ecol..

[28] Liu C (2019). Secondary metabolites from *Solanum rostratum* and their antifeedant defense mechanisms against *Helicoverpa armigera*. J. Agric. Food. Chem..

[29] Miao SX (2021). Pd-catalyzed direct diversification of natural anti-alzheimer’s disease drug: Synthesis and biological evaluation of *N*-aryl huperzine A analogues. J. Nat. Prod..

[30] Shang CC, Soderlund DM (1984). Monooxygenase activity of tobacco budworm (Heliothis virescens F.) larvae: Tissue distribution and optimal assay conditions for the gut activity. Comp. Biochem. Phys. B..

[31] Kumrungsee N (2014). Toxicity of essential oil compounds against diamondback moth, *Plutella xylostella*, and their impact on detoxification enzyme activities. J. Pest. Sci..

[32] Nobsathian S (2018). Larvicidal effect of compounds isolated from *Maerua siamensis* (Capparidaceae) against *Aedes aegypti* (Diptera: Culicidae) larvae. Chem. Biol. Technol. Agric..

