# Supplementary material for: Design, synthesis and insecticidal activity and mechanism research of Chasmanthinine derivatives
Source: Sci Rep. 2022 Sep 10;12:15290. doi: 10.1038/s41598-022-19523-8 (PMC9464227; doi:10.1038/s41598-022-19523-8)
Supplement: Supplementary file 1 — Supplementary Information 1. [file 41598_2022_19523_MOESM1_ESM.pdf]

Supplementary Information

for

Design, synthesis and insecticidal activity and mechanism research of Chasmanthinine derivatives

Ziyu Song,[a‡] Xiangyu Li,[a‡] Ke Xu,[a] Guoqing Sun,[a] Liu Yang,[a] Linyu Huang,[a] Junqi Liu,[a] Pengyuan Yin,[a] Shuai Huang,[a] Feng Gao,[a] Xianli Zhou[a,b*] and Lin Chen[a*]

[a]School of Life Science and Engineering, Southwest Jiaotong University, Chengdu 610031, Sichuan, P.R. China.

[b]Affiliated Hospital of Southwest Jiaotong University ＆ The Third People Hospital of Chengdu, Chengdu 610031, Sichuan, P.R. China.

*Corresponding Author: Phone +86-28-887603201; E-mail: zhouxl@swjtu.edu.cn; linch@swjtu.edu.cn.

[‡] These authors contributed equally to this work and should be considered co-first authors.

Content

Spectroscopic data for the synthesized compounds: 6

Data of HR-ESI-MS: 15

figure 1 ^{1}H NMR (400 MHz) spectrum of Chasmanine in $CDCl_3$ 56

figure 2 ^{13}C NMR (100 MHz) spectrum of Chasmanine in $CDCl_3$ 56

figure 3 HR-ESI-MS data of Chasmanine 57

figure 4 ^{1}H NMR (400 MHz) spectrum of compound 1 in $CDCl_3$ 57

figure 5 ^{13}C NMR (100 MHz) spectrum of compound 1 in $CDCl_3$ 58

figure 6 HR-ESI-MS data of Compound 1 58

figure 7 ^{1}H NMR (400 MHz) spectrum of compound 2 in $CDCl_3$ 59

figure 8 ^{13}C NMR (100 MHz) spectrum of compound 2 in $CDCl_3$ 59

figure 9 HR-ESI-MS data of Compound 2 60

figure 10 ^{1}H NMR (400 MHz) spectrum of compound 3 in $CDCl_3$ 60

figure 11 ^{13}C NMR (100 MHz) spectrum of compound 3 in $CDCl_3$ 61

figure 12 HR-ESI-MS data of Compound 3 61

figure 13 ^{1}H NMR (400 MHz) spectrum of compound 4 in $CDCl_3$ 62

figure 14 ^{13}C NMR (100 MHz) spectrum of compound 4 in $CDCl_3$ 62

figure 15 HR-ESI-MS data of Compound 4 63

figure 16 ^{1}H NMR (400 MHz) spectrum of compound 5 in $CDCl_3$ 63

figure 17 ^{13}C NMR (100 MHz) spectrum of compound 5 in $CDCl_3$ 64

figure 18 HR-ESI-MS data of Compound 5 64

figure 19 ^{1}H NMR (400 MHz) spectrum of compound 6 in $CDCl_3$ 65

figure 20 ^{13}C NMR (100 MHz) spectrum of compound 6 in $CDCl_3$ 65

figure 21 HR-ESI-MS data of Compound 6 66

figure 22 ^{1}H NMR (400 MHz) spectrum of compound 7 in $CDCl_3$ 66

figure 23 ^{13}C NMR (100 MHz) spectrum of compound 7 in $CDCl_3$ 67

figure 24 HR-ESI-MS data of Compound 7 67

figure 25 ^{1}H NMR (400 MHz) spectrum of compound 8 in $CDCl_3$ 68

figure 26 ^{13}C NMR (100 MHz) spectrum of compound 8 in $CDCl_3$ 68

figure 27 HR-ESI-MS data of Compound 8 69

figure 28 ^{1}H NMR (400 MHz) spectrum of compound 9 in $CDCl_3$ 69

figure 29 ^{13}C NMR (100 MHz) spectrum of compound 9 in $CDCl_3$ 70
figure 30 HR-ESI-MS data of Compound 9 70
figure 31 ^{1}H NMR (400 MHz) spectrum of compound 10 in $CDCl_3$ 71
figure 32 ^{13}C NMR (100 MHz) spectrum of compound 10 in $CDCl_3$ 71
figure 33 HR-ESI-MS data of Compound 10 72
figure 34 ^{1}H NMR (400 MHz) spectrum of compound 11 in $CDCl_3$ 72
figure 35 ^{13}C NMR (100 MHz) spectrum of compound 11 in $CDCl_3$ 73
figure 36 HR-ESI-MS data of Compound 11 73
figure 37 ^{1}H NMR (400 MHz) spectrum of compound 12 in $CDCl_3$ 74
figure 38 ^{13}C NMR (100 MHz) spectrum of compound 12 in $CDCl_3$ 74
figure 39 HR-ESI-MS data of Compound 12 75
figure 40 ^{1}H NMR (400 MHz) spectrum of compound 13 in $CDCl_3$ 75
figure 41 ^{13}C NMR (100 MHz) spectrum of compound 13 in $CDCl_3$ 76
figure 42 HR-ESI-MS data of Compound 13 76
figure 43 ^{1}H NMR (400 MHz) spectrum of compound 14 in $CDCl_3$ 77
figure 44 ^{13}C NMR (100 MHz) spectrum of compound 14 in $CDCl_3$ 77
figure 45 HR-ESI-MS data of Compound 14 78
figure 46 ^{1}H NMR (400 MHz) spectrum of compound 15 in $CDCl_3$ 78
figure 47 ^{13}C NMR (100 MHz) spectrum of compound 15 in $CDCl_3$ 79
figure 48 HR-ESI-MS data of Compound 15 79
figure 49 ^{1}H NMR (400 MHz) spectrum of compound 16 in $CDCl_3$ 80
figure 50 ^{13}C NMR (100 MHz) spectrum of compound 16 in $CDCl_3$ 80
figure 51 HR-ESI-MS data of Compound 16 81
figure 52 ^{1}H NMR (400 MHz) spectrum of compound 17 in $CDCl_3$ 81
figure 53 ^{13}C NMR (100 MHz) spectrum of compound 17 in $CDCl_3$ 82
figure 54 HR-ESI-MS data of Compound 17 82
figure 55 ^{1}H NMR (400 MHz) spectrum of compound 18 in $CDCl_3$ 83
figure 56 ^{13}C NMR (100 MHz) spectrum of compound 18 in $CDCl_3$ 83
figure 57 HR-ESI-MS data of Compound 18 84
figure 58 ^{1}H NMR (400 MHz) spectrum of compound 19 in $CDCl_3$ 84
figure 59 ^{13}C NMR (100 MHz) spectrum of compound 19 in $CDCl_3$ 85
figure 60 HR-ESI-MS data of Compound 19 85

figure 61 ^{1}H NMR (400 MHz) spectrum of compound 20 in $CDCl_3$ 86
figure 62 ^{13}C NMR (100 MHz) spectrum of compound 20 in $CDCl_3$ 86
figure 63 HR-ESI-MS data of Compound 20 87
figure 64 ^{1}H NMR (400 MHz) spectrum of compound 21 in $CDCl_3$ 87
figure 65 ^{13}C NMR (100 MHz) spectrum of compound 21 in $CDCl_3$ 88
figure 66 HR-ESI-MS data of Compound 21 88
figure 67 ^{1}H NMR (400 MHz) spectrum of compound 22 in $CDCl_3$ 89
figure 68 ^{13}C NMR (100 MHz) spectrum of compound 22 in $CDCl_3$ 89
figure 69 HR-ESI-MS data of Compound 22 90
figure 70 ^{1}H NMR (400 MHz) spectrum of compound 23 in $CDCl_3$ 90
figure 71 ^{13}C NMR (100 MHz) spectrum of compound 23 in $CDCl_3$ 91
figure 72 HR-ESI-MS data of Compound 23 91
figure 73 ^{1}H NMR (400 MHz) spectrum of compound 24 in $CDCl_3$ 92
figure 74 ^{13}C NMR (100 MHz) spectrum of compound 24 in $CDCl_3$ 92
figure 75 HR-ESI-MS data of Compound 24 93
figure 76 ^{1}H NMR (400 MHz) spectrum of compound 25 in $CDCl_3$ 93
figure 77 ^{13}C NMR (100 MHz) spectrum of compound 25 in $CDCl_3$ 94
figure 78 HR-ESI-MS data of Compound 25 94
figure 79 ^{1}H NMR (400 MHz) spectrum of compound 26 in $CDCl_3$ 95
figure 80 ^{13}C NMR (100 MHz) spectrum of compound 26 in $CDCl_3$ 95
figure 81 HR-ESI-MS data of Compound 26 96
figure 82 ^{1}H NMR (400 MHz) spectrum of compound 27 in $CDCl_3$ 96
figure 83 ^{13}C NMR (100 MHz) spectrum of compound 27 in $CDCl_3$ 97
figure 84 HR-ESI-MS data of Compound 27 97
figure 85 ^{1}H NMR (400 MHz) spectrum of compound 28 in $CDCl_3$ 98
figure 86 ^{13}C NMR (100 MHz) spectrum of compound 28 in $CDCl_3$ 98
figure 87 HR-ESI-MS data of Compound 28 99
figure 88 ^{1}H NMR (400 MHz) spectrum of compound 29 in $CDCl_3$ 99
figure 89 ^{13}C NMR (100 MHz) spectrum of compound 29 in $CDCl_3$ 100
figure 90 HR-ESI-MS data of Compound 29 100
figure 91 ^{1}H NMR (400 MHz) spectrum of compound 30 in $CDCl_3$ 101
figure 92 ^{13}C NMR (100 MHz) spectrum of compound 30 in $CDCl_3$ 101

figure 93 HR-ESI-MS data of Compound 30 102
figure 94 ^{1}H NMR (400 MHz) spectrum of compound 31 in $CDCl_3$ 102
figure 95 ^{13}C NMR (100 MHz) spectrum of compound 31 in $CDCl_3$ 103
figure 96 HR-ESI-MS data of Compound 31 103
figure 97 ^{1}H NMR (400 MHz) spectrum of compound 32 in $CDCl_3$ 104
figure 98 ^{13}C NMR (100 MHz) spectrum of compound 32 in $CDCl_3$ 104
figure 99 HR-ESI-MS data of Compound 32 105
figure 100. ^{1}H NMR (400 MHz) spectrum of compound 33 in $CDCl_3$ 105
figure 101 ^{13}C NMR (100 MHz) spectrum of compound 33 in $CDCl_3$ 106
figure 102 HR-ESI-MS data of Compound 33 106
figure 103 ^{1}H NMR (400 MHz) spectrum of compound 34 in $CDCl_3$ 107
figure 104 ^{13}C NMR (100 MHz) spectrum of compound 34 in $CDCl_3$ 107
figure 105 HR-ESI-MS data of Compound 34 108
figure 106 ^{1}H NMR (400 MHz) spectrum of compound 35 in $CDCl_3$ 108
figure 107 ^{13}C NMR (100 MHz) spectrum of compound 35 in $CDCl_3$ 109
figure 108 HR-ESI-MS data of Compound 35 109
figure 109 ^{1}H NMR (400 MHz) spectrum of compound 36 in $CDCl_3$ 110
figure 110 ^{13}C NMR (100 MHz) spectrum of compound 36in $CDCl_3$ 110
figure 111 HR-ESI-MS data of Compound 36 111
figure 112 ^{1}H NMR (400 MHz) spectrum of compound 37 in $CDCl_3$ 111
figure 113 ^{13}C NMR (100 MHz) spectrum of compound 37 in $CDCl_3$ 112
figure 114 HR-ESI-MS data of Compound 37 112
figure 115 ^{1}H NMR (400 MHz) spectrum of compound 38 in $CDCl_3$ 113
figure 116 ^{13}C NMR (100 MHz) spectrum of compound 38 in $CDCl_3$ 113
figure 117 HR-ESI-MS data of Compound 38 114
figure 118 ^{1}H NMR (400 MHz) spectrum of Intermediate 1 in $CDCl_3$ 114
figure 119 ^{13}C NMR (100 MHz) spectrum of Intermediate 1 in $CDCl_3$ 115
figure 121 ^{1}H NMR (400 MHz) spectrum of Intermediate 2 in $CDCl_3$ 116
figure 122 ^{13}C NMR (100 MHz) spectrum of Intermediate 2 in $CDCl_3$ 116
figure 123 HR-ESI-MS data of Intermediate 2 117

Spectroscopic data for the synthesized compounds:

Compound 1. (72% yield, white amorphous powder, mp, 145.8-147.0 °C), ^{1}H NMR (400 MHz, $CDCl_3$) δ 4.80 (t, *J* = 5.1 Hz, 1H), 4.10 (d, *J* = 7.0 Hz, 1H), 3.69 (d, *J* = 8.4 Hz, 1H), 3.29 (s, 3H), 3.27 (s, 3H), 3.21 (s, 3H), 3.18 (s, 3H), 2.99 (dd, *J* = 10.2, 6.4 Hz, 1H), 2.90 (s, 1H), 1.11 (t, *J* = 7.6 Hz, 3H), 1.04 (t, *J* = 7.2 Hz, 3H). ^{13}C NMR (100 MHz, $CDCl_3$) δ 173.9, 85.6, 82.7, 81.9, 80.8, 77.1, 73.7, 61.9, 59.3, 57.6, 56.2, 56.1, 54.0, 53.3, 50.3, 49.9, 49.2, 47.2, 44.9, 41.4, 39.3, 36.0, 35.1, 29.3, 27.9, 26.3, 13.7, 9.1. HRMS (ESI): *m/z* [M + H] $^{+}$ calcd for $C_{28}H_{45}NO_7$: 508.3281, found: 508.3274.

Compound 2. (80% yield, white amorphous powder, mp, 149.3-152.0 °C), ^{1}H NMR (400 MHz, $CDCl_3$) δ 4.82 (t, *J* = 5.0 Hz, 1H), 4.11 (d, *J* = 6.9 Hz, 1H), 3.70 (d, *J* = 8.4 Hz, 1H), 3.31 (s, 3H), 3.29 (s, 3H), 3.23 (s, 3H), 3.20 (s, 3H), 3.04 – 2.98 (m, 1H), 2.91 (s, 1H), 1.06 (t, *J* = 7.1 Hz, 3H), 0.94 (t, *J* = 7.4 Hz, 3H). ^{13}C NMR (100 MHz, $CDCl_3$) δ 173.1, 85.6, 82.8, 81.9, 80.8, 77.1, 73.8, 62.0, 59.3, 57.7, 56.2, 56.1, 54.1, 53.3, 50.4, 49.9, 49.2, 47.3, 44.9, 41.5, 39.3, 36.6, 36.1, 29.4, 27.3, 26.4, 18.3, 13.8, 13.7. HRMS (ESI): *m/z* [M + H] $^{+}$ calcd for $C_{29}H_{47}NO_7$: 522.3430, found: 522.3431.

Compound 3. (83% yield, white amorphous powder, mp, 151.4-152.9 °C), ^{1}H NMR (400 MHz, $CDCl_3$) δ 5.83 (dq, *J* = 15.5, 1.7 Hz, 1H), 4.91 (t, *J* = 4.6 Hz, 1H), 4.11 (d, *J* = 6.8 Hz, 1H), 3.70 (d, *J* = 8.5 Hz, 1H), 3.31 (s, 3H), 3.29 (s, 3H), 3.23 (s, 3H), 3.21 (s, 3H), 3.02 (dd, *J* = 10.2, 6.4 Hz, 1H), 2.92 (s, 1H), 1.86 (dd, *J* = 6.9, 1.7 Hz, 5H), 1.06 (t, *J* = 7.1 Hz, 3H). ^{13}C NMR (100 MHz, $CDCl_3$) δ 166.1, 145.0, 122.9, 85.6, 82.9, 82.0, 80.8, 76.8, 73.9, 62.0, 59.3, 57.7, 56.3, 56.1, 54.1, 53.5, 50.4, 49.9, 49.2, 47.2, 45.1, 41.5, 39.3, 36.6, 35.1, 29.4, 26.4, 18.2, 13.7. HRMS (ESI): *m/z* [M + H] $^{+}$ calcd for $C_{29}H_{45}NO_7$: 520.3259, found: 520.3274.

Compound 4. (75% yield, white amorphous powder, mp, 156.2-157.8 °C), ^{1}H NMR (400 MHz, $CDCl_3$) δ 4.81 (t, *J* = 4.9 Hz, 1H), 4.11 (d, *J* = 6.9 Hz, 1H), 3.70 (d, *J* = 8.4 Hz, 1H), 3.31 (s, 3H), 3.29 (s, 3H), 3.22 (s, 3H), 3.19 (s, 3H), 3.01 (dd, *J* = 10.2, 6.4 Hz, 1H), 2.90 (s, 1H), 1.05 (t, *J* = 7.1 Hz, 3H), 0.89 (t, *J* = 7.4 Hz, 3H). ^{13}C NMR (100 MHz, $CDCl_3$) δ 173.3, 85.6, 82.8, 81.9, 80.8, 77.1, 73.8, 62.0, 59.3, 57.7, 56.3, 56.1, 54.0, 53.3, 50.3, 49.9, 49.2, 47.3, 44.9, 41.5, 39.3, 36.1, 35.1, 34.4, 29.3, 26.9, 26.4, 22.3, 13.8, 13.7 HRMS (ESI): *m/z* [M + H] $^{+}$ calcd for $C_{30}H_{49}NO_7$: 536.3591, found: 536.3587.

Compound 5. (77% yield, white amorphous powder, mp, 158.2-160.0 °C), ^{1}H NMR (400 MHz, $CDCl_3$) δ 4.77 (t, *J* = 5.4 Hz, 1H), 4.07 (d, *J* = 6.9 Hz, 1H), 3.66 (d, *J* = 10.1 Hz, 1H), 3.27 (s, 3H), 3.24 (s, 3H), 3.18 (s, 3H), 3.16 (s, 3H), 3.01 – 2.94 (m, 1H), 2.86 (s, 1H), 1.01 (t, *J* = 7.1 Hz, 3H), 0.82 (t, *J* = 7.0 Hz, 3H). ^{13}C NMR (100 MHz, $CDCl_3$) δ 173.2, 85.5, 82.7, 81.8, 80.7, 77.0, 73.6, 61.8, 59.2, 57.6, 56.1, 56.0, 54.0, 53.3, 50.3, 49.8, 49.1, 47.2, 44.8, 41.4, 39.3, 36.1, 35.0, 34.6, 31.5, 29.3, 28.8, 26.3, 24.7, 22.5, 14.0, 13.6. HRMS (ESI): *m/z* [M + H] $^{+}$ calcd for $C_{32}H_{53}NO_7$: 564.3893, found: 564.3900.

Compound 6. (65% yield, white amorphous powder, mp, 154.7-155.3 °C), ^{1}H NMR (400 MHz, $CDCl_3$) δ 4.79 (t, *J* = 5.1 Hz, 1H), 4.10 (d, *J* = 7.0 Hz, 1H), 3.69 (d, *J* = 8.4 Hz, 1H), 3.30 (s, 3H), 3.27 (s, 3H), 3.21 (s, 3H), 3.17 (s, 3H), 3.04 – 2.96 (m, 1H), 2.89 (s, 1H), 1.15 (dd, *J* = 7.0, 3.1 Hz, 6H), 1.04 (t, *J* = 7.1 Hz, 3H). ^{13}C NMR (100 MHz, $CDCl_3$) δ 176.3, 85.6, 82.7, 81.9, 80.8, 77.2, 73.8, 61.9, 59.3, 57.6, 56.2, 55.9, 54.0, 53.3, 50.3, 49.9, 49.2, 47.2, 44.9, 41.5, 39.3, 36.2, 35.1, 34.3, 29.3, 26.4, 19.1, 19.0, 13.7. HRMS (ESI): *m/z* $[M + H]^+$ calcd for $C_{29}H_{47}NO_7$: 522.3430, found: 522.3431.

Compound 7. (72% yield, white amorphous powder, mp, 151.6-153.2 °C), ^{1}H NMR (400 MHz, $CDCl_3$) δ 4.79 (t, *J* = 5.1 Hz, 1H), 4.11 (d, *J* = 6.9 Hz, 1H), 3.69 (d, *J* = 8.4 Hz, 1H), 3.30 (s, 3H), 3.28 (s, 3H), 3.22 (s, 3H), 3.19 (s, 3H), 3.04 – 2.96 (m, 1H), 2.89 (s, 1H), 1.05 (d, *J* = 7.1 Hz, 3H), 1.02 (s, 9H). ^{13}C NMR (100 MHz, $CDCl_3$) δ 171.7, 85.6, 82.7, 81.9, 80.8, 76.9, 73.7, 61.9, 59.3, 57.6, 56.2, 56.0, 54.1, 53.2, 50.3, 49.9, 49.2, 48.2, 47.4, 44.9, 41.5, 39.3, 36.1, 35.1, 30.6, 29.7 (2C), 29.4, 26.4, 13.7. HRMS (ESI): *m/z* $[M + H]^+$ calcd for $C_{31}H_{51}NO_7$: 550.3740, found: 550.3744.

Compound 8. (87% yield, white amorphous powder, mp, 157.9-160.2 °C), ^{1}H NMR (400 MHz, $CDCl_3$) δ 4.78 (t, *J* = 5.1 Hz, 1H), 4.10 (d, *J* = 7.0 Hz, 1H), 3.69 (d, *J* = 8.5 Hz, 1H), 3.30 (s, 3H), 3.27 (s, 3H), 3.21 (s, 3H), 3.17 (s, 3H), 3.00 (dd, *J* = 10.1, 6.6 Hz, 1H), 2.89 (s, 1H), 2.61 (s, 1H), 1.04 (t, *J* = 7.1 Hz, 3H). ^{13}C NMR (100 MHz, $CDCl_3$) δ 175.3, 85.5, 82.7, 81.9, 80.8, 77.1, 73.8, 61.9, 59.3, 57.6, 56.2, 55.9, 54.0, 53.2, 50.3, 49.9, 49.2, 47.2, 44.9, 43.5, 41.5, 39.3, 36.1, 35.1, 29.3, 29.2, 29.1, 26.4, 25.8, 25.6, 13.7. HRMS (ESI): *m/z* $[M + H]^+$ calcd for $C_{32}H_{51}NO_7$: 562.3749, found: 562.3744.

Compound 9. (92% yield, white amorphous powder, mp, 145.8-147.0 °C), ^{1}H NMR (400 MHz, $CDCl_3$) δ 4.84 (t, *J* = 5.0 Hz, 1H), 4.11 (d, *J* = 6.9 Hz, 1H), 3.70 (d, *J* = 8.5 Hz, 1H), 3.31 (s, 3H), 3.29 (s, 3H), 3.22 (s, 3H), 3.21 (s, 3H), 3.04 – 2.97 (m, 1H), 2.91 (s, 1H), 1.06 (t, *J* = 7.2 Hz, 3H), 1.03 – 0.96 (m, 2H). ^{13}C NMR (100 MHz, $CDCl_3$) δ 174.4, 85.6, 82.8, 81.9, 80.8, 77.1, 73.8, 62.0, 59.3, 57.7, 56.2, 56.1, 54.1, 53.5, 50.4, 49.9, 49.2, 47.2, 45.0, 41.5, 39.3, 36.3, 35.1, 29.3, 26.4, 13.7, 13.4, 8.4, 8.3. HRMS (ESI): *m/z* $[M + H]^+$ calcd for $C_{29}H_{46}NO_7$: 520.3288, found: 520.3274.

Compound 10. (77% yield, yellow oil), ^{1}H NMR (400 MHz, $CDCl_3$) δ 4.79 (t, *J* = 5.0 Hz, 1H), 4.10 (d, *J* = 6.9 Hz, 1H), 3.69 (d, *J* = 8.4 Hz, 1H), 3.29 (s, 3H), 3.27 (s, 3H), 3.21 (s, 3H), 3.18 (s, 3H), 2.99 (dd, *J* = 10.1, 6.4 Hz, 1H), 2.89 (s, 1H), 1.22 (s, 32H), 1.04 (t, *J* = 7.1 Hz, 3H), 0.87 – 0.82 (m, 5H). ^{13}C NMR (101 MHz, CDC13) δ 173.2, 85.6, 82.8, 81.9, 80.8, 77.1, 73.7, 61.9, 59.2, 57.6, 56.2, 56.0, 54.0, 53.3, 50.3, 49.9, 49.2, 47.3, 44.9, 41.5, 39.3, 36.1, 35.1, 34.6, 32.0, 29.8, 29.8, 29.7, 29.7, 29.6, 29.5, 29.4, 29.4, 29.3, 29.3, 29.2, 26.4, 24.8, 22.8, 14.2, 13.7. HRMS (ESI): *m/z* $[M + H]^+$ calcd for $C_{41}H_{71}NO_7$: 690.5314, found: 690.5309.

Compound 11. (79% yield, yellow oil), ^{1}H NMR (400 MHz, $CDCl_3$) δ 4.72 (t, *J* = 5.1 Hz, 1H), 4.11 (d, *J* = 6.9 Hz, 1H), 4.06 (d, *J* = 6.6 Hz, 1H), 3.70 (d, *J* = 8.5 Hz, 1H), 3.30 (s, 3H), 3.28 (s, 3H), 3.22 (s, 6H), 3.00 (dd, *J* = 10.3, 6.4 Hz, 1H), 2.90 (s, 1H), 1.05 (t, *J* = 7.1 Hz, 3H), 0.92 (t, *J* = 7.4 Hz, 3H). ^{13}C NMR (100 MHz, $CDCl_3$) δ 154.6,

85.6, 82.7, 81.8, 80.8, 80.1, 73.5, 69.6, 61.9, 59.3, 57.7, 56.2 (2C), 54.0, 53.5, 50.4, 49.9, 49.2, 47.3, 44.8, 41.2, 39.3, 36.5, 35.1, 29.2, 26.4, 22.1, 13.7, 10.2. HRMS (ESI): *m/z* [M + H] $^+$ calcd for $C_{29}H_{47}NO_8$: 538.3384, found: 538.3380.

Compound 12. (69% yield, white amorphous powder, mp, 162.5-163.8 °C), ^{1}H NMR (400 MHz, $CDCl_3$) δ 4.74 (t, *J* = 5.0 Hz, 1H), 4.12 (td, *J* = 6.8, 2.4 Hz, 3H), 3.72 (d, *J* = 8.4 Hz, 1H), 3.32 (s, 3H), 3.30 (s, 3H), 3.24 (s, 6H), 3.02 (dd, *J* = 10.3, 6.5 Hz, 1H), 2.92 (s, 1H), 1.26 (d, *J* = 12.6 Hz, 1H), 1.06 (t, *J* = 7.1 Hz, 3H), 0.93 (t, *J* = 7.4 Hz, 3H). ^{13}C NMR (100 MHz, $CDCl_3$) δ 154.7, 85.7, 82.8, 81.9, 80.9, 80.2, 73.6, 67.9, 62.0, 59.3, 57.7, 56.3, 54.1, 53.5, 50.4, 50.0, 49.3, 47.4, 44.9, 41.2, 39.4, 36.6, 35.2, 30.8, 29.2, 26.5, 19.0, 13.8, 13.8. HRMS (ESI): *m/z* [M + H] $^+$ calcd for $C_{30}H_{49}NO_8$: 552.3549, found: 552.3536.

Compound 13. (85% yield, white amorphous powder, mp, 164.8-165.9 °C), ^{1}H NMR (400 MHz, $CDCl_3$) δ 4.72 (t, *J* = 5.1 Hz, 1H), 4.14 – 4.06 (m, 3H), 3.70 (d, *J* = 8.4 Hz, 1H), 3.30 (s, 3H), 3.28 (s, 3H), 3.22 (s, 6H), 3.04 – 2.97 (m, 1H), 2.90 (s, 1H), 1.05 (t, *J* = 7.1 Hz, 3H), 0.93 – 0.84 (m, 4H). ^{13}C NMR (100 MHz, $CDCl_3$) δ 154.6, 85.6, 82.8, 81.8, 80.8, 80.1, 73.5, 68.1, 61.9, 59.3, 57.7, 56.2, 54.1, 53.5, 50.4, 49.9, 49.2, 47.3, 44.9, 41.2, 39.3, 36.5, 35.1, 29.2, 28.5, 27.9, 26.4, 22.4, 14.0, 13.7. HRMS (ESI): *m/z* [M + H] $^+$ calcd for $C_{31}H_{51}NO_8$: 566.3677, found: 566.3693.

Compound 14. (58% yield, white amorphous powder, mp, 161.3-162.5 °C), ^{1}H NMR (400 MHz, $CDCl_3$) δ 4.84 (p, *J* = 6.3 Hz, 1H), 4.70 (t, *J* = 5.1 Hz, 1H), 4.11 (d, *J* = 6.9 Hz, 1H), 3.71 (d, *J* = 8.5 Hz, 1H), 3.31 (s, 3H), 3.29 (s, 3H), 3.23 (d, *J* = 2.3 Hz, 6H), 3.05 – 2.98 (m, 1H), 2.91 (s, 1H), 1.26 (dd, *J* = 6.3, 3.4 Hz, 6H), 1.05 (t, *J* = 7.1 Hz, 3H). ^{13}C NMR (100 MHz, $CDCl_3$) δ 154.1, 85.7, 82.8, 81.8, 80.9, 79.9, 77.4, 73.5, 71.8, 62.0, 59.3, 57.7, 56.3, 56.2, 54.1, 53.5, 50.4, 49.9, 49.2, 47.4, 44.9, 41.1, 39.4, 36.5, 35.2, 29.2, 26.4, 21.9, 13.7. HRMS (ESI): *m/z* [M + H] $^+$ calcd for $C_{29}H_{47}NO_8$: 538.3378, found: 538.3380.

Compound 15. (73% yield, white amorphous powder, mp, 162.7-164.1 °C), ^{1}H NMR (400 MHz, $CDCl_3$) δ 4.71 (t, *J* = 5.1 Hz, 1H), 4.10 (d, *J* = 6.9 Hz, 1H), 3.91 (dd, *J* = 10.4, 6.7 Hz, 1H), 3.83 (dd, *J* = 10.3, 6.8 Hz, 1H), 3.69 (d, *J* = 8.4 Hz, 1H), 3.30 (s, 3H), 3.28 (s, 3H), 3.22 (s, 3H), 3.20 (s, 3H), 3.03 – 2.96 (m, 1H), 2.90 (s, 1H), 1.04 (t, *J* = 7.2 Hz, 3H), 0.91 (d, *J* = 6.8 Hz, 6H). ^{13}C NMR (100 MHz, $CDCl_3$) δ 154.6, 85.6, 82.7, 81.8, 80.8, 80.1, 74.0, 73.5, 61.9, 59.3, 57.6, 56.2, 56.2, 54.0, 53.4, 50.3, 49.9, 49.2, 47.4, 44.8, 41.2, 39.3, 36.5, 35.1, 29.2, 27.8, 26.4, 18.9, 13.7. HRMS (ESI): *m/z* [M + H] $^+$ calcd for $C_{30}H_{49}NO_8$: 552.3539, found: 552.3536.

Compound 16. (88% yield, white amorphous powder, mp, 152.8-153.6 °C), ^{1}H NMR (400 MHz, $CDCl_3$) δ 7.99 (dd, *J* = 8.4, 1.4 Hz, 2H), 7.57 – 7.50 (m, 1H), 5.15 (t, *J* = 4.8 Hz, 1H), 4.14 (d, *J* = 6.8 Hz, 1H), 3.70 (d, *J* = 8.4 Hz, 1H), 3.31 (s, 3H), 3.30 (s, 3H), 3.26 (s, 3H), 3.18 (s, 3H), 3.05 (dd, *J* = 10.2, 6.4 Hz, 1H), 2.96 (s, 1H), 1.08 (t, *J* = 7.1 Hz, 3H). ^{13}C NMR (100 MHz, $CDCl_3$) δ 166.6, 132.9 (2C), 130.6, 129.7 (2C), 128.6, 85.6, 82.9, 81.9, 80.8, 77.2, 73.9, 62.0, 59.3, 57.7, 56.3, 56.1, 54.1, 53.8, 50.4,

49.9, 49.3, 47.1, 45.2, 41.4, 39.4, 36.9, 35.1, 29.3, 26.4, 13.7. HRMS (ESI): *m/z* [M + H] $^+$ calcd for $C_{32}H_{45}NO_7$: 556.3271, found: 556.3274.

Compound 17. (73% yield, white amorphous powder, mp, 155.2-156.5 °C), ^{1}H NMR (400 MHz, $CDCl_3$) δ 7.37 – 7.30 (m, 3H), 7.33 (d, *J* = 1.9 Hz, 2H), 7.27 (d, *J* = 1.9 Hz, 2H), 4.82 (t, *J* = 5.0 Hz, 1H), 4.03 (d, *J* = 6.8 Hz, 1H), 3.29 (s, 3H), 3.27 (s, 3H), 3.22 (s, 3H), 3.19 (s, 3H), 2.99 (dt, *J* = 12.2, 6.2 Hz, 1H), 2.85 (s, 1H), 1.05 (t, *J* = 7.8 Hz, 3H). ^{13}C NMR (100 MHz, $CDCl_3$) δ 171.0, 133.9, 129.7 (2C), 128.8 (2C), 127.4, 85.6, 82.7, 81.8, 80.8, 77.7, 73.4, 61.9, 59.3, 57.7, 56.3, 56.1, 54.1, 53.5, 50.4, 49.8, 49.2, 47.2, 44.9, 41.9, 41.2, 39.3, 36.3, 35.1, 29.3, 26.4, 13.7. HRMS (ESI): *m/z* [M + H] $^+$ calcd for $C_{33}H_{47}NO_7$: 570.3446, found: 570.3431.

Compound 18. (74% yield, white amorphous powder, mp, 160.1-162.2 °C), ^{1}H NMR (400 MHz, $CDCl_3$) δ 7.65 (d, *J* = 16.0 Hz, 1H), 7.49 (dd, *J* = 6.6, 3.0 Hz, 2H), 7.34 (d, *J* = 6.5 Hz, 3H), 6.40 (d, *J* = 16.0 Hz, 1H), 4.97 (s, 1H), 4.13 (d, *J* = 6.9 Hz, 1H), 3.69 (d, *J* = 8.5 Hz, 1H), 3.31 (s, 3H), 3.28 (s, 3H), 3.23 (s, 3H), 3.21 (s, 3H), 3.03 (dd, *J* = 10.1, 6.4 Hz, 1H), 2.93 (s, 1H), 1.06 (t, *J* = 7.1 Hz, 3H). ^{13}C NMR (100 MHz, $CDCl_3$) δ 166.6, 145.1, 134.4, 130.3 (2C), 128.9 (2C), 128.2, 118.1, 85.4, 82.8, 81.9, 80.7, 77.0, 73.8, 61.9, 59.2, 57.6, 56.1, 56.1, 54.1, 53.7, 50.4, 49.7, 49.2, 47.1, 45.0, 41.4, 39.3, 36.7, 34.9, 29.3, 26.3, 13.6. HRMS (ESI): *m/z* [M + H] $^+$ calcd for $C_{34}H_{47}NO_7$: 582.3441, found: 582.3431.

Compound 19. (65% yield, white amorphous powder, mp, 157.1-159.4 °C), ^{1}H NMR (400 MHz, $CDCl_3$) δ 7.33 – 7.25 (m, 3H), 7.23 – 7.17 (m, 3H), 4.83 (t, *J* = 5.0 Hz, 1H), 4.09 (d, *J* = 6.9 Hz, 1H), 3.72 (d, *J* = 8.4 Hz, 1H), 3.65 (dd, *J* = 3.6, 2.0 Hz, 1H), 3.31 (s, 3H), 3.30 (s, 3H), 3.24 (s, 3H), 3.19 (s, 3H), 3.05 – 2.99 (m, 1H), 2.98 – 2.90 (m, 3H), 1.06 (t, *J* = 7.1 Hz, 3H). ^{13}C NMR (100 MHz, $CDCl_3$) δ 172.5, 140.7, 128.7(2C), 128.4(2C), 126.4, 85.7, 82.8, 81.9, 80.8, 77.3, 73.7, 62.0, 59.3, 57.7, 56.3, 56.2, 54.0, 53.4, 50.4, 49.9, 49.3, 47.2, 44.9, 41.5, 39.4, 36.3, 36.2, 35.2, 30.9, 29.3, 26.4, 13.8. HRMS (ESI): *m/z* [M + H] $^+$ calcd for $C_{34}H_{49}NO_7$: 584.3586, found: 584.3587.

Compound 20. (48% yield, white amorphous powder, mp, 162.3-165.1 °C), ^{1}H NMR (400 MHz, $CDCl_3$) δ 7.88 (d, *J* = 8.1 Hz, 2H), 7.21 (s, 2H), 5.14 (t, *J* = 5.0 Hz, 1H), 4.14 (d, *J* = 6.9 Hz, 1H), 3.70 (d, *J* = 8.4 Hz, 1H), 3.31 (s, 3H), 3.30 (s, 3H), 3.26 (s, 3H), 3.18 (s, 3H), 3.06 (dd, *J* = 10.1, 6.4 Hz, 1H), 2.97 (s, 1H), 1.08 (t, *J* = 7.1 Hz, 3H). ^{13}C NMR (100 MHz, $CDCl_3$) δ 166.6, 143.6, 129.7 (2C), 129.3 (2C), 127.8, 85.6, 82.9, 81.9, 80.8, 77.0, 74.0, 62.0, 59.3, 57.7, 56.3, 56.1, 54.1, 53.7, 50.5, 49.9, 49.3, 47.1, 45.2, 41.5, 39.4, 37.0, 35.0, 29.3, 26.4, 21.8, 13.7. HRMS (ESI): *m/z* [M + H] $^+$ calcd for $C_{33}H_{47}NO_7$: 570.3414, found: 570.3431.

Compound 21. (83% yield, white amorphous powder, mp, 159.8-161.6 °C), ^{1}H NMR (400 MHz, $CDCl_3$) δ 7.94 (d, *J* = 8.8 Hz, 2H), 7.40 (d, *J* = 8.8 Hz, 2H), 5.13 (t, *J* = 5.1 Hz, 1H), 4.13 (d, *J* = 6.9 Hz, 1H), 3.71 (d, *J* = 8.4 Hz, 1H), 3.31 (s, 3H), 3.30 (s, 3H), 3.26 (s, 3H), 3.18 (s, 3H), 3.09 – 3.01 (m, 1H), 2.96 (s, 1H), 1.08 (t, *J* = 7.1 Hz, 3H). ^{13}C NMR (100 MHz, $CDCl_3$) δ 165.8, 139.4, 131.1 (2C), 129.1 (2C), 129.0, 85.6, 82.9,

81.9, 80.8, 77.4, 74.0, 62.0, 59.3, 57.8, 56.3, 56.2, 54.1, 54.1, 50.5, 49.9, 49.3, 46.9, 45.2, 41.4, 39.4, 37.0, 35.1, 29.2, 26.4, 13.7. HRMS (ESI): *m/z* [M + H] $^+$ calcd for $C_{32}H_{44}ClNO_7$: 590.2885, found: 590.2885.

Compound 22. (58% yield, white amorphous powder, mp, 155.3-156.5 °C), ^{1}H NMR (400 MHz, $CDCl_3$) δ 7.85 (dd, *J* = 8.6, 1.1 Hz, 2H), 7.55 (dd, *J* = 8.5, 1.5 Hz, 2H), 5.10 (t, *J* = 5.1 Hz, 1H), 4.11 (d, *J* = 7.1 Hz, 1H), 3.69 (d, *J* = 9.5 Hz, 1H), 3.30 (s, 3H), 3.29 (s, 3H), 3.24 (s, 3H), 3.16 (s, 3H), 3.04 (dd, *J* = 10.2, 6.3 Hz, 1H), 2.94 (s, 1H), 1.06 (t, *J* = 6.6 Hz, 3H). ^{13}C NMR (100 MHz, $CDCl_3$) δ 165.9, 131.9 (2C), 131.2 (2C), 129.5, 128.0, 85.6, 82.9, 81.8, 80.8, 77.3, 73.9, 62.0, 59.3, 57.7, 56.2, 56.1, 54.0 (2C), 50.4, 49.8, 49.2, 46.8, 45.2, 41.3, 39.3, 36.9, 35.1, 29.2, 26.4, 13.7. HRMS (ESI): *m/z* [M + H] $^+$ calcd for $C_{32}H_{44}BrNO_7$: 636.2353, found: 636.2359.

Compound 23. (72% yield, white amorphous powder, mp, 157.9-159.2 °C), ^{1}H NMR (400 MHz, $CDCl_3$) δ 8.05 – 7.99 (m, 2H), 7.14 – 7.05 (m, 2H), 5.13 (t, *J* = 4.7 Hz, 1H), 3.31 (s, 3H), 3.30 (s, 3H), 3.26 (s, 4H), 3.19 (s, 3H), 3.06 (dd, *J* = 10.0, 6.4 Hz, 1H), 2.96 (s, 1H), 2.43 – 2.37 (m, 2H), 2.08 (s, 1H), 1.09 (s, 3H). ^{13}C NMR (100 MHz, $CDCl_3$) δ 165.8 (d, *J* = 253.6 Hz), 165.7, 132.3 (2C) (d, *J* = 9.4 Hz), 126.9, 115.8 (2C) (d, *J* = 21.8 Hz), 85.5, 82.9, 81.9, 80.8, 77.4, 77.2, 74.0, 62.1, 59.3, 57.8, 56.3, 56.2, 54.1, 54.0, 50.5, 49.3, 46.9, 45.2, 41.4, 39.4, 37.0, 35.0, 29.2, 26.3, 13.7. HRMS (ESI): *m/z* [M + H] $^+$ calcd for $C_{32}H_{44}FNO_7$: 574.3179, found: 574.3180.

Compound 24. (75% yield, white amorphous powder, mp, 153.8-155.1 °C), ^{1}H NMR (400 MHz, $CDCl_3$) δ 8.16 (t, *J* = 1.8 Hz, 1H), 7.91 (dt, *J* = 7.8, 1.2 Hz, 1H), 7.66 (ddd, *J* = 8.0, 2.1, 1.1 Hz, 1H), 7.30 (t, *J* = 7.9 Hz, 1H), 5.12 (t, *J* = 5.0 Hz, 1H), 4.13 (d, *J* = 6.8 Hz, 1H), 3.70 (d, *J* = 8.5 Hz, 1H), 3.31 (s, 3H), 3.30 (s, 3H), 3.26 (s, 3H), 3.20 (s, 3H), 3.06 (dd, *J* = 10.2, 6.6 Hz, 1H), 2.96 (s, 1H), 1.08 (t, *J* = 7.2 Hz, 3H). ^{13}C NMR (100 MHz, $CDCl_3$) δ 165.4, 135.9, 132.9, 132.6, 130.2, 128.2, 122.7, 85.5, 82.9, 81.8, 80.7, 77.4, 73.9, 62.0, 59.3, 57.8, 56.3, 56.2, 54.1 (2C), 50.5, 49.7, 49.3, 46.8, 45.2, 41.3, 39.4, 37.1, 35.0, 29.2, 26.3, 13.7. HRMS (ESI): *m/z* [M + H] $^+$ calcd for $C_{32}H_{44}BrNO_7$: 636.2353, found: 636.2359.

Compound 25. (89% yield, white amorphous powder, mp, 156.9-158.2 °C), ^{1}H NMR (400 MHz, $CDCl_3$) δ 7.85 – 7.80 (m, 1H), 7.62 (dd, *J* = 7.17, 1.4 Hz, 1H), 7.35 (td, *J* = 7.5, 1.5 Hz, 1H), 7.30 (td, *J* = 7.6, 2.0 Hz, 1H), 5.08 (t, *J* = 5.0 Hz, 1H), 4.13 (d, *J* = 6.9 Hz, 1H), 3.71 (d, *J* = 8.4 Hz, 1H), 3.30 (s, 6H), 3.26 (s, 3H), 3.22 (s, 3H), 3.05 (dd, *J* = 10.3, 6.5 Hz, 1H), 2.96 (s, 1H), 1.07 (t, *J* = 7.1 Hz, 3H). ^{13}C NMR (100 MHz, $CDCl_3$) δ 165.9, 134.2, 132.9, 132.5, 131.3, 127.5, 121.3, 85.7, 82.7, 81.8, 80.8, 78.3, 73.6, 62.1, 59.3, 57.7, 56.3, 56.2, 54.0, 53.5, 50.4, 50.0, 49.3, 47.2, 44.9, 41.4, 39.4, 36.0, 35.2, 29.2, 26.4, 13.8. HRMS (ESI): *m/z* [M + H] $^+$ calcd for $C_{32}H_{44}BrNO_7$: 634.2397, found: 634.2379.

Compound 26. (85% yield, white amorphous powder, mp, 149.1-151.1 °C),^{1}H NMR (400 MHz, $CDCl_3$) δ 8.00 (td, *J* = 7.7, 1.9 Hz, 1H), 7.55 – 7.44 (m, 1H), 7.25 – 7.16 (m, 1H), 7.11 (ddt, *J* = 10.4, 8.4, 1.0 Hz, 1H), 5.06 (t, *J* = 5.0 Hz, 1H), 4.17 (d, *J* = 6.9 Hz,

1H), 3.82 – 3.62 (m, 1H), 3.33 (s, 3H), 3.29 (s, 3H), 3.25 (s, 3H), 3.12 (s, 3H), 3.05 (dt, *J* = 10.3, 4.6 Hz, 1H), 2.96 (s, 1H), 1.06 (t, *J* = 7.1 Hz, 3H). ^{13}C NMR (100 MHz, $CDCl_3$) δ 164.3 (d, *J* = 3.3 Hz), 161.7 (d, *J* = 256.1 Hz), 134.7 (d, *J* = 9.4 Hz), 132.8, 124.4 (d, *J* = 3.6 Hz), 118.7 (d, *J* = 10.2 Hz), 117.1 (d, *J* = 23.6 Hz), 85.7, 82.7, 81.8, 80.9, 78.2, 73.3, 62.0, 59.3, 57.7, 56.3, 56.0, 54.1, 53.4, 50.4, 50.0, 49.3, 47.5, 44.9, 41.0, 39.4, 36.3, 35.2, 29.3, 26.4, 13.7. HRMS (ESI): *m/z* [M + H] $^+$ calcd for $C_{32}H_{44}FNO_7$: 574.3179, found: 574.3180.

Compound 27. (84% yield, white amorphous powder, mp, 170.2-171.5 °C), ^{1}H NMR (400 MHz, $CDCl_3$) δ 7.30 (s, 2H), 7.19 (d, *J* = 8.4 Hz, 2H), 4.81 (t, *J* = 5.1 Hz, 1H), 4.05 (d, *J* = 7.0 Hz, 1H), 3.69 (d, *J* = 8.5 Hz, 1H), 3.60 (s, 2H), 3.28 (s, 6H), 3.22 (s, 3H), 3.16 (s, 3H), 3.00 (dd, *J* = 9.9, 6.3 Hz, 1H), 2.87 (s, 1H), 1.11 (t, *J* = 7.1 Hz, 2H), 1.06 (t, *J* = 7.1 Hz, 3H). ^{13}C NMR (100 MHz, $CDCl_3$) δ 170.6, 133.3, 132.4, 131.0, 130.3, 128.9, 128.8, 85.5, 82.7, 81.8, 80.7, 77.8, 73.5, 61.8, 59.3, 57.7, 56.2, 56.2, 54.2, 53.5, 50.4, 49.6, 49.2, 47.1, 44.9, 41.3, 41.1, 39.3, 36.3, 29.2, 26.3, 13.6. HRMS (ESI): *m/z* [M + H] $^+$ calcd for $C_{33}H_{46}ClNO_7$: 604.3052, found: 604.3041.

Compound 28. (90% yield, yellow oil), ^{1}H NMR (400 MHz, $CDCl_3$) δ 7.80 (s, 1H), 5.00 (t, *J* = 5.1 Hz, 1H), 4.03 (d, *J* = 6.9 Hz, 1H), 3.61 (d, *J* = 8.4 Hz, 1H), 3.21 (s, 3H), 3.20 (s, 3H), 3.16 (s, 3H), 3.15 (s, 3H), 2.98 – 2.93 (m, 1H), 2.87 (s, 1H), 0.98 (t, *J* = 7.2 Hz, 3H). ^{13}C NMR (100 MHz, $CDCl_3$) δ 164.2, 132.9, 132.3, 132.2, 132.0, 131.5, 131.3, 85.5, 82.7, 81.7, 80.7, 78.4, 73.5, 61.9, 59.3, 57.6, 56.2, 56.1, 54.1, 53.7, 50.4, 49.8, 49.2, 47.0, 44.9, 41.2, 39.3, 36.6, 35.0, 29.0, 26.3, 13.7. HRMS (ESI): *m/z* [M + H] $^+$ calcd for $C_{32}H_{43}Cl_2NO_7$: 624.2485, found: 624.2495.

Compound 29. (89% yield, white amorphous powder, mp, 150.4-151.8 °C), ^{1}H NMR (400 MHz, $CDCl_3$) δ 7.63 – 7.44 (m, 2H), 6.97 (tt, *J* = 8.6, 2.5 Hz, 1H), 5.09 (t, *J* = 5.0 Hz, 1H), 4.11 (d, *J* = 6.8 Hz, 1H), 3.69 (d, *J* = 8.5 Hz, 1H), 3.30 (s, 3H), 3.29 (s, 3H), 3.25 (s, 3H), 3.20 (s, 3H), 3.05 (dd, *J* = 10.1, 6.4 Hz, 1H), 2.95 (s, 1H), 1.07 (t, *J* = 7.1 Hz, 3H). ^{13}C NMR (100 MHz, $CDCl_3$) δ 164.5 (t, *J* = 3.4 Hz), 164.1 (d, *J* = 11.9 Hz), 161.6 (d, *J* = 12.0 Hz), 134.0 (t, *J* = 9.1 Hz), 113.3 – 112.4 (m) (2C), 108.3 (t, *J* = 25.4 Hz), 85.5, 82.9, 81.8, 80.7, 77.7, 73.9, 62.0, 59.3, 57.8, 56.2, 56.2, 54.4, 54.1, 50.5, 49.7, 49.2, 46.6, 45.2, 41.2, 39.4, 37.1, 35.0, 29.1, 26.4, 13.7. HRMS (ESI): *m/z* [M + H] $^+$ calcd for $C_{32}H_{43}F_2NO_7$: 592.3085, found: 592.3086.

Compound 30. (76% yield, white amorphous powder, mp, 162.3-163.5 °C), ^{1}H NMR (400 MHz, $CDCl_3$) δ 7.66 (d, *J* = 8.4 Hz, 1H), 7.09 (s, 1H), 4.89 (t, *J* = 5.1 Hz, 1H), 3.94 (d, *J* = 6.9 Hz, 1H), 3.52 (dd, *J* = 8.4, 1.4 Hz, 1H), 3.11 (s, 6H), 3.07 (s, 3H), 3.01 (s, 3H), 2.86 (dd, *J* = 10.3, 6.3 Hz, 1H), 2.77 (s, 1H), 0.88 (t, *J* = 7.1 Hz, 3H). ^{13}C NMR (100 MHz, $CDCl_3$) δ 164.5, 138.1, 134.3, 132.5, 130.8, 129.1, 127.3, 85.6, 82.7, 81.8, 80.8, 78.3, 73.5, 62.0, 59.3, 57.6, 56.2, 56.2, 54.0, 53.7, 50.4, 49.9, 49.2, 47.1, 44.9, 41.2, 39.4, 36.1, 35.1, 29.1, 26.4, 13.7. HRMS (ESI): *m/z* [M + H] $^+$ calcd for $C_{32}H_{43}Cl_2NO_7$: 624.2502, found: 624.2495.

Compound 31. (87% yield, white amorphous powder, mp, 161.9-163.4 °C), ^{1}H NMR (400 MHz, $CDCl_3$) δ 7.75 (dd, *J* = 7.8, 2.1 Hz, 1H), 7.57 (d, *J* = 9.5 Hz, 1H), 7.28 (d, *J* = 2.1 Hz, 1H), 5.09 (t, *J* = 5.1 Hz, 1H), 4.14 (d, *J* = 7.0 Hz, 1H), 3.72 (d, *J* = 8.4 Hz, 1H), 3.31 (s, 6H), 3.27 (s, 3H), 3.23 (s, 3H), 3.07 (dd, *J* = 10.3, 6.5 Hz, 1H), 2.98 (s, 1H), 1.09 (t, *J* = 7.1 Hz, 3H). ^{13}C NMR (100 MHz, $CDCl_3$) δ 164.9, 134.5, 133.5, 133.0, 131.2, 129.1, 127.5, 85.6, 82.7, 81.8, 80.8, 78.6, 73.6, 62.0, 59.3, 57.6, 56.2, 56.2, 54.1, 53.6, 50.4, 50.0, 49.3, 47.2, 44.9, 41.3, 39.4, 36.1, 35.1, 29.2, 26.4, 13.7. HRMS (ESI): *m/z* [M + H] $^+$ calcd for $C_{32}H_{43}Cl_2NO_7$: 624.2485, found: 624.2495.

Compound 32. (90% yield, white amorphous powder, mp, 153.8-154.7 °C), ^{1}H NMR (400 MHz, $CDCl_3$) δ 7.75 (dd, *J* = 7.8, 1.6 Hz, 1H), 7.57 (dd, *J* = 8.1, 1.6 Hz, 1H), 7.26 (d, *J* = 15.9 Hz, 1H), 5.09 (t, *J* = 4.7 Hz, 1H), 4.14 (d, *J* = 6.8 Hz, 1H), 3.72 (d, *J* = 8.5 Hz, 1H), 3.31 (s, 6H), 3.27 (s, 3H), 3.23 (s, 3H), 3.07 (dd, *J* = 10.3, 6.4 Hz, 1H), 2.98 (s, 1H), 1.09 (t, *J* = 7.2 Hz, 3H). ^{13}C NMR (100 MHz, $CDCl_3$) δ 164.9, 134.5, 133.5, 133.0, 131.2, 129.1, 127.5, 85.6, 82.7, 81.8, 80.8, 78.6, 73.5, 62.0, 59.3, 57.6, 56.2, 56.2, 54.0, 53.5, 50.4, 49.9, 49.3, 47.2, 44.8, 41.3, 39.4, 36.0, 35.1, 29.1, 26.4, 13.7. HRMS (ESI): *m/z* [M + H] $^+$ calcd for $C_{32}H_{43}F_2NO_7$: 592.3085, found: 592.3086.

Compound 33. (85% yield, white amorphous powder, mp, 142.9-144.3 °C), ^{1}H NMR (400 MHz, $CDCl_3$) δ 7.76 (dd, *J* = 3.8, 1.4 Hz, 1H), 7.51 (dd, *J* = 5.0, 1.3 Hz, 1H), 7.10 – 7.03 (m, 1H), 5.07 (t, *J* = 4.7 Hz, 1H), 4.13 (d, *J* = 6.9 Hz, 1H), 3.69 (d, *J* = 8.5 Hz, 1H), 3.30 (s, 3H), 3.28 (s, 3H), 3.23 (s, 3H), 3.15 (s, 3H), 3.08 – 2.99 (m, 1H), 2.93 (s, 1H), 1.06 (t, *J* = 7.1 Hz, 3H). ^{13}C NMR (100 MHz, $CDCl_3$) δ 161.6, 133.7, 133.5, 132.2, 127.9, 85.5, 82.8, 81.7, 80.7, 77.4, 73.8, 61.9, 59.3, 57.7, 56.2, 56.0, 54.1, 53.6, 50.4, 49.8, 49.2, 47.1, 45.0, 41.4, 39.3, 36.9, 34.9, 29.2, 26.3, 13.6. HRMS (ESI): *m/z* [M + H] $^+$ calcd for $C_{30}H_{43}NO_7S$: 562.2834, found: 562.2838.

Compound 34. (86% yield, yellow oil), ^{1}H NMR (400 MHz, $CDCl_3$) δ 7.57 (d, *J* = 4.1 Hz, 1H), 6.92 (d, *J* = 4.0 Hz, 1H), 5.07 (t, *J* = 5.0 Hz, 1H), 4.13 (d, *J* = 6.9 Hz, 1H), 3.71 (d, *J* = 8.5 Hz, 1H), 3.32 (s, 3H), 3.30 (s, 3H), 3.25 (s, 3H), 3.19 (s, 3H), 3.04 (dd, *J* = 10.3, 6.4 Hz, 1H), 2.94 (s, 1H), 1.08 (t, *J* = 7.1 Hz, 3H). ^{13}C NMR (100 MHz, $CDCl_3$) δ 160.8, 137.4, 133.1, 132.0, 127.5, 85.6, 82.9, 81.7, 80.8, 77.8, 73.9, 62.0, 59.3, 57.8, 56.3, 56.2, 54.1, 53.9, 50.5, 49.9, 49.3, 47.1, 45.1, 41.4, 39.4, 37.0, 35.2, 29.2, 26.4, 13.7. HRMS (ESI): *m/z* [M + H] $^+$ calcd for $C_{30}H_{42}ClO_7S$: 596.2442, found: 596.2449.

Compound 35. (81% yield, yellow oil), ^{1}H NMR (400 MHz, $CDCl_3$) δ 4.82 (t, *J* = 5.1 Hz, 1H), 4.10 (d, *J* = 6.9 Hz, 1H), 3.97 (t, *J* = 3.7 Hz, 1H), 3.94 (t, *J* = 3.6 Hz, 1H), 3.71 (d, *J* = 8.5 Hz, 1H), 3.38 (ddd, *J* = 11.3, 8.9, 2.4 Hz, 2H), 3.31 (s, 3H), 3.29 (s, 3H), 3.23 (s, 3H), 3.19 (s, 3H), 3.01 (dd, *J* = 10.3, 6.4 Hz, 1H), 2.90 (s, 1H), 1.05 (t, *J* = 7.1 Hz, 3H). ^{13}C NMR (100 MHz, $CDCl_3$) δ 173.9, 85.6, 82.8, 81.9, 80.8, 77.4, 73.8, 67.3, 67.2, 62.0, 59.3, 57.7, 56.2, 56.1, 54.1, 53.4, 50.4, 49.9, 49.2, 47.1, 44.9, 41.5, 40.4, 39.4, 36.2, 35.1, 29.3, 28.8, 28.8, 26.4, 13.7. HRMS (ESI): *m/z* [M + H] $^+$ calcd for $C_{31}H_{49}NO_8$: 564.3553, found: 564.3536.

Compound 36. (73% yield, yellow oil), ^{1}H NMR (400 MHz, $CDCl_3$) δ 9.21 (dd, *J* = 2.2, 0.9 Hz, 1H), 8.75 (dd, *J* = 4.8, 1.8 Hz, 1H), 8.26 (dt, *J* = 7.9, 1.9 Hz, 1H), 7.41 – 7.33 (m, 1H), 5.15 (t, *J* = 4.8 Hz, 1H), 4.13 (d, *J* = 6.8 Hz, 1H), 3.70 (d, *J* = 8.5 Hz, 1H), 3.31 (s, 3H), 3.30 (s, 3H), 3.26 (s, 3H), 3.18 (s, 3H), 3.05 (dd, *J* = 10.1, 6.5 Hz, 1H), 2.95 (s, 1H), 1.08 (t, *J* = 7.1 Hz, 3H). ^{13}C NMR (100 MHz, $CDCl_3$) δ 165.4, 153.4, 151.1, 137.2, 126.6, 123.5, 85.6, 82.9, 81.9, 80.8, 77.4, 73.9, 62.0, 59.3, 57.8, 56.3, 56.3, 54.3, 54.1, 50.5, 49.8, 49.3, 46.7, 45.2, 41.3, 39.4, 37.1, 35.1, 29.2, 26.4, 13.7. HRMS (ESI): *m/z* [M + H] $^+$ calcd for $C_{31}H_{44}N_2O_7$: 557.3232, found: 557.3227.

Compound 37. (90% yield, yellow oil.) ^{1}H NMR (400 MHz, $CDCl_3$) δ 7.79 (dd, *J* = 3.7, 1.3 Hz, 1H), 7.53 (dd, *J* = 4.9, 1.3 Hz, 1H), 7.08 (dd, *J* = 5.0, 3.7 Hz, 1H), 5.02 (t, *J* = 4.9 Hz, 1H), 4.04 (d, *J* = 6.6 Hz, 1H), 3.63 (d, *J* = 8.4 Hz, 1H), 3.34 (s, 3H), 3.27 (s, 3H), 3.24 (s, 3H), 3.18 (s, 3H), 3.13 (d, *J* = 8.4 Hz, 1H), 3.07 – 2.99 (m, 2H), 2.72 (t, *J* = 5.8 Hz, 1H), 2.08 (d, *J* = 6.1 Hz, 1H), 1.06 (t, *J* = 7.2 Hz, 3H). ^{13}C NMR (100 MHz, $CDCl_3$) δ 169.9, 162.1, 134.4, 133.6, 132.6, 127.9, 86.0, 85.1, 83.6, 82.7, 80.5, 76.0, 61.7, 59.2, 58.0, 56.6, 56.2, 53.9, 50.5, 49.3, 49.2, 49.1, 44.9, 44.2, 39.5, 39.2, 37.8, 35.0, 29.1, 26.5, 21.8, 13.6. HRMS (ESI): *m/z* [M + H] $^+$ calcd for $C_{32}H_{45}NO_8S$: 604.2930, found: 604.2944.

Compound 38. (62% yield, white amorphous powder, mp, 156.8-158.1 °C) ^{1}H NMR (400 MHz, $CDCl_3$) δ 7.68 (d, *J* = 16.0 Hz, 1H), 7.53 – 7.47 (m, 2H), 7.41 – 7.34 (m, 3H), 6.43 (d, *J* = 16.0 Hz, 1H), 4.93 (t, *J* = 4.8 Hz, 1H), 4.06 (d, *J* = 7.8 Hz, 1H), 3.63 (d, *J* = 8.5 Hz, 1H), 3.36 (s, 3H), 3.28 (s, 3H), 3.25 (s, 3H), 3.20 (s, 3H), 3.14 (d, *J* = 8.4 Hz, 1H), 3.06 – 2.99 (m, 2H), 2.72 (dd, *J* = 6.9, 4.9 Hz, 1H), 1.07 (t, *J* = 7.2 Hz, 3H). ^{13}C NMR (100 MHz, $CDCl_3$) δ 169.8, 166.9, 145.1, 134.4, 130.5, 129.1(2C), 128.2(2C), 118.5, 86.0, 85.1, 83.6, 82.9, 80.5, 75.6, 61.7, 59.2, 58.0, 56.7, 56.2, 53.9, 50.4, 49.2, 49.1(2C), 45.0, 44.1, 39.5, 39.1, 38.0, 34.9, 29.2, 26.5, 22.5, 13.6. HRMS (ESI): *m/z* [M + H] $^+$ calcd for $C_{36}H_{49}NO_8$: 624.3524, found: 624.3536.

Chasmanine. (90% yield, white amorphous powder, mp, 145.8-147.3 °C) ^{1}H NMR (400 MHz, $CDCl_3$) δ 4.19 (d, *J* = 7.0 Hz, 1H), 4.11 (q, *J* = 4.9 Hz, 1H), 3.70 (d, *J* = 8.5 Hz, 1H), 3.39 (d, *J* = 9.3 Hz, 1H), 3.33 (s, 3H), 3.29 (d, *J* = 2.1 Hz, 6H), 3.23 (s, 3H), 3.12 (s, 1H), 2.99 (dd, *J* = 10.8, 6.4 Hz, 1H), 1.06 (t, *J* = 7.1 Hz, 3H). ^{13}C NMR (100 MHz, $CDCl_3$) δ 86.4, 82.5, 82.2, 80.9, 75.7, 72.7, 62.8, 59.4, 57.5, 56.6, 56.3, 54.0, 52.8, 50.5, 50.4, 49.5, 48.8, 45.7, 39.6, 39.0, 38.0, 35.4, 28.5, 26.1, 13.9. HRMS (ESI): *m/z* [M + H] $^+$ calcd for $C_{25}H_{42}NO_6$: 452.3026, found: 452.3012.

intermediate 1. (95% yield, White granular crystal, mp, 142.3-143.3 °C) ^{1}H NMR (400 MHz, $CDCl_3$) δ 8.10 – 8.01 (m, 2H), 6.97 – 6.87 (m, 2H), 5.22 (d, *J* = 5.4 Hz, 1H), 4.38 (dd, *J* = 9.0, 5.5 Hz, 1H), 3.97 (d, *J* = 6.6 Hz, 1H), 3.86 (s, 3H), 3.75 (dd, *J* = 15.6, 5.9 Hz, 1H), 3.62 (d, *J* = 8.5 Hz, 1H), 3.37 (s, 3H), 3.28 (s, 3H), 3.25 (s, 3H), 3.15 (s, 3H), 2.95 (s, 1H), 2.92 – 2.86 (m, 1H), 1.33 (s, 3H), 1.09 (t, *J* = 7.1 Hz, 3H). ^{13}C NMR (100 MHz, $CDCl_3$) δ 214.1, 170.0, 166.2, 163.7, 132.1(2C), 122.6, 113.9(2C), 90.0, 85.4, 85.1, 83.4, 80.5, 79.3, 77.4, 62.4, 59.3, 58.2, 57.9, 56.5, 55.6, 53.5, 50.4, 49.5, 49.4,

49.1, 43.8, 42.0, 39.9, 39.3, 35.1, 34.8, 26.5, 21.8, 19.5, 13.6. HRMS (ESI): *m/z* [M + H] $^+$ calcd for $C_{37}H_{51}NO_{10}S_2$: 734.3030, found: 734.3033.

intermediate 2. (86% yield, white amorphous powder, mp, 159.8-161.0 °C) ^{1}H NMR (400 MHz, $CDCl_3$) δ 8.10 – 8.01 (m, 2H), 6.97 – 6.87 (m, 2H), 5.22 (d, *J* = 5.4 Hz, 1H), 4.38 (dd, *J* = 9.0, 5.5 Hz, 1H), 3.97 (d, *J* = 6.6 Hz, 1H), 3.86 (s, 3H), 3.75 (dd, *J* = 15.6, 5.9 Hz, 1H), 3.62 (d, *J* = 8.5 Hz, 1H), 3.37 (s, 3H), 3.28 (s, 3H), 3.25 (s, 3H), 3.15 (s, 3H), 2.95 (s, 1H), 2.92 – 2.86 (m, 1H), 1.33 (s, 3H), 1.09 (t, *J* = 7.1 Hz, 3H). ^{13}C NMR (100 MHz, $CDCl_3$) δ 169.9, 166.3, 163.5, 131.8, 123.1, 113.8, 86.0, 85.2, 83.6, 83.0, 80.6, 75.5, 61.8, 59.2, 58.0, 56.7, 56.2, 55.6, 53.9, 50.5, 49.5, 49.3, 49.2, 45.1, 44.1, 39.3, 38.0, 35.0, 29.1, 26.5, 21.9, 13.6. HRMS (ESI): *m/z* [M + H] $^+$ calcd for $C_{35}H_{49}NO_9$: 628.3483, found: 628.3486.

Data of HR-ESI-MS：

Compound 1

Elemental Composition Report

Single Mass Analysis

Tolerance = 2.0 mDa / DBE: min = -20.0, max = 20.0

Element prediction: Off

Monoisotopic Mass, Even Electron Ions

208 formula(e) evaluated with 2 results within limits (all results (up to 1000) for each mass)

Elements Used:

C: 15-50 H: 20-60 N: 1-5 O: 1-12

Minimum:				-20.0	
Maximum:		2.0	10.0	20.0	
Mass	Calc. Mass	mDa	PPM	DBE	Formula
508.3281	508.3274	0.7	1.4	6.5	C28 H46 N O7
	508.3288	-0.7	-1.4	11.5	C29 H42 N5 O3

Compound 2

Elemental Composition Report

Single Mass Analysis

Tolerance = 2.0 mDa / DBE: min = -20.0, max = 20.0

Element prediction: Off

Monoisotopic Mass, Even Electron Ions

160 formula(e) evaluated with 2 results within limits (all results (up to 1000) for each mass)

Elements Used:

C: 15-50 H: 20-50 N: 1-5 O: 1-12

Minimum:				-20.0	
Maximum:		2.0	10.0	20.0	
Mass	Calc. Mass	mDa	PPM	DBE	Formula
522.3430	522.3431	-0.1	-0.2	6.5	C29 H48 N O7
	522.3444	-1.4	-2.7	11.5	C30 H44 N5 O3

Compound 3

Elemental Composition Report

Single Mass Analysis

Tolerance = 2.0 mDa / DBE: min = -20.0, max = 20.0

Element prediction: Off

Monoisotopic Mass, Even Electron Ions

208 formula(e) evaluated with 1 results within limits (all results (up to 1000) for each mass)

Elements Used:

C: 15-50 H: 20-60 N: 1-5 O: 1-12

Minimum:				-20.0	
Maximum:		2.0	10.0	20.0	
Mass	Calc. Mass	mDa	PPM	DBE	Formula
520.3259	520.3274	-1.5	-2.9	7.5	C29 H46 N O7

Compound 4

Elemental Composition Report

Single Mass Analysis

Tolerance = 2.0 mDa / DBE: min = -20.0, max = 20.0

Element prediction: Off

Monoisotopic Mass, Even Electron Ions

208 formula(e) evaluated with 2 results within limits (all results (up to 1000) for each mass)

Elements Used:

C: 15-50 H: 20-60 N: 1-5 O: 1-12

Minimum:				-20.0	
Maximum:		2.0	10.0	20.0	
Mass	Calc. Mass	mDa	PPM	DBE	Formula
536.3591	536.3587	0.4	0.7	6.5	C30 H50 N O7
	536.3601	-1.0	-1.9	11.5	C31 H46 N5 O3

Compound 5

Elemental Composition Report

Single Mass Analysis

Tolerance = 2.0 mDa / DBE: min = -20.0, max = 20.0

Element prediction: Off

Monoisotopic Mass, Even Electron Ions

208 formula(e) evaluated with 1 results within limits (all results (up to 1000) for each mass)

Elements Used:

C: 15-50 H: 20-60 N: 1-5 O: 1-12

Minimum:				-20.0	
Maximum:		2.0	10.0	20.0	
Mass	Calc. Mass	mDa	PPM	DBE	Formula
564.3893	564.3900	-0.7	-1.2	6.5	C32 H54 N O7

Compound 6

Elemental Composition Report

Single Mass Analysis

Tolerance = 2.0 mDa / DBE: min = -20.0, max = 20.0

Element prediction: Off

Monoisotopic Mass, Even Electron Ions

212 formula(e) evaluated with 2 results within limits (all results (up to 1000) for each mass)

Elements Used:

C: 15-50 H: 20-60 N: 1-5 O: 1-12

Minimum: -20.0

Maximum: 2.0 10.0 20.0

Mass Calc. Mass mDa PPM DBE Formula

522.3430 522.3431 -0.1 -0.2 6.5 C29 H48 N O7

522.3444 -1.4 -2.7 11.5 C30 H44 N5 O3

Compound 7

Elemental Composition Report

Single Mass Analysis

Tolerance = 2.0 mDa / DBE: min = -20.0, max = 20.0

Element prediction: Off

Monoisotopic Mass, Even Electron Ions

212 formula(e) evaluated with 2 results within limits (all results (up to 1000) for each mass)

Elements Used:

C: 15-50 H: 20-60 N: 1-5 O: 1-12

Minimum:				-20.0	
Maximum:		2.0	10.0	20.0	
Mass	Calc. Mass	mDa	PPM	DBE	Formula
550.3740	550.3744	-0.4	-0.7	6.5	C31 H52 N O7
	550.3757	-1.7	-3.1	11.5	C32 H48 N5 O3

Compound 8

Elemental Composition Report

Single Mass Analysis

Tolerance = 2.0 mDa / DBE: min = -20.0, max = 20.0

Element prediction: Off

Monoisotopic Mass, Even Electron Ions

212 formula(e) evaluated with 2 results within limits (all results (up to 1000) for each mass)

Elements Used:

C: 15-50 H: 20-60 N: 1-5 O: 1-12

Minimum: -20.0

Maximum: 2.0 10.0 20.0

Mass Calc. Mass mDa PPM DBE Formula

562.3749 562.3744 0.5 0.9 7.5 C32 H52 N O7

562.3757 -0.8 -1.4 12.5 C33 H48 N5 O3

Compound 9

Elemental Composition Report

Single Mass Analysis

Tolerance = 2.0 mDa / DBE: min = -20.0, max = 20.0

Element prediction: Off

Monoisotopic Mass, Even Electron Ions

430 formula(e) evaluated with 4 results within limits (all results (up to 1000) for each mass)

Elements Used:

C: 5-30 H: 15-60 N: 1-9 O: 1-15

Minimum:				-20.0	
Maximum:		2.0	10.0	20.0	
Mass	Calc. Mass	mDa	PPM	DBE	Formula
520.3288	520.3288	0.0	0.0	12.5	C30 H42 N5 O3
	520.3293	-0.5	-1.0	-5.5	C17 H50 N3 O14
	520.3274	1.4	2.7	7.5	C29 H46 N O7
	520.3306	-1.8	-3.5	-0.5	C18 H46 N7 O10

Compound 10

Elemental Composition Report

Single Mass Analysis

Tolerance = 2.0 mDa / DBE: min = -20.0, max = 20.0

Element prediction: Off

Monoisotopic Mass, Even Electron Ions

352 formula(e) evaluated with 2 results within limits (all results (up to 1000) for each mass)

Elements Used:

C: 15-50 H: 20-90 N: 1-5 O: 1-12

Minimum:				-20.0	
Maximum:		2.0	10.0	20.0	
Mass	Calc. Mass	mDa	PPM	DBE	Formula
690.5314	690.5309	0.5	0.7	6.5	C41 H72 N O7
	690.5322	-0.8	-1.2	11.5	C42 H68 N5 O3

Compound 11

Elemental Composition Report

Single Mass Analysis

Tolerance = 2.0 mDa / DBE: min = -20.0, max = 20.0

Element prediction: Off

Monoisotopic Mass, Even Electron Ions

212 formula(e) evaluated with 2 results within limits (all results (up to 1000) for each mass)

Elements Used:

C: 15-50 H: 20-60 N: 1-5 O: 1-12

Minimum: -20.0

Maximum: 2.0 10.0 20.0

Mass Calc. Mass mDa PPM DBE Formula

538.3384 538.3380 0.4 0.7 6.5 C29 H48 N O8

538.3393 -0.9 -1.7 11.5 C30 H44 N5 O4

Compound 12

Elemental Composition Report

Single Mass Analysis

Tolerance = 2.0 mDa / DBE: min = -20.0, max = 20.0

Element prediction: Off

Monoisotopic Mass, Even Electron Ions

208 formula(e) evaluated with 2 results within limits (all results (up to 1000) for each mass)

Elements Used:

C: 15-50 H: 20-60 N: 1-5 O: 1-12

Mass	Calc. Mass	mDa	PPM	DBE	Formula
Minimum:				-20.0	
Maximum:		2.0	10.0	20.0	
552.3549	552.3550	-0.1	-0.2	11.5	C31 H46 N5 O4
	552.3536	1.3	2.4	6.5	C30 H50 N O8

Compound 13

Elemental Composition Report

Single Mass Analysis

Tolerance = 2.0 mDa / DBE: min = -20.0, max = 20.0

Element prediction: Off

Monoisotopic Mass, Even Electron Ions

212 formula(e) evaluated with 1 results within limits (all results (up to 1000) for each mass)

Elements Used:

C: 15-50 H: 20-60 N: 1-5 O: 1-12

Minimum:				-20.0	
Maximum:		2.0	10.0	20.0	
Mass	Calc. Mass	mDa	PPM	DBE	Formula
566.3677	566.3693	-1.6	-2.8	6.5	C31 H52 N O8

Compound 14

Elemental Composition Report

Single Mass Analysis

Tolerance = 2.0 mDa / DBE: min = -20.0, max = 20.0

Element prediction: Off

Monoisotopic Mass, Even Electron Ions

212 formula(e) evaluated with 2 results within limits (all results (up to 1000) for each mass)

Elements Used:

C: 15-50 H: 20-60 N: 1-5 O: 1-12

Minimum: -20.0

Maximum: 2.0 10.0 20.0

Mass Calc. Mass mDa PPM DBE Formula

538.3378 538.3380 -0.2 -0.4 6.5 C29 H48 N O8

538.3393 -1.5 -2.8 11.5 C30 H44 N5 O4

Compound 15

Elemental Composition Report

Single Mass Analysis

Tolerance = 2.0 mDa / DBE: min = -20.0, max = 20.0

Element prediction: Off

Monoisotopic Mass, Even Electron Ions

208 formula(e) evaluated with 2 results within limits (all results (up to 1000) for each mass)

Elements Used:

C: 15-50 H: 20-60 N: 1-5 O: 1-12

Minimum:				-20.0	
Maximum:		2.0	10.0	20.0	
Mass	Calc. Mass	mDa	PPM	DBE	Formula
552.3539	552.3536	0.3	0.5	6.5	C30 H50 N O8
	552.3550	-1.1	-2.0	11.5	C31 H46 N5 O4

Compound 16

Elemental Composition Report

Single Mass Analysis

Tolerance = 2.0 mDa / DBE: min = -20.0, max = 20.0

Element prediction: Off

Monoisotopic Mass, Even Electron Ions

208 formula(e) evaluated with 2 results within limits (all results (up to 1000) for each mass)

Elements Used:

C: 15-50 H: 20-60 N: 1-5 O: 1-12

Minimum:				-20.0	
Maximum:		2.0	10.0	20.0	
Mass	Calc. Mass	mDa	PPM	DBE	Formula
556.3271	556.3274	-0.3	-0.5	10.5	C32 H46 N O7
	556.3288	-1.7	-3.1	15.5	C33 H42 N5 O3

Compound 17

Elemental Composition Report

Single Mass Analysis

Tolerance = 2.0 mDa / DBE: min = -20.0, max = 20.0

Element prediction: Off

Monoisotopic Mass, Even Electron Ions

212 formula(e) evaluated with 2 results within limits (all results (up to 1000) for each mass)

Elements Used:

C: 15-50 H: 20-60 N: 1-5 O: 1-12

Minimum:				-20.0	
Maximum:		2.0	10.0	20.0	
Mass	Calc. Mass	mDa	PPM	DBE	Formula
570.3446	570.3444	0.2	0.4	15.5	C34 H44 N5 O3
	570.3431	1.5	2.6	10.5	C33 H48 N O7

Compound 18

Elemental Composition Report

Single Mass Analysis

Tolerance = 2.0 mDa / DBE: min = -20.0, max = 20.0

Element prediction: Off

Monoisotopic Mass, Even Electron Ions

212 formula(e) evaluated with 2 results within limits (all results (up to 1000) for each mass)

Elements Used:

C: 15-50 H: 20-60 N: 1-5 O: 1-12

Mass	Calc. Mass	mDa	PPM	DBE	Formula
Minimum:				-20.0	
Maximum:		2.0	10.0	20.0	
582.3441	582.3444	-0.3	-0.5	16.5	C35 H44 N5 O3
	582.3431	1.0	1.7	11.5	C34 H48 N O7

Compound 19

Elemental Composition Report

Single Mass Analysis

Tolerance = 2.0 mDa / DBE: min = -20.0, max = 20.0

Element prediction: Off

Monoisotopic Mass, Even Electron Ions

208 formula(e) evaluated with 2 results within limits (all results (up to 1000) for each mass)

Elements Used:

C: 15-50 H: 20-60 N: 1-5 O: 1-12

Minimum:				-20.0	
Maximum:		2.0	10.0	20.0	
Mass	Calc. Mass	mDa	PPM	DBE	Formula
584.3586	584.3587	-0.1	-0.2	10.5	C34 H50 N O7
	584.3601	-1.5	-2.6	15.5	C35 H46 N5 O3

Compound 20

Elemental Composition Report

Single Mass Analysis

Tolerance = 2.0 mDa / DBE: min = -20.0, max = 20.0

Element prediction: Off

Monoisotopic Mass, Even Electron Ions

212 formula(e) evaluated with 1 results within limits (all results (up to 1000) for each mass)

Elements Used:

C: 15-50 H: 20-60 N: 1-5 O: 1-12

Minimum:				-20.0	
Maximum:		2.0	10.0	20.0	
Mass	Calc. Mass	mDa	PPM	DBE	Formula
570.3414	570.3431	-1.7	-3.0	10.5	C33 H48 N O7

Compound 21

Elemental Composition Report

Single Mass Analysis

Tolerance = 2.0 mDa / DBE: min = -20.0, max = 20.0

Element prediction: Off

Monoisotopic Mass, Even Electron Ions

408 formula(e) evaluated with 3 results within limits (all results (up to 1000) for each mass)

Elements Used:

C: 15-50 H: 20-60 N: 1-5 O: 1-12 Cl: 1-2

Mass	Calc. Mass	mDa	PPM	DBE	Formula
Minimum:				-20.0	
Maximum:		2.0	10.0	20.0	
590.2885	590.2885	0.0	0.0	10.5	C32 H45 N O7 Cl
	590.2876	0.9	1.5	6.5	C27 H46 N5 O5 Cl2
	590.2898	-1.3	-2.2	15.5	C33 H41 N5 O3 Cl

Compound 22 Elemental Composition Report

Single Mass Analysis

Tolerance = 2.0 mDa / DBE: min = -20.0, max = 20.0

Element prediction: Off

Monoisotopic Mass, Even Electron Ions

931 formula(e) evaluated with 3 results within limits (all results (up to 1000) for each mass)

Elements Used:

C: 15-50 H: 20-60 N: 1-5 O: 1-12 81Br: 0-8

Minimum:				-20.0	
Maximum:		2.0	10.0	20.0	
Mass	Calc. Mass	mDa	PPM	DBE	Formula
636.2353	636.2359	-0.6	-0.9	10.5	C32 H45 N O7 81Br
	636.2345	0.8	1.3	0.5	C24 H52 N5 O4 81Br2
	636.2372	-1.9	-3.0	15.5	C33 H41 N5 O3 81Br

Compound 23 Elemental Composition Report

Single Mass Analysis

Tolerance = 2.0 mDa / DBE: min = -20.0, max = 20.0

Element prediction: Off

Monoisotopic Mass, Even Electron Ions

608 formula(e) evaluated with 4 results within limits (all results (up to 1000) for each mass)

Elements Used:

C: 15-50 H: 20-60 N: 1-5 O: 1-12 F: 1-3

Minimum:				-20.0	
Maximum:		2.0	10.0	20.0	
Mass	Calc. Mass	mDa	PPM	DBE	Formula
574.3179	574.3180	-0.1	-0.2	10.5	C32 H45 N O7 F
	574.3191	-1.2	-2.1	6.5	C29 H46 N O8 F2
	574.3193	-1.4	-2.4	15.5	C33 H41 N5 O3 F
	574.3163	1.6	2.8	-1.5	C21 H47 N3 O11 F3

Compound 24

Elemental Composition Report

Single Mass Analysis

Tolerance = 2.0 mDa / DBE: min = -20.0, max = 20.0

Element prediction: Off

Monoisotopic Mass, Even Electron Ions

931 formula(e) evaluated with 3 results within limits (all results (up to 1000) for each mass)

Elements Used:

C: 15-50 H: 20-60 N: 1-5 O: 1-12 81Br: 0-8

Minimum:				-20.0	
Maximum:		2.0	10.0	20.0	
Mass	Calc. Mass	mDa	PPM	DBE	Formula
636.2353	636.2359	-0.6	-0.9	10.5	C32 H45 N O7 81Br
	636.2345	0.8	1.3	0.5	C24 H52 N5 O4 81Br2
	636.2372	-1.9	-3.0	15.5	C33 H41 N5 O3 81Br

Compound 25

Elemental Composition Report

Single Mass Analysis

Tolerance = 2.0 mDa / DBE: min = -20.0, max = 20.0

Element prediction: Off

Monoisotopic Mass, Even Electron Ions

617 formula(e) evaluated with 4 results within limits (all results (up to 1000) for each mass)

Elements Used:

C: 15-50 H: 20-60 N: 1-5 O: 1-12 Br: 0-2

Mass	Calc. Mass	mDa	PPM	DBE	Formula
Minimum:				-20.0	
Maximum:		2.0	10.0	20.0	
634.2397	634.2393	0.4	0.6	15.5	C33 H41 N5 O3 Br
	634.2401	-0.4	-0.6	17.5	C33 H36 N3 O10
	634.2390	0.7	1.1	-4.5	C20 H54 N5 O7 Br2
	634.2379	1.8	2.8	10.5	C32 H45 N O7 Br

Compound 26

Elemental Composition Report

Single Mass Analysis

Tolerance = 2.0 mDa / DBE: min = -20.0, max = 20.0

Element prediction: Off

Monoisotopic Mass, Even Electron Ions

608 formula(e) evaluated with 4 results within limits (all results (up to 1000) for each mass)

Elements Used:

C: 15-50 H: 20-60 N: 1-5 O: 1-12 F: 1-3

Minimum:				-20.0	
Maximum:		2.0	10.0	20.0	
Mass	Calc. Mass	mDa	PPM	DBE	Formula
574.3179	574.3180	-0.1	-0.2	10.5	C32 H45 N O7 F
574.3191	-1.2	-2.1	6.5	C29 H46 N O8 F2	
574.3193	-1.4	-2.4	15.5	C33 H41 N5 O3 F	
574.3163	1.6	2.8	-1.5	C21 H47 N3 O11 F3	

Compound 27

Elemental Composition Report

Single Mass Analysis

Tolerance = 2.0 mDa / DBE: min = -20.0, max = 20.0

Element prediction: Off

Monoisotopic Mass, Even Electron Ions

412 formula(e) evaluated with 3 results within limits (all results (up to 1000) for each mass)

Elements Used:

C: 15-50 H: 20-60 N: 1-5 O: 1-12 Cl: 1-2

Minimum: -20.0

Maximum: 2.0 10.0 20.0

Mass Calc. Mass mDa PPM DBE Formula

604.3052 604.3054 -0.2 -0.3 15.5 C34 H43 N5 O3 Cl

604.3041 1.1 1.8 10.5 C33 H47 N O7 Cl

604.3033 1.9 3.1 6.5 C28 H48 N5 O5 Cl2

Compound 28

Elemental Composition Report

Single Mass Analysis

Tolerance = 2.0 mDa / DBE: min = -20.0, max = 20.0

Element prediction: Off

Monoisotopic Mass, Even Electron Ions

412 formula(e) evaluated with 2 results within limits (all results (up to 1000) for each mass)

Elements Used:

C: 15-50 H: 20-60 N: 1-5 O: 1-12 Cl: 1-2

Minimum:				-20.0	
Maximum:		2.0	10.0	20.0	
Mass	Calc. Mass	mDa	PPM	DBE	Formula
624.2485	624.2477	0.8	1.3	15.5	C33 H39 N3 O7 Cl
	624.2495	-1.0	-1.6	10.5	C32 H44 N O7 Cl2

Compound 29

Elemental Composition Report

Single Mass Analysis

Tolerance = 2.0 mDa / DBE: min = -20.0, max = 20.0

Element prediction: Off

Monoisotopic Mass, Even Electron Ions

612 formula(e) evaluated with 5 results within limits (all results (up to 1000) for each mass)

Elements Used:

C: 15-50 H: 20-60 N: 1-5 O: 1-12 F: 1-3

Minimum:				-20.0	
Maximum:		2.0	10.0	20.0	
Mass	Calc. Mass	mDa	PPM	DBE	Formula
592.3085	592.3086	-0.1	-0.2	10.5	C32 H44 N O7 F2
	592.3088	-0.3	-0.5	19.5	C36 H39 N5 O2 F
	592.3074	1.1	1.9	14.5	C35 H43 N O6 F
	592.3097	-1.2	-2.0	6.5	C29 H45 N O8 F3
	592.3099	-1.4	-2.4	15.5	C33 H40 N5 O3 F2

Compound 30

Elemental Composition Report

Single Mass Analysis

Tolerance = 2.0 mDa / DBE: min = -20.0, max = 20.0

Element prediction: Off

Monoisotopic Mass, Even Electron Ions

412 formula(e) evaluated with 3 results within limits (all results (up to 1000) for each mass)

Elements Used:

C: 15-50 H: 20-60 N: 1-5 O: 1-12 Cl: 1-2

Minimum:				-20.0	
Maximum:		2.0	10.0	20.0	
Mass	Calc. Mass	mDa	PPM	DBE	Formula
624.2502	624.2508	-0.6	-1.0	15.5	C33 H40 N5 O3 Cl2
	624.2495	0.7	1.1	10.5	C32 H44 N O7 Cl2
	624.2517	-1.5	-2.4	19.5	C38 H39 N O5 Cl

Compound 31

Elemental Composition Report

Single Mass Analysis

Tolerance = 2.0 mDa / DBE: min = -20.0, max = 20.0

Element prediction: Off

Monoisotopic Mass, Even Electron Ions

412 formula(e) evaluated with 2 results within limits (all results (up to 1000) for each mass)

Elements Used:

C: 15-50 H: 20-60 N: 1-5 O: 1-12 Cl: 1-2

Minimum:				-20.0	
Maximum:		2.0	10.0	20.0	
Mass	Calc. Mass	mDa	PPM	DBE	Formula
624.2485	624.2477	0.8	1.3	15.5	C33 H39 N3 O7 Cl
	624.2495	-1.0	-1.6	10.5	C32 H44 N O7 Cl2

Compound 32

Elemental Composition Report

Single Mass Analysis

Tolerance = 2.0 mDa / DBE: min = -20.0, max = 20.0

Element prediction: Off

Monoisotopic Mass, Even Electron Ions

612 formula(e) evaluated with 5 results within limits (all results (up to 1000) for each mass)

Elements Used:

C: 15-50 H: 20-60 N: 1-5 O: 1-12 F: 1-3

Minimum:				-20.0	
Maximum:		2.0	10.0	20.0	
Mass	Calc. Mass	mDa	PPM	DBE	Formula
592.3085	592.3086	-0.1	-0.2	10.5	C32 H44 N O7 F2
	592.3088	-0.3	-0.5	19.5	C36 H39 N5 O2 F
	592.3074	1.1	1.9	14.5	C35 H43 N O6 F
	592.3097	-1.2	-2.0	6.5	C29 H45 N O8 F3
	592.3099	-1.4	-2.4	15.5	C33 H40 N5 O3 F2

Compound 33

Elemental Composition Report

Single Mass Analysis

Tolerance = 2.0 mDa / DBE: min = -20.0, max = 20.0

Element prediction: Off

Monoisotopic Mass, Even Electron Ions

631 formula(e) evaluated with 4 results within limits (all results (up to 1000) for each mass)

Elements Used:

C: 15-50 H: 20-60 N: 1-5 O: 1-12 S: 1-3

Minimum:				-20.0	
Maximum:		2.0	10.0	20.0	
Mass	Calc. Mass	mDa	PPM	DBE	Formula
562.2834	562.2832	0.2	0.4	0.5	C22 H48 N3 O9 S2
	562.2838	-0.4	-0.7	9.5	C30 H44 N O7 S
	562.2847	-1.3	-2.3	8.5	C31 H48 N O2 S3
	562.2852	-1.8	-3.2	14.5	C31 H40 N5 O3 S

Compound 34

Elemental Composition Report

Single Mass Analysis

Tolerance = 2.0 mDa / DBE: min = -20.0, max = 20.0

Element prediction: Off

Monoisotopic Mass, Even Electron Ions

595 formula(e) evaluated with 4 results within limits (all results (up to 1000) for each mass)

Elements Used:

C: 15-50 H: 20-60 N: 1-5 O: 1-12 S: 1-3 Cl: 1-1

Minimum: -20.0

Maximum: 2.0 10.0 20.0

Mass	Calc. Mass	mDa	PPM	DBE	Formula
596.2442	596.2442	0.0	0.0	0.5	C22 H47 N3 O9 S2 Cl
596.2449		-0.7	-1.2	9.5	C30 H43 N O7 S Cl
596.2457		-1.5	-2.5	8.5	C31 H47 N O2 S3 Cl
596.2424		1.8	3.0	13.5	C34 H43 N O2 S2 Cl

Compound 35

Elemental Composition Report

Single Mass Analysis

Tolerance = 2.0 mDa / DBE: min = -20.0, max = 20.0

Element prediction: Off

Monoisotopic Mass, Even Electron Ions

208 formula(e) evaluated with 2 results within limits (all results (up to 1000) for each mass)

Elements Used:

C: 15-50 H: 20-60 N: 1-5 O: 1-12

Minimum:				-20.0	
Maximum:		2.0	10.0	20.0	
Mass	Calc. Mass	mDa	PPM	DBE	Formula
564.3553	564.3550	0.3	0.5	12.5	C32 H46 N5 O4
	564.3536	1.7	3.0	7.5	C31 H50 N O8

Compound 36

Elemental Composition Report

Single Mass Analysis

Tolerance = 2.0 mDa / DBE: min = -20.0, max = 20.0

Element prediction: Off

Monoisotopic Mass, Even Electron Ions

200 formula(e) evaluated with 1 results within limits (all results (up to 1000) for each mass)

Elements Used:

C: 15-50 H: 20-60 N: 1-5 O: 1-12

Minimum:				-20.0	
Maximum:		2.0	10.0	20.0	
Mass	Calc. Mass	mDa	PPM	DBE	Formula
557.3232	557.3227	0.5	0.9	10.5	C31 H45 N2 O7

Compound 37

Elemental Composition Report

Single Mass Analysis

Tolerance = 2.0 mDa / DBE: min = -20.0, max = 20.0

Element prediction: Off

Monoisotopic Mass, Even Electron Ions

832 formula(e) evaluated with 6 results within limits (all results (up to 1000) for each mass)

Elements Used:

C: 15-50 H: 20-60 N: 1-5 O: 1-12 S: 0-3

Minimum:				-20.0	
Maximum:		2.0	10.0	20.0	
Mass	Calc. Mass	mDa	PPM	DBE	Formula
604.2930	604.2931	-0.1	-0.2	-7.5	C16 H54 N5 O12 S3
	604.2938	-0.8	-1.3	1.5	C24 H50 N3 O10 S2
	604.2919	1.1	1.8	14.5	C36 H46 N O3 S2
	604.2944	-1.4	-2.3	10.5	C32 H46 N O8 S
	604.2913	1.7	2.8	5.5	C28 H50 N3 O5 S3
	604.2910	2.0	3.3	15.5	C35 H42 N O8

Compound 38

Elemental Composition Report

Single Mass Analysis

Tolerance = 2.0 mDa / DBE: min = -20.0, max = 20.0

Element prediction: Off

Monoisotopic Mass, Even Electron Ions

208 formula(e) evaluated with 1 results within limits (all results (up to 1000) for each mass)

Elements Used:

C: 15-50 H: 20-60 N: 1-5 O: 1-12

Minimum:				-20.0	
Maximum:		2.0	10.0	20.0	
Mass	Calc. Mass	mDa	PPM	DBE	Formula
624.3524	624.3536	-1.2	-1.9	12.5	C36 H50 N O8

Chasmanine

Elemental Composition Report

Single Mass Analysis

Tolerance = 2.0 mDa / DBE: min = -20.0, max = 20.0

Element prediction: Off

Monoisotopic Mass, Even Electron Ions

152 formula(e) evaluated with 2 results within limits (all results (up to 1000) for each mass)

Elements Used:

C: 15-50 H: 20-50 N: 1-5 O: 1-12

Minimum:				-20.0	
Maximum:		2.0	10.0	20.0	
Mass	Calc. Mass	mDa	PPM	DBE	Formula
452.3026	452.3026	0.0	0.0	10.5	C26 H38 N5 O2
452.3012	1.4	3.1	5.5	C25 H42 N O6	

intermdite 1

Elemental Composition Report

Single Mass Analysis

Tolerance = 2.0 mDa / DBE: min = -20.0, max = 20.0

Element prediction: Off

Monoisotopic Mass, Even Electron Ions

621 formula(e) evaluated with 3 results within limits (all results (up to 1000) for each mass)

Elements Used:

C: 15-50 H: 20-60 N: 1-5 O: 1-12 S: 1-3

Minimum:				-20.0	
Maximum:		2.0	10.0	20.0	
Mass	Calc. Mass	mDa	PPM	DBE	Formula
734.3030	734.3033	-0.3	-0.4	12.5	C37 H52 N O10 S2
	734.3026	0.4	0.5	3.5	C29 H56 N3 O12 S3
	734.3046	-1.6	-2.2	17.5	C38 H48 N5 O6 S2

intermite 2

Elemental Composition Report

Single Mass Analysis

Tolerance = 2.0 mDa / DBE: min = -20.0, max = 20.0

Element prediction: Off

Monoisotopic Mass, Even Electron Ions

208 formula(e) evaluated with 2 results within limits (all results (up to 1000) for each mass)

Elements Used:

C: 15-50 H: 20-60 N: 1-5 O: 1-12

Minimum: -20.0

Maximum: 2.0 10.0 20.0

Mass Calc. Mass mDa PPM DBE Formula

628.3483 628.3486 -0.3 -0.5 11.5 C35 H50 N O9

628.3499 -1.6 -2.5 16.5 C36 H46 N5 O5

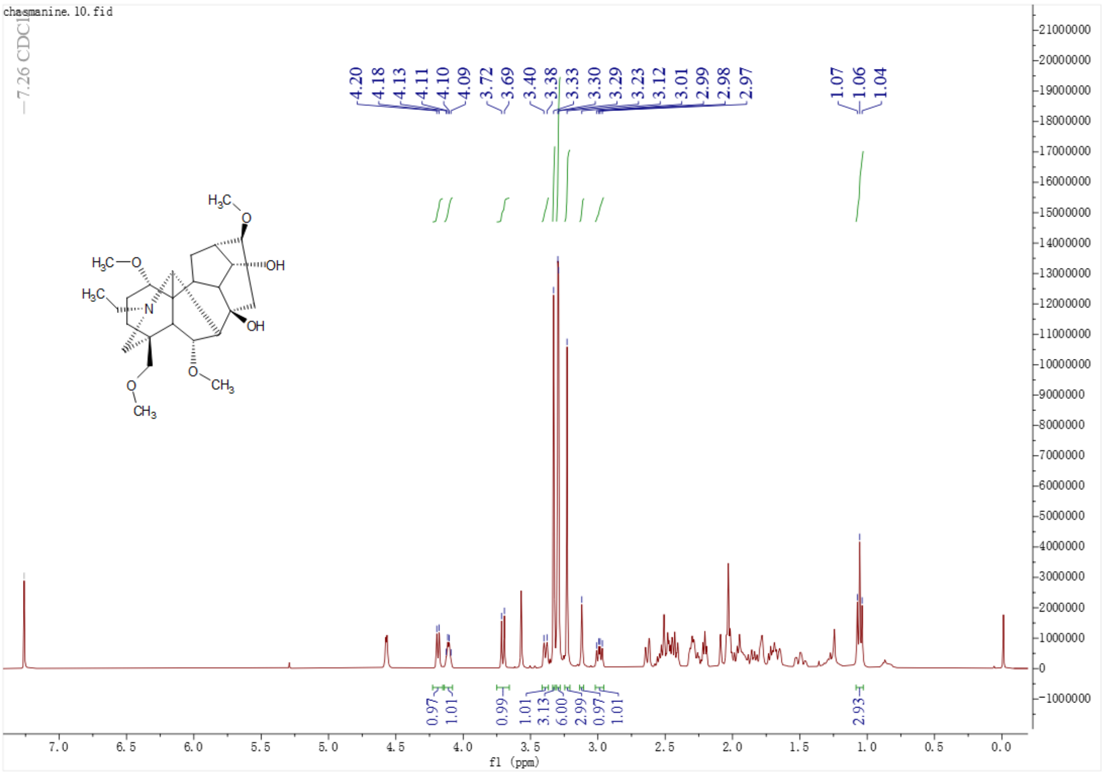

figure 1 ^{1}H NMR (400 MHz) spectrum of Chasmanine in $CDCl_3$

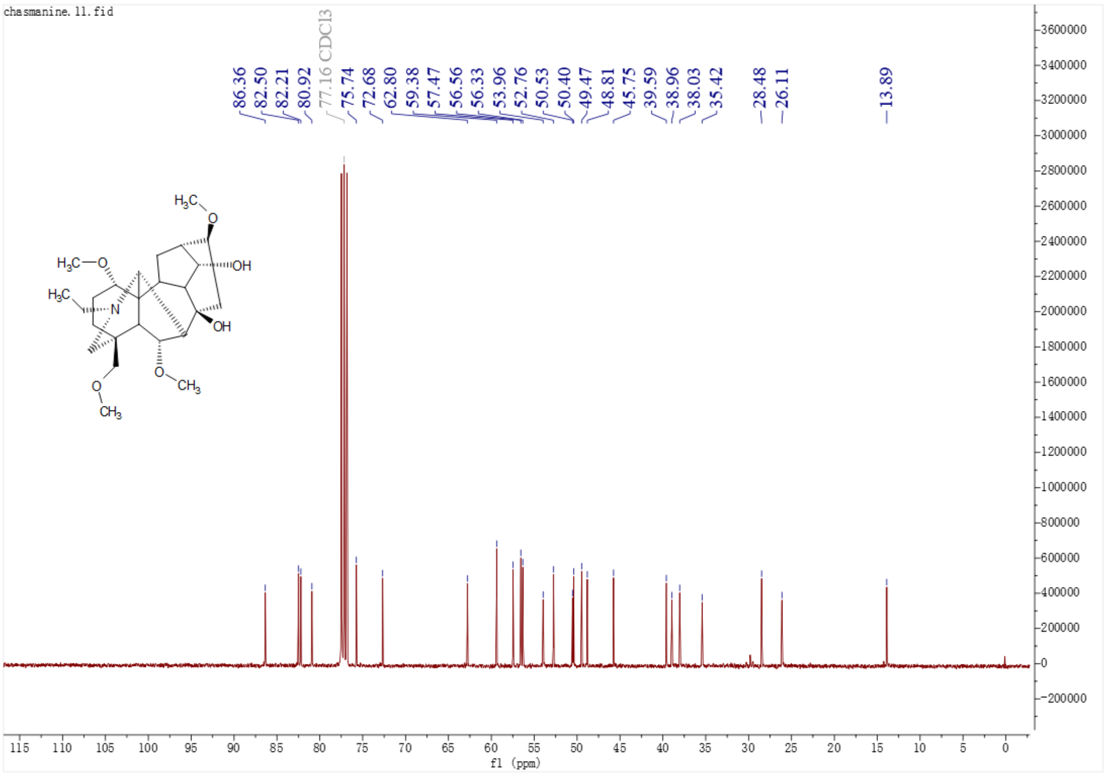

figure 2 ^{13}C NMR (100 MHz) spectrum of Chasmanine in $CDCl_3$

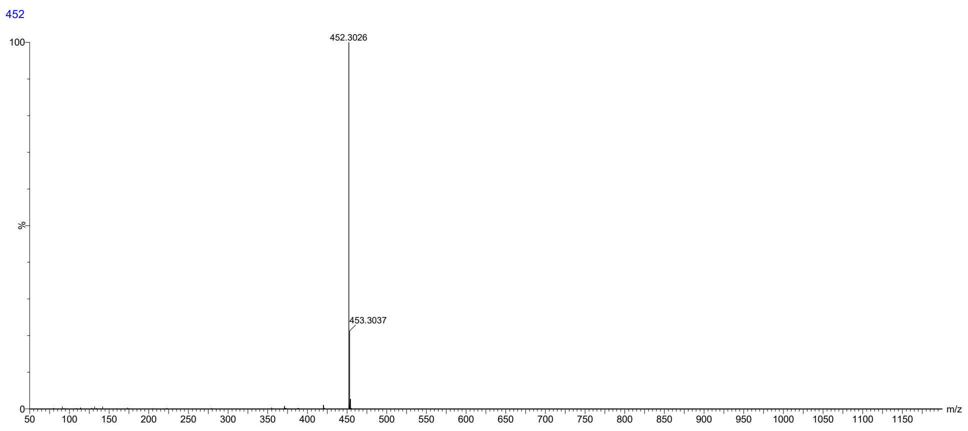

figure 3 HR-ESI-MS data of Chasmanine

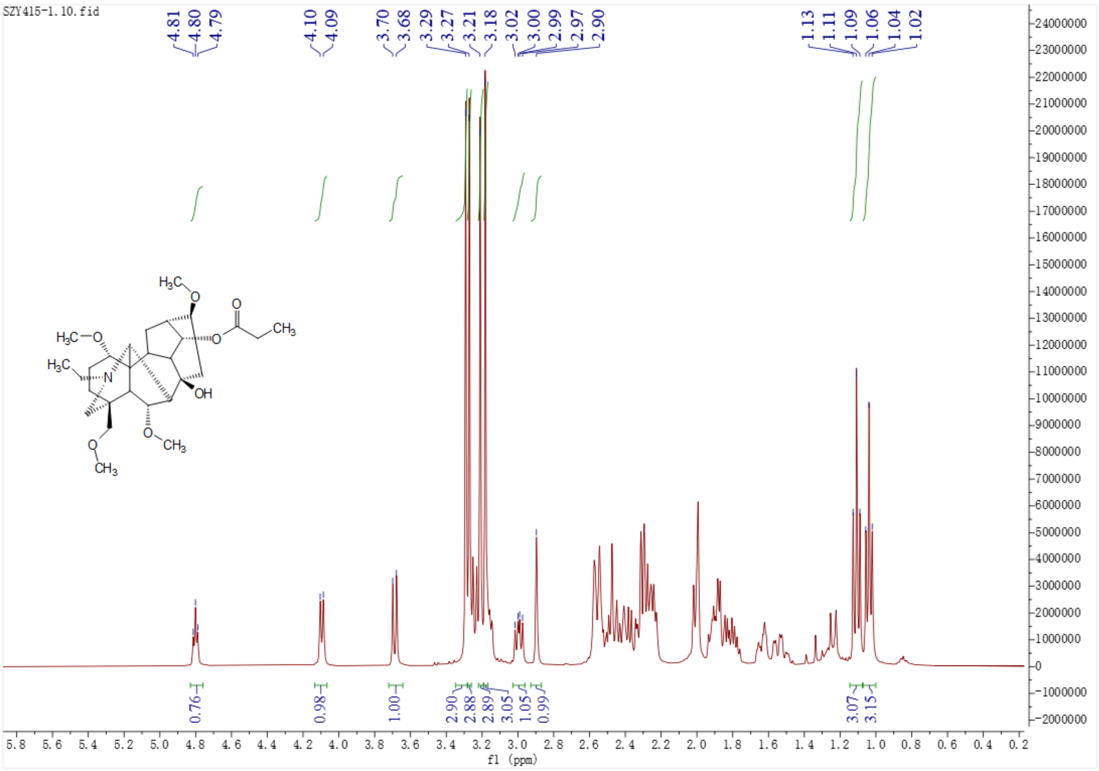

figure 4 ^{1}H NMR (400 MHz) spectrum of compound 1 in $CDCl_3$

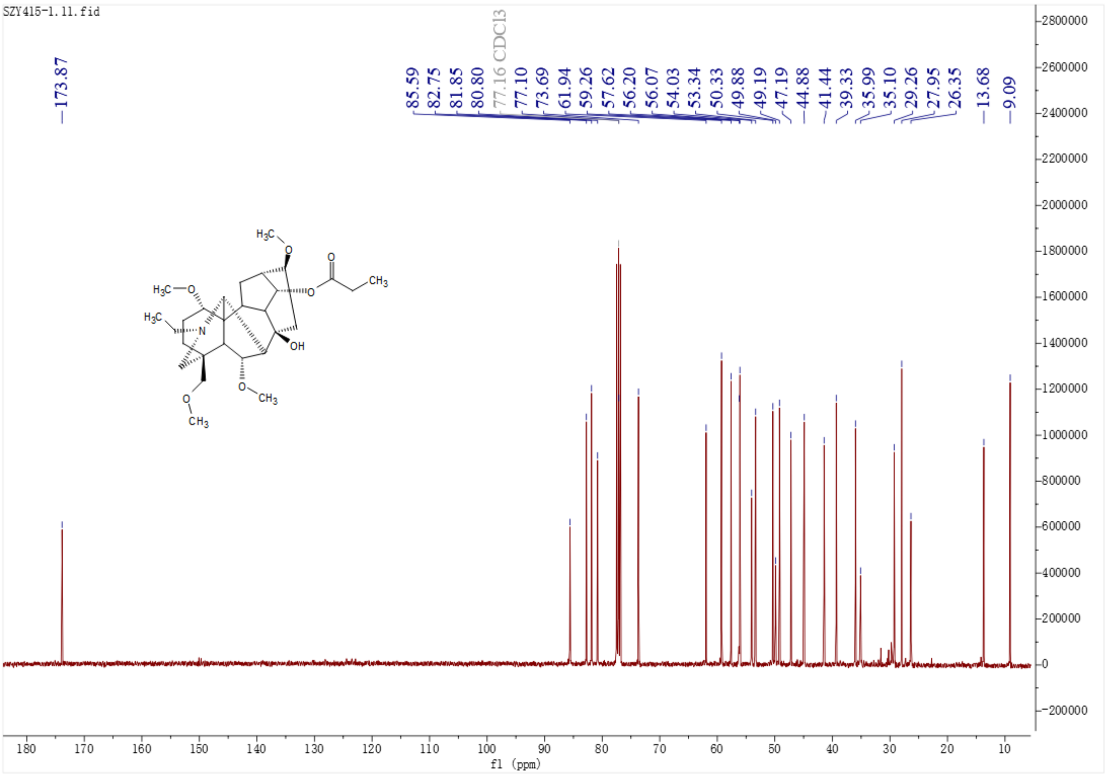

figure 5 ^{13}C NMR (100 MHz) spectrum of compound 1 in $CDCl_3$

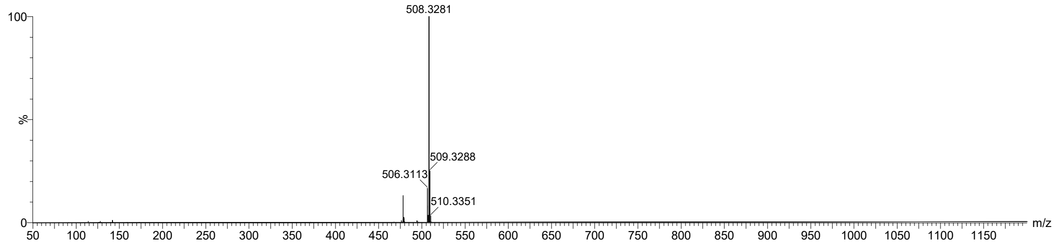

figure 6 HR-ESI-MS data of Compound 1

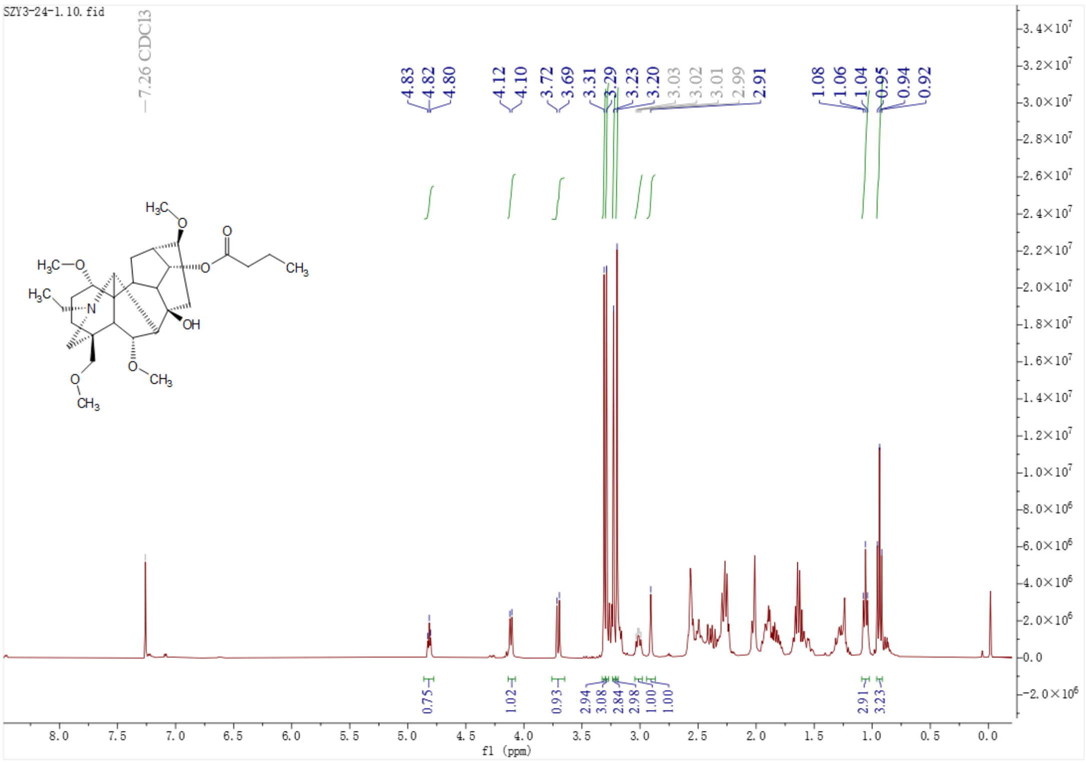

figure 7 ^{1}H NMR (400 MHz) spectrum of compound 2 in $CDCl_3$

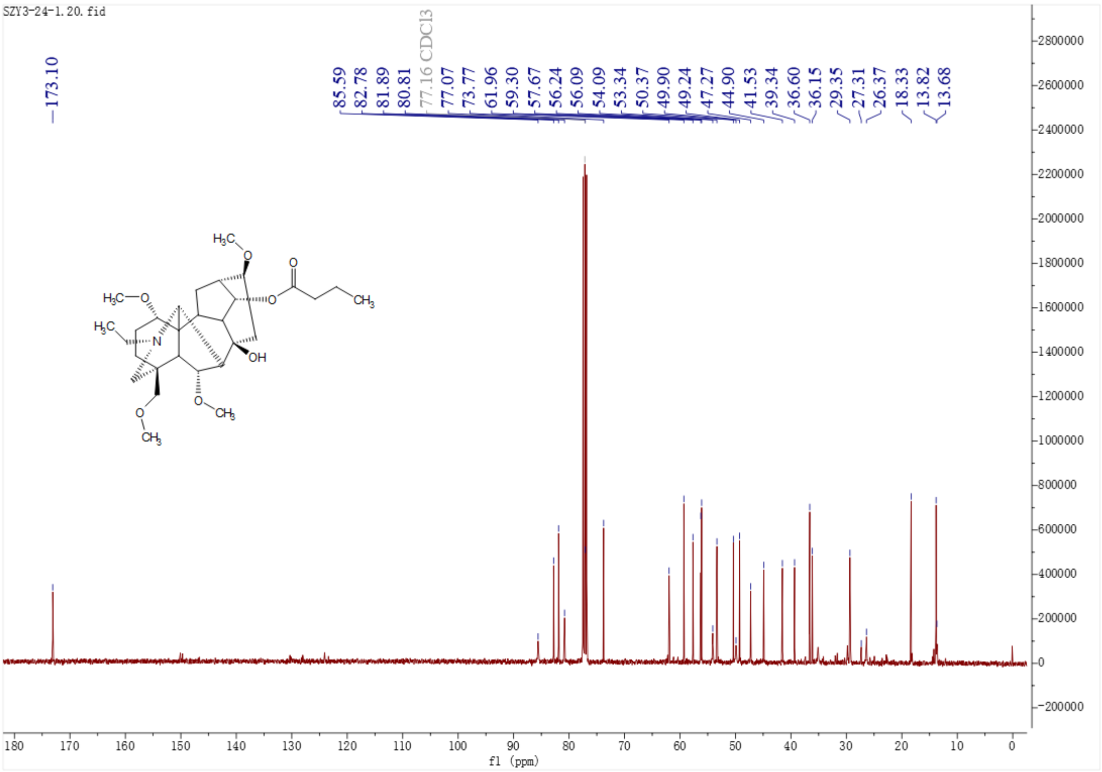

figure 8 ^{13}C NMR (100 MHz) spectrum of compound 2 in $CDCl_3$

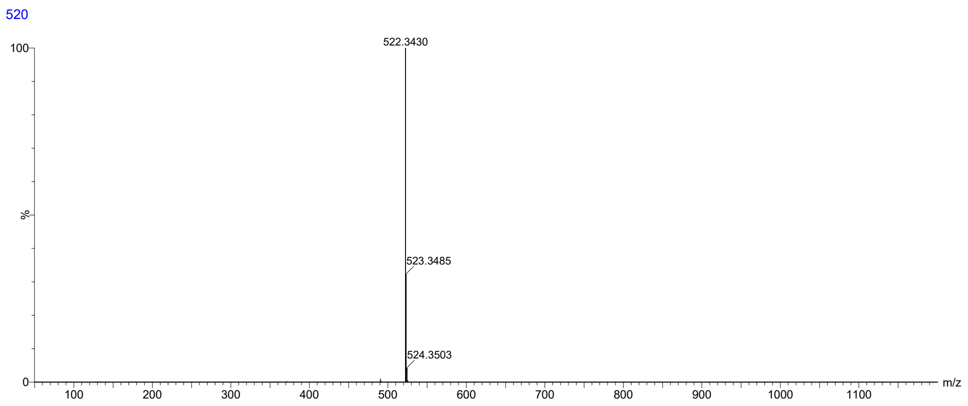

figure 9 HR-ESI-MS data of Compound 2

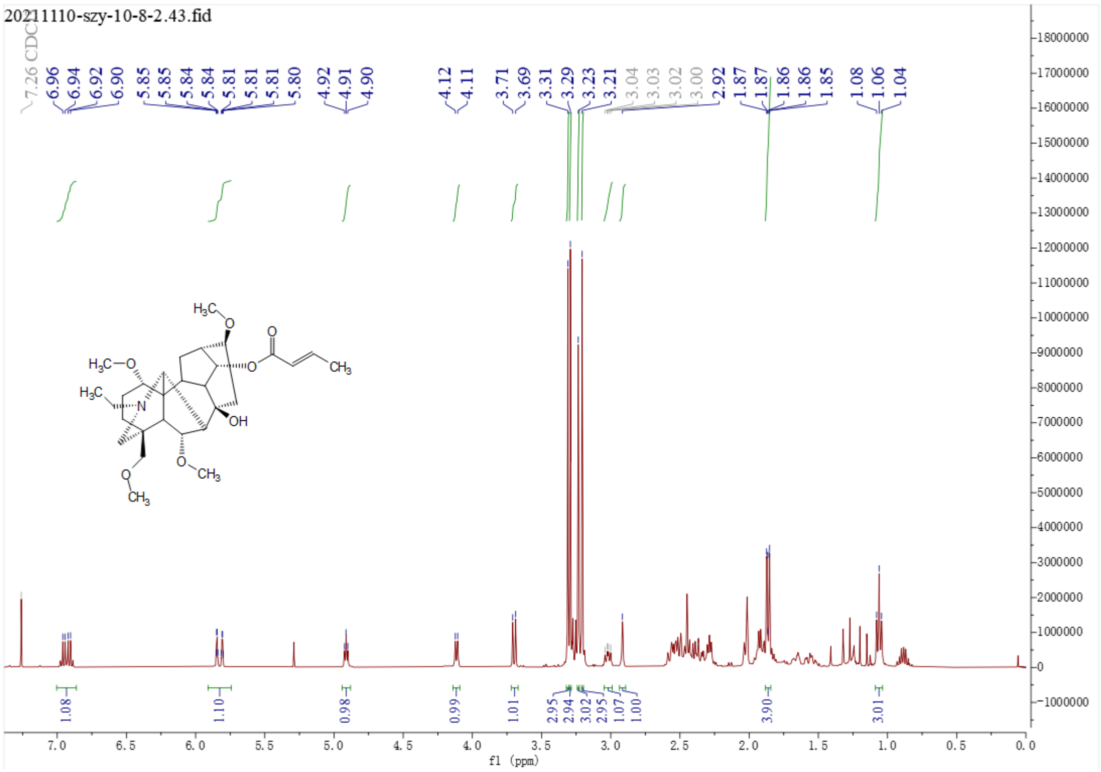

figure 10 ^{1}H NMR (400 MHz) spectrum of compound 3 in $CDCl_3$

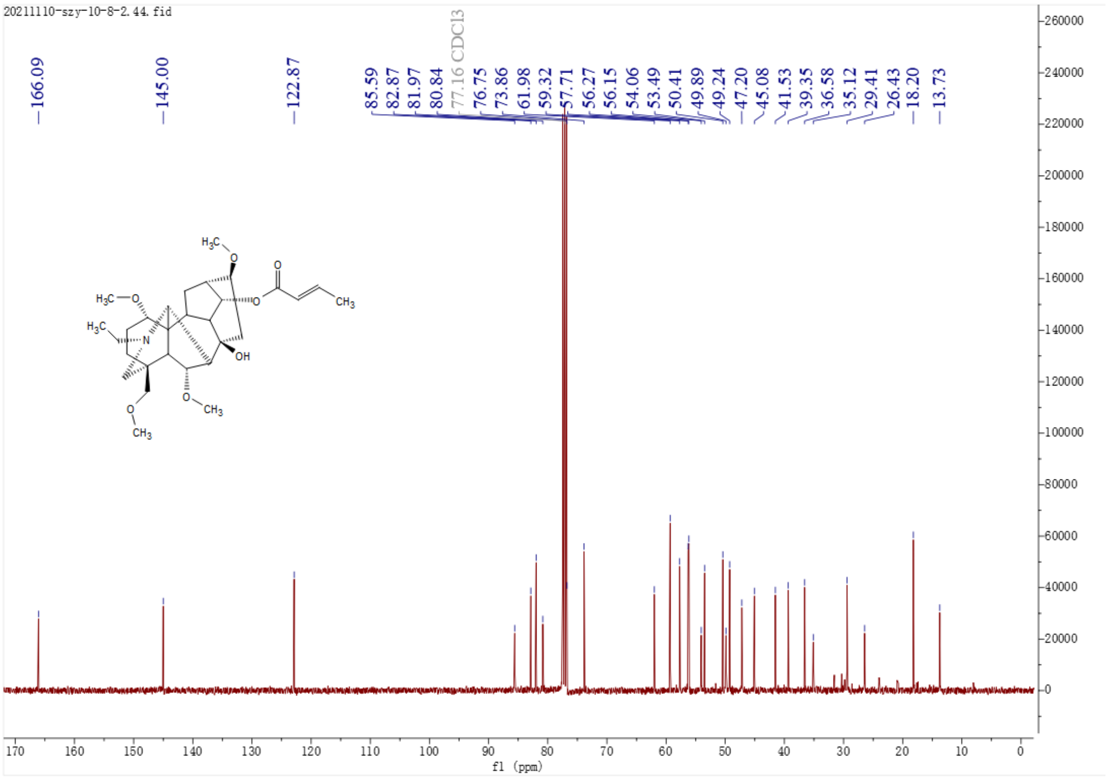

figure 11 ^{13}C NMR (100 MHz) spectrum of compound 3 in $CDCl_3$

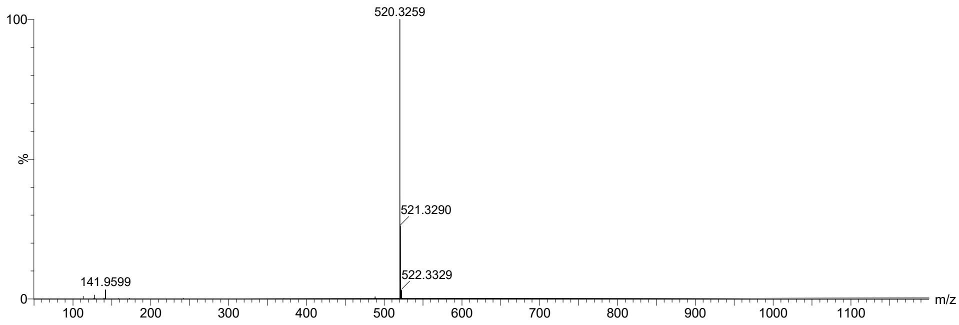

figure 12 HR-ESI-MS data of Compound 3

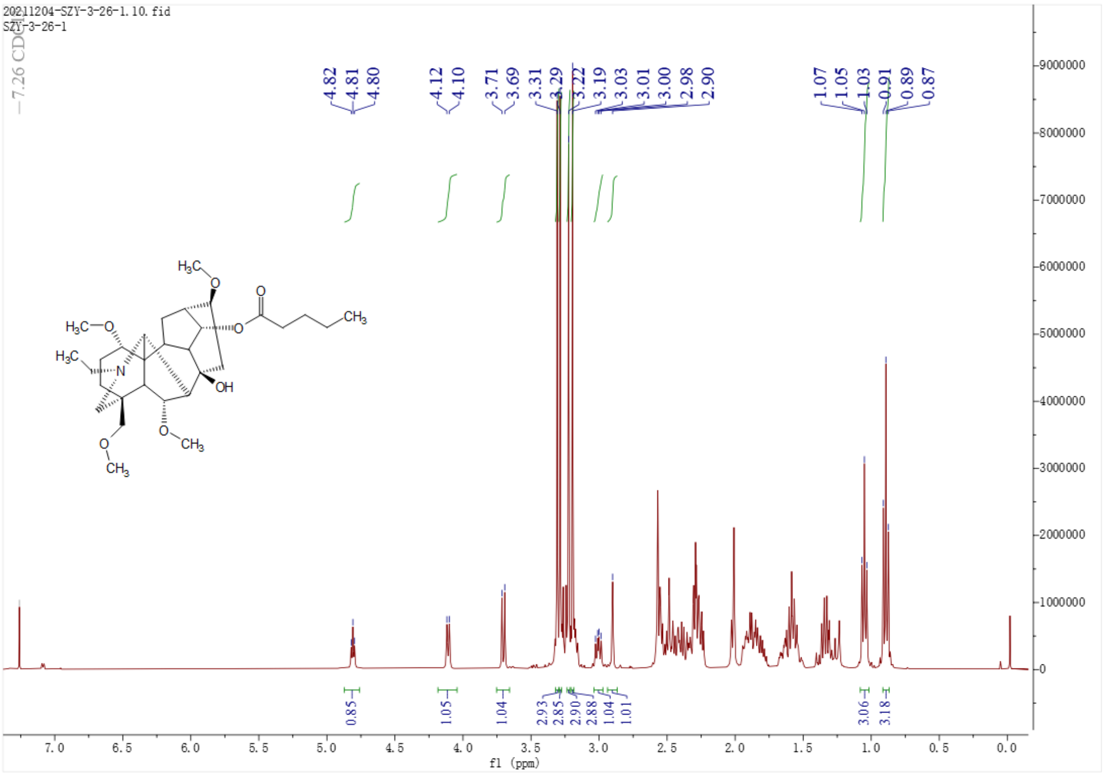

figure 13 ^{1}H NMR (400 MHz) spectrum of compound 4 in $CDCl_3$

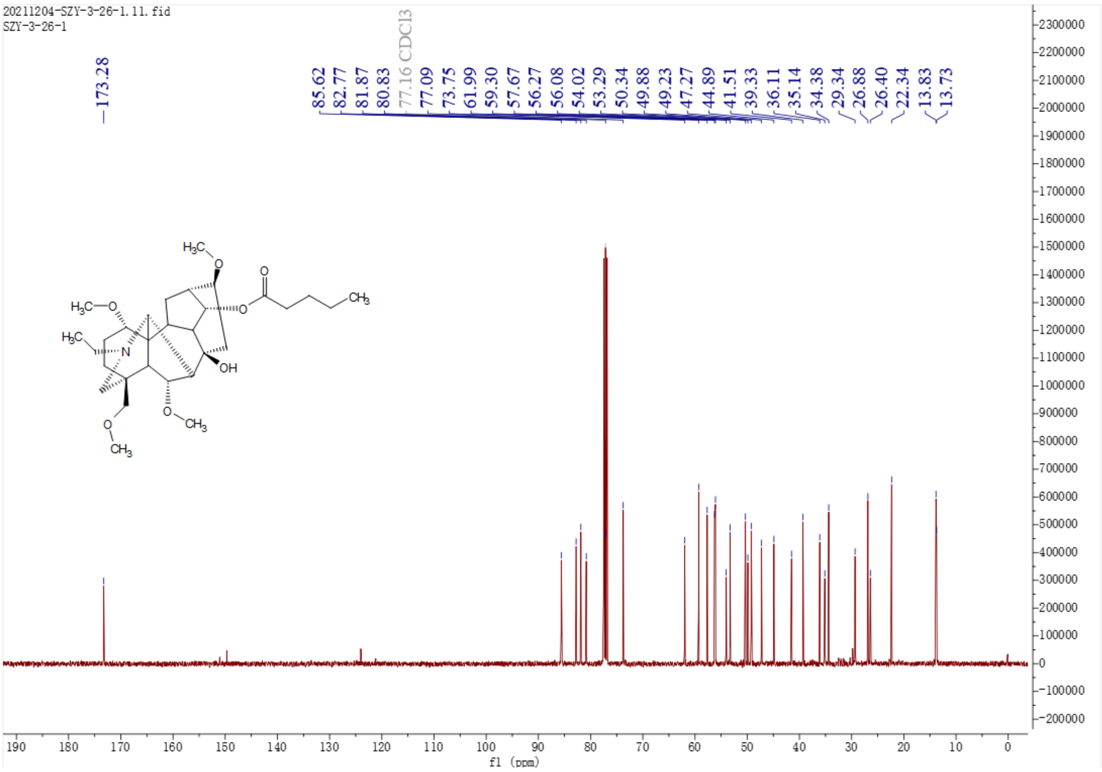

figure 14 ^{13}C NMR (100 MHz) spectrum of compound 4 in $CDCl_3$

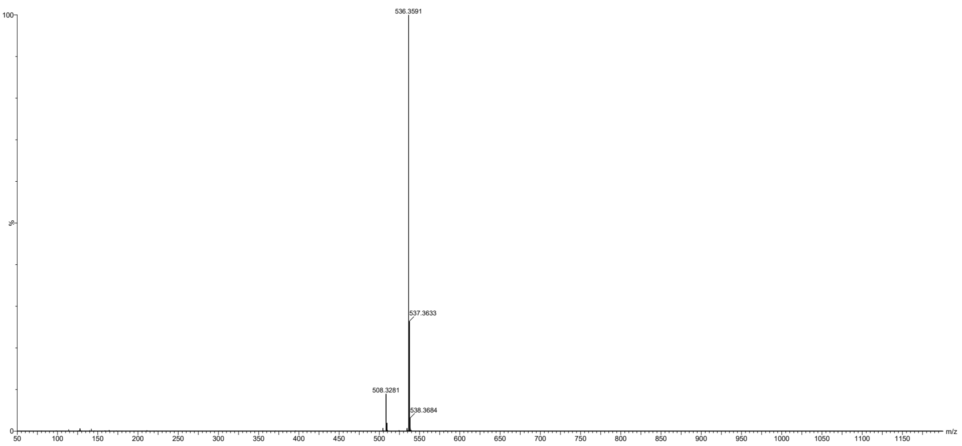

figure 15 HR-ESI-MS data of Compound 4

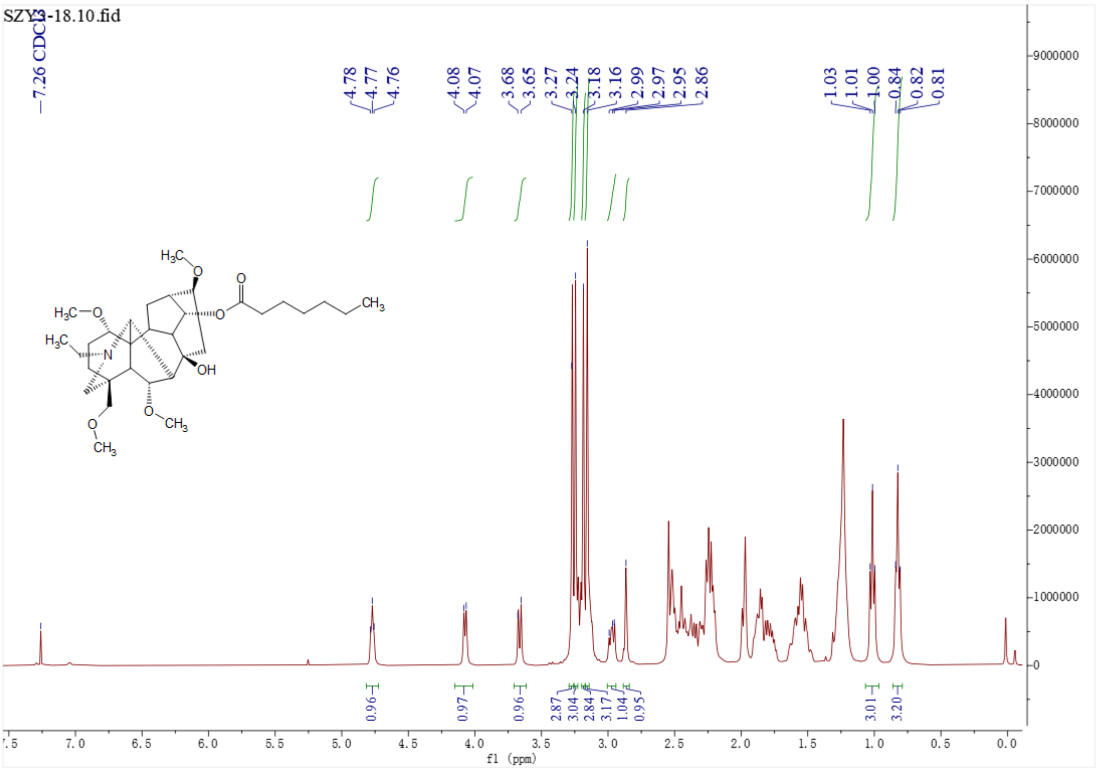

figure 16 ^1H NMR (400 MHz) spectrum of compound 5 in $CDCl_3$

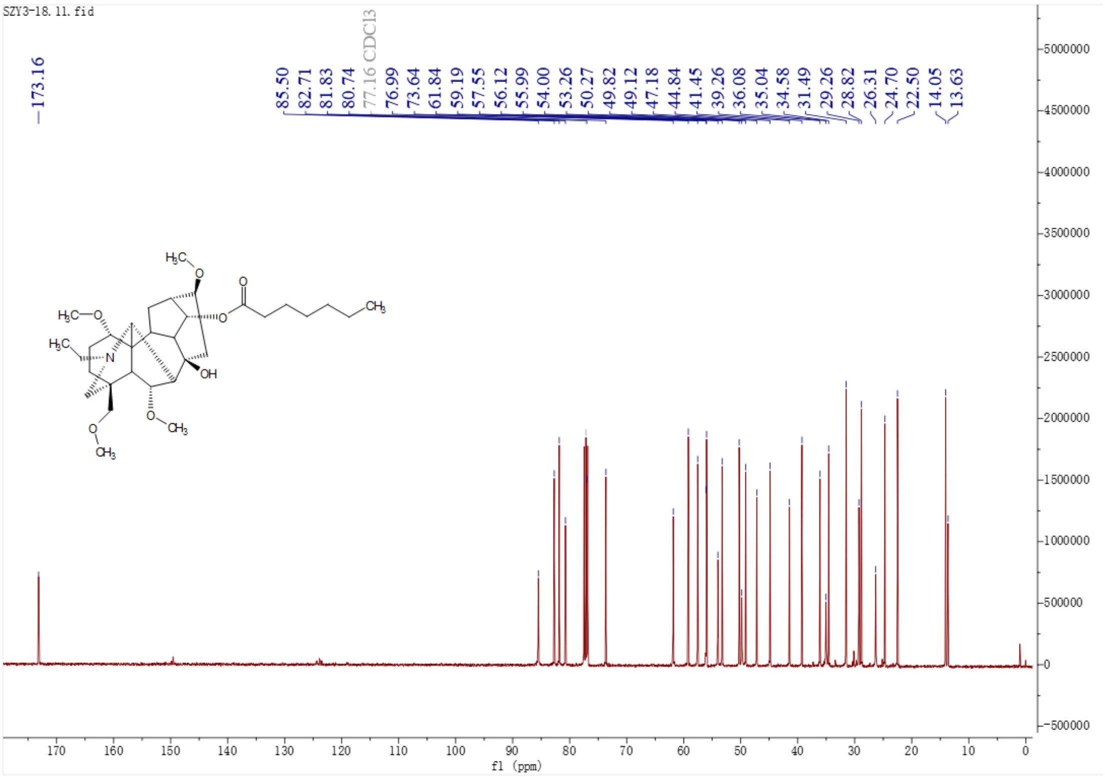

figure 17 ^{13}C NMR (100 MHz) spectrum of compound 5 in $CDCl_3$

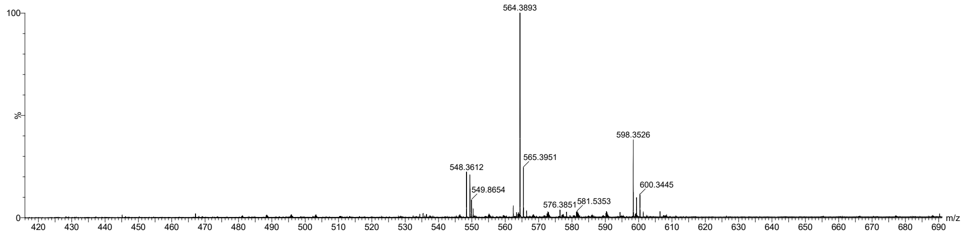

figure 18 HR-ESI-MS data of Compound 5

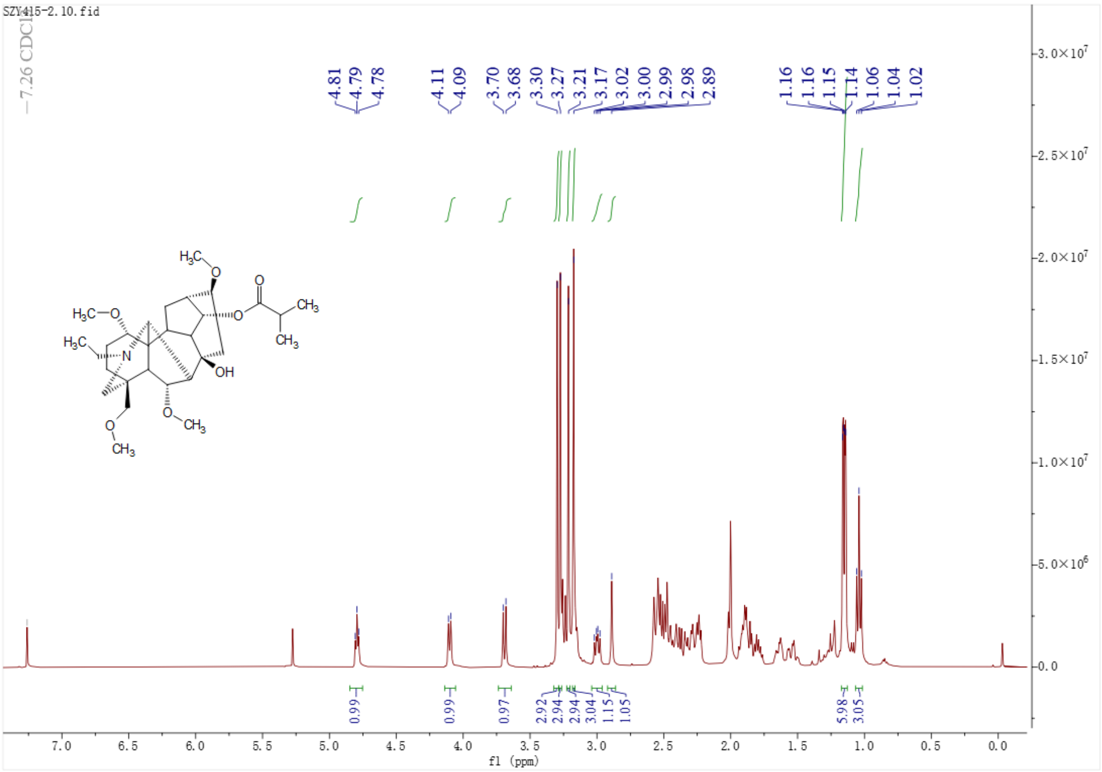

figure 19 ^{1}H NMR (400 MHz) spectrum of compound 6 in $CDCl_3$

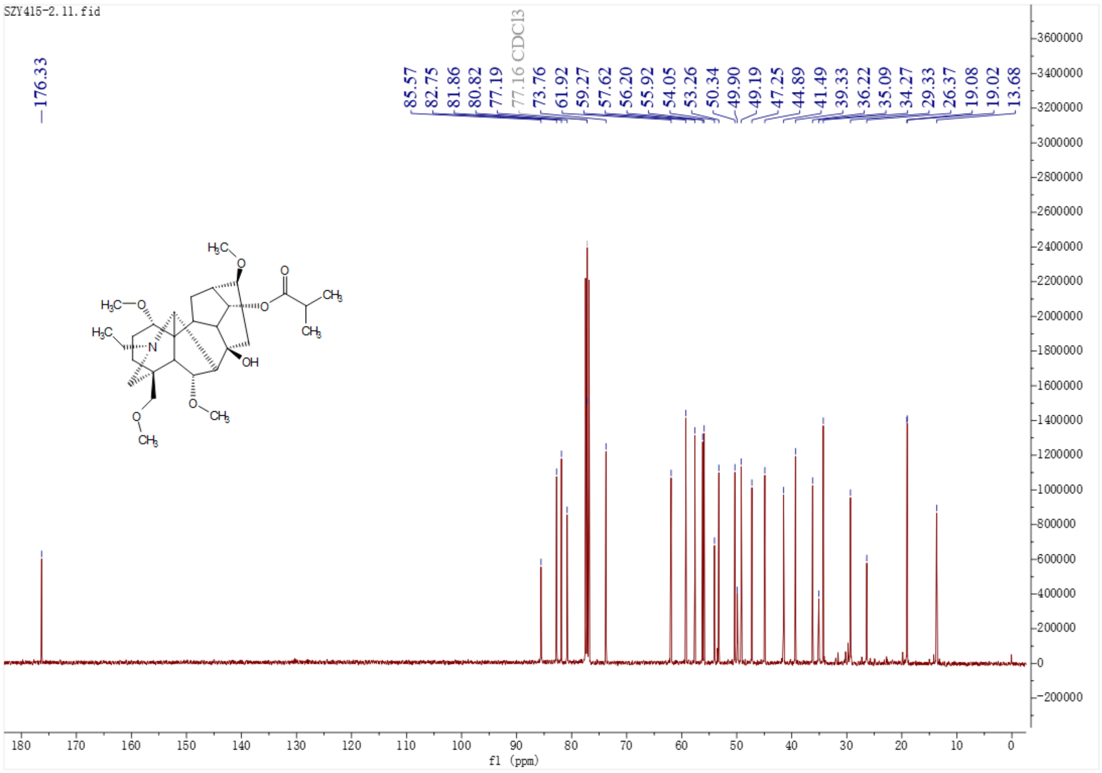

figure 20 ^{13}C NMR (100 MHz) spectrum of compound 6 in $CDCl_3$

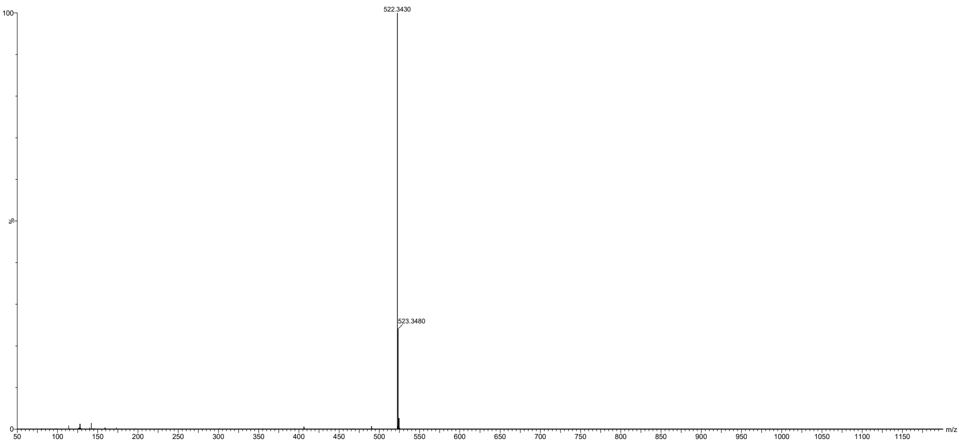

figure 21 HR-ESI-MS data of Compound 6

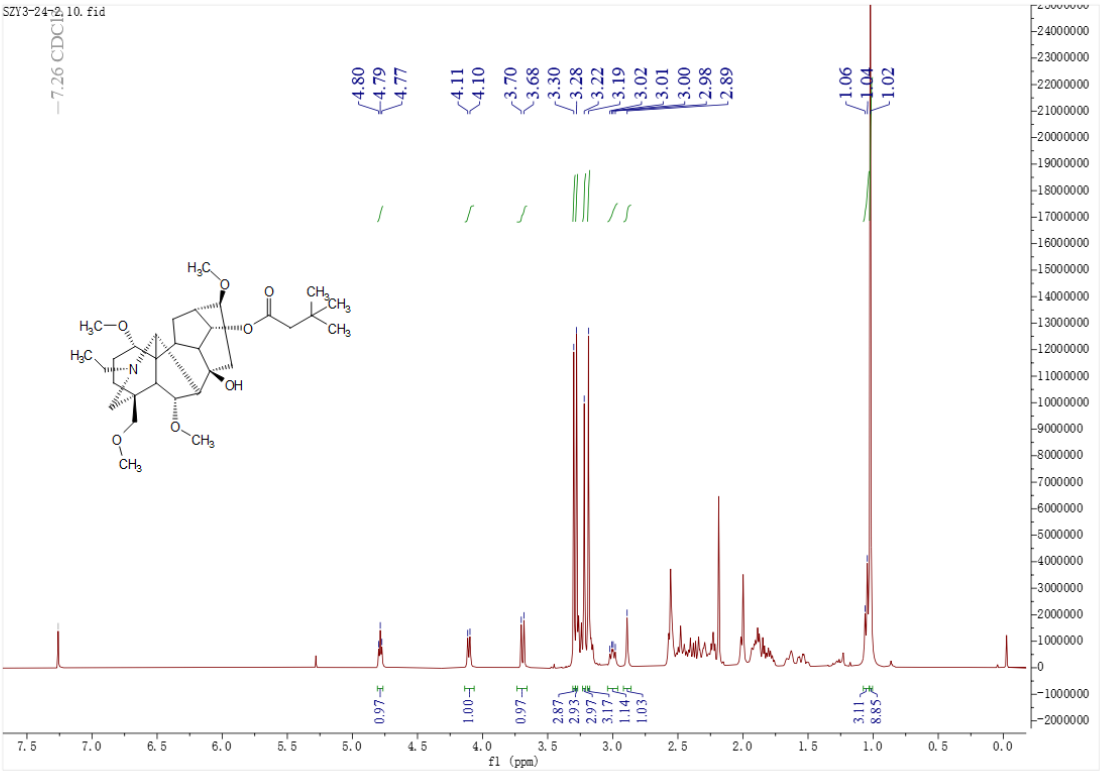

figure 22 ^{1}H NMR (400 MHz) spectrum of compound 7 in $CDCl_3$

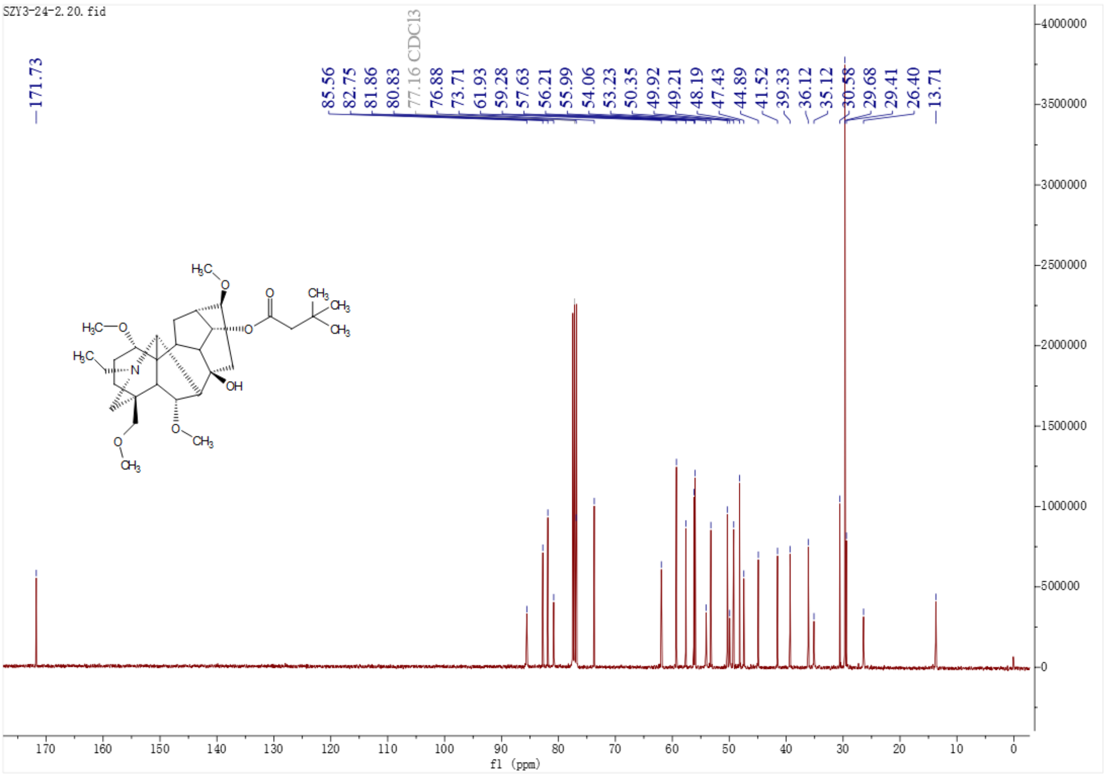

figure 23 ^{13}C NMR (100 MHz) spectrum of compound 7 in $CDCl_3$

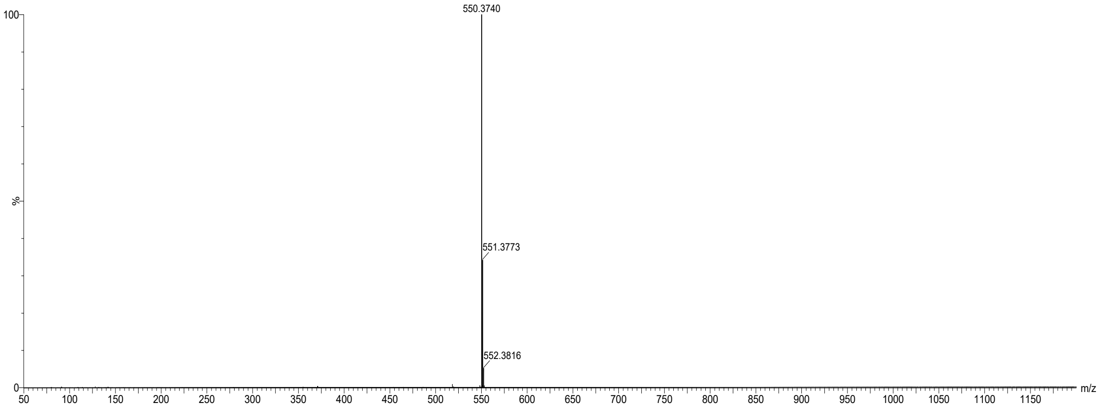

figure 24 HR-ESI-MS data of Compound 7

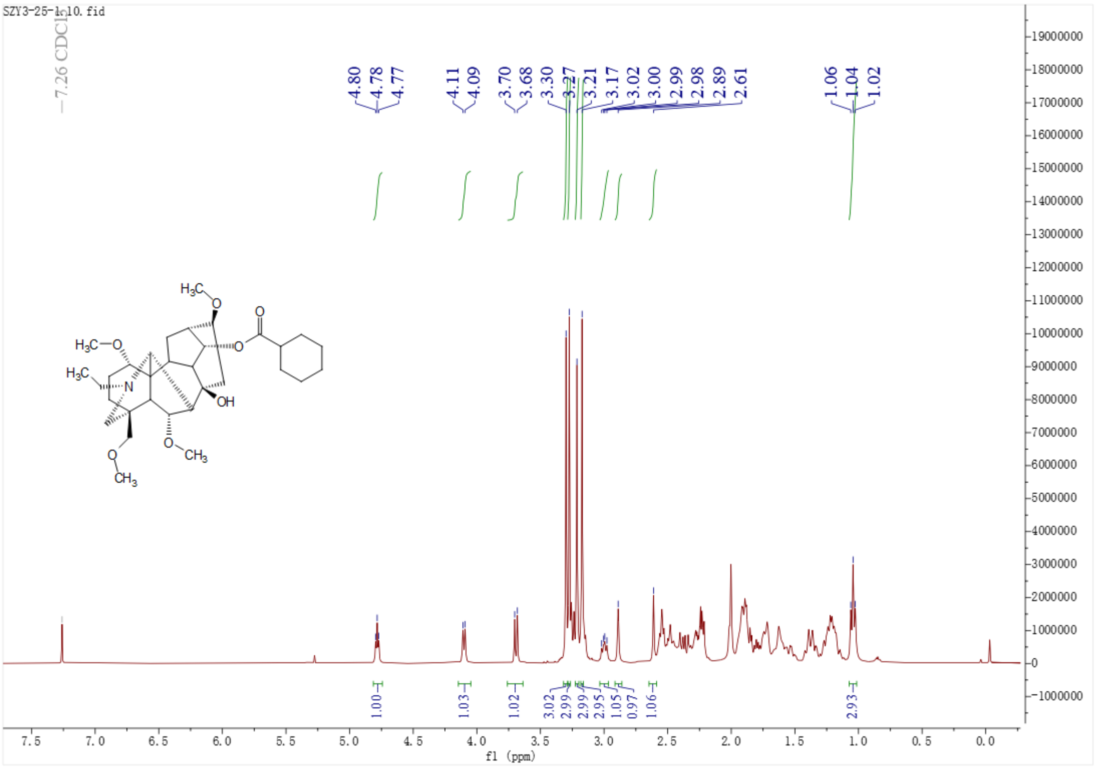

figure 25 ^{1}H NMR (400 MHz) spectrum of compound 8 in $CDCl_3$

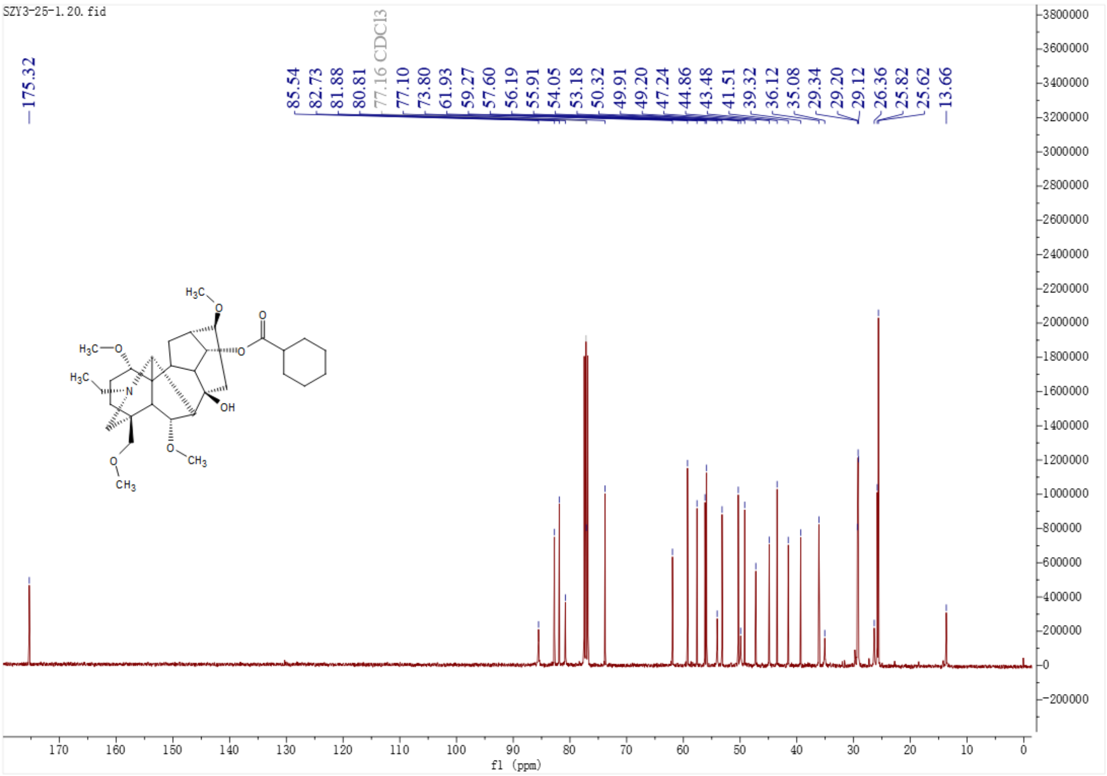

figure 26 ^{13}C NMR (100 MHz) spectrum of compound 8 in $CDCl_3$

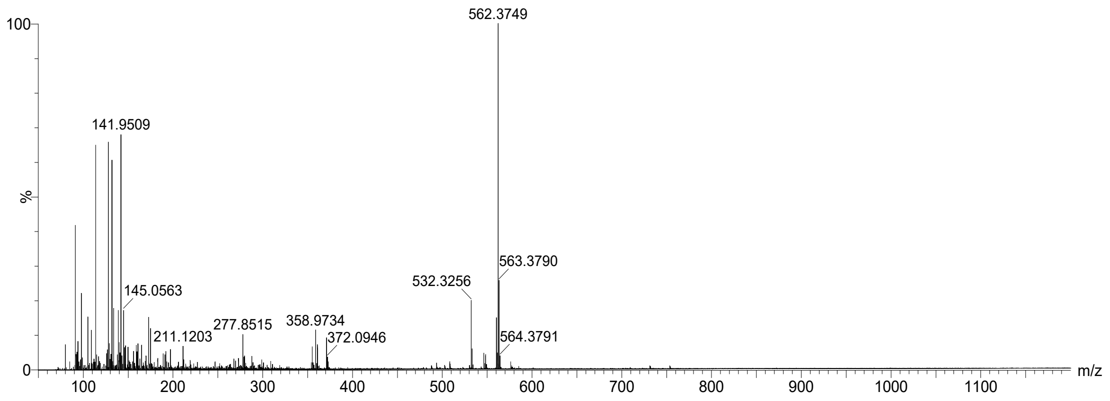

figure 27 HR-ESI-MS data of Compound 8

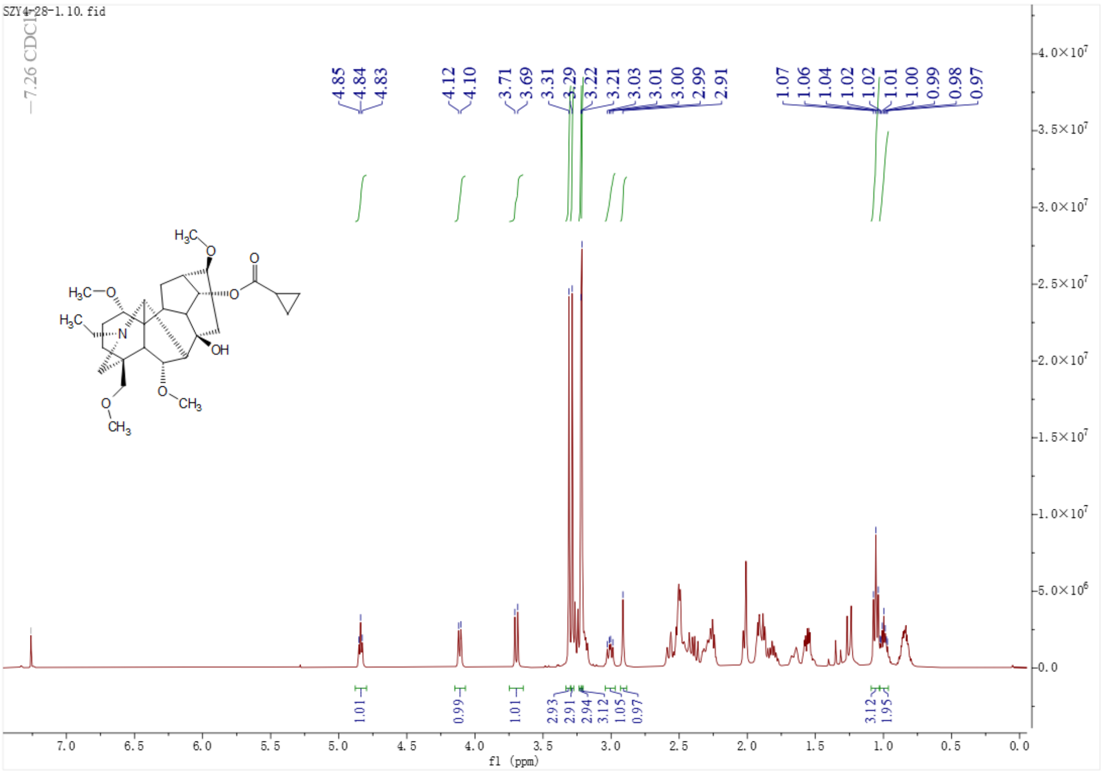

figure 28 ^{1}H NMR (400 MHz) spectrum of compound 9 in $CDCl_3$

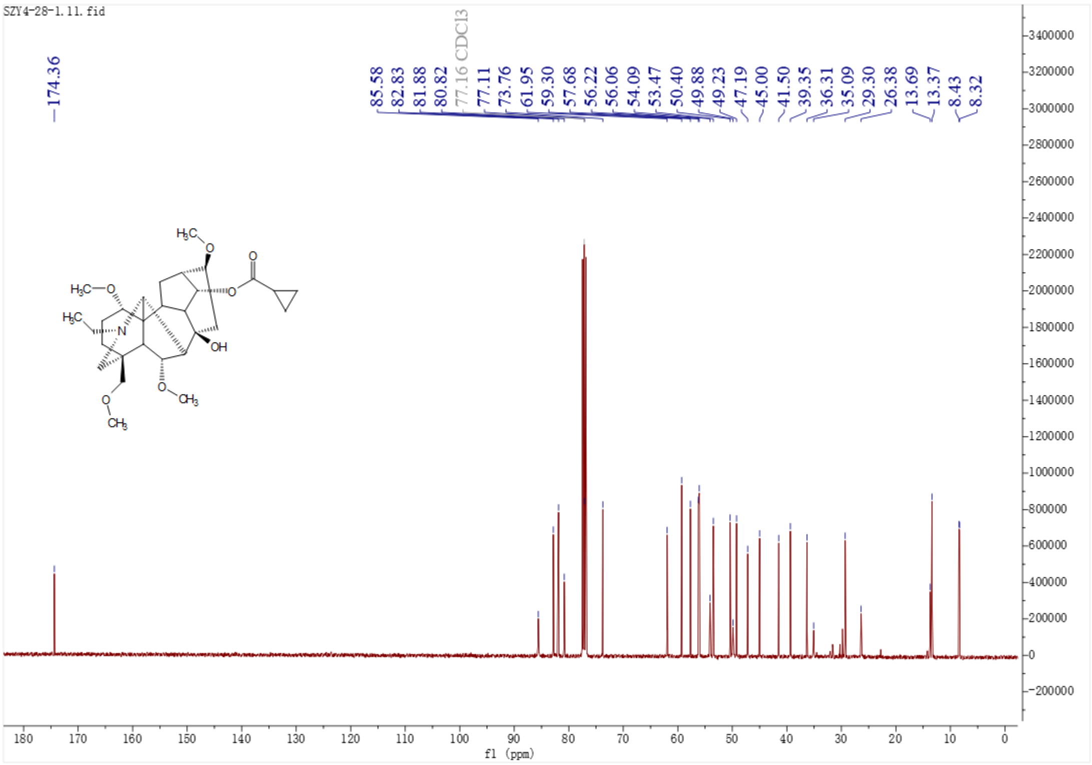

figure 29 ^{13}C NMR (100 MHz) spectrum of compound 9 in $CDCl_3$

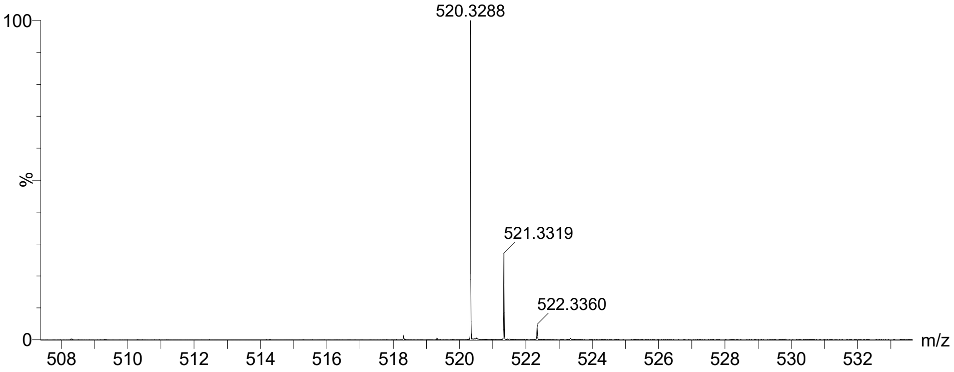

figure 30 HR-ESI-MS data of Compound 9

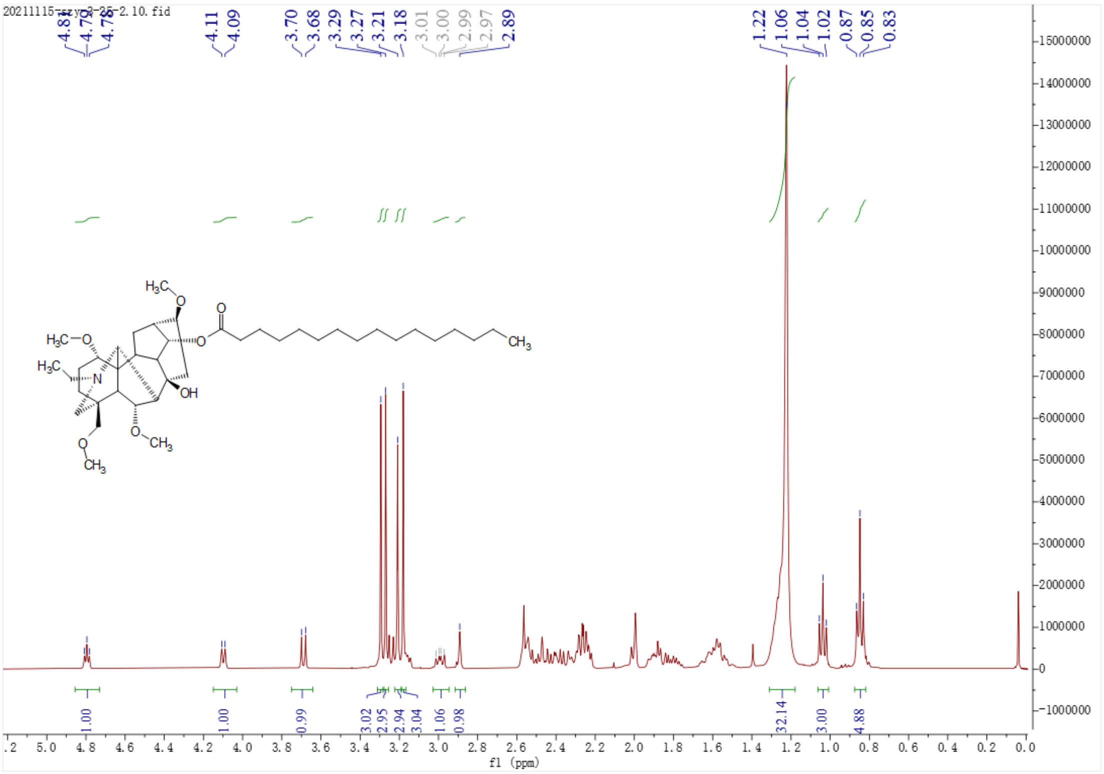

figure 31 ^1H NMR (400 MHz) spectrum of compound 10 in $CDCl_3$

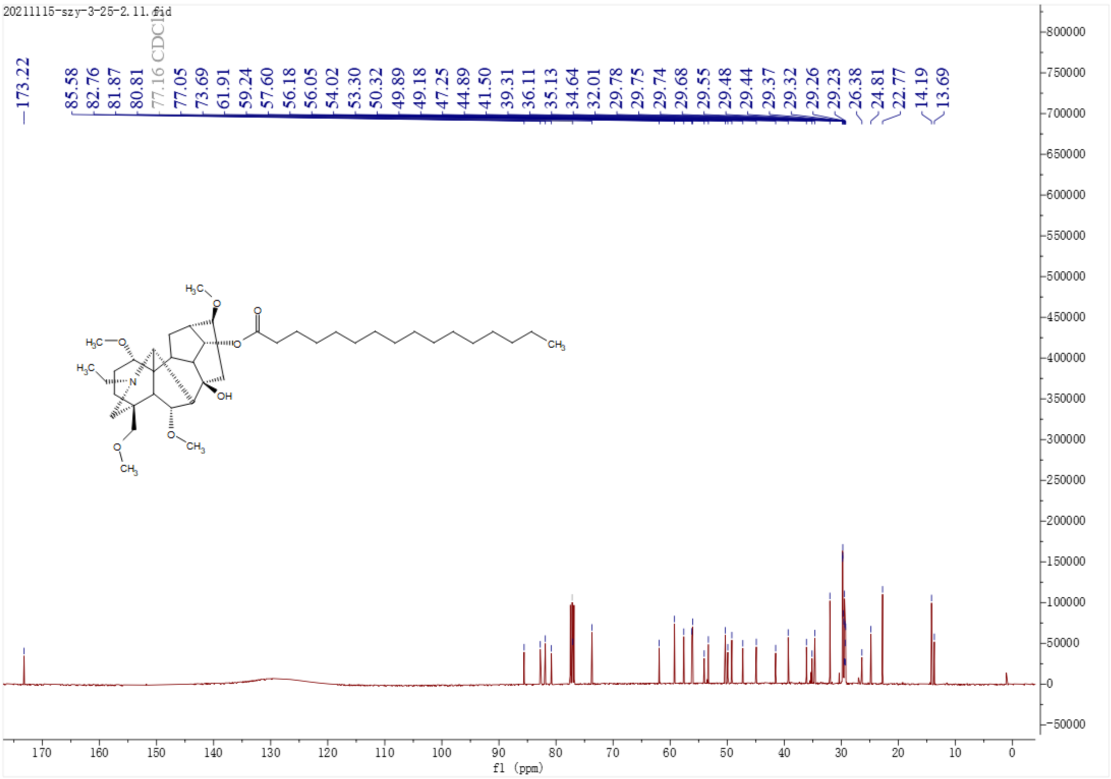

figure 32 ^{13}C NMR (100 MHz) spectrum of compound 10 in $CDCl_3$

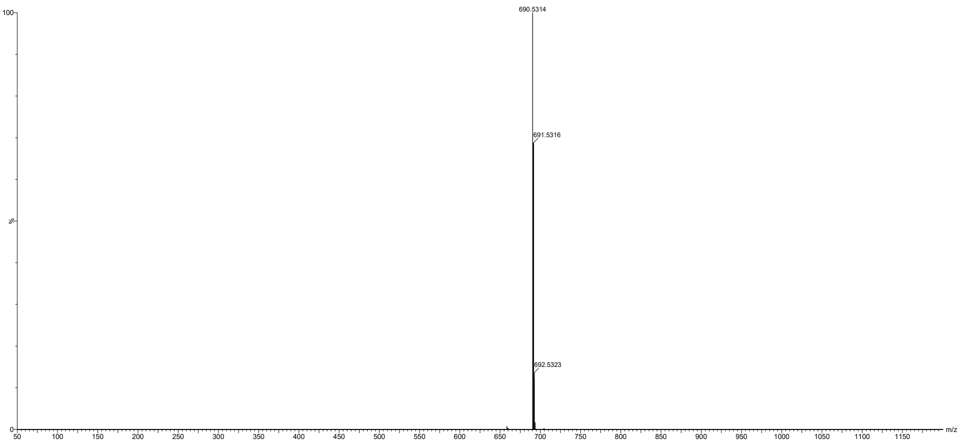

figure 33 HR-ESI-MS data of Compound 10

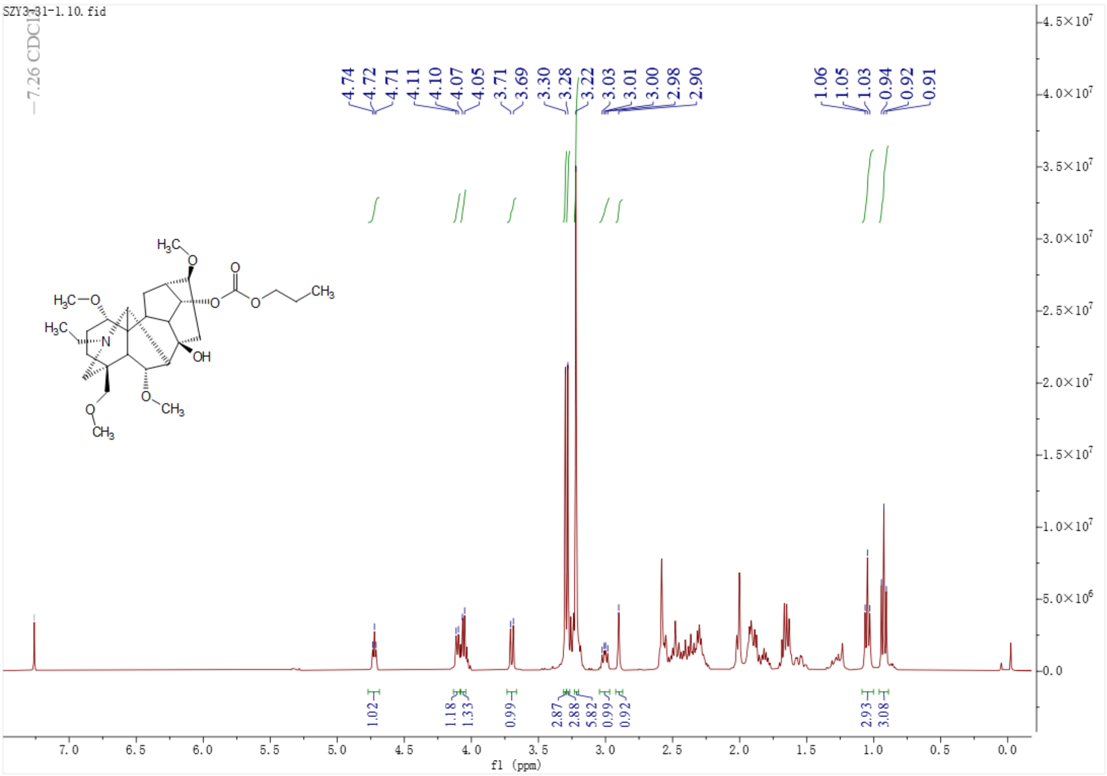

figure 34 ^{1}H NMR (400 MHz) spectrum of compound 11 in $CDCl_3$

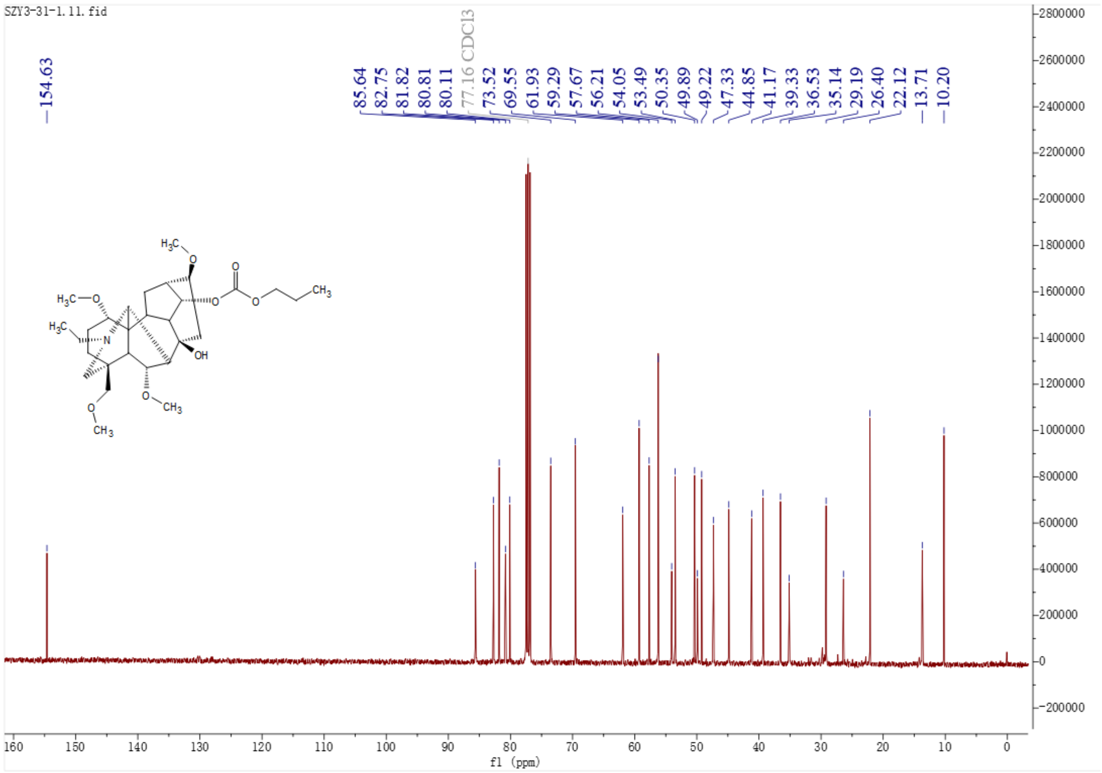

figure 35 ^{13}C NMR (100 MHz) spectrum of compound 11 in $CDCl_3$

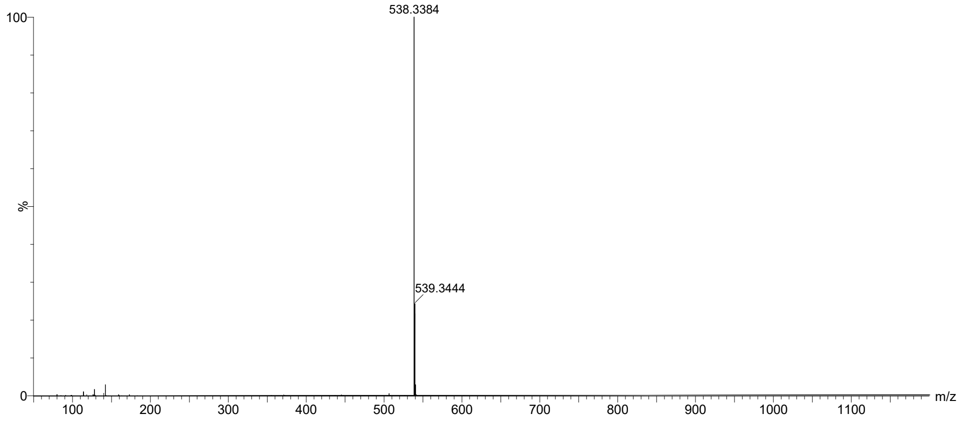

figure 36 HR-ESI-MS data of Compound 11

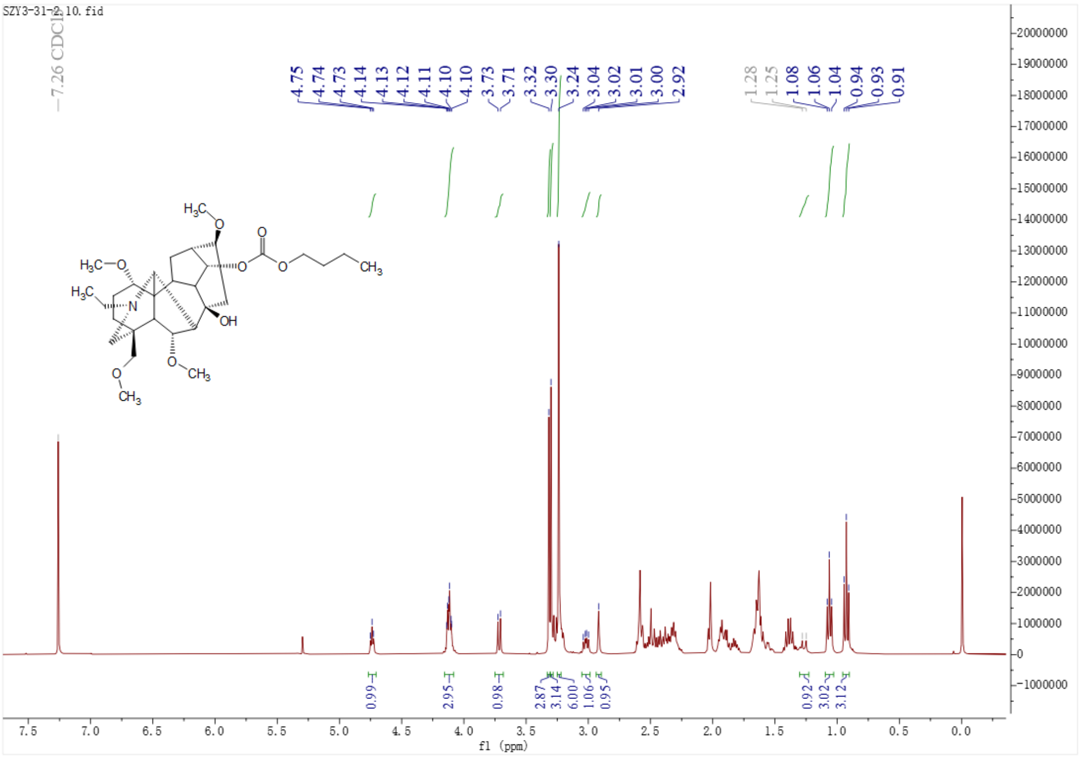

figure 37 ^{1}H NMR (400 MHz) spectrum of compound 12 in $CDCl_3$

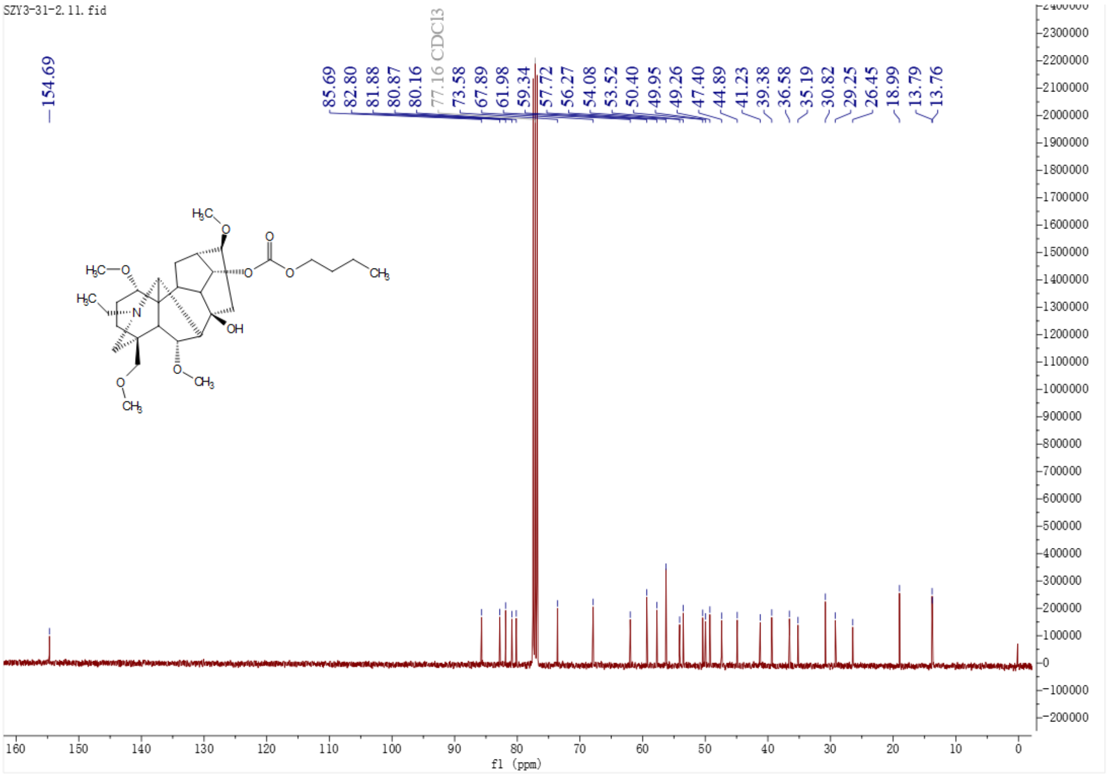

figure 38 ^{13}C NMR (100 MHz) spectrum of compound 12 in $CDCl_3$

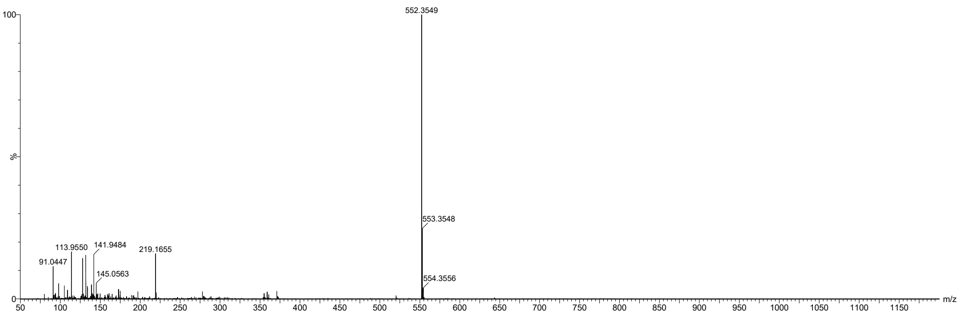

figure 39 HR-ESI-MS data of Compound 12

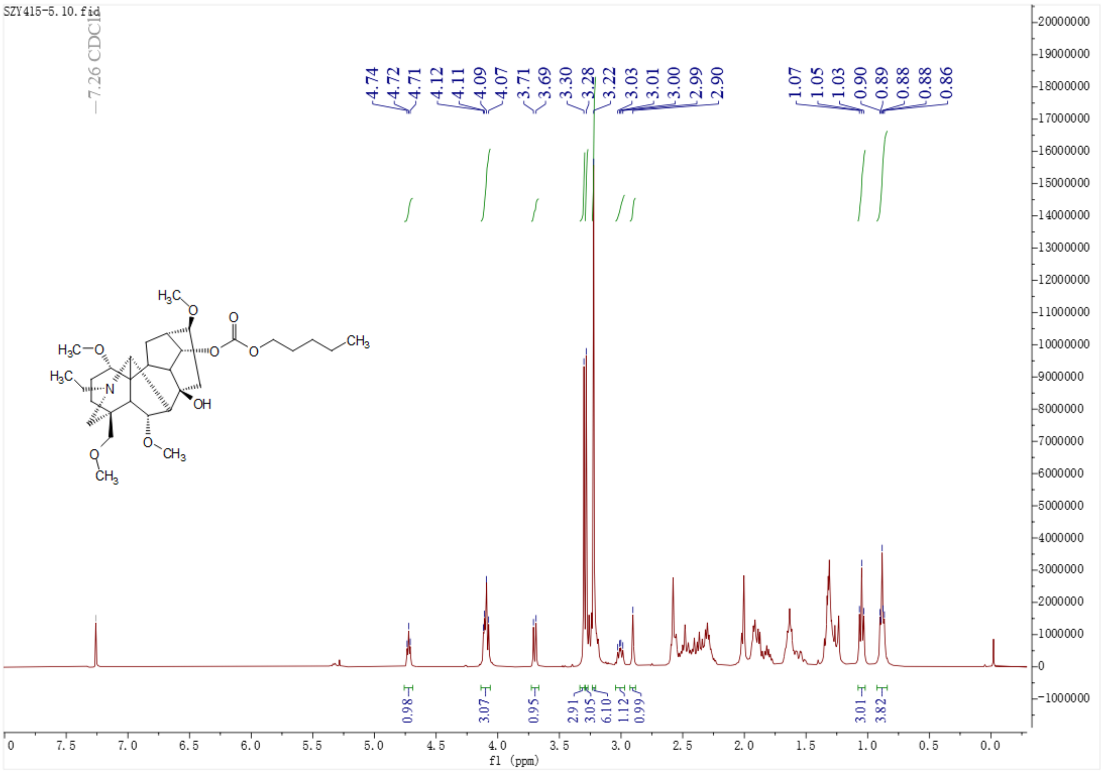

figure 40 ^{1}H NMR (400 MHz) spectrum of compound 13 in $CDCl_3$

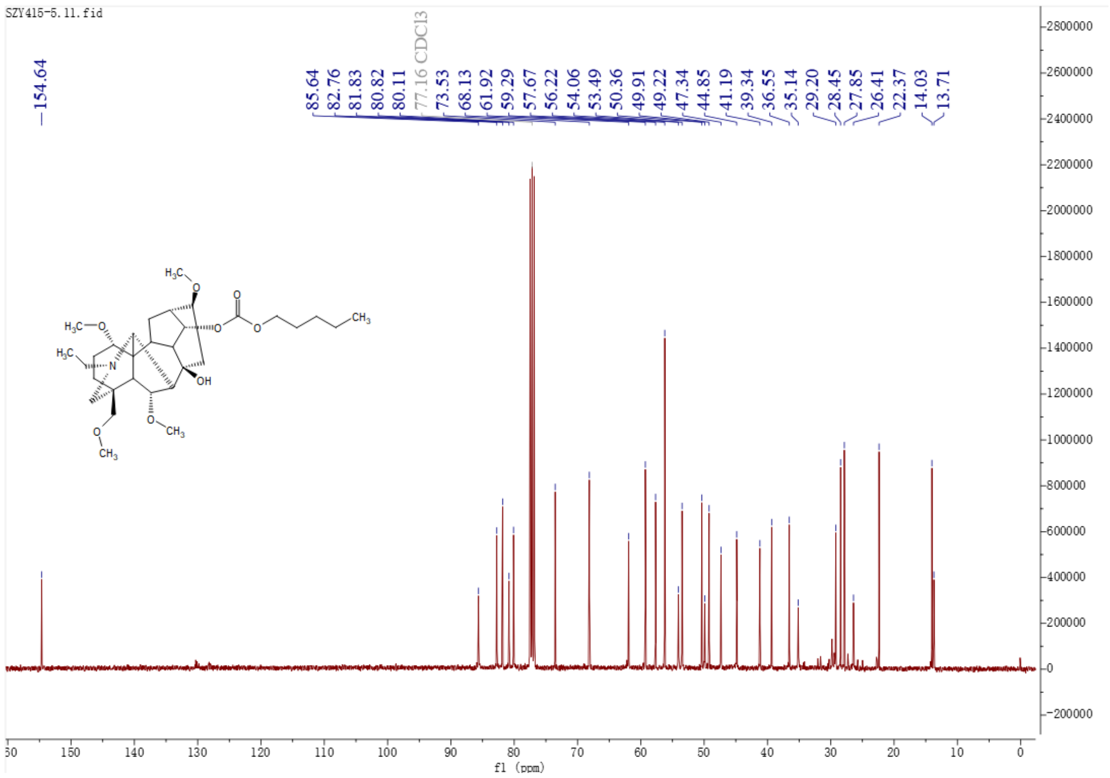

figure 41 ^{13}C NMR (100 MHz) spectrum of compound 13 in $CDCl_3$

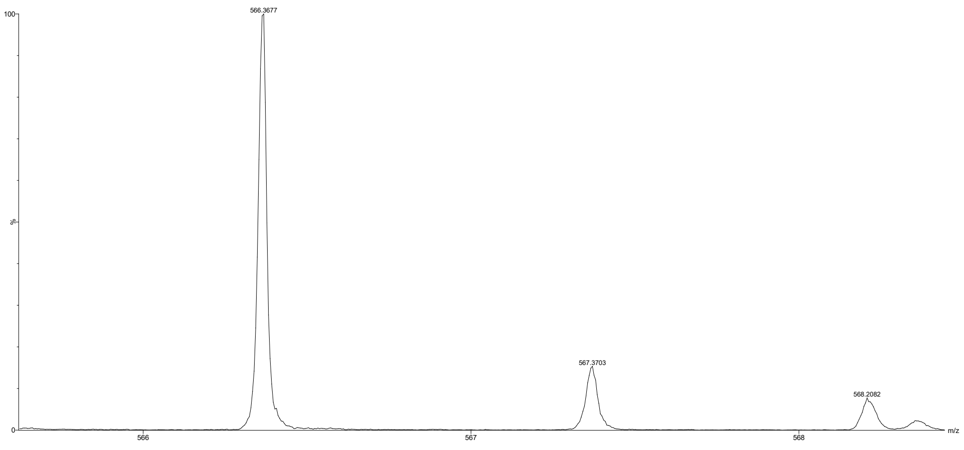

figure 42 HR-ESI-MS data of Compound 13

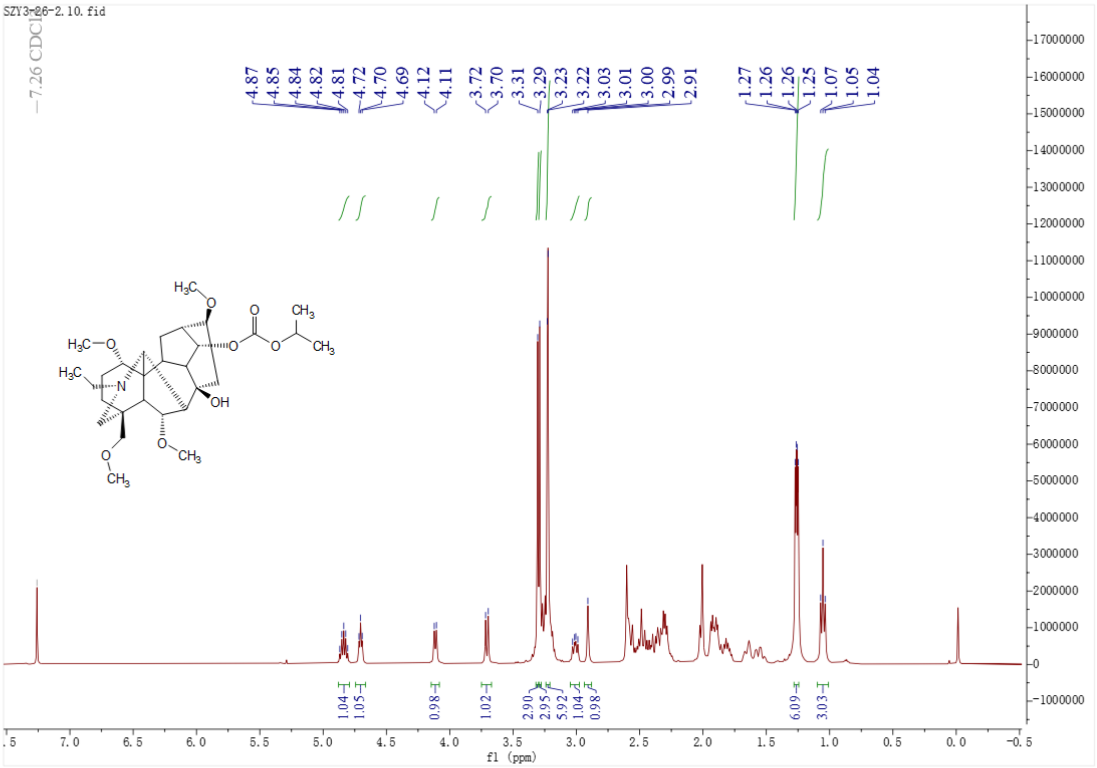

figure 43 ^{1}H NMR (400 MHz) spectrum of compound 14 in $CDCl_3$

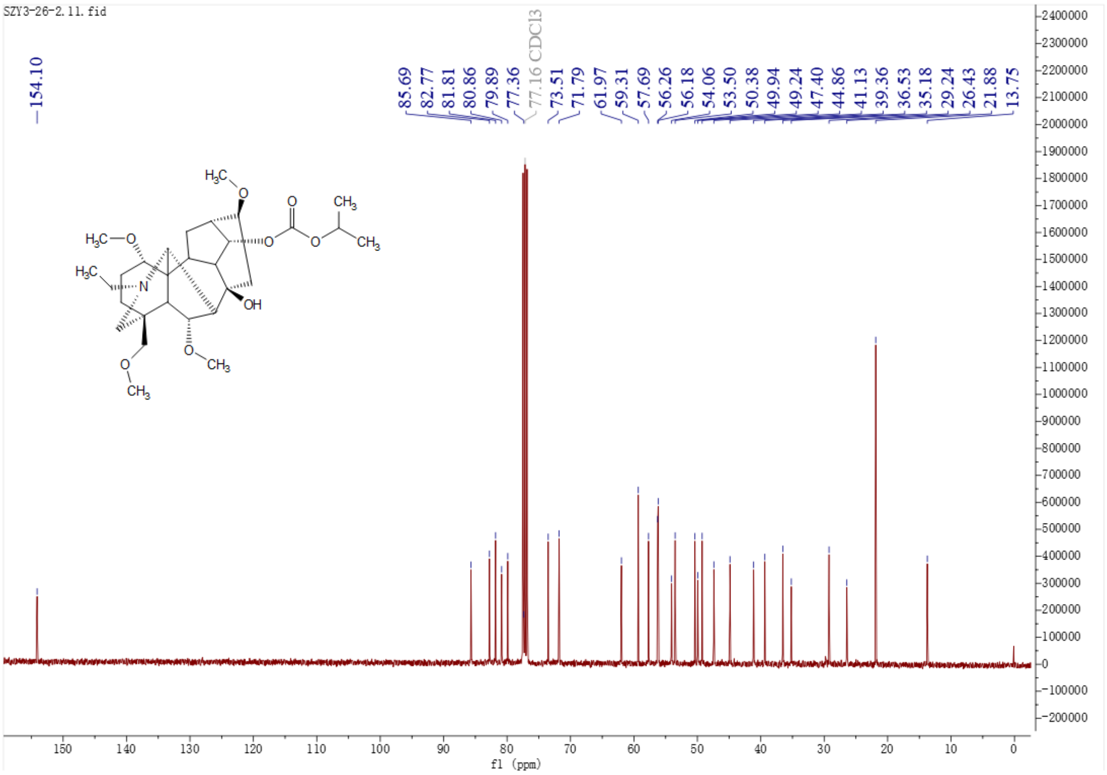

figure 44 ^{13}C NMR (100 MHz) spectrum of compound 14 in $CDCl_3$

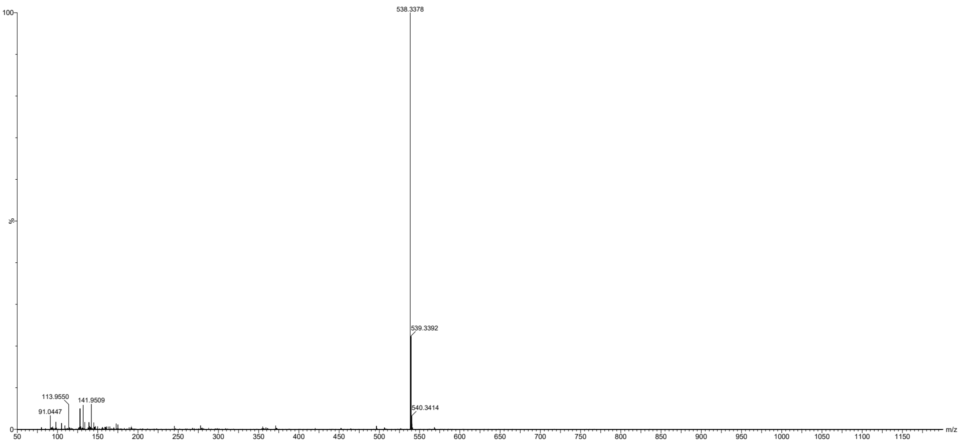

figure 45 HR-ESI-MS data of Compound 14

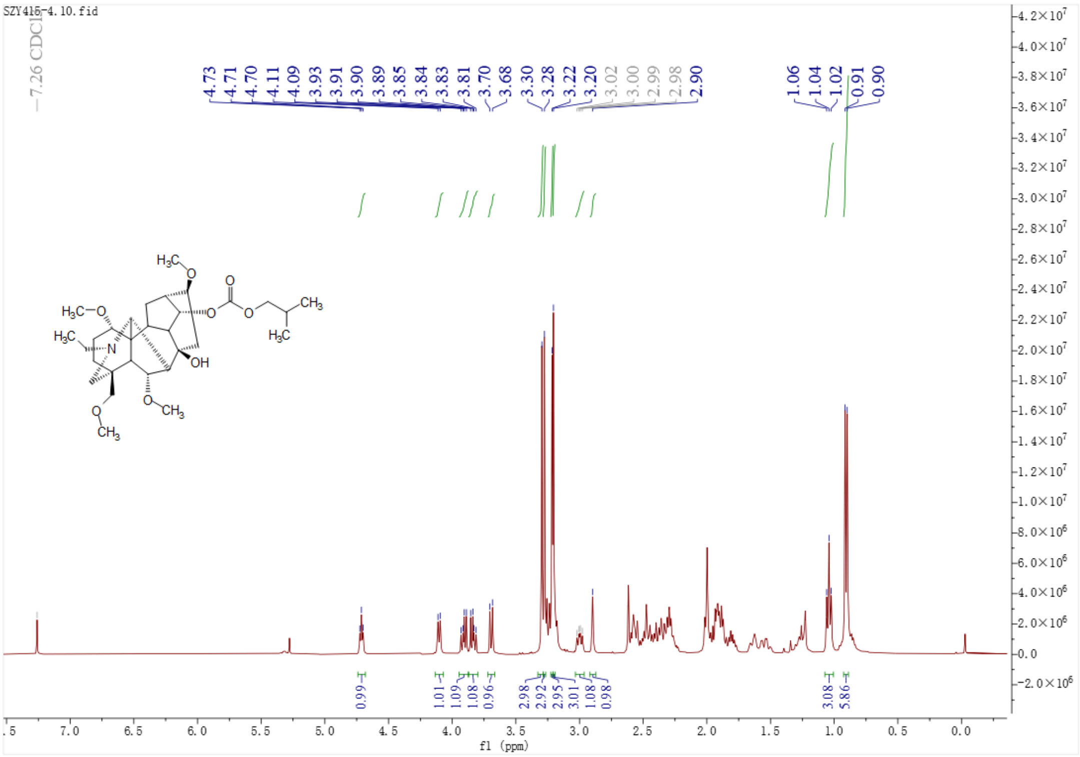

figure 46 ^{1}H NMR (400 MHz) spectrum of compound 15 in $CDCl_3$

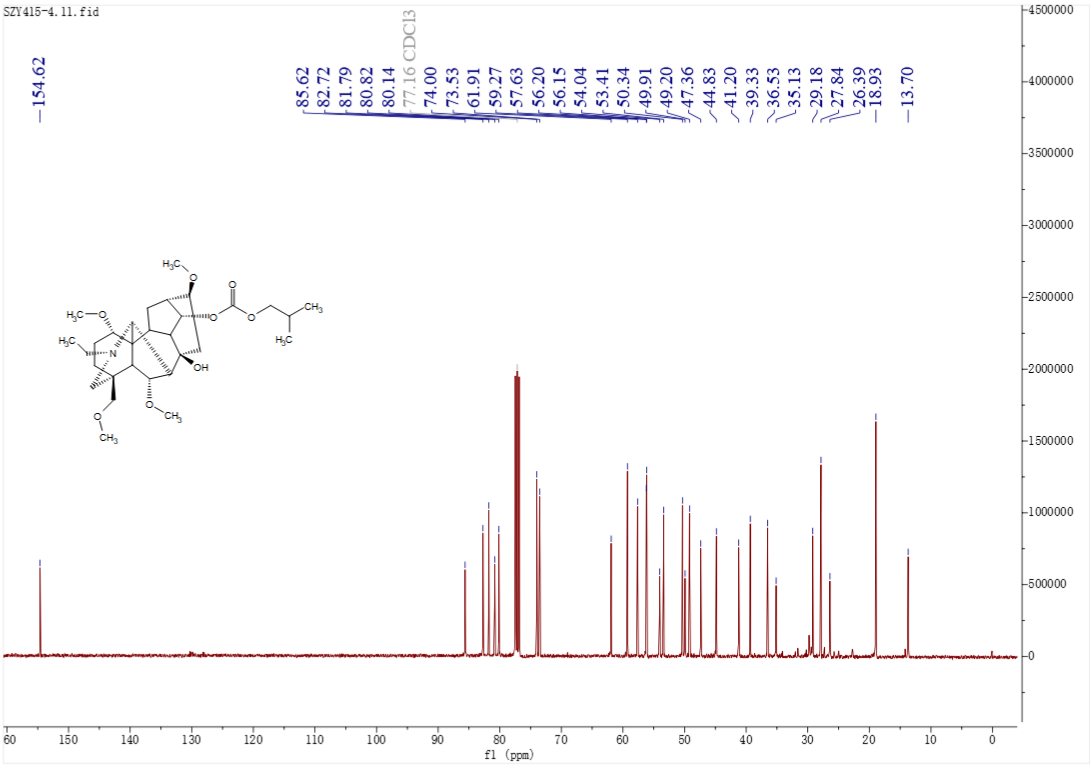

figure 47 ^{13}C NMR (100 MHz) spectrum of compound 15 in $CDCl_3$

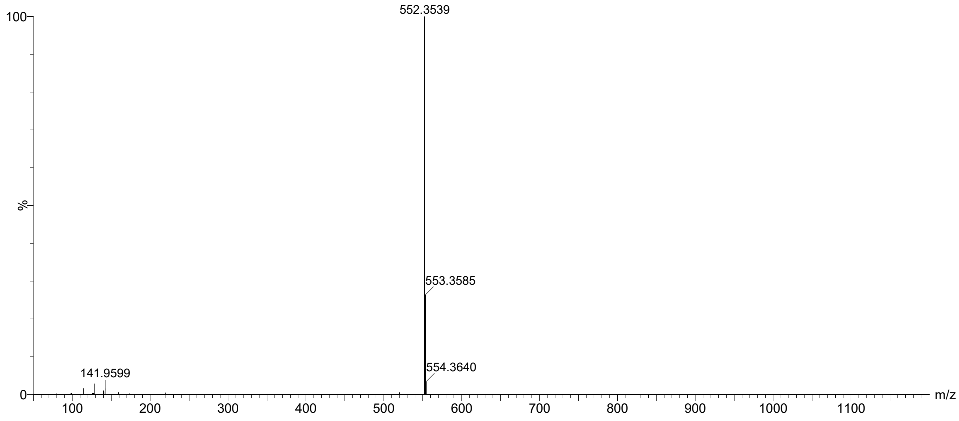

figure 48 HR-ESI-MS data of Compound 15

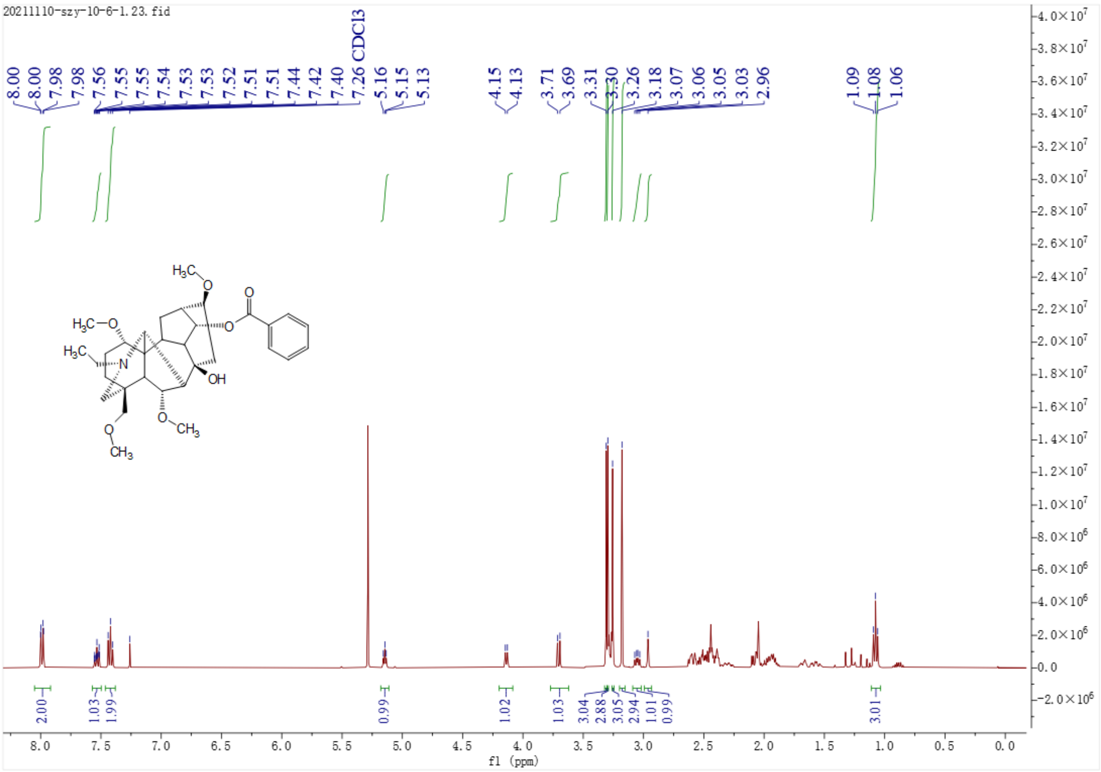

figure 49 ^{1}H NMR (400 MHz) spectrum of compound 16 in $CDCl_3$

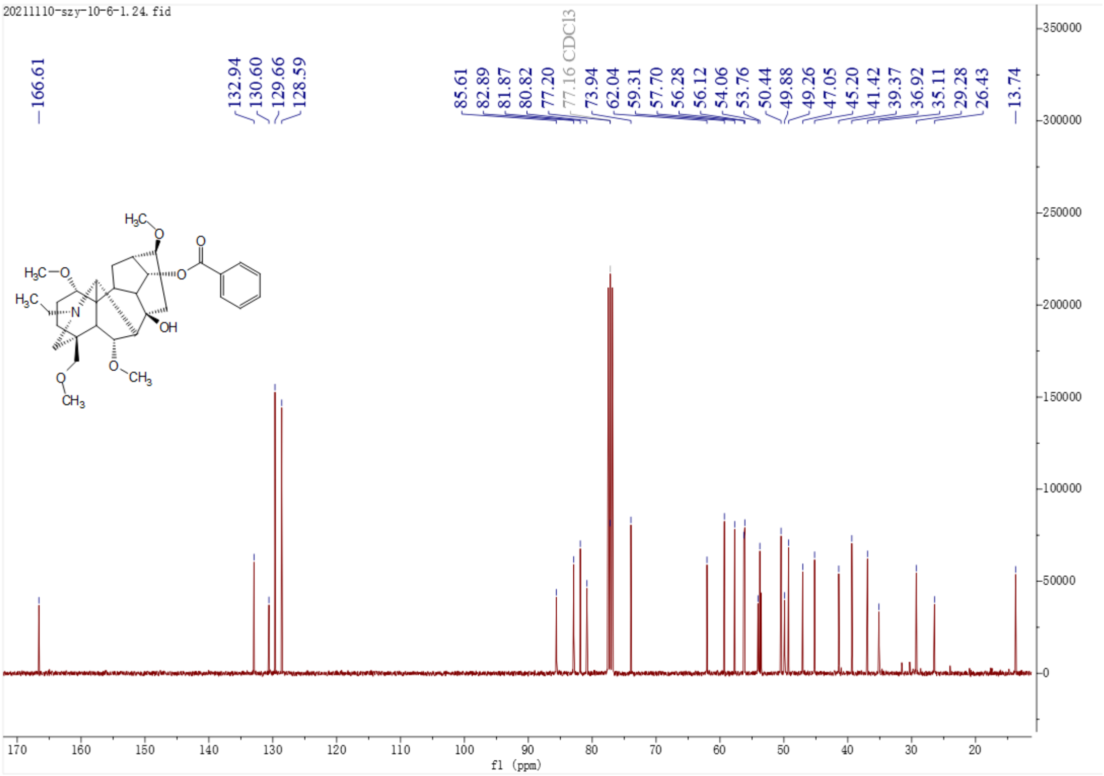

figure 50 ^{13}C NMR (100 MHz) spectrum of compound 16 in $CDCl_3$

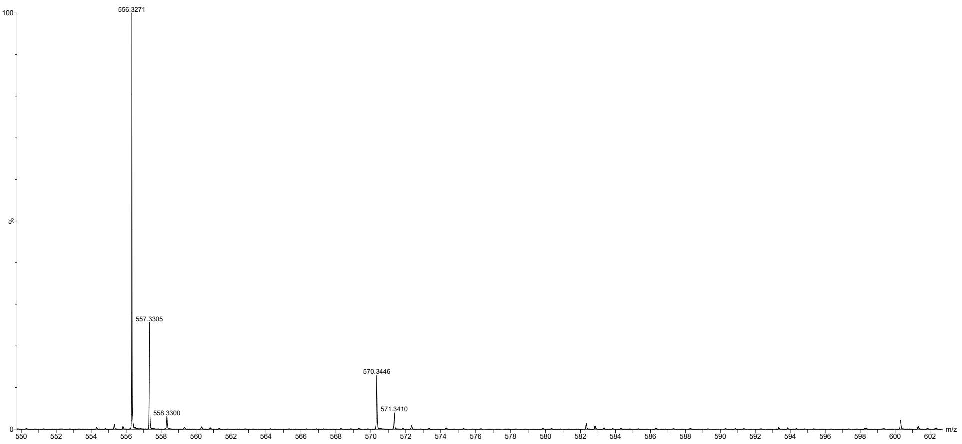

figure 51 HR-ESI-MS data of Compound 16

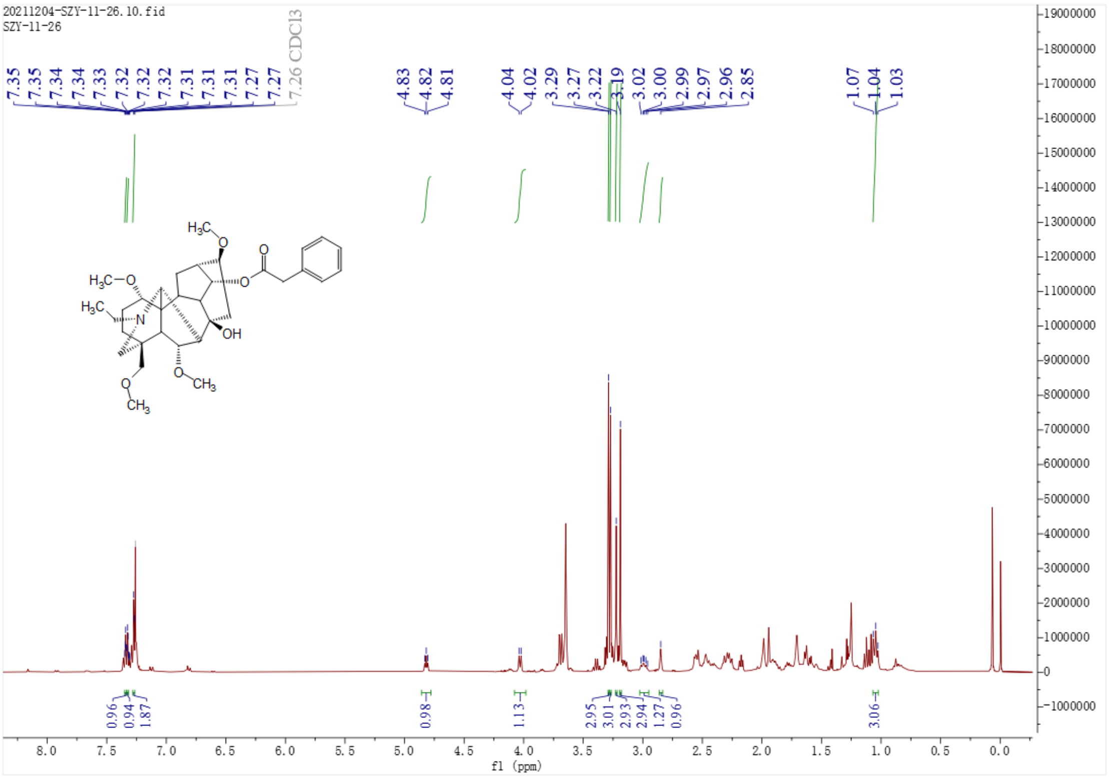

figure 52 ^{1}H NMR (400 MHz) spectrum of compound 17 in $CDCl_3$

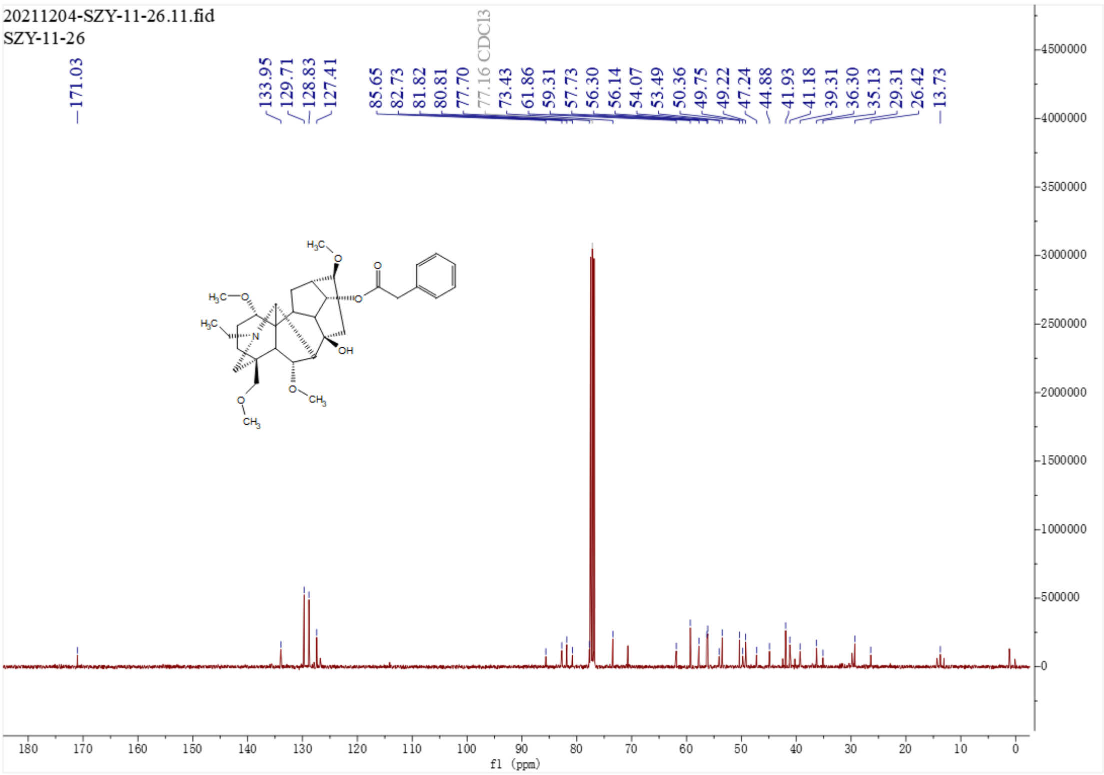

figure 53 ^{13}C NMR (100 MHz) spectrum of compound 17 in $CDCl_3$

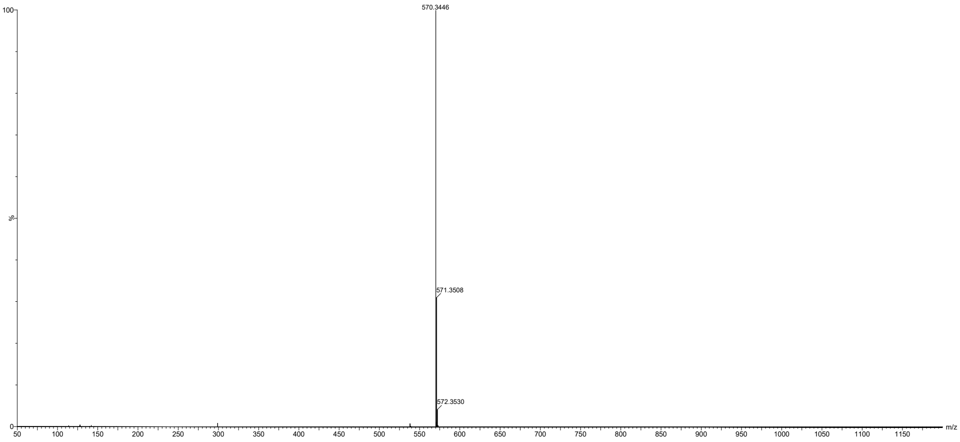

figure 54 HR-ESI-MS data of Compound 17

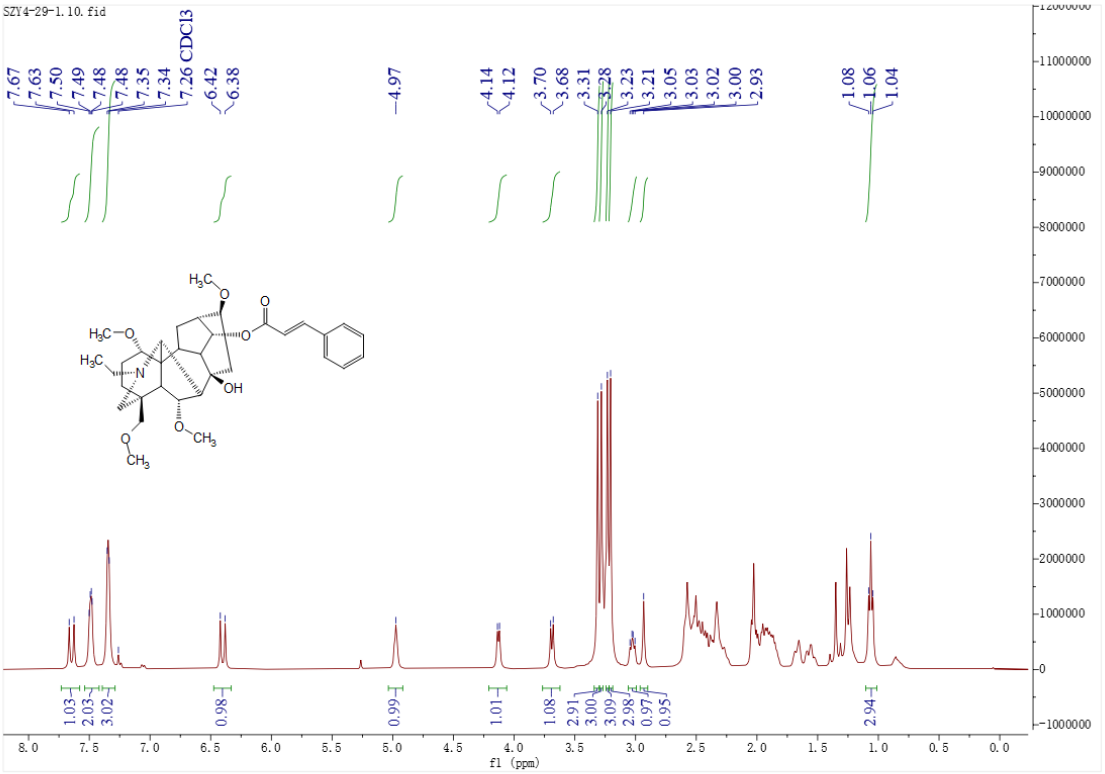

figure 55 ^{1}H NMR (400 MHz) spectrum of compound 18 in $CDCl_3$

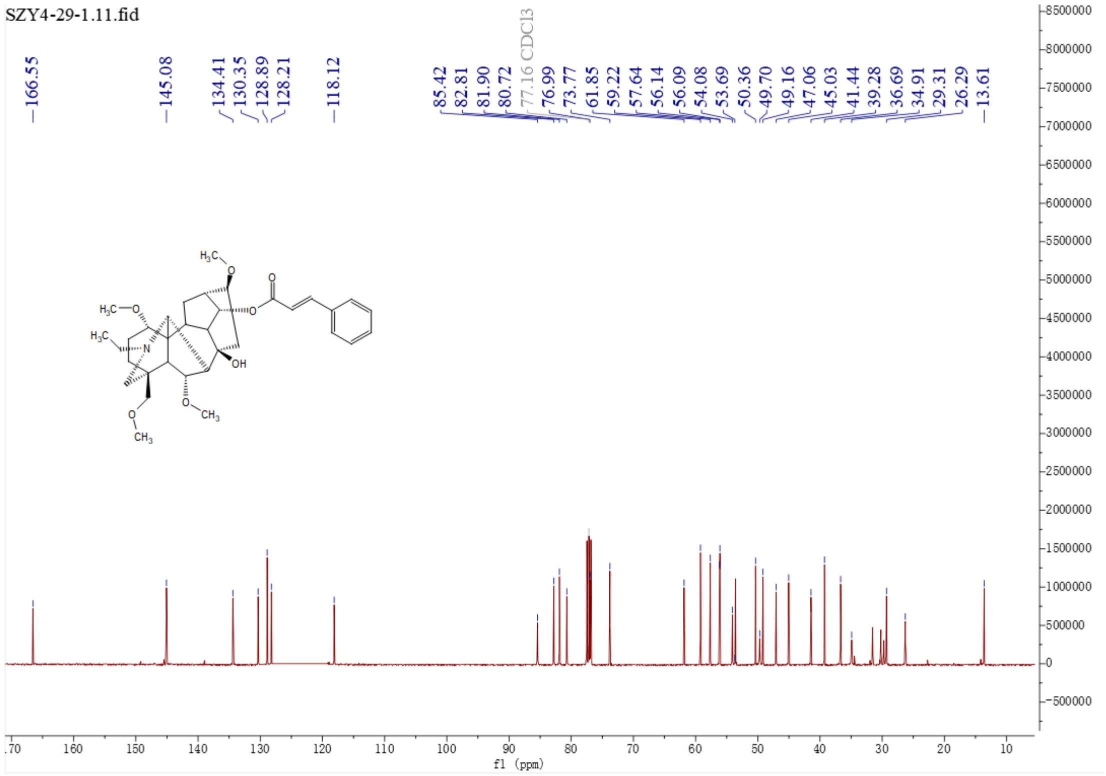

figure 56 ^{13}C NMR (100 MHz) spectrum of compound 18 in $CDCl_3$

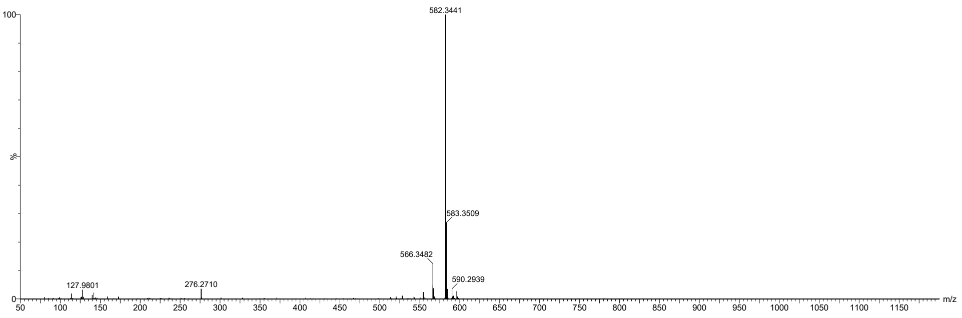

figure 57 HR-ESI-MS data of Compound 18

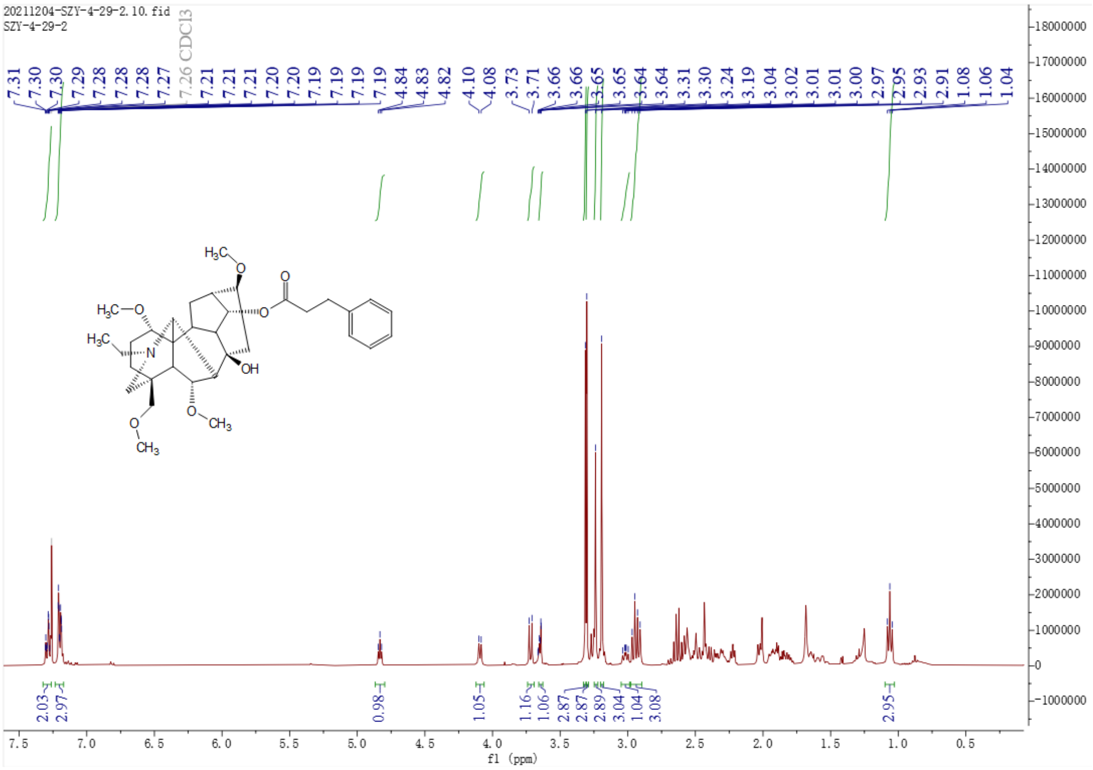

figure 58 ^{1}H NMR (400 MHz) spectrum of compound 19 in $CDCl_3$

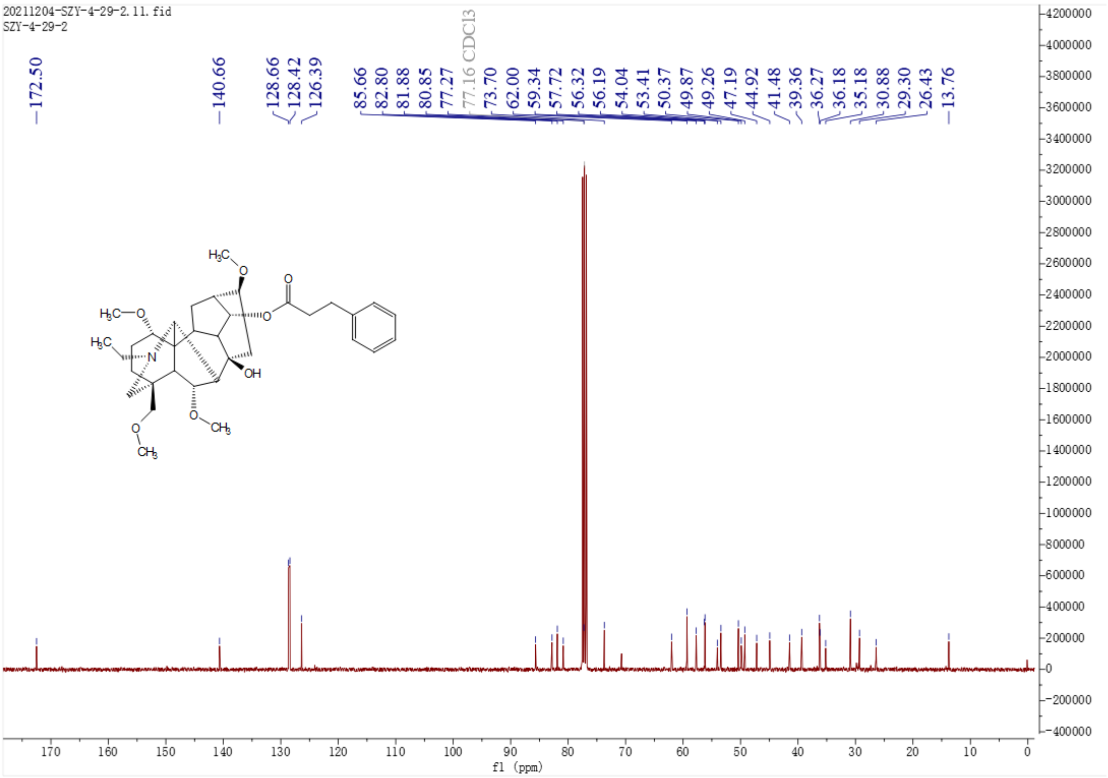

figure 59 ^{13}C NMR (100 MHz) spectrum of compound 19 in $CDCl_3$

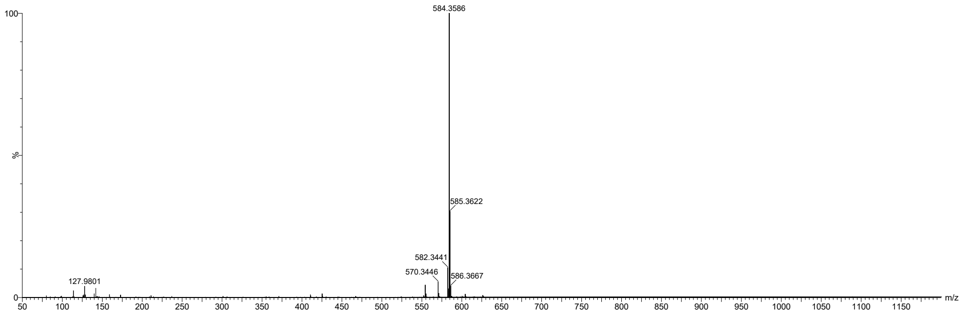

figure 60 HR-ESI-MS data of Compound 19

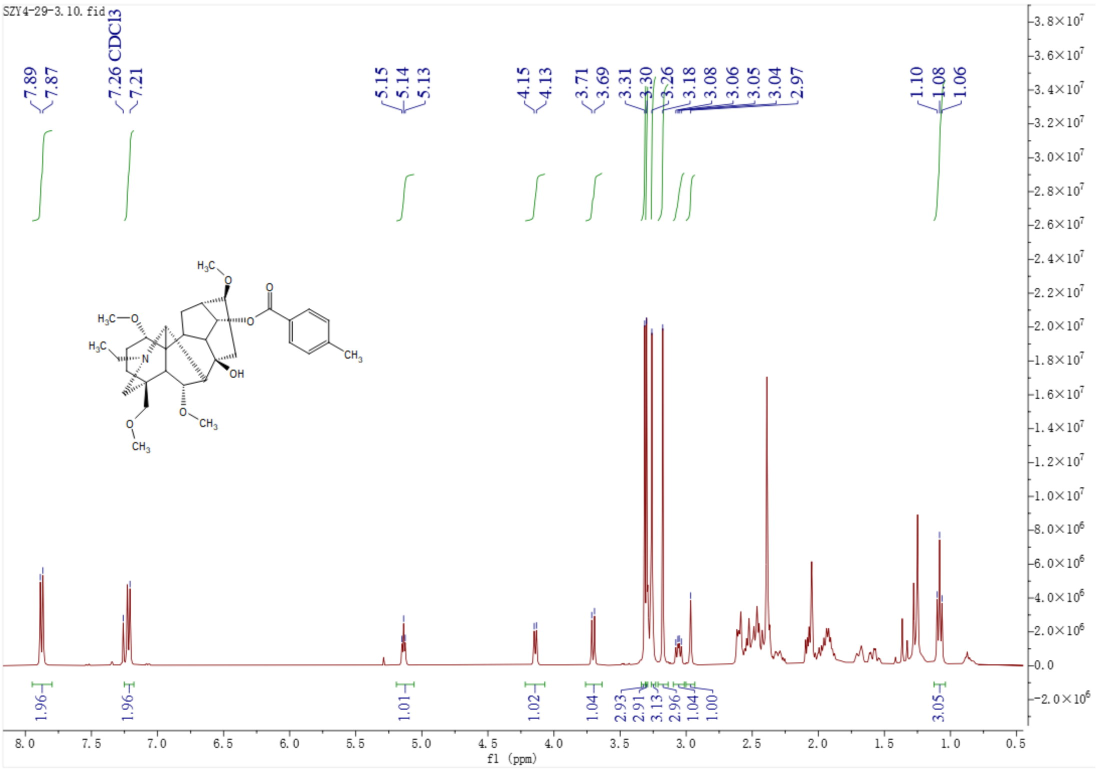

figure 61 ^{1}H NMR (400 MHz) spectrum of compound 20 in $CDCl_3$

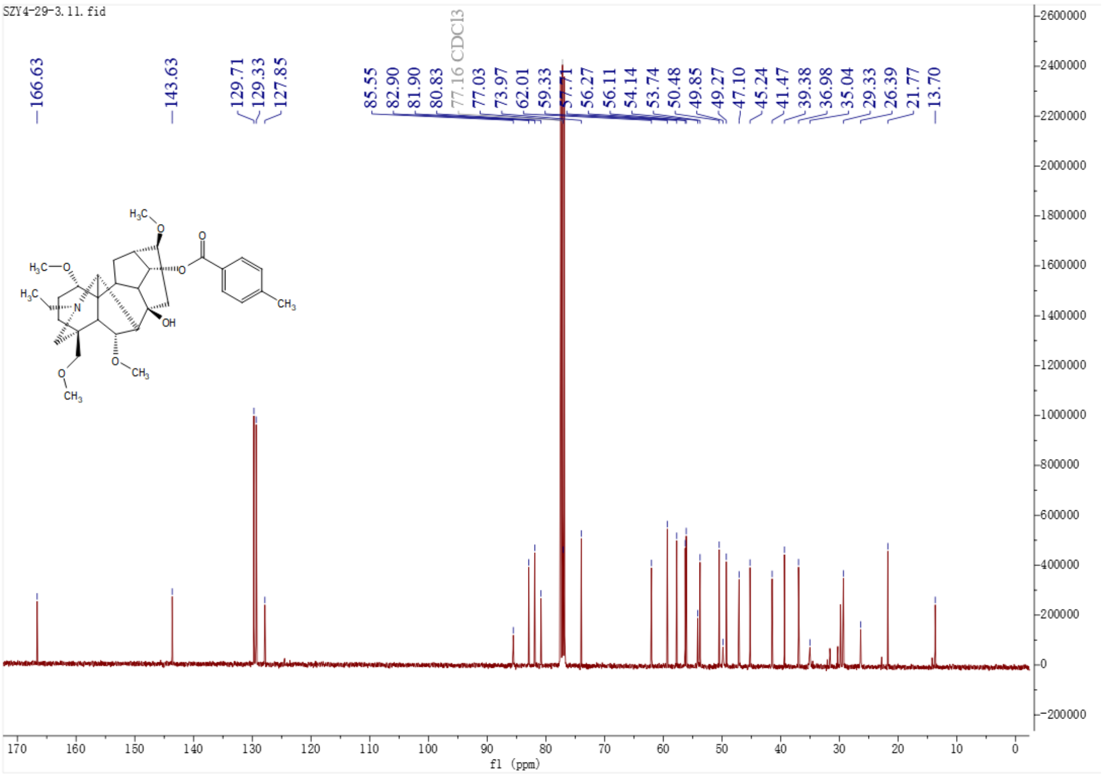

figure 62 ^{13}C NMR (100 MHz) spectrum of compound 20 in $CDCl_3$

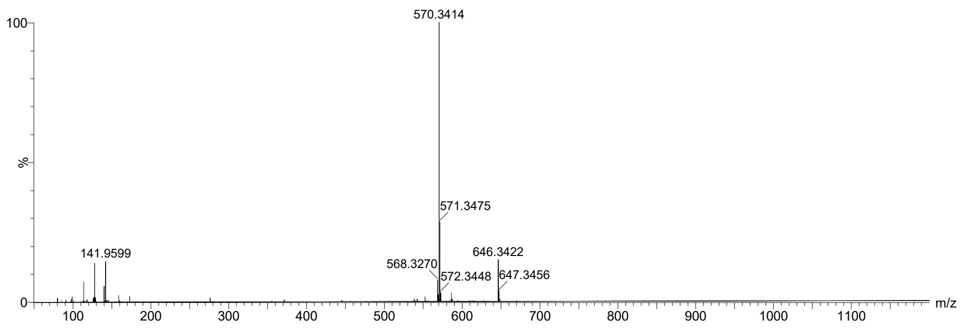

figure 63 HR-ESI-MS data of Compound 20

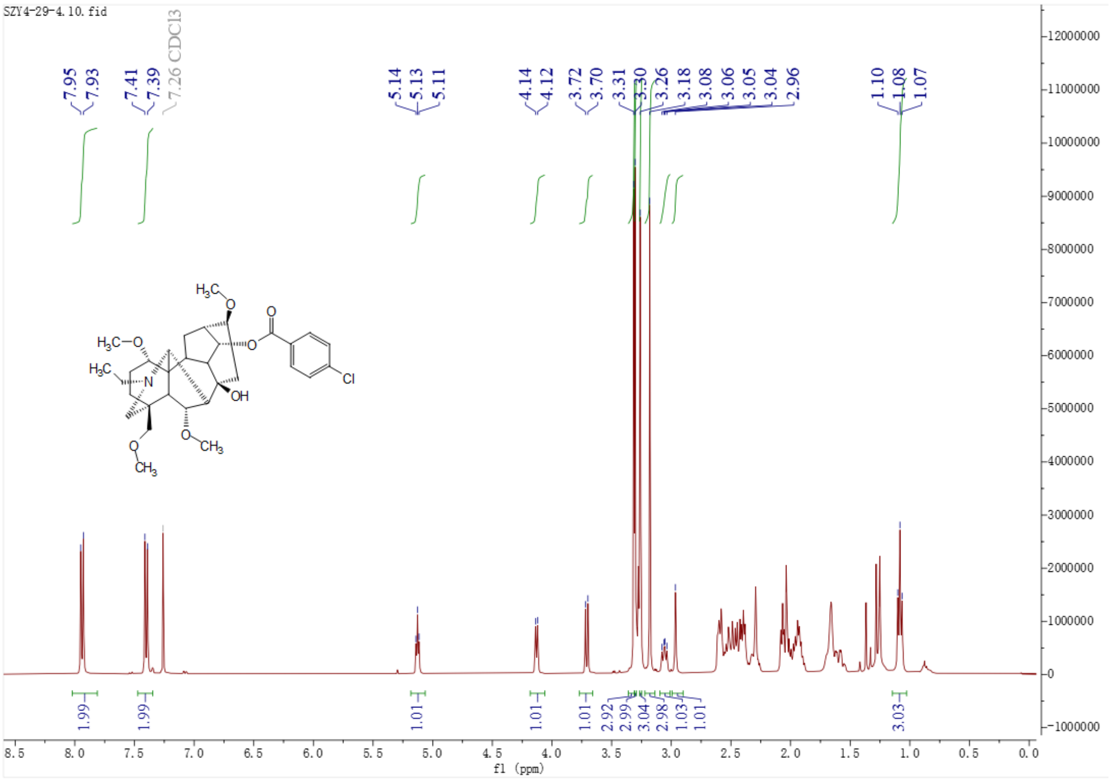

figure 64 ^{1}H NMR (400 MHz) spectrum of compound 21 in $CDCl_3$

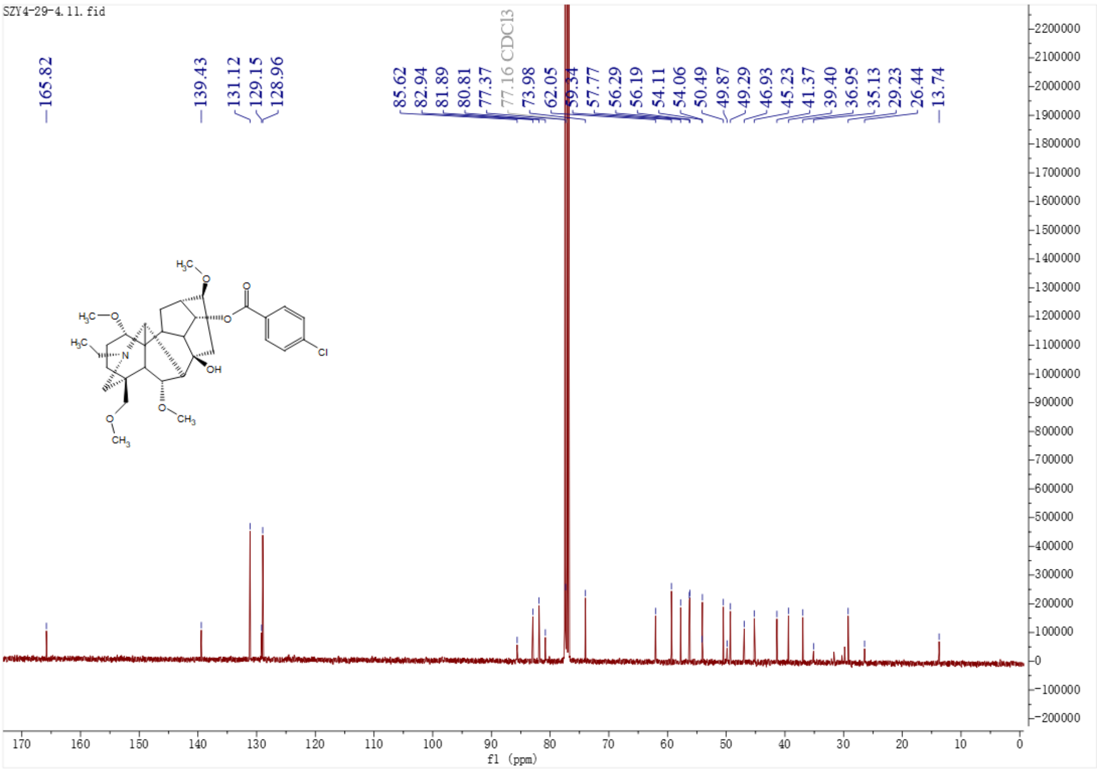

figure 65 ^{13}C NMR (100 MHz) spectrum of compound 21 in $CDCl_3$

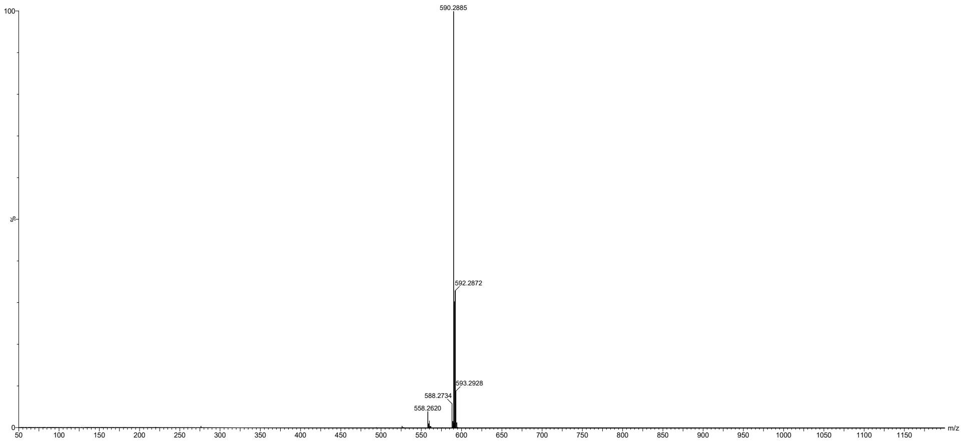

figure 66 HR-ESI-MS data of Compound 21

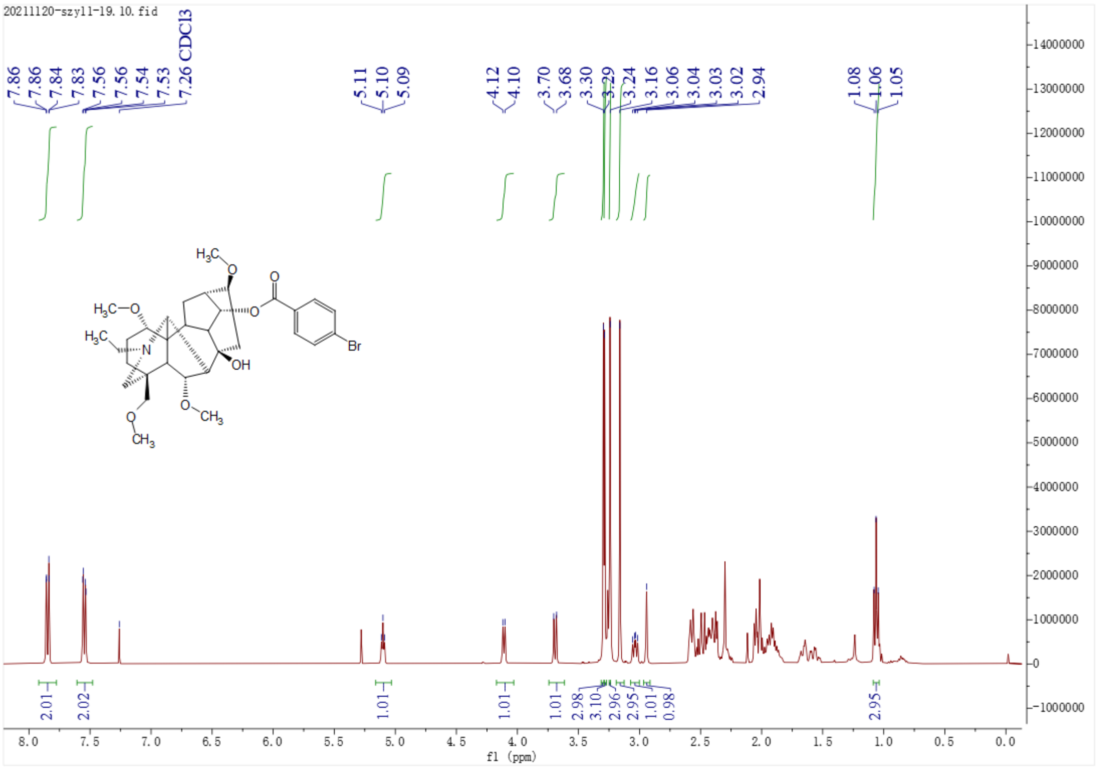

figure 67 ^{1}H NMR (400 MHz) spectrum of compound 22 in $CDCl_3$

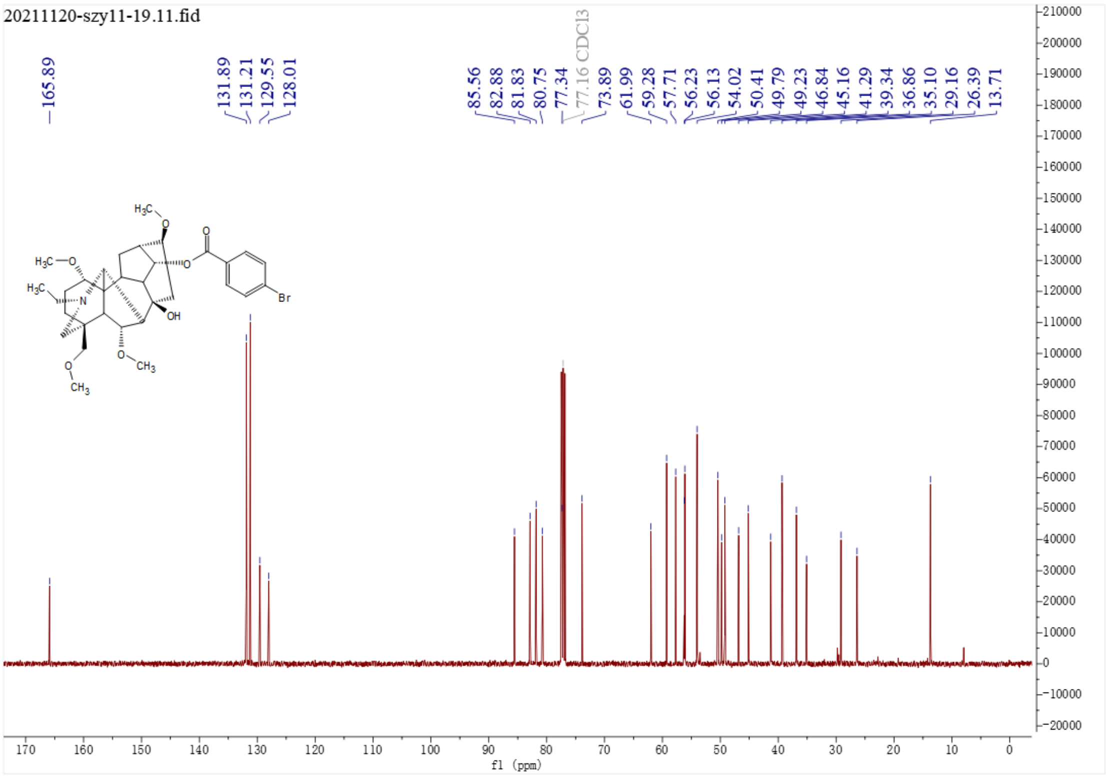

figure 68 ^{13}C NMR (100 MHz) spectrum of compound 22 in $CDCl_3$

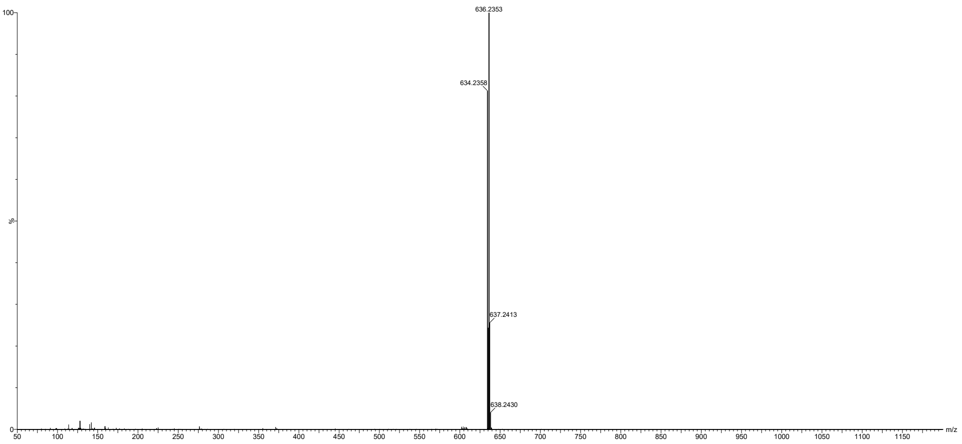

figure 69 HR-ESI-MS data of Compound 22

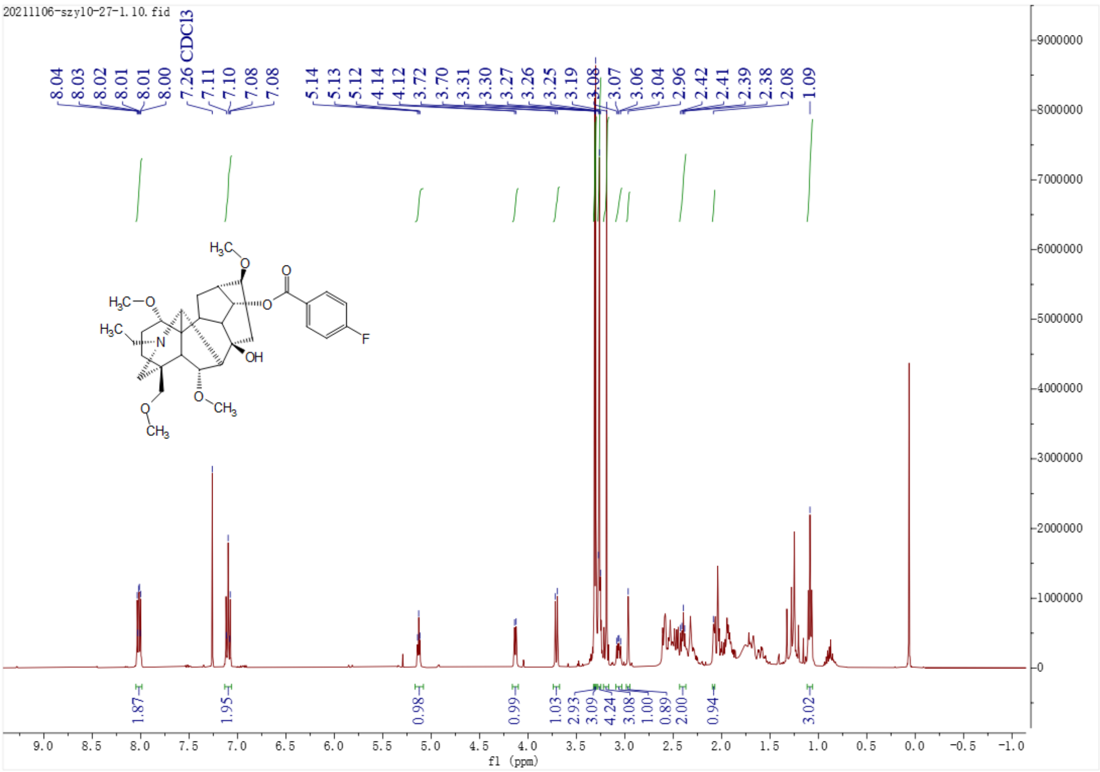

figure 70 ^{1}H NMR (400 MHz) spectrum of compound 23 in $CDCl_3$

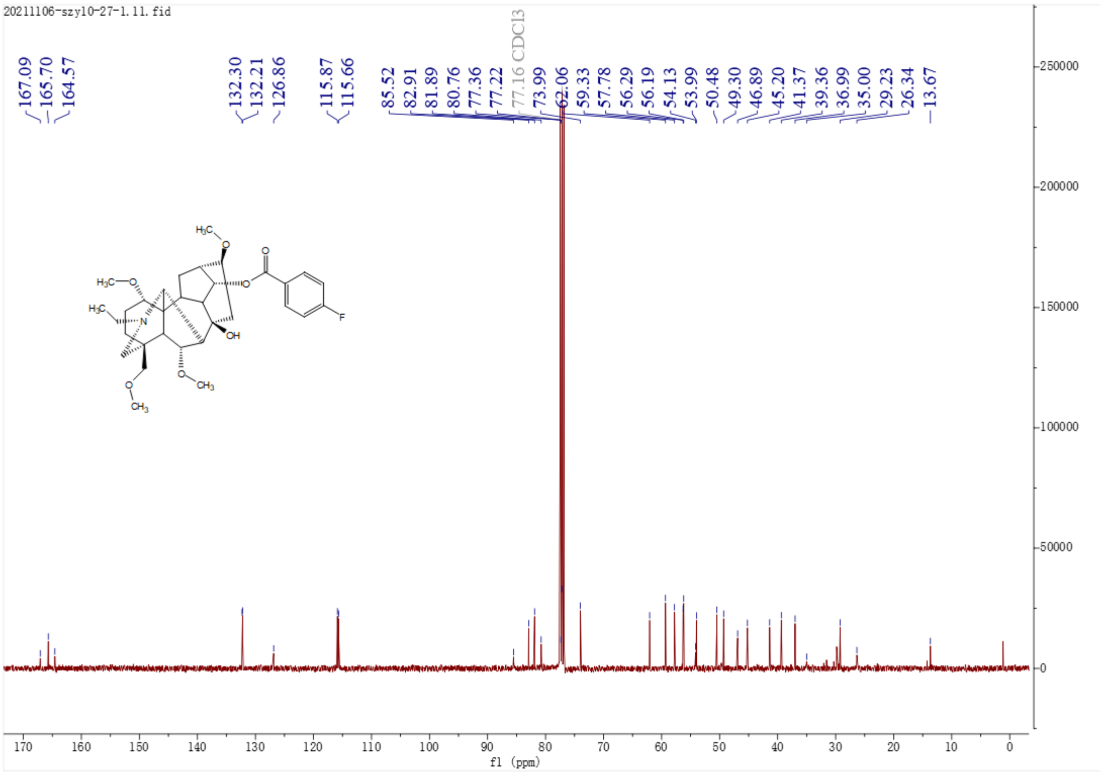

figure 71 ^{13}C NMR (100 MHz) spectrum of compound 23 in $CDCl_3$

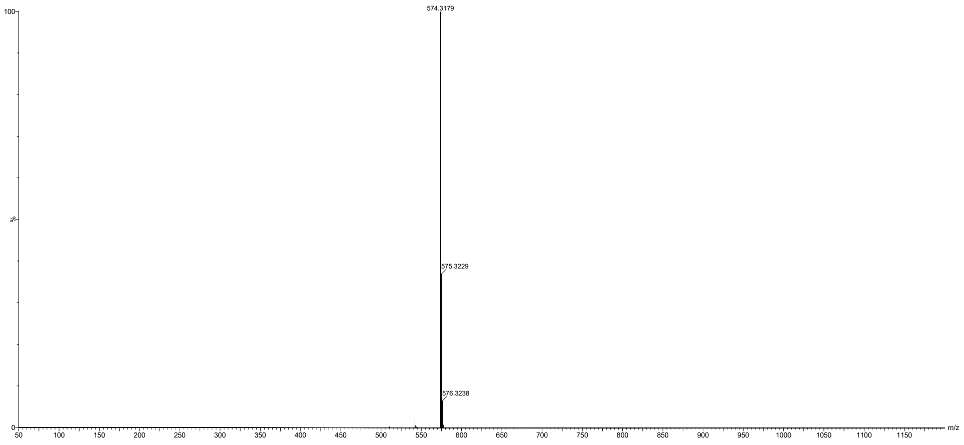

figure 72 HR-ESI-MS data of Compound 23

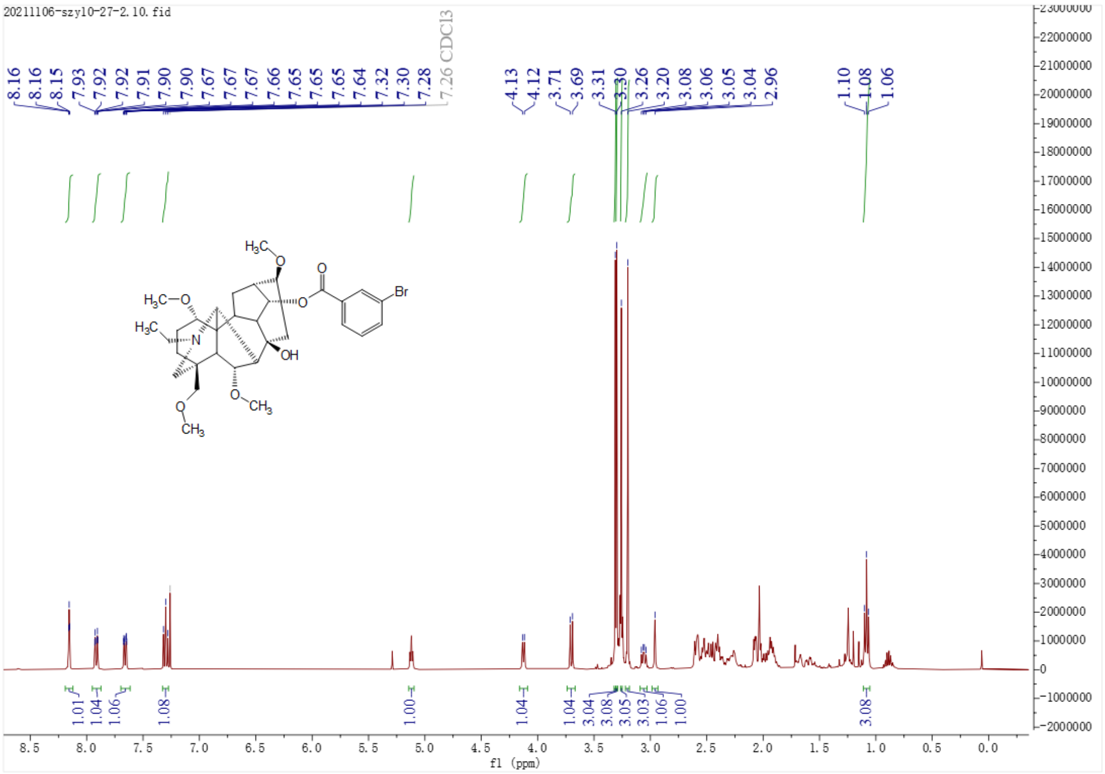

figure 73 ^{1}H NMR (400 MHz) spectrum of compound 24 in $CDCl_3$

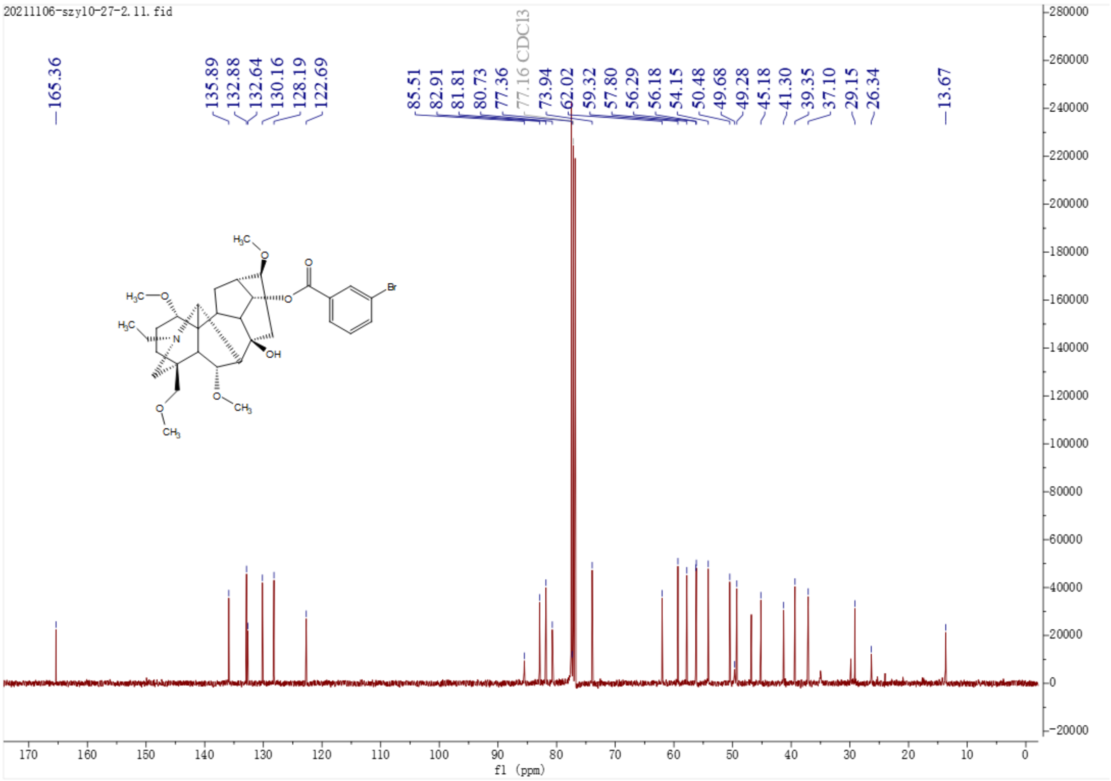

figure 74 ^{13}C NMR (100 MHz) spectrum of compound 24 in $CDCl_3$

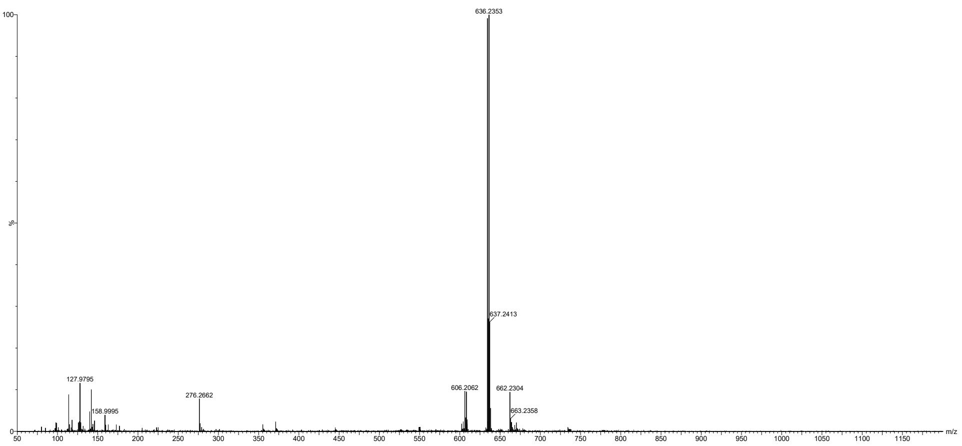

figure 75 HR-ESI-MS data of Compound 24

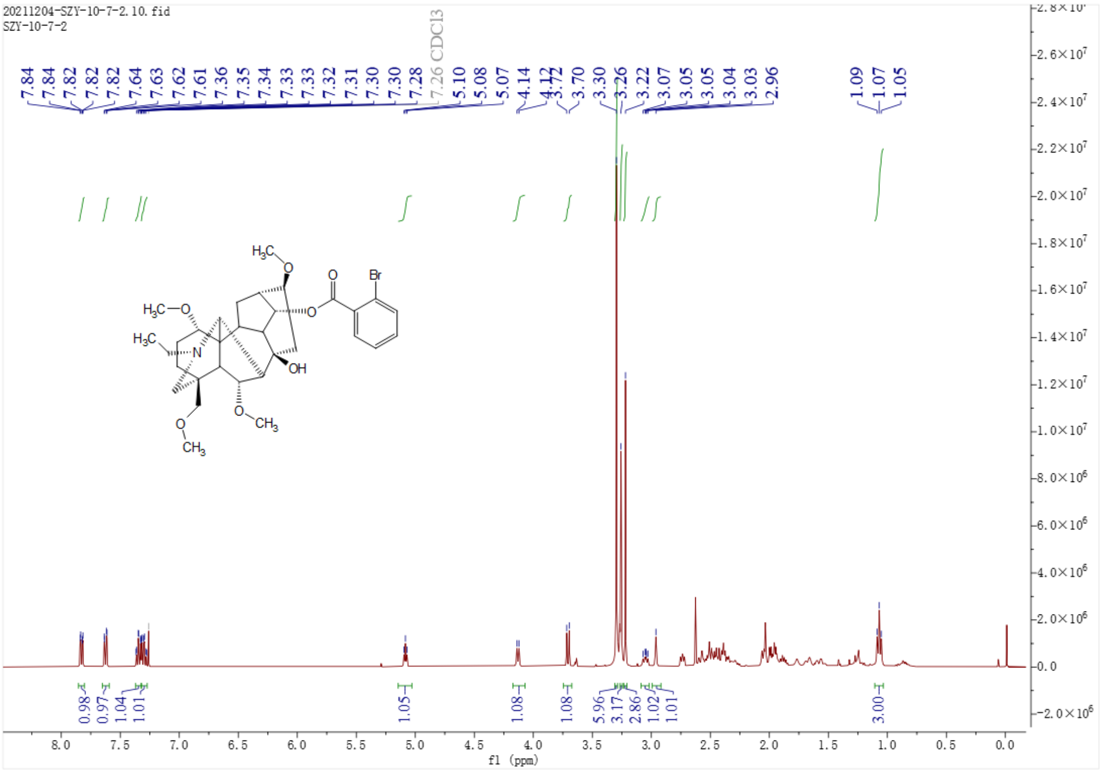

figure 76 ^{1}H NMR (400 MHz) spectrum of compound 25 in $CDCl_3$

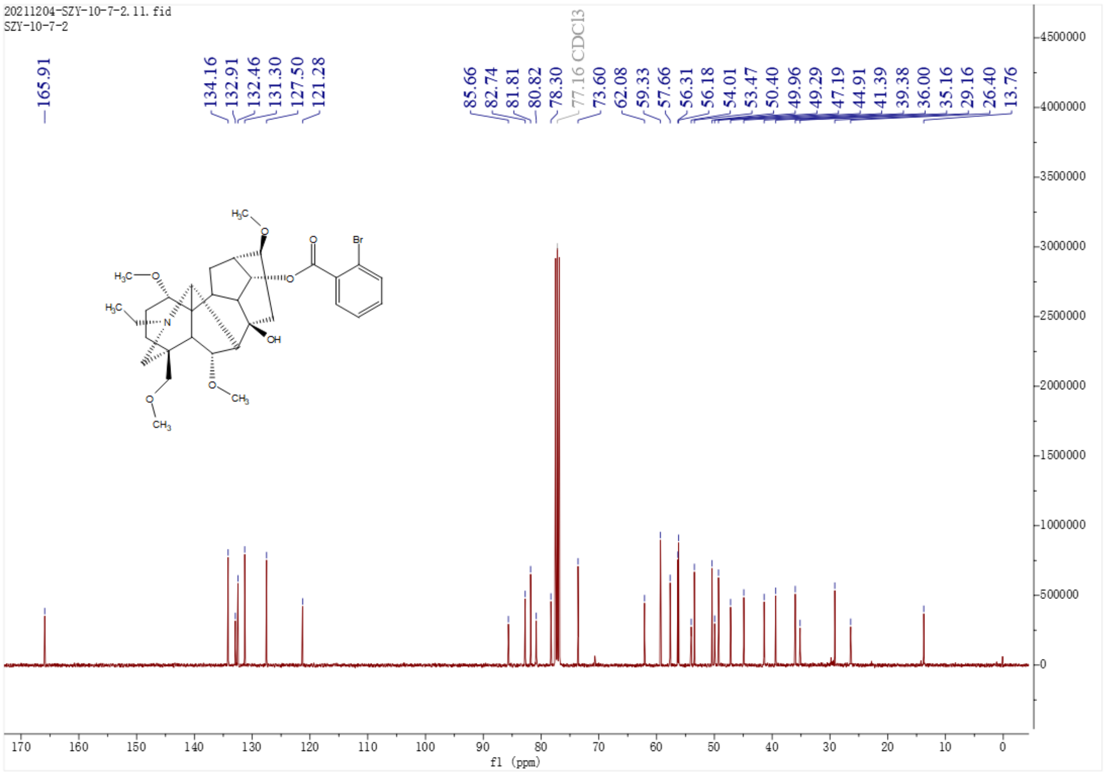

figure 77 ^{13}C NMR (100 MHz) spectrum of compound 25 in $CDCl_3$

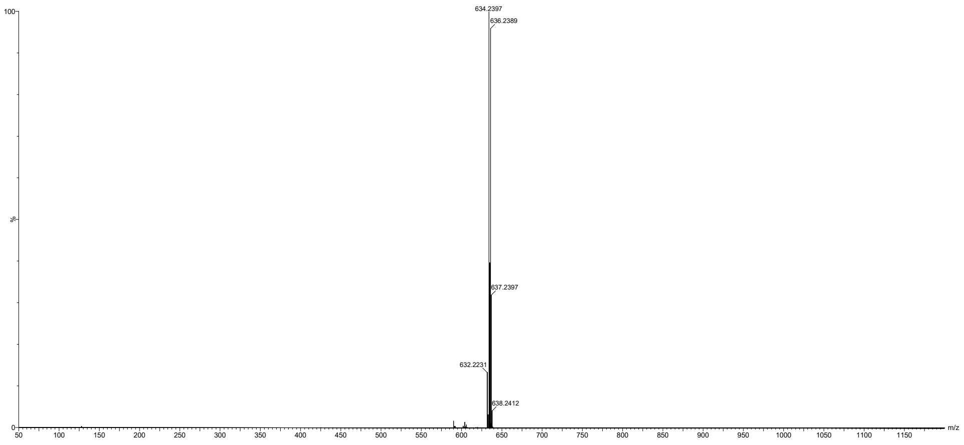

figure 78 HR-ESI-MS data of Compound 25

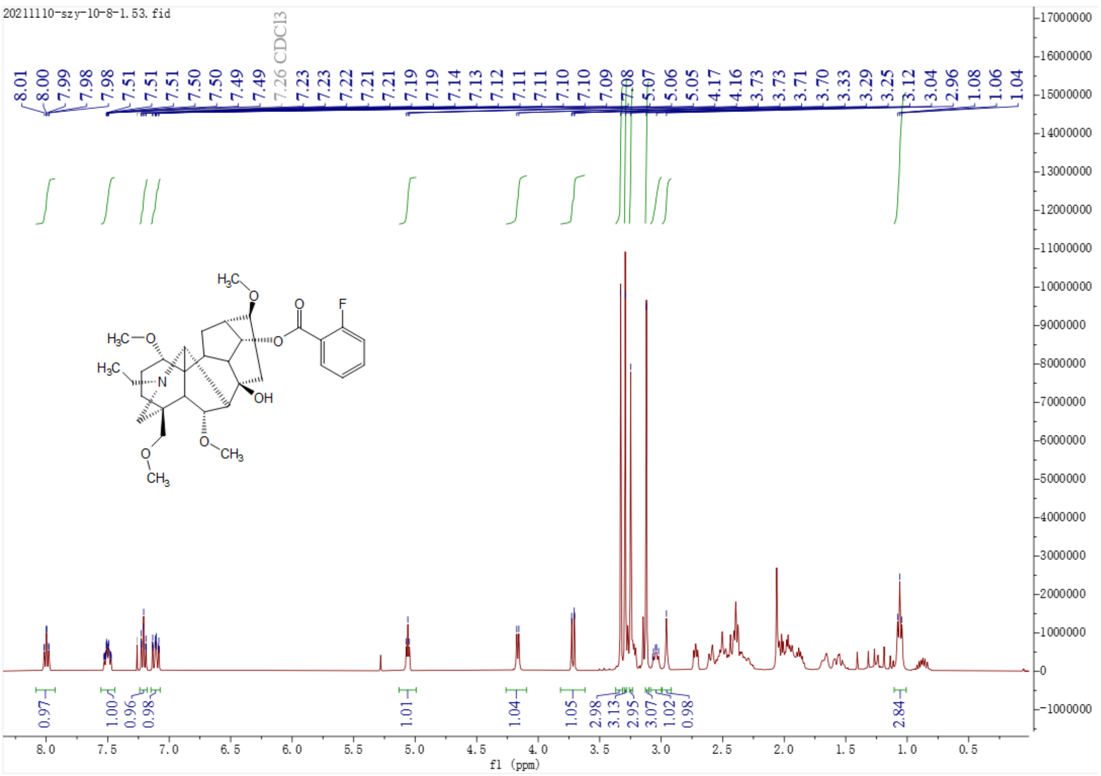

figure 79 ^{1}H NMR (400 MHz) spectrum of compound 26 in $CDCl_3$

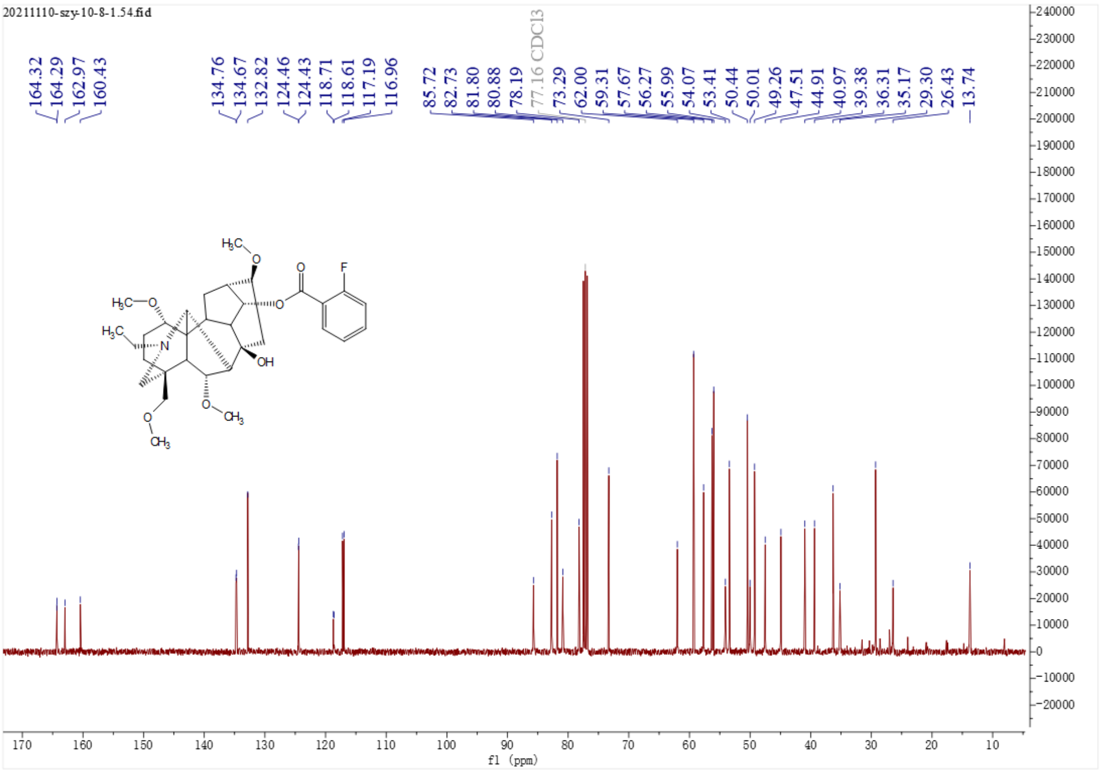

figure 80 ^{13}C NMR (100 MHz) spectrum of compound 26 in $CDCl_3$

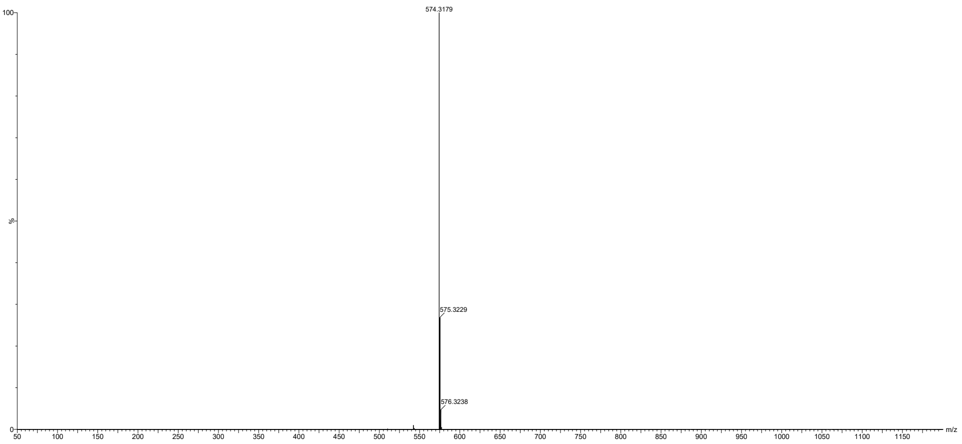

figure 81 HR-ESI-MS data of Compound 26

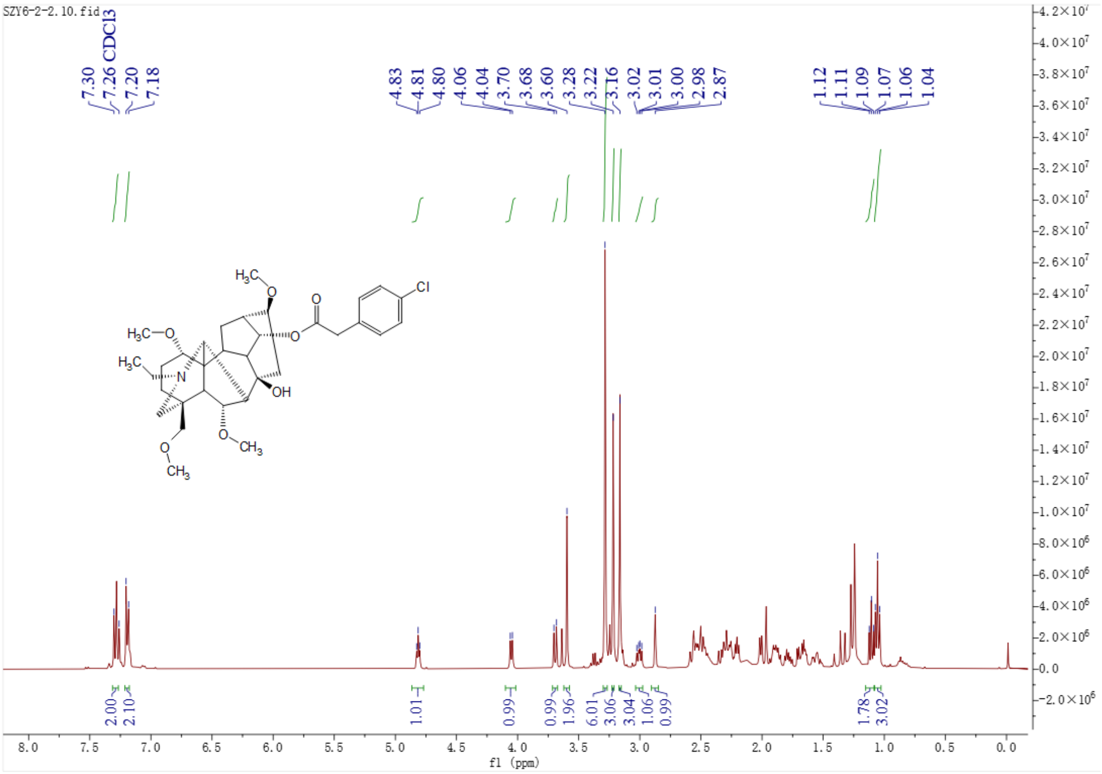

figure 82 ^{1}H NMR (400 MHz) spectrum of compound 27 in $CDCl_3$

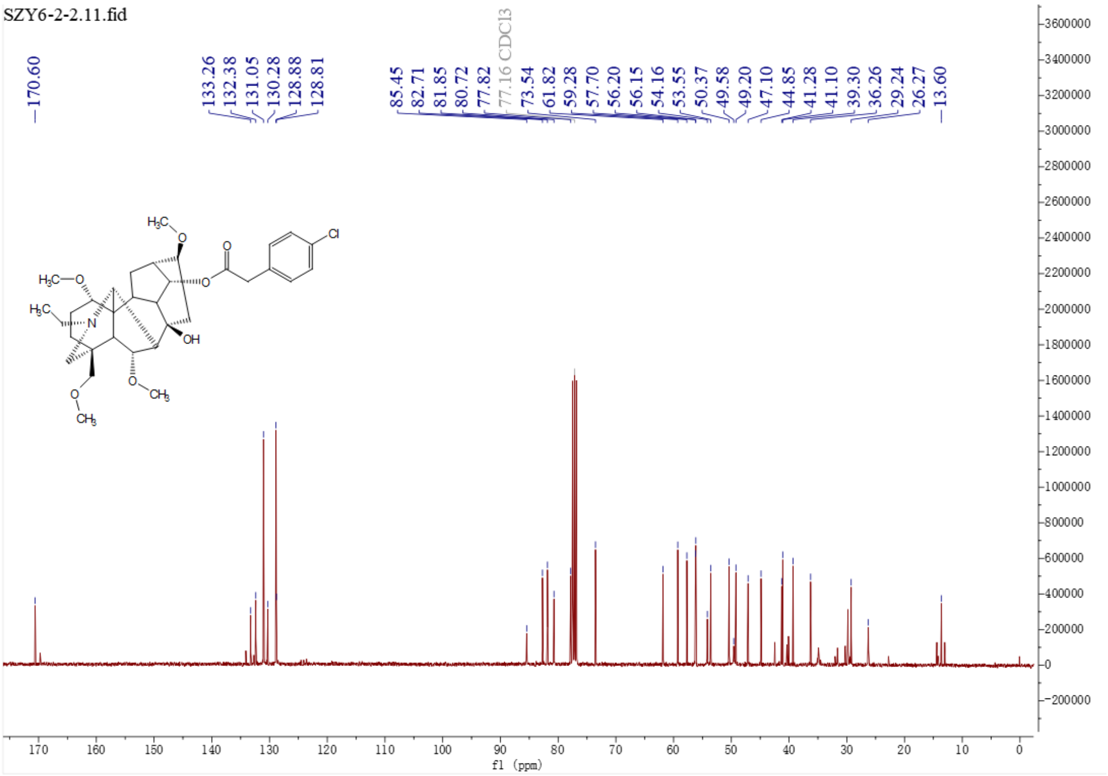

figure 83 ^{13}C NMR (100 MHz) spectrum of compound 27 in $CDCl_3$

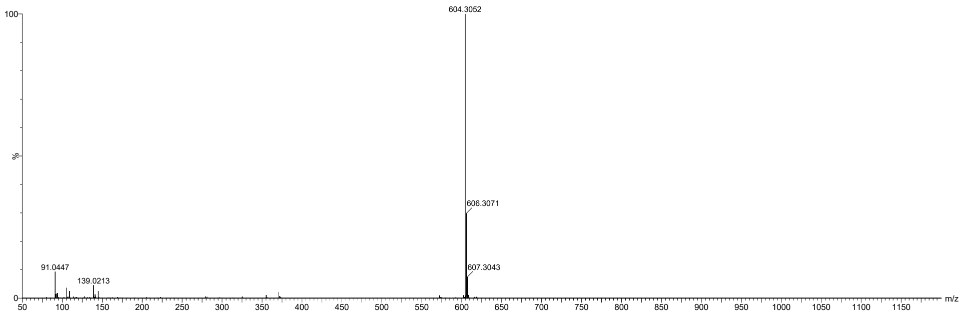

figure 84 HR-ESI-MS data of Compound 27

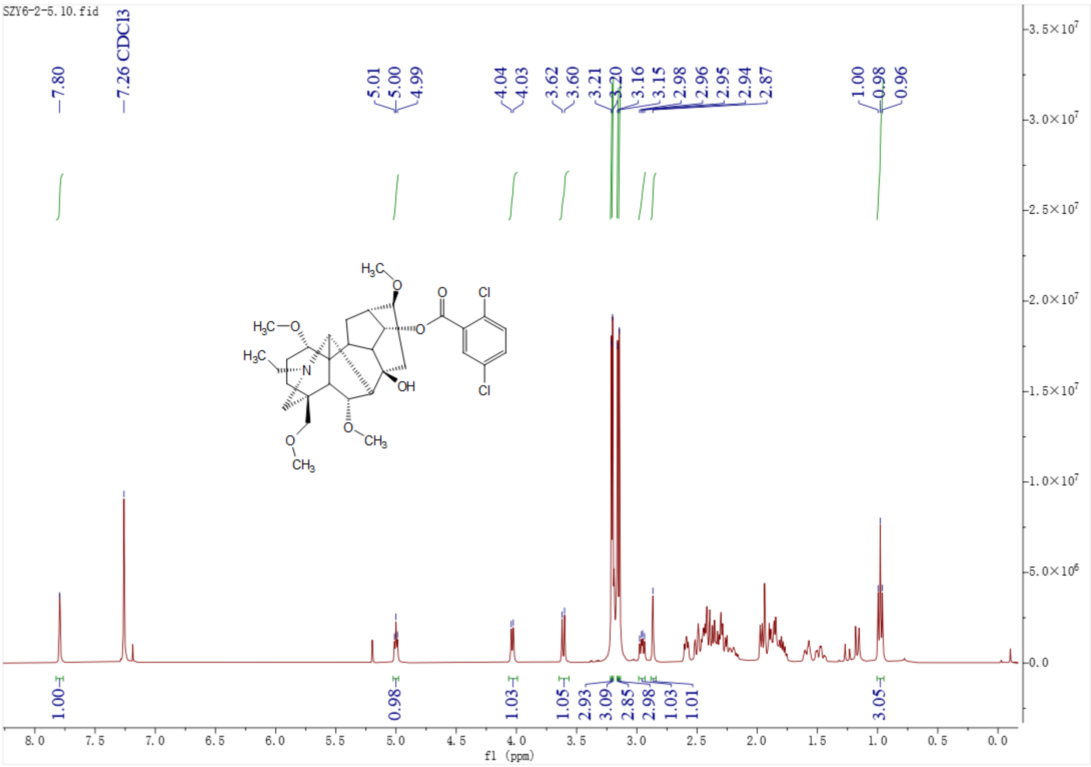

figure 85 ^{1}H NMR (400 MHz) spectrum of compound 28 in $CDCl_3$

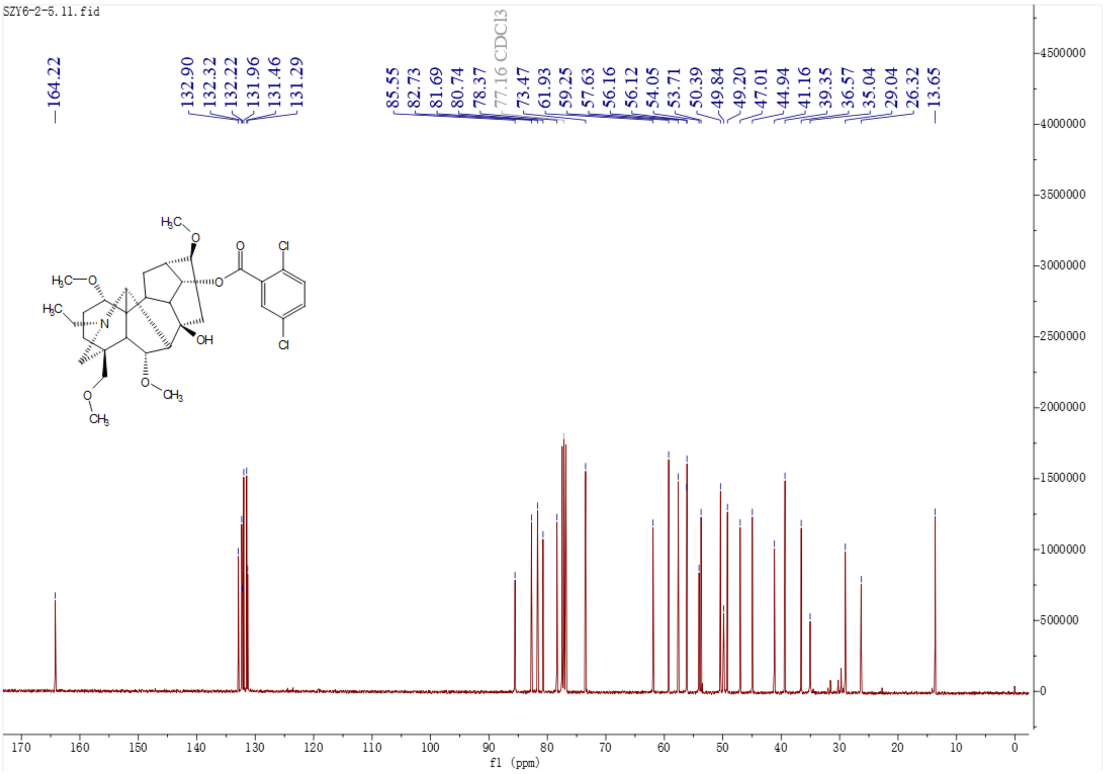

figure 86 ^{13}C NMR (100 MHz) spectrum of compound 28 in $CDCl_3$

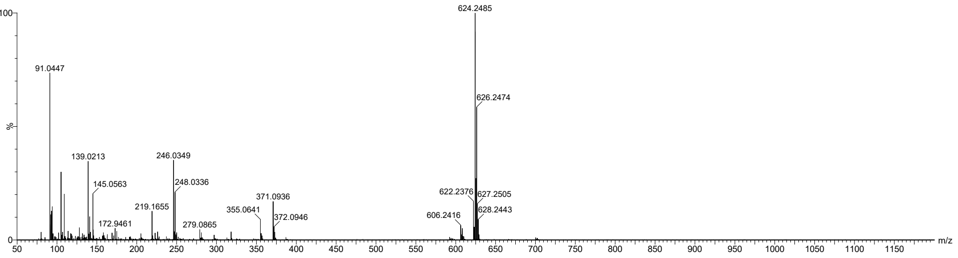

figure 87 HR-ESI-MS data of Compound 28

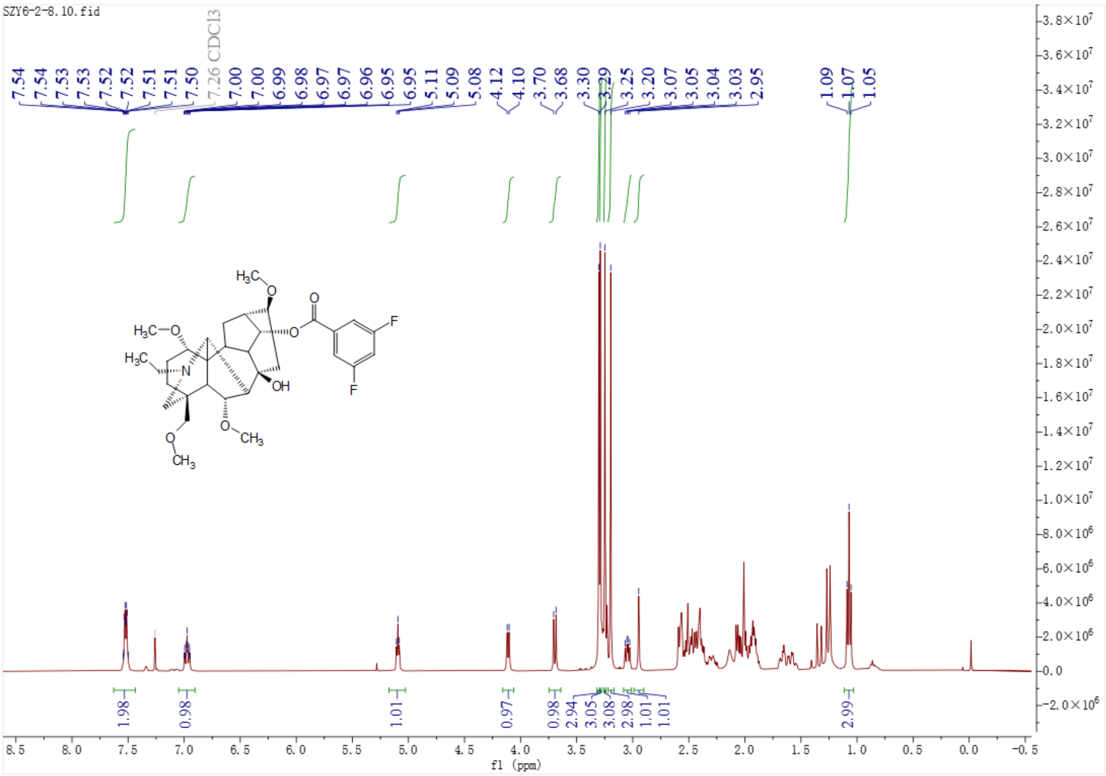

figure 88 ^{1}H NMR (400 MHz) spectrum of compound 29 in $CDCl_3$

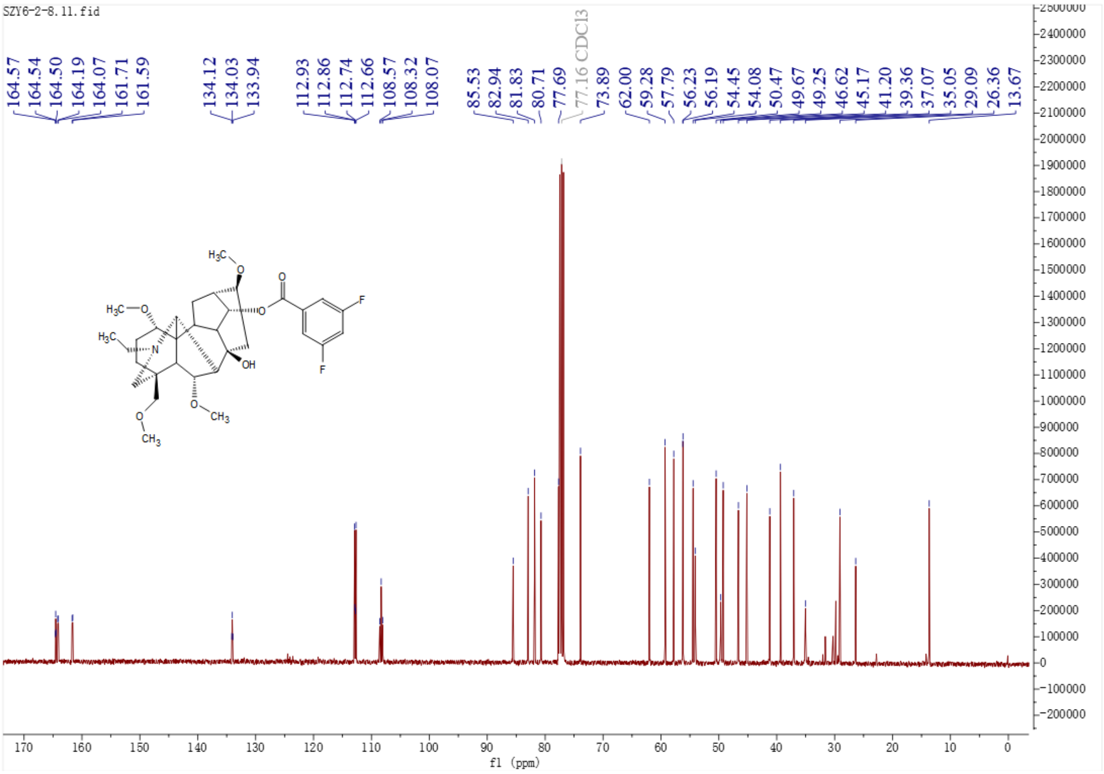

figure 89 ^{13}C NMR (100 MHz) spectrum of compound 29 in $CDCl_3$

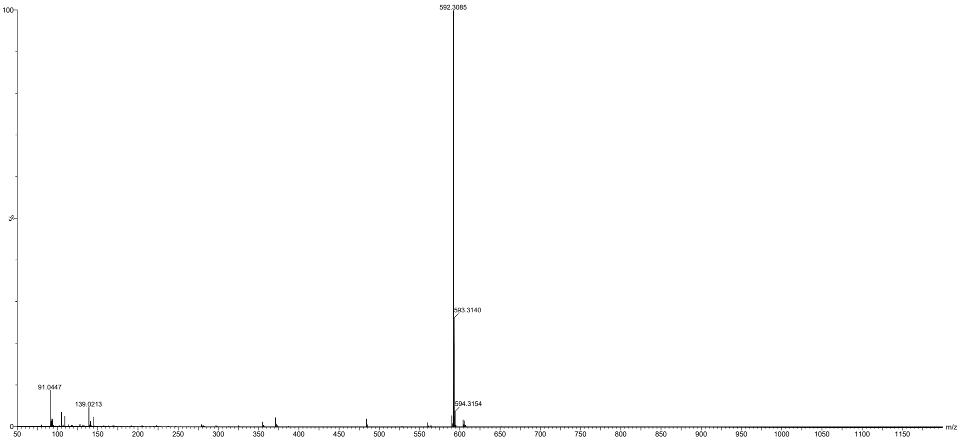

figure 90 HR-ESI-MS data of Compound 29

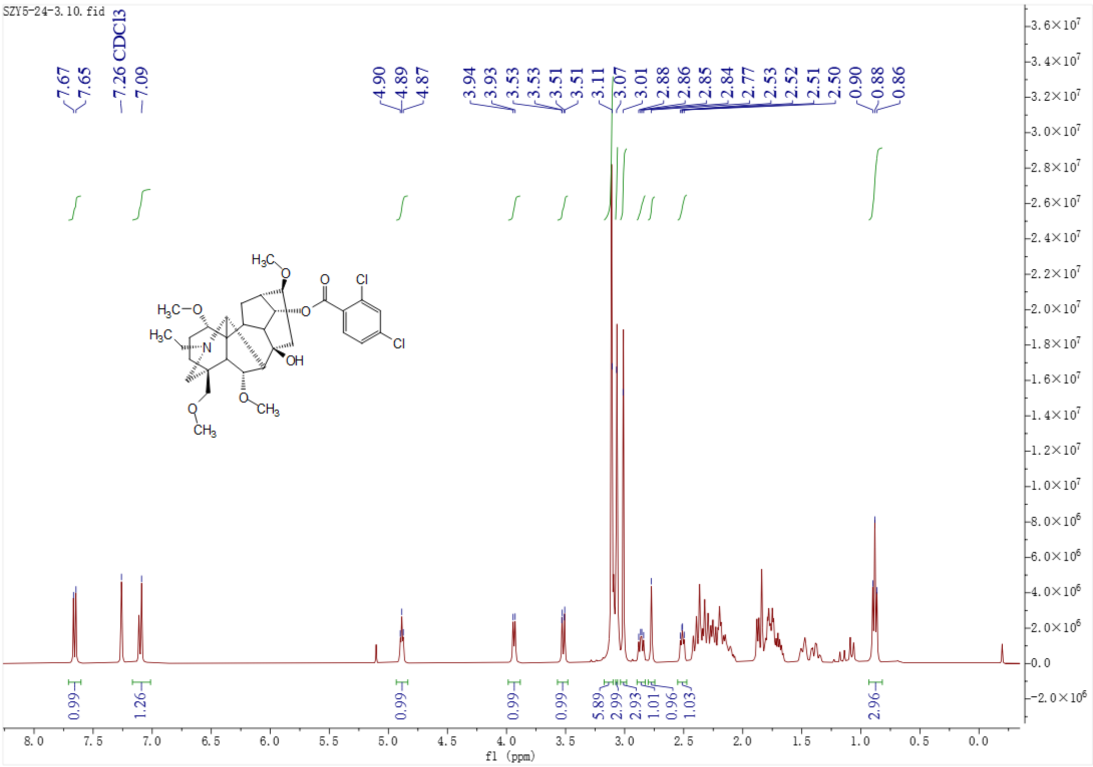

figure 91 ^{1}H NMR (400 MHz) spectrum of compound 30 in $CDCl_3$

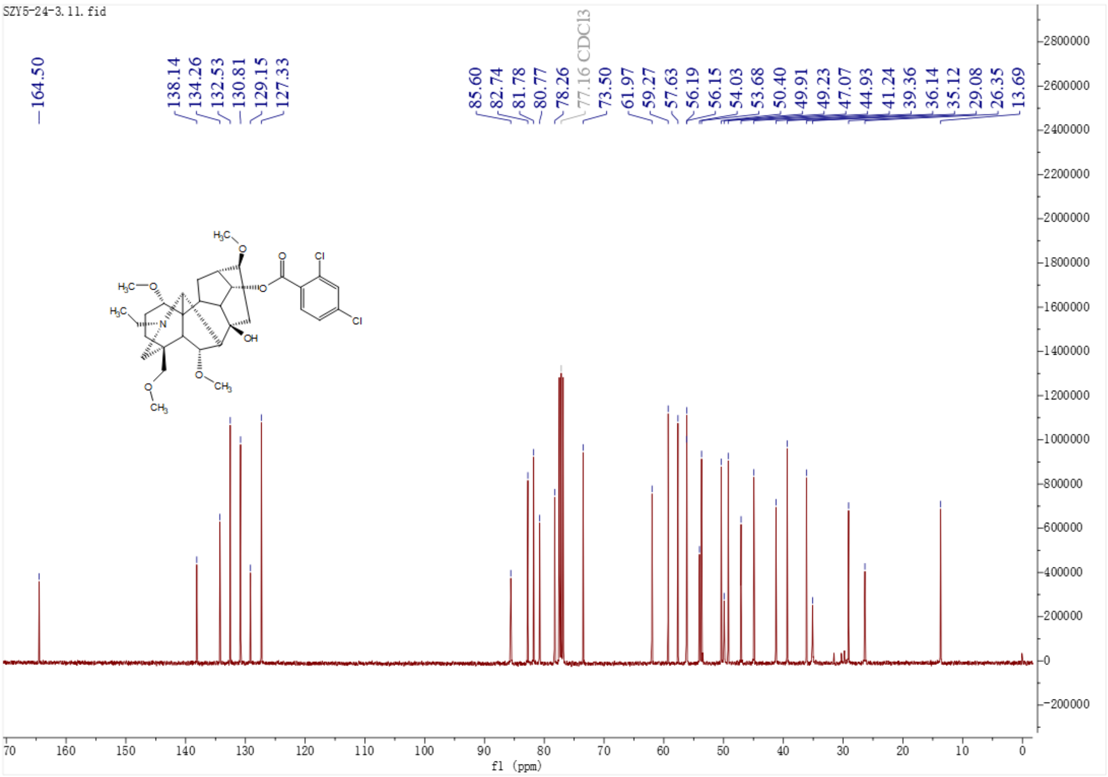

figure 92 ^{13}C NMR (100 MHz) spectrum of compound 30 in $CDCl_3$

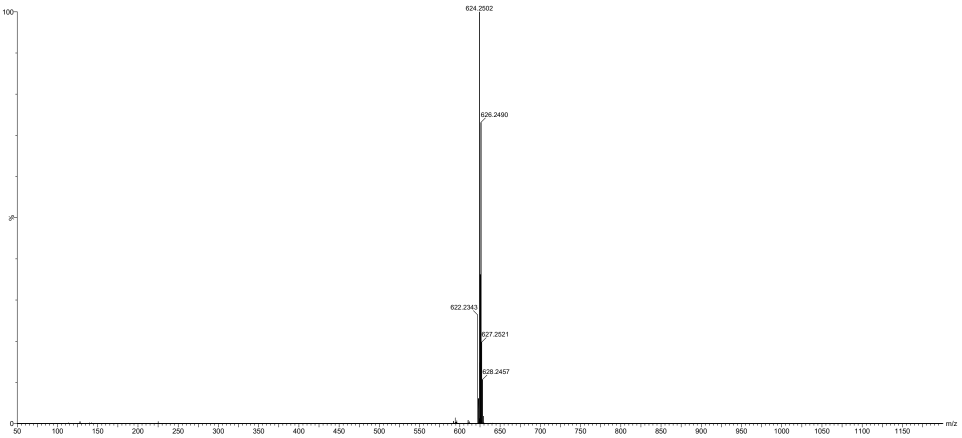

figure 93 HR-ESI-MS data of Compound 30

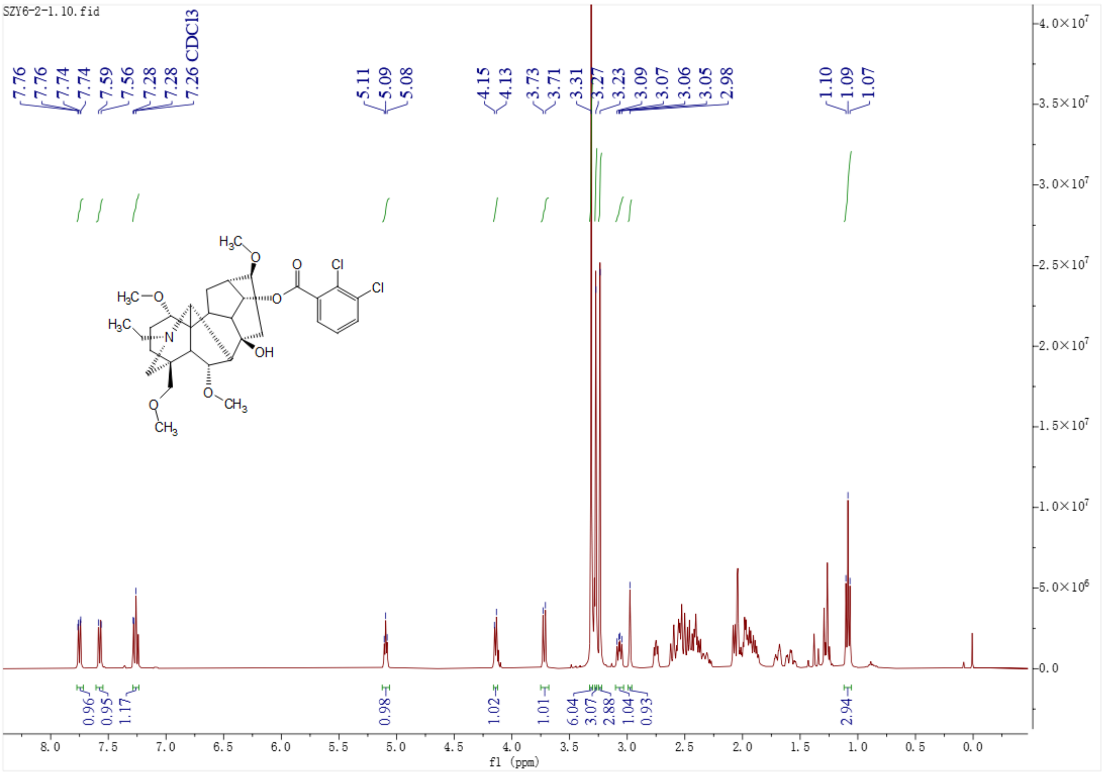

figure 94 ^{1}H NMR (400 MHz) spectrum of compound 31 in $CDCl_3$

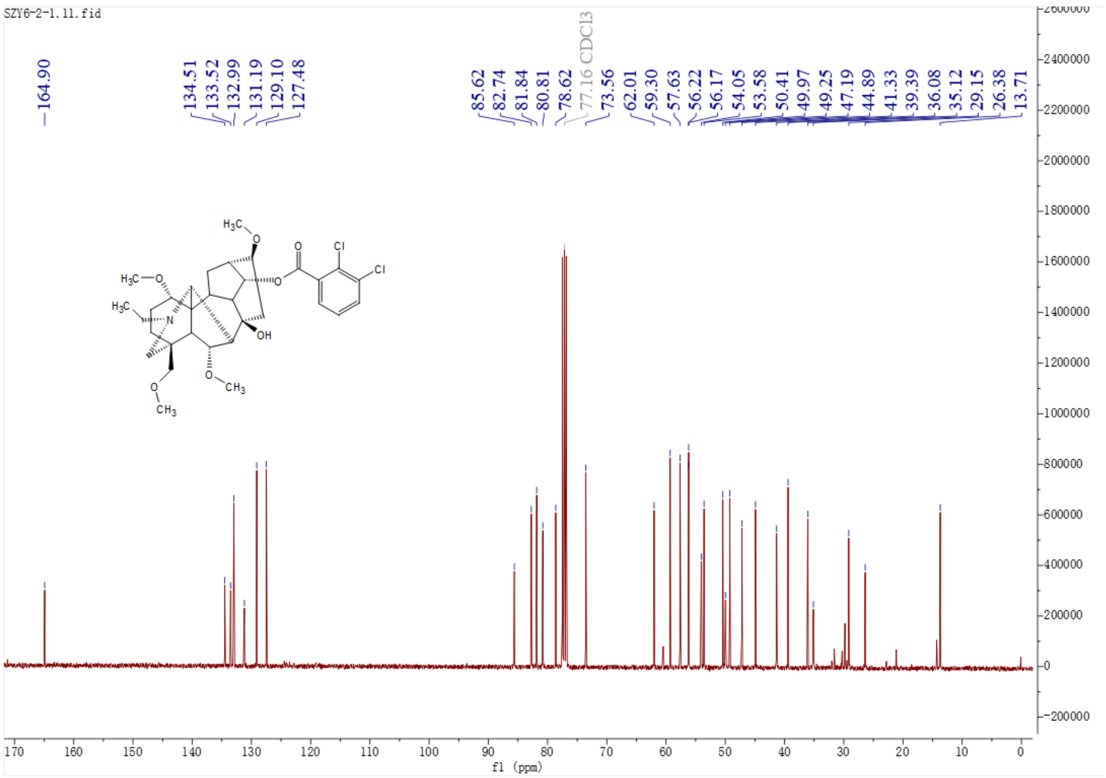

figure 95 ^{13}C NMR (100 MHz) spectrum of compound 31 in $CDCl_3$

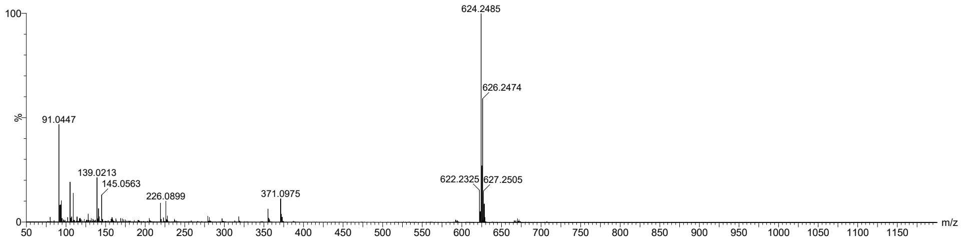

figure 96 HR-ESI-MS data of Compound 31

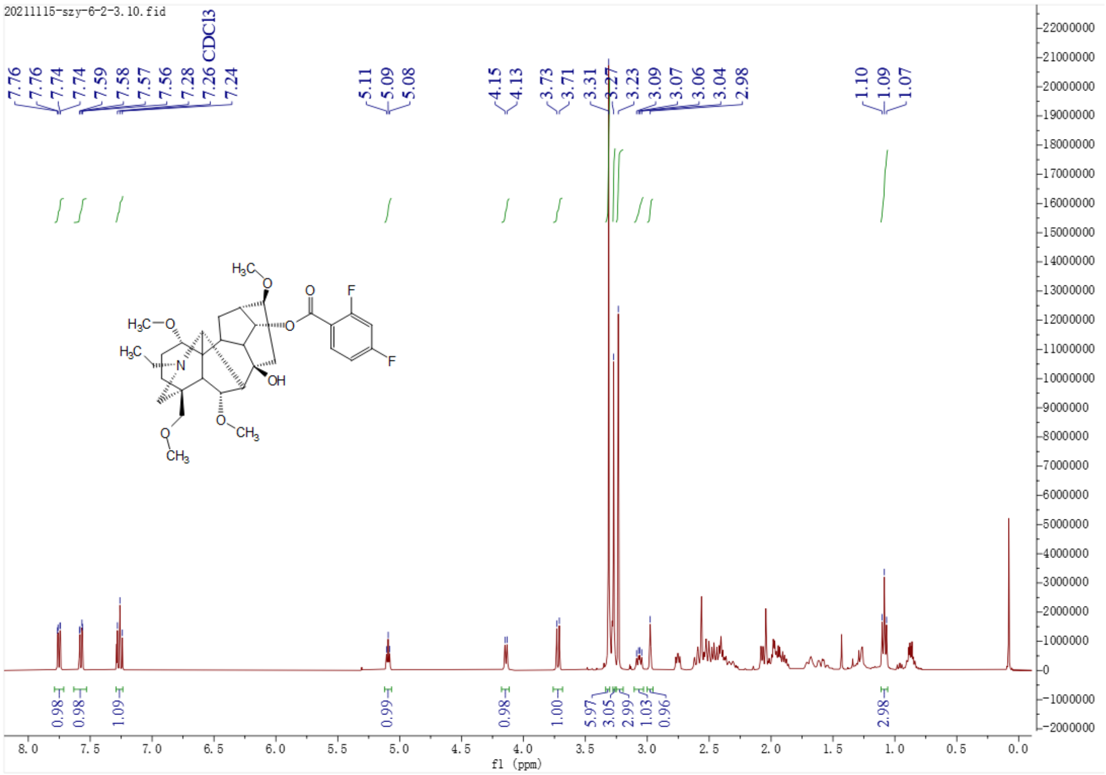

figure 97 ^{1}H NMR (400 MHz) spectrum of compound 32 in $CDCl_3$

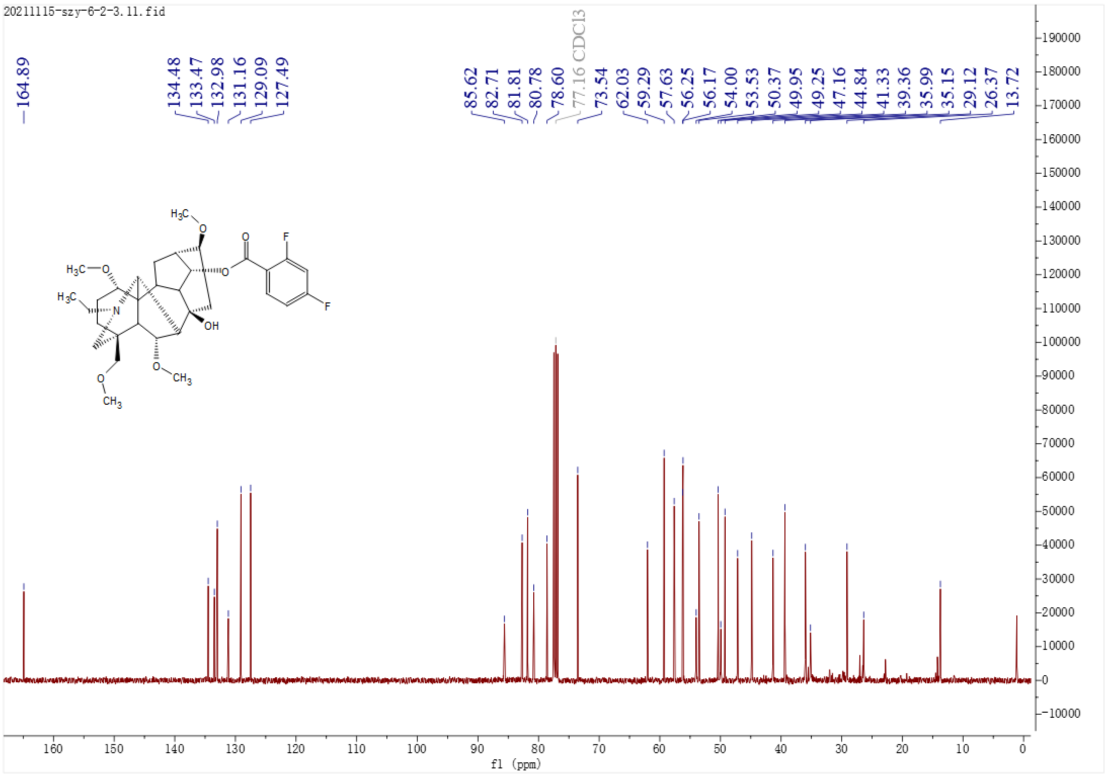

figure 98 ^{13}C NMR (100 MHz) spectrum of compound 32 in $CDCl_3$

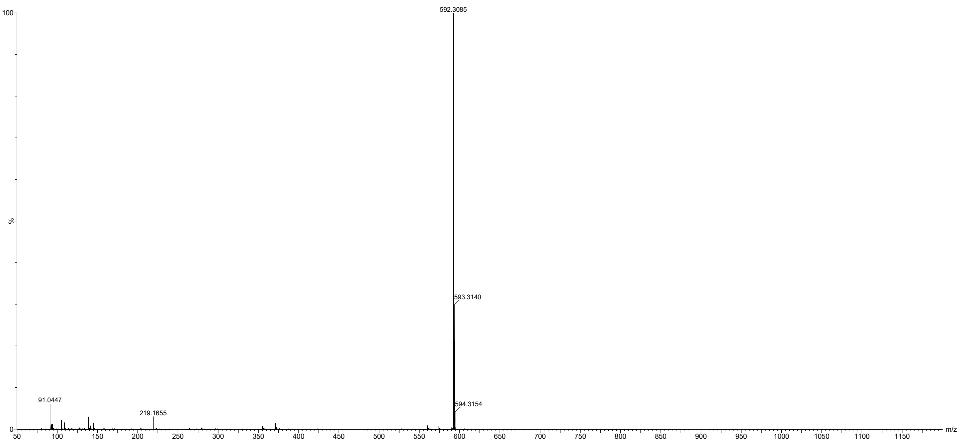

figure 99 HR-ESI-MS data of Compound 32

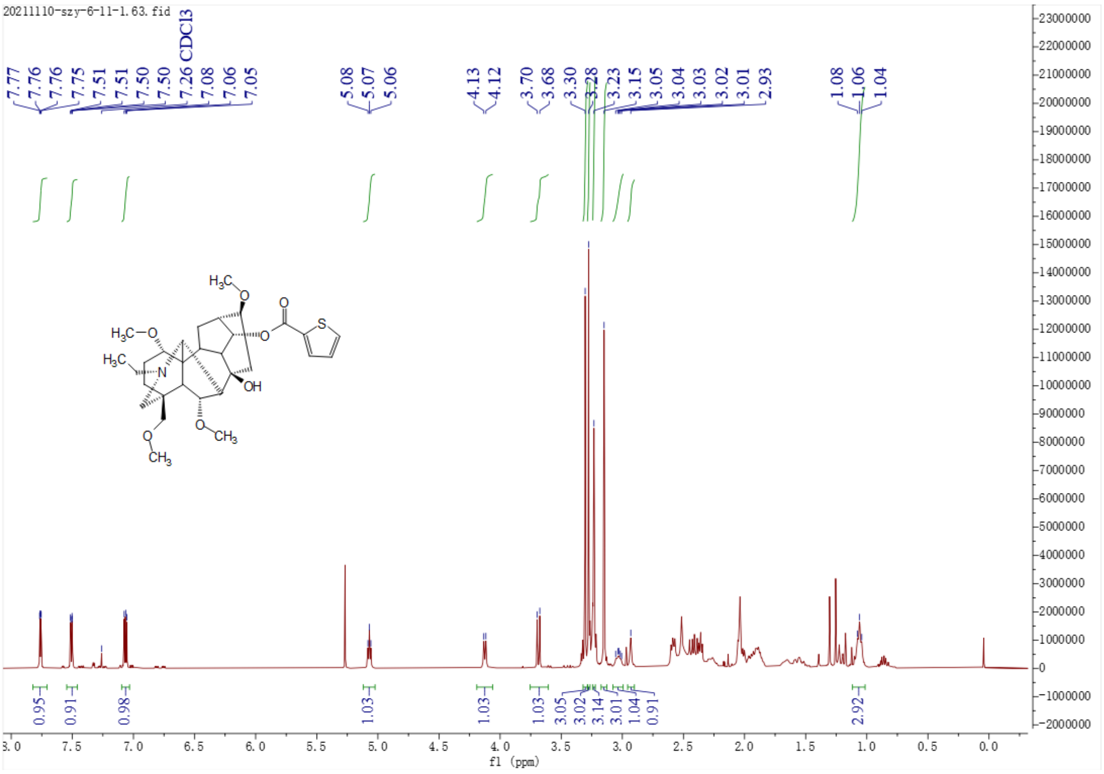

figure 100. ^{1}H NMR (400 MHz) spectrum of compound 33 in $CDCl_3$

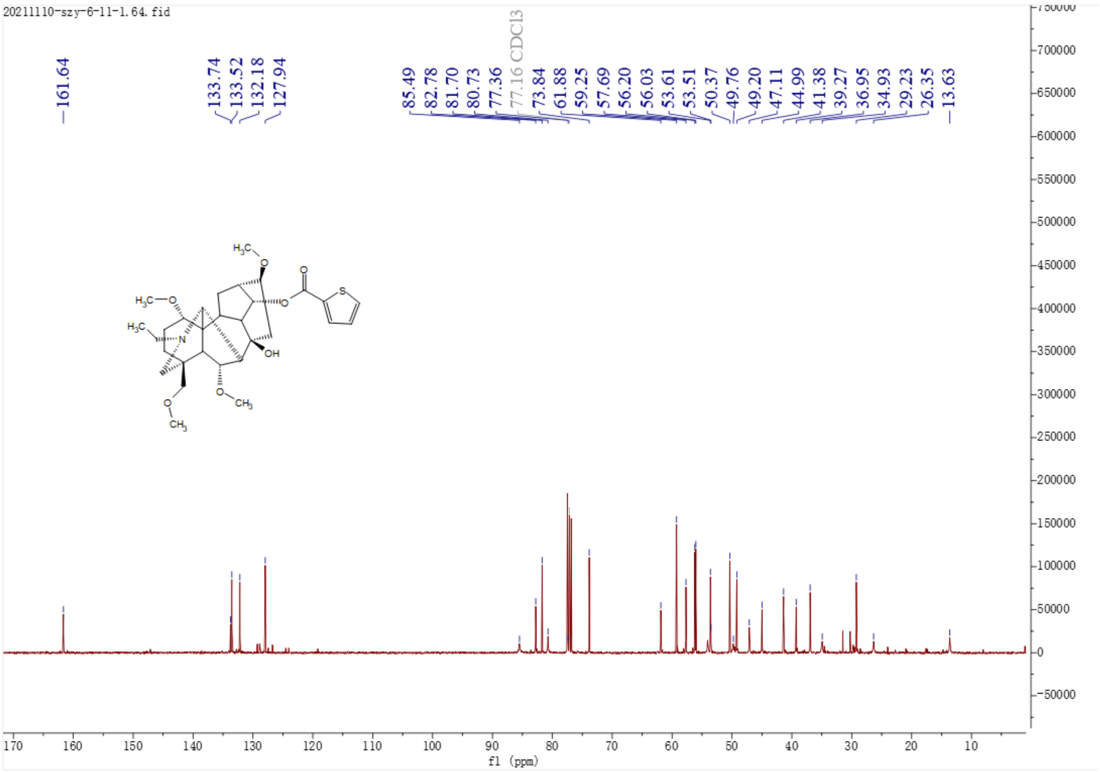

figure 101 ¹³C NMR (100 MHz) spectrum of compound 33 in CDCl₃

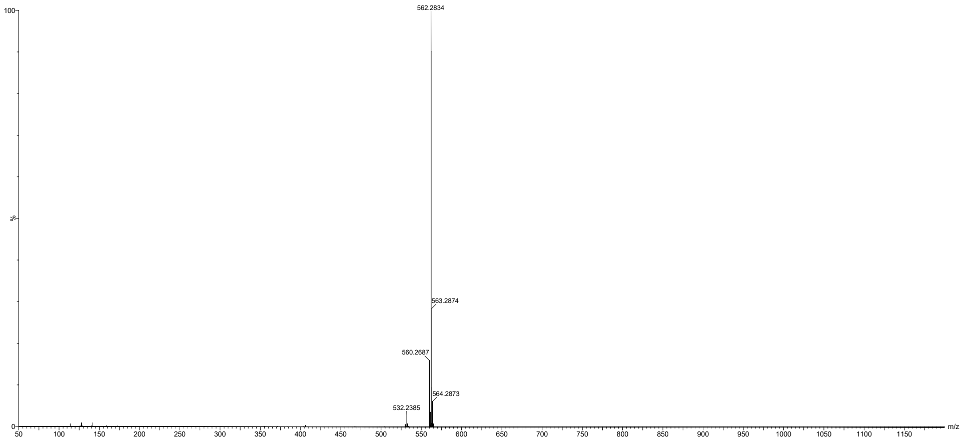

figure 102 HR-ESI-MS data of Compound 33

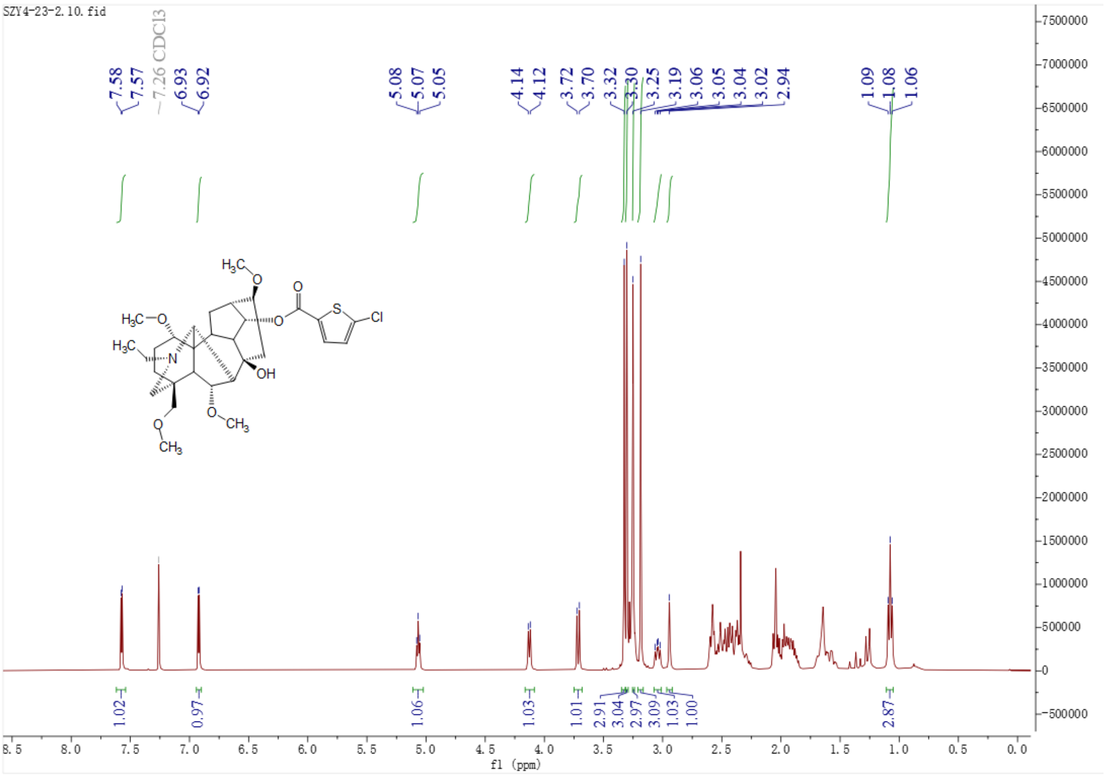

figure 103 ^{1}H NMR (400 MHz) spectrum of compound 34 in $CDCl_3$

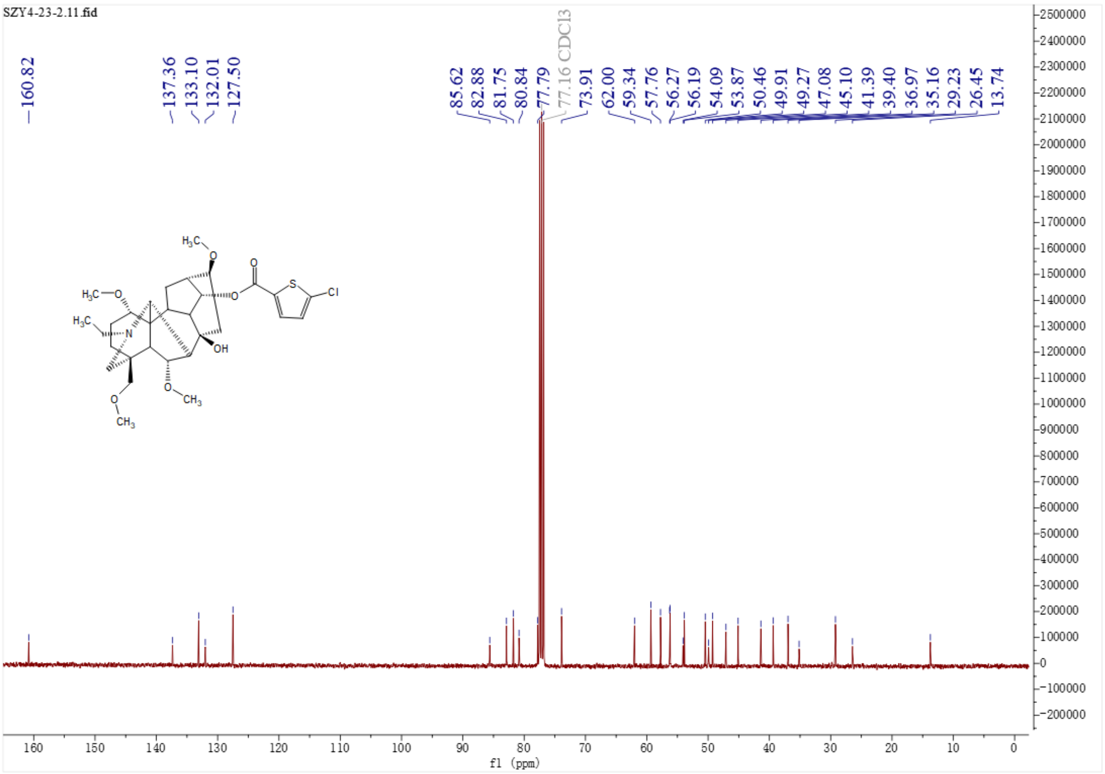

figure 104 ^{13}C NMR (100 MHz) spectrum of compound 34 in $CDCl_3$

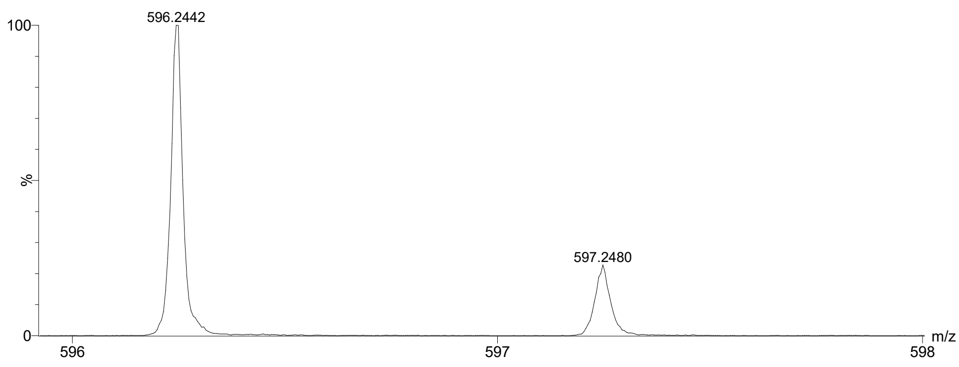

figure 105 HR-ESI-MS data of Compound 34

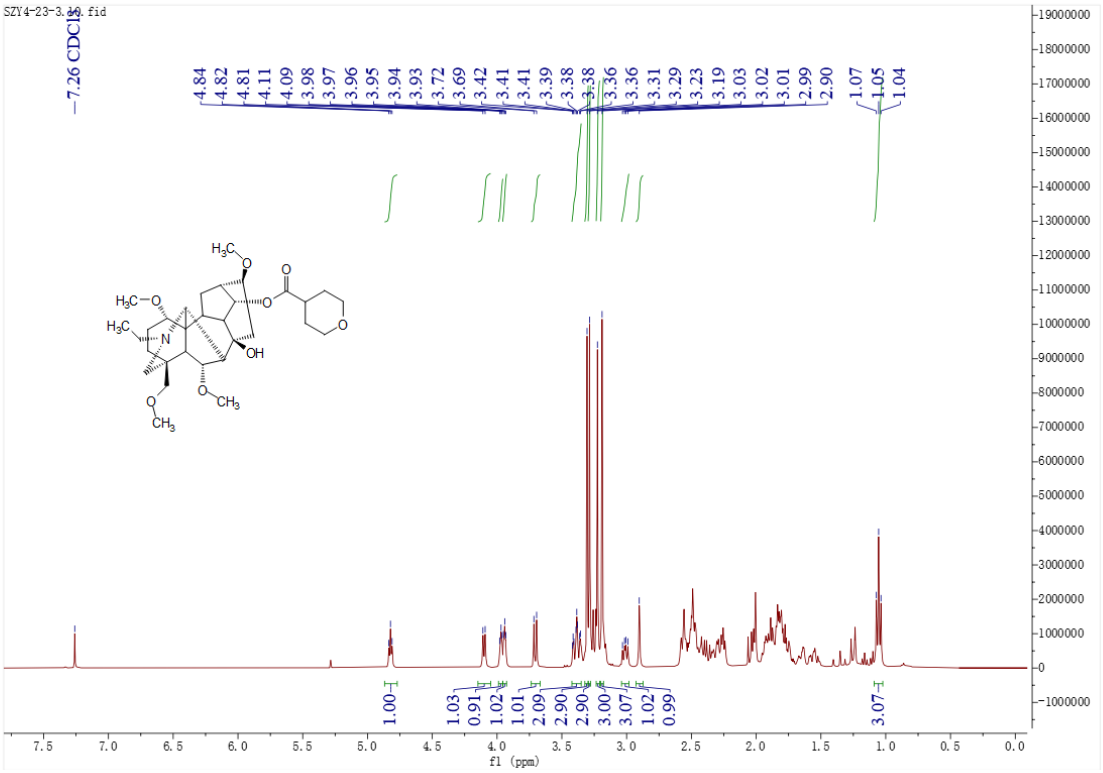

figure 106 ^{1}H NMR (400 MHz) spectrum of compound 35 in CDCl3

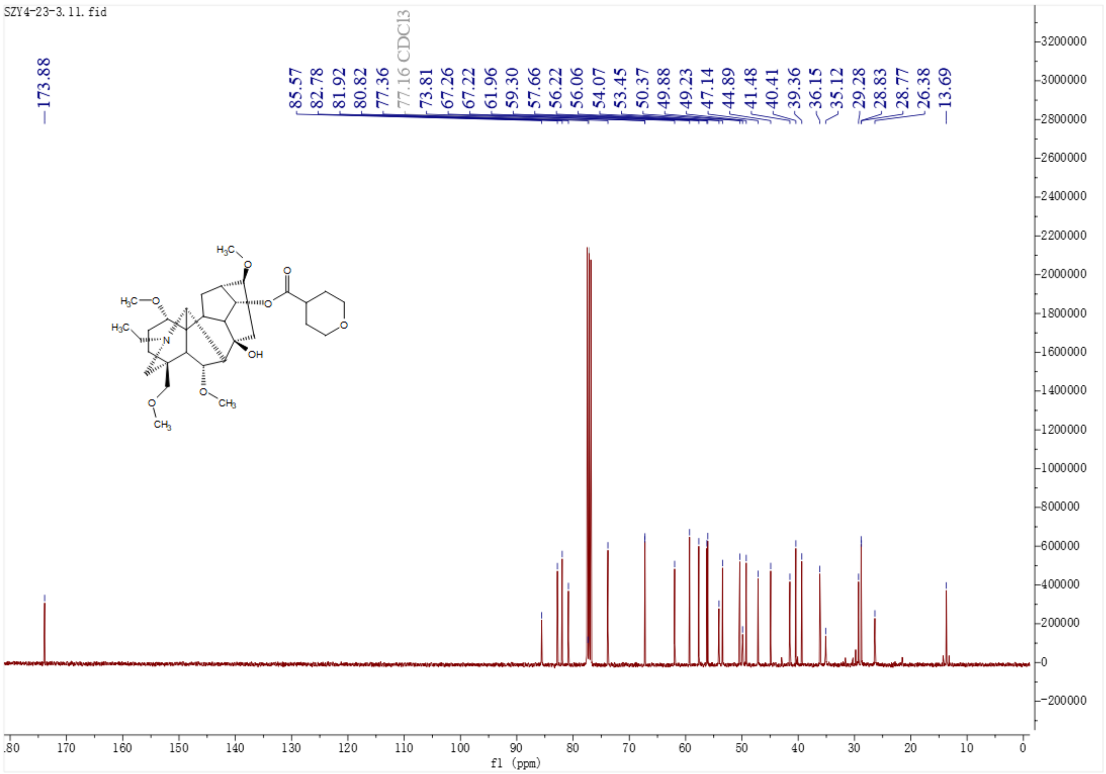

figure 107 ^{13}C NMR (100 MHz) spectrum of compound 35 in $CDCl_3$

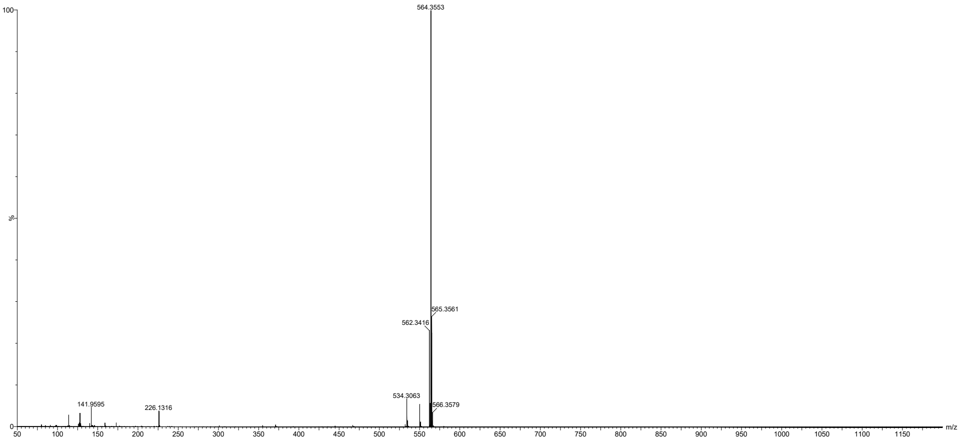

figure 108 HR-ESI-MS data of Compound 35

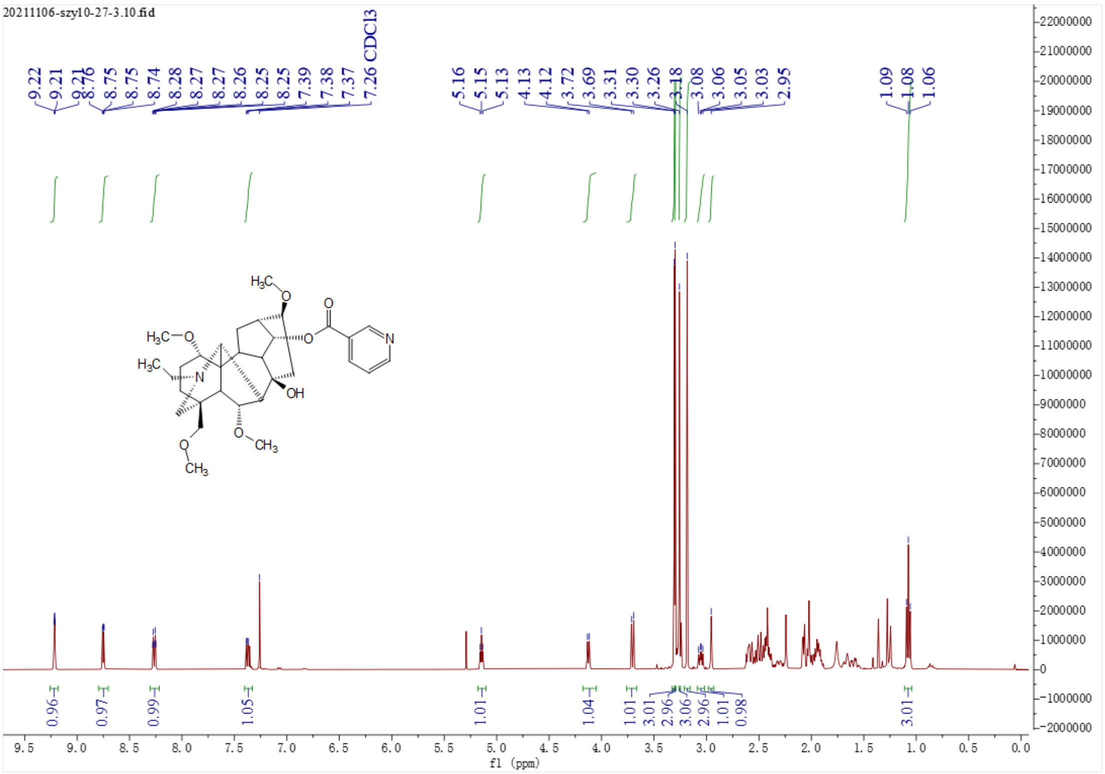

figure 109 ^{1}H NMR (400 MHz) spectrum of compound 36 in $CDCl_3$

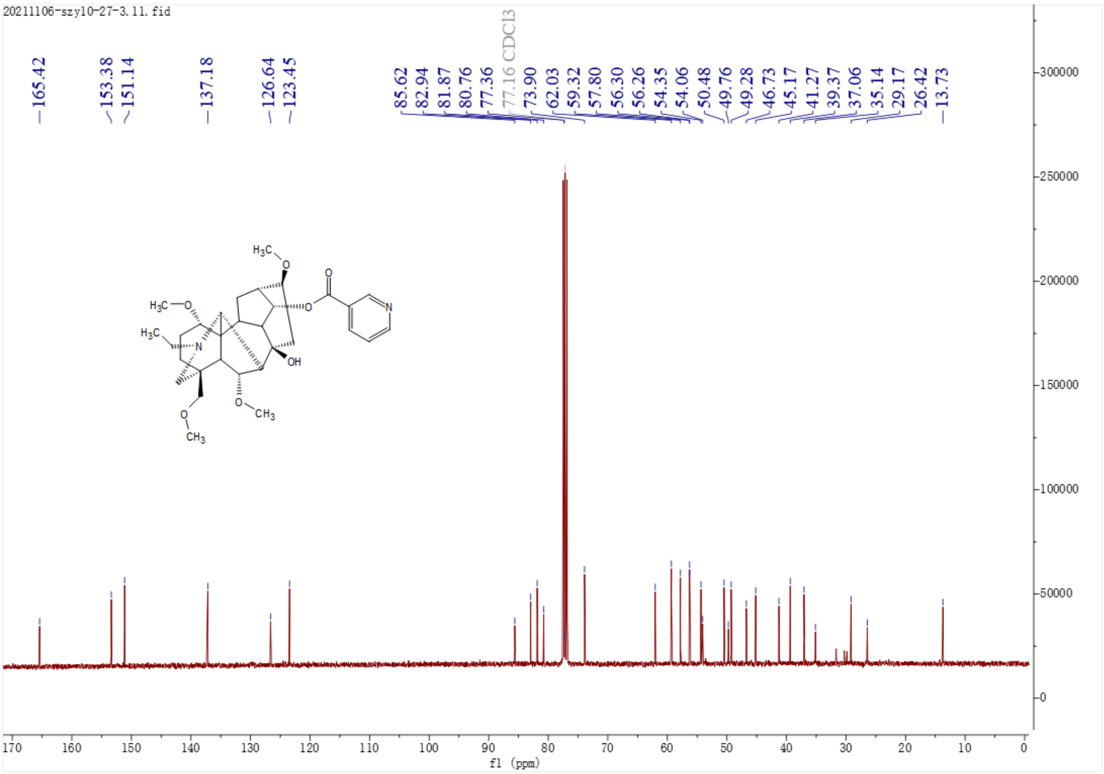

figure 110 ^{13}C NMR (100 MHz) spectrum of compound 36in $CDCl_3$

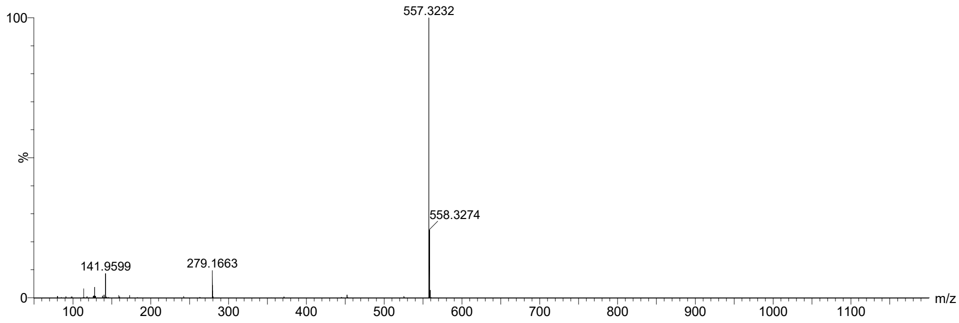

figure 111 HR-ESI-MS data of Compound 36

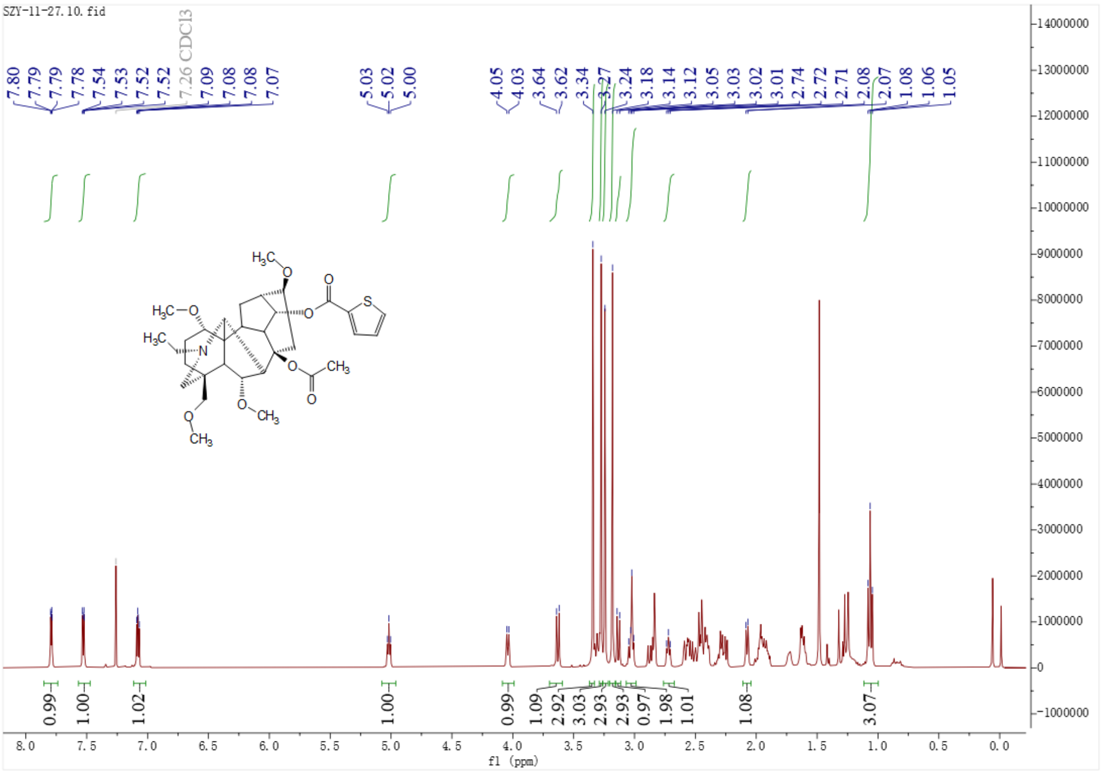

figure 112 ^{1}H NMR (400 MHz) spectrum of compound 37 in $CDCl_3$

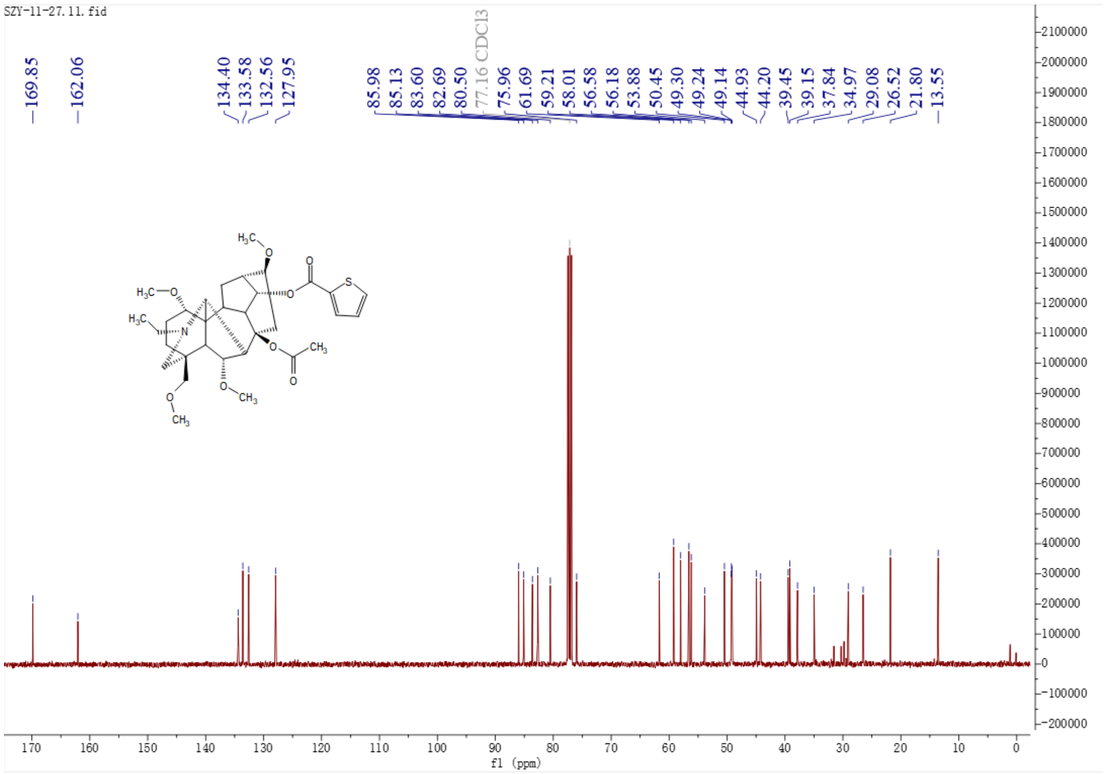

figure 113 ^{13}C NMR (100 MHz) spectrum of compound 37 in $CDCl_3$

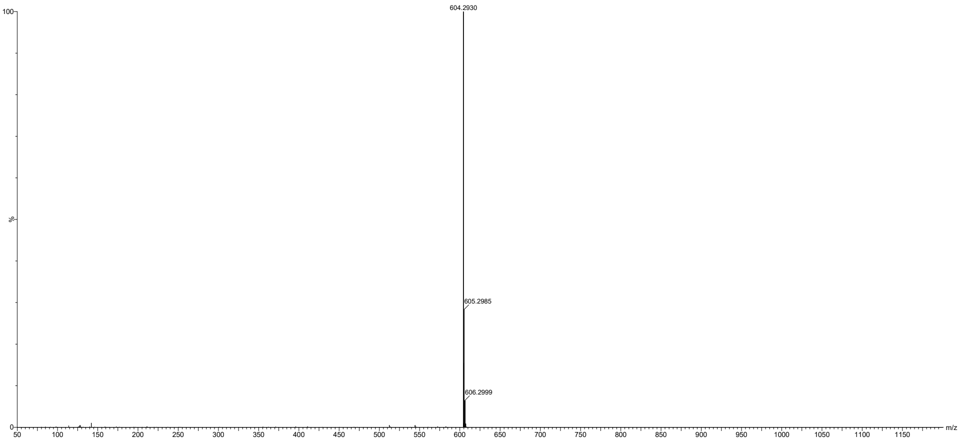

figure 114 HR-ESI-MS data of Compound 37

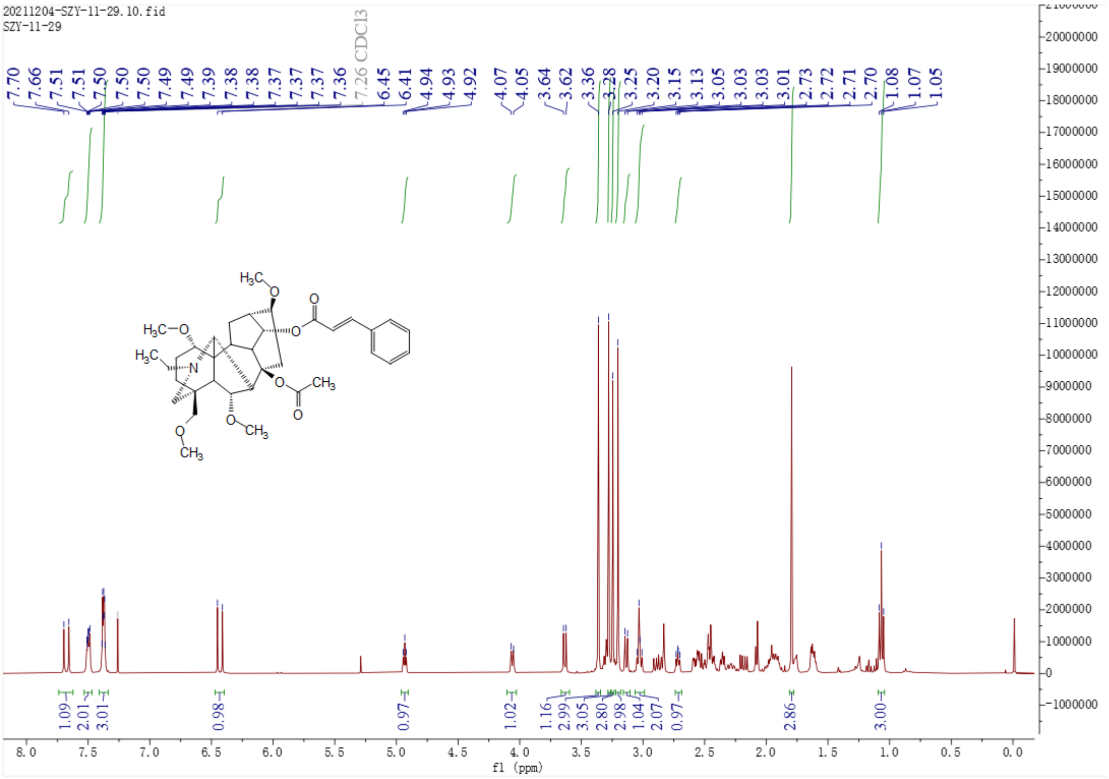

figure 115 ^{1}H NMR (400 MHz) spectrum of compound 38 in $CDCl_3$

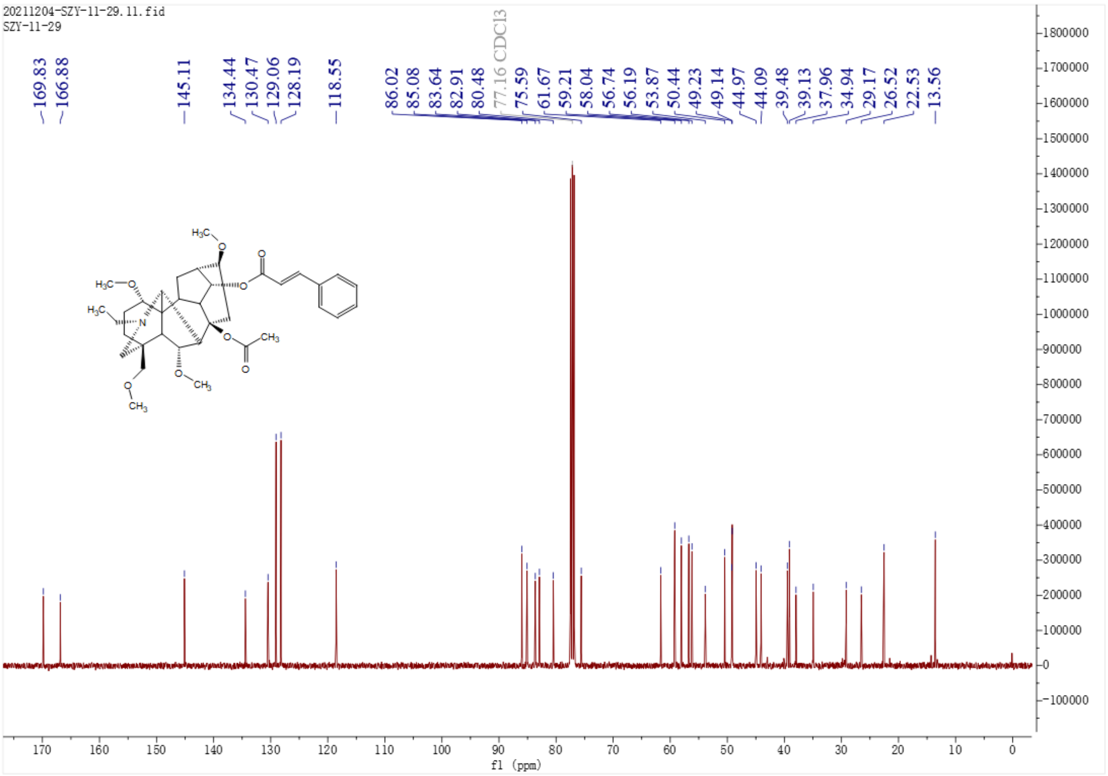

figure 116 ^{13}C NMR (100 MHz) spectrum of compound 38 in $CDCl_3$

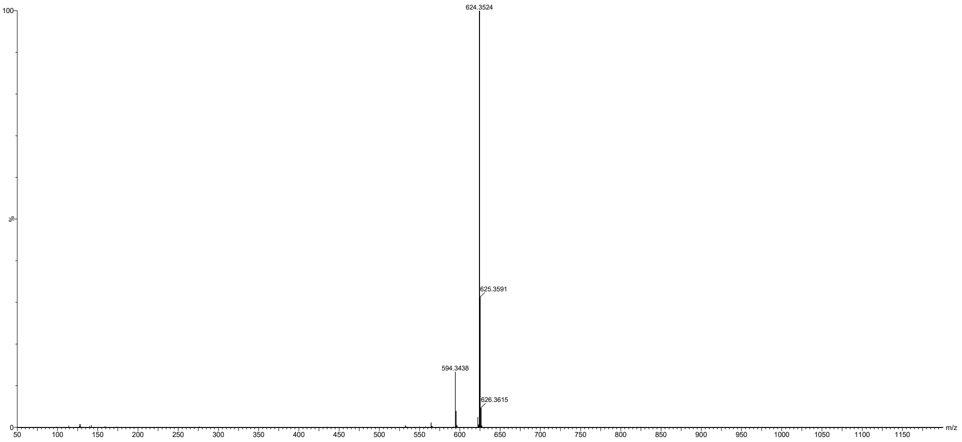

figure 117 HR-ESI-MS data of Compound 38

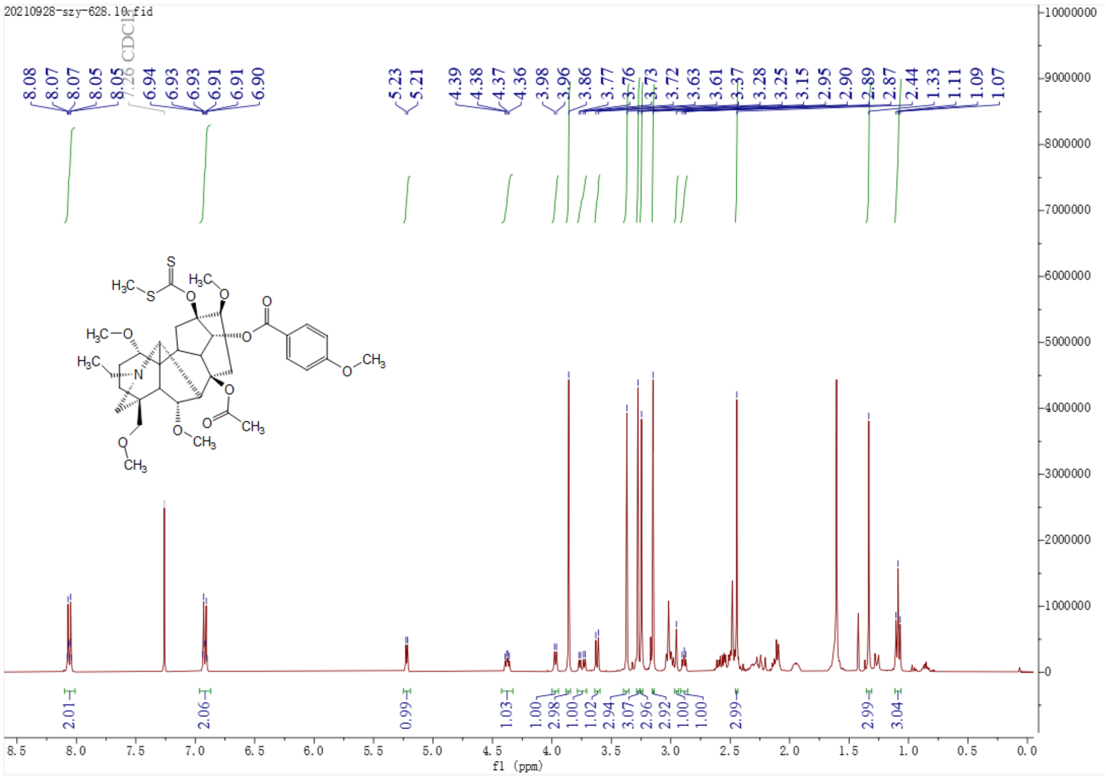

figure 118 ^{1}H NMR (400 MHz) spectrum of Intermediate 1 in $CDCl_3$

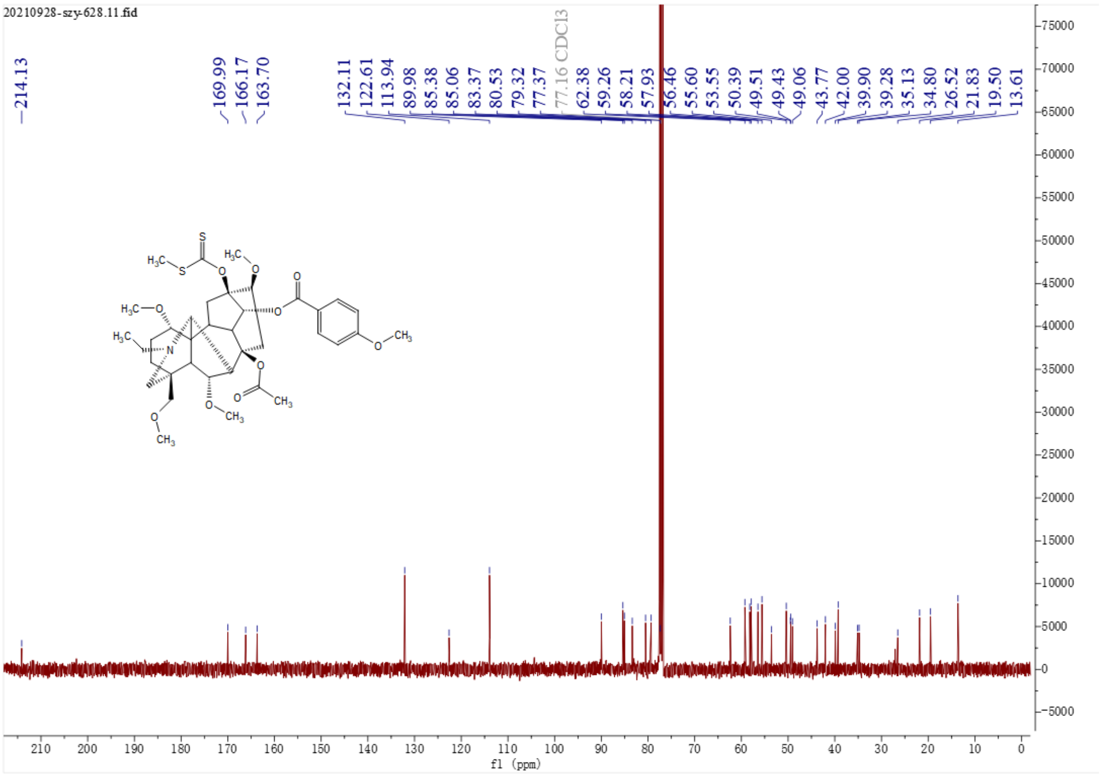

figure 119 ^{13}C NMR (100 MHz) spectrum of Intermediate 1 in $CDCl_3$

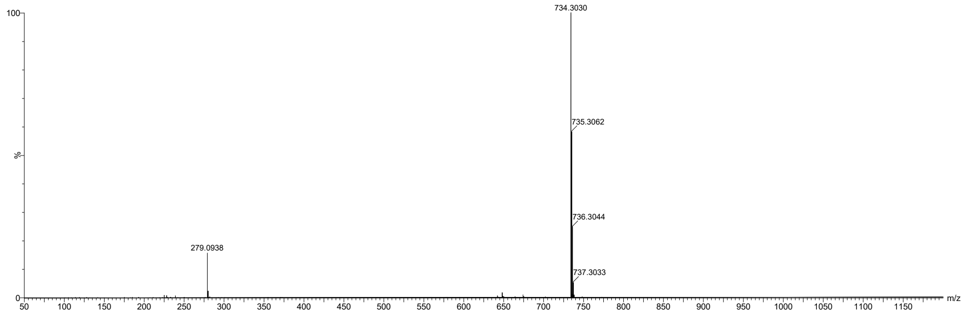

figure 120 HR-ESI-MS data of Intermediate 1

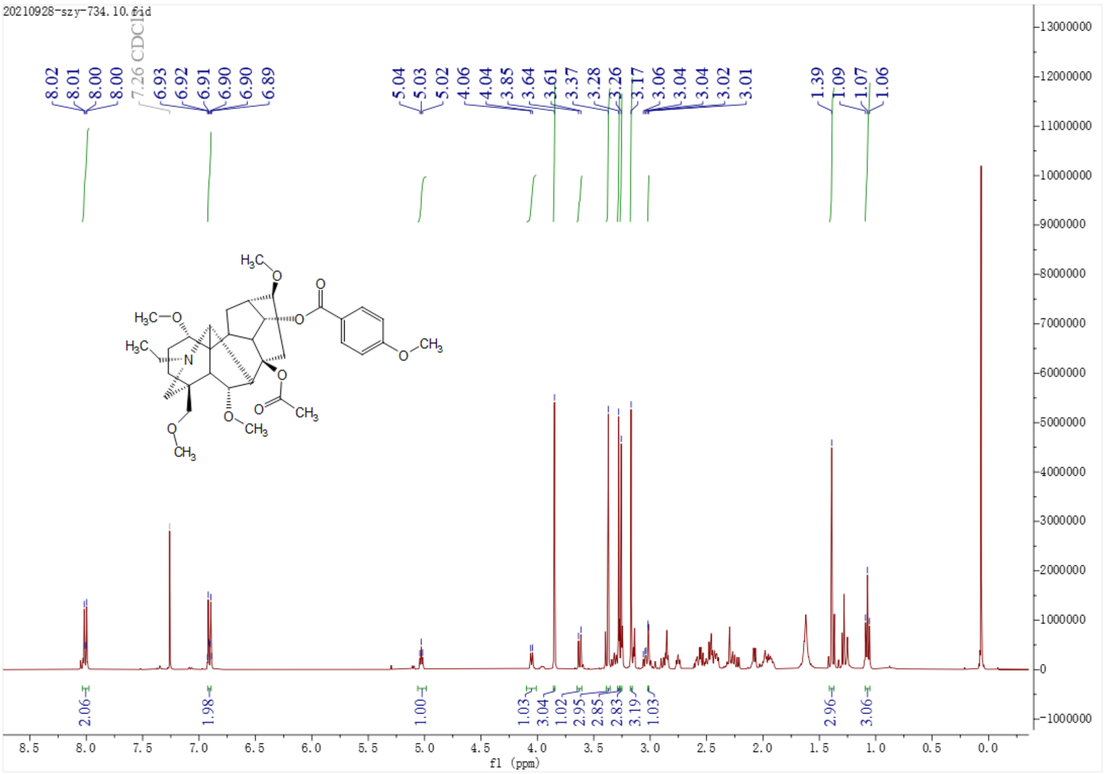

figure 121 ^{1}H NMR (400 MHz) spectrum of Intermediate 2 in $CDCl_3$

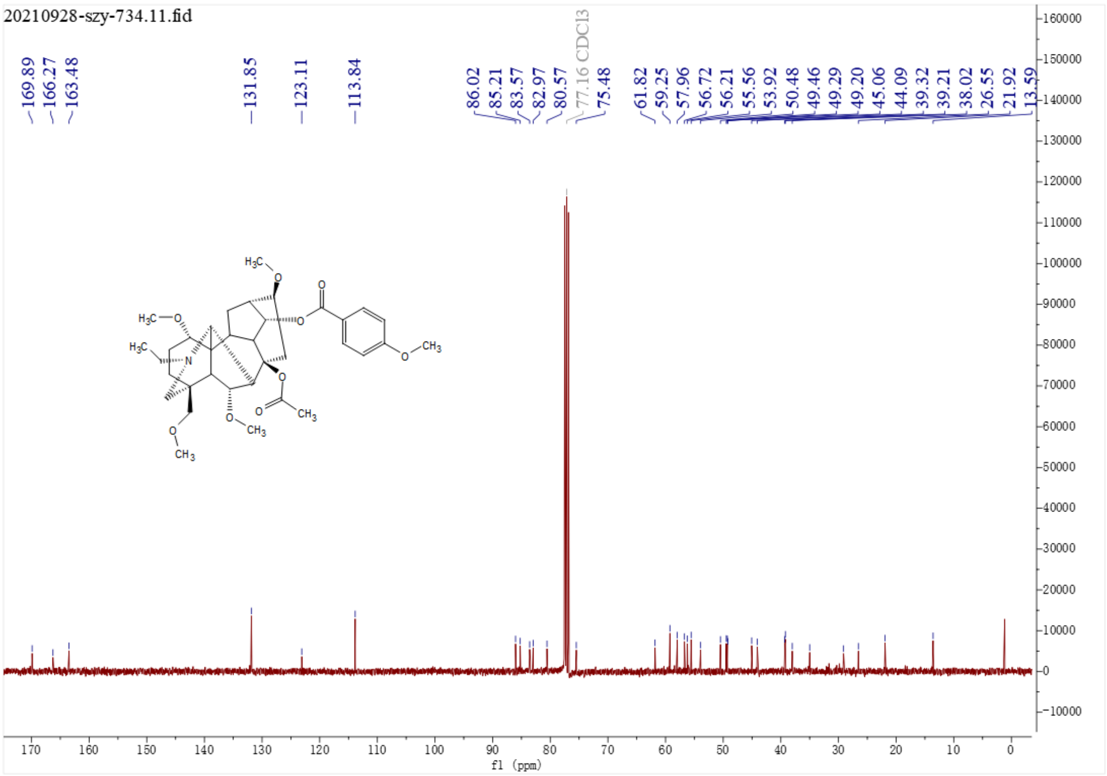

figure 122 ^{13}C NMR (100 MHz) spectrum of Intermediate 2 in $CDCl_3$

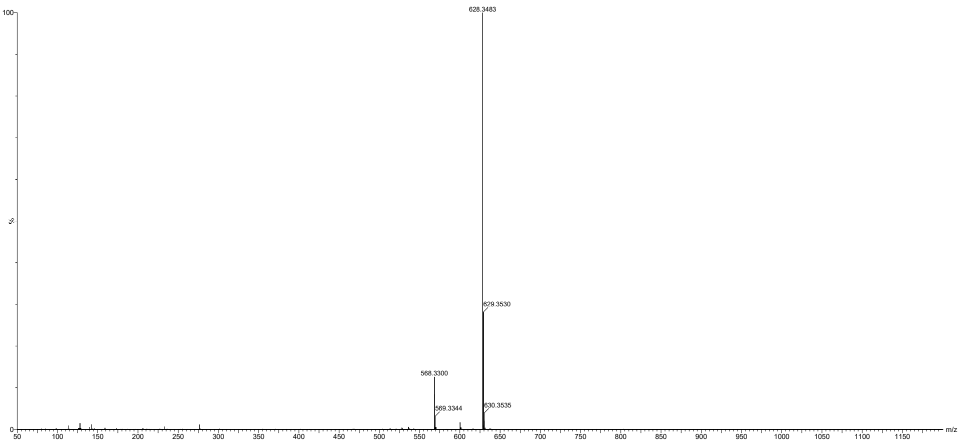

figure 123 HR-ESI-MS data of Intermediate 2
