# Supplementary material for: Design, synthesis and insecticidal activity and mechanism research of Chasmanthinine derivatives
Source: Sci Rep. 2022 Sep 10;12:15290. doi: 10.1038/s41598-022-19523-8 (PMC9464227; doi:10.1038/s41598-022-19523-8)
Supplement: Supplementary file 2 — Supplementary Information 2. [file 41598_2022_19523_MOESM2_ESM.docx]

**Raw data of Manuscript**

**Of**

**Design, synthesis and insecticidal activity and mechanism research of Chasmanthinine derivatives**

Ziyu Song,^aǂ^ Xiangyu Li,^aǂ^ Ke Xu,^a^ Guoqing Sun,^a^ Liu Yang,^a^ Linyu Huang,^a^ Junqi Liu,^a^ Pengyuan Yin,^a^ Shuai Huang,^a^ Feng Gao,^a^ Xianli Zhou^a,b*^and Lin Chen^a*^

^a^School of Life Science and Engineering, Southwest Jiaotong University, Chengdu 610031, Sichuan, P.R. China.

^b^Affiliated Hospital of Southwest Jiaotong University ＆ The Third People Hospital of Chengdu, Chengdu 610031, Sichuan, P.R. China.

*Corresponding Author: Phone +86-28-887603201; E-mail: [zhouxl@swjtu.edu.cn](mailto:zhouxl@swjtu.edu.cn); linch@swjtu.edu.cn.

^ǂ^ These authors contributed equally to this work and should be considered co-first authors.

**CONTENT**

[Raw data of antifeedant activity 3](#_Toc111026278)

[Raw data on cytotoxicity of Sf9 cells 6](#_Toc111026279)

[Raw data of contact toxicity against *S. exigua* (Hübner) 8](#_Toc111026280)

[Raw data on inhibition rate of enzymes 9](#_Toc111026281)

# Raw data of antifeedant activity

Table R1 The refusal rate (FR) of target compounds against *S. exigua* at 0.50 mg/mL.

| **Compd.** | **R** | **FR (%) ± SD** | **Compd.** | **R** | **FR (%) ± SD** | |
| --- | --- | --- | --- | --- | --- | --- |
| **1** | propionyl | 23.95 ± 0.14 | **21** | 4-chlorobenzoyl | | 44.00 ± 0.19 |
| **2** | butyryl | 36.57 ± 0.19 | **22** | 4-bromobenzoyl | | 71.23 ± 0.03 |
| **3** | 2-butenoyl | 41.89 ± 0.10 | **23** | 4-Fluorobenzoyl | | 63.17 ± 0.12 |
| **4** | valeryl | 28.76 ± 0.13 | **24** | 3-bromobenzoyl | | 56.07 ± 0.01 |
| **5** | heptanoyl | 53.75 ± 0.10 | **25** | 2-bromobenzoyl | | 37.18 ± 0.14 |
| **6** | isobutyryl | 21.65 ± 0.12 | **26** | 2-fluorobenzoyl | | 37.31 ± 0.07 |
| **7** | 3,3-dimethylbutyryl | 29.51 ± 0.15 | **27** | 4-chlorophenylacetyl | | 44.00 ± 0.11 |
| **8** | cyclohexylformyl | 43.76 ± 0.12 | **28** | 2,5-dichlorobenzoyl | | 45.58 ± 0.07 |
| **9** | cyclopropylformyl | 44.14 ± 0.23 | **29** | 3,5-difluorobenzoyl | | 53.05 ± 0.09 |
| **10** | palmitoyl | 26.02 ± 0.11 | **30** | 2,4-dichlorobenzoyl | | 21.45 ± 0.15 |
| **11** | propyl Formyl | 49.85 ± 0.17 | **31** | 2,3-dichlorobenzoyl | | 39.30 ± 0.04 |
| **12** | butyl Formyl | 19.00 ± 0.12 | **32** | 2,4-difluorobenzoyl | | 38.69 ± 0.15 |
| **13** | n-pentyl formyl | 34.03 ± 0.25 | **33** | thienoyl | | 90.20 ± 0.11 |
| **14** | isopropyl Formyl | 38.91 ± 0.22 | **34** | 2-chlorothiophene formyl | | 51.84 ± 0.20 |
| **15** | isobutyl formyl | 43.19 ± 0.13 | **35** | tetrahydropyranoyl | | 29.12 ± 0.11 |
| **16** | benzoyl | 46.17 ± 0.04 | **36** | Nicotinyl | | 48.00 ±0.10 |
| **17** | Phenylacetyl | 39.62 ± 0.03 | **37** | 8-OAc, 14- thienoyl | | 61.27 ± 0.12 |
| **18** | cinnamoyl | 83.33 ± 0.02 | **38** | 8-OAc, 14- cinnamoyl | | 73.12 ± 0.08 |
| **19** | 3-phenylpropionyl | 28.61 ± 0.21 | **Chasmanthinine** |  | | 90.07 ±0.07 |
| **20** | 4-methylbenzoyl | 30.38 ± 0.07 | **Azadirachtin A** |  | | 98.20 ± 1.29 |

The experiments were repeated three times.

**Table R2** Refusal rate (FR) of compounds in concentration gradients against *S. exigua* larvae

| **Compds.** | **FR (%) ± SD** | | | | | |
| --- | --- | --- | --- | --- | --- | --- |
|  | **0.0313 mg/mL** | **0.0625 mg/mL** | **0.1250 mg/mL** | **0.2500 mg/mL** | **0.5000 mg/mL** | **1.0000 mg/mL** |
| **18** | - | 19.00 ± 0.03 | 46.91 ± 0.03 | 71.31 ± 0.03 | 82.90 ± 0.04 | 86.62 ± 0.06 |
| **22** | - | 14.18 ± 0.12 | 26.88 ± 0.04 | 47.10 ± 0.07 | 66.51 ± 0.01 | 73.50 ± 0.10 |
| **23** | - | 12.00 ± 0.04 | 22.87 ± 0.12 | 37.45 ± 0.07 | 54.78 ± 0.10 | 67.21 ± 0.07 |
| **33** | 15.30 ± 0.04 | 31.90 ± 0.12 | 60.00 ± 0.05 | 79.42 ± 0.06 | 90.11 ± 0.05 | 93.60 ± 0.01 |
| **37** | - | 15.79 ± 0.04 | 33.33 ± 0.08 | 53.54 ± 0.13 | 72.40 ± 0.05 | 79.03 ± 0.06 |
| **38** | - | 15.55 ± 0.03 | 33.33 ± 0.10 | 52.20 ± 0.06 | 62.70 ± 0.06 | 70.00 ± 0.02 |
| **Chasmanthinine** | - | 23.68 ± 0.14 | 54.95 ± 0.11 | 79.51 ± 0.11 | 88.21 ± 0.06 | 92.71 ± 0.04 |
| **Azadirachtin A** | 10.1 ± 0.03 | 32.80 ± 0.04 | 67.38 ± 0.04 | 86.90 ± 0.04 | 94.82 ± 0.02 | 96.42 ± 0.01 |

The experiments were repeated three times.

**Table R3** EC_50_ values of antifeedant activity and regression equation of title compounds against *S. exigua* (Hübner).

| **Compds.** | **Regression equation** | **R^2^** | **EC_50_ (mg /cm^2^)** | **95% Confidence Interval** |
| --- | --- | --- | --- | --- |
| **18** | y = 2.84 / (1 + (x / 0.09) ^1.38) | 0.99976 | 0.13 | (0.12, 0.15) |
| **22** | y = 3.57 / (1 + (x / 0.18) ^1.55) | 0.99751 | 0.27 | (0.17, 0.46) |
| **23** | y = 2.74 / (1 + (x / 0.17) ^0.76) | 0.99901 | 0.41 | (0.30, 0.60) |
| **33** | y = 3.17 / (1 + (x / 0.10) ^1.11) | 0.99913 | 0.10 | (0.07, 0.15) |
| **37** | y =3.22 / (1 + (x / 0.14) ^1.21) | 0.99458 | 0.21 | (0.10, 0.49) |
| **38** | y = 1.56 / (1 + (x / 0.44) ^0.88) | 0.99972 | 0.24 | (0.20, 0.29) |
| **Chasmanthinine** | y = 1.91 / (1 + (x / 0.06) ^1.09) | 0.99771 | 0.11 | (0.07, 0.16) |
| **Azadirachtin A** | y = 2.52 / (1 + (x / 0.07) ^1.17) | 0.99966 | 0.08 | (0.08, 0.09) |

The experiments were repeated three times.


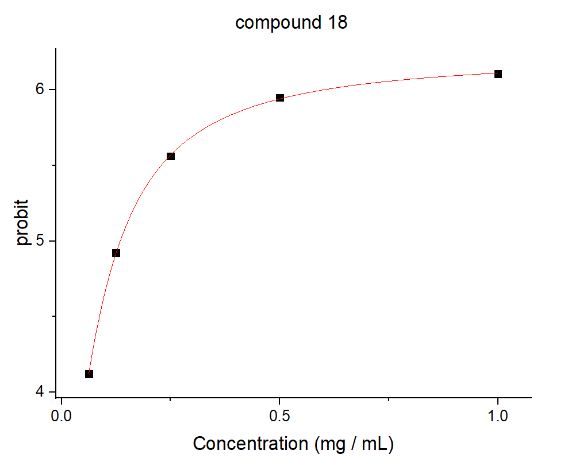


**Figure R1** Regression curve of compound 18 in antifeedant activity


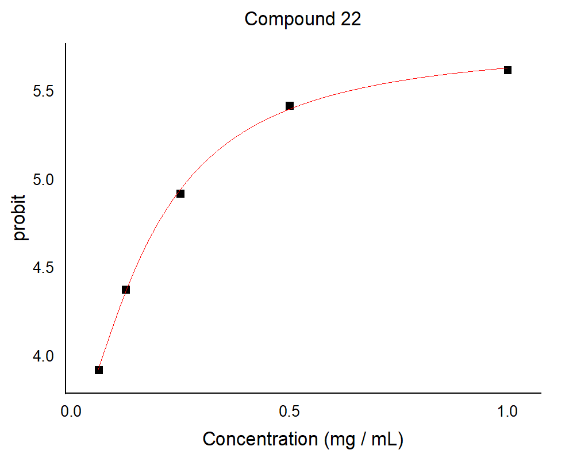


**Figure R2** Regression curve of compound 22 in antifeedant activity


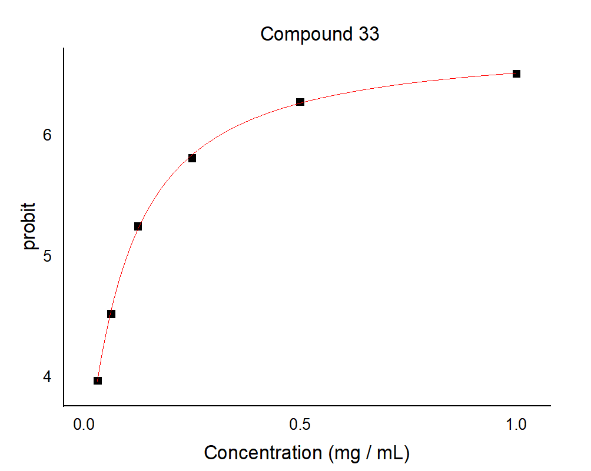

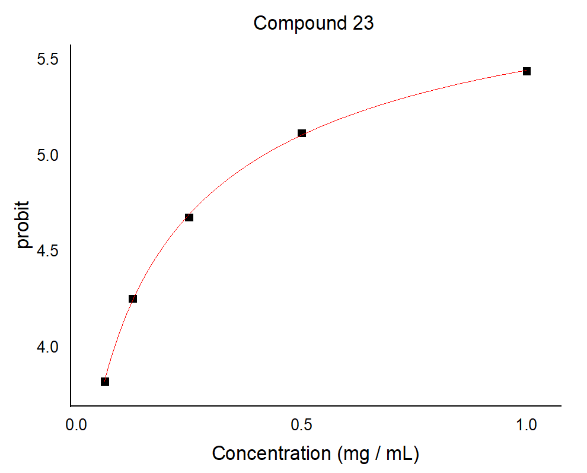


**Figure R4** Regression curve of compound 33 in antifeedant activity

**Figure R3** Regression curve of compound 23 in antifeedant activity


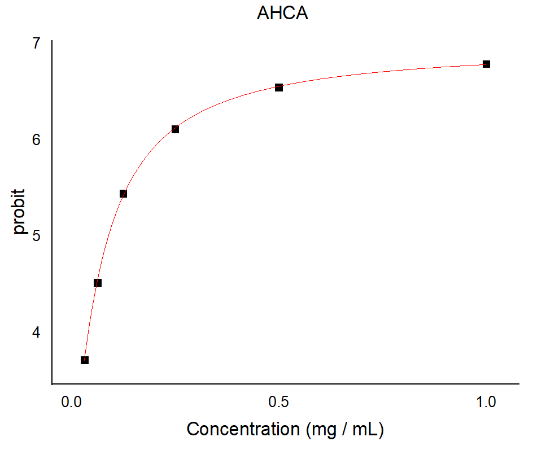

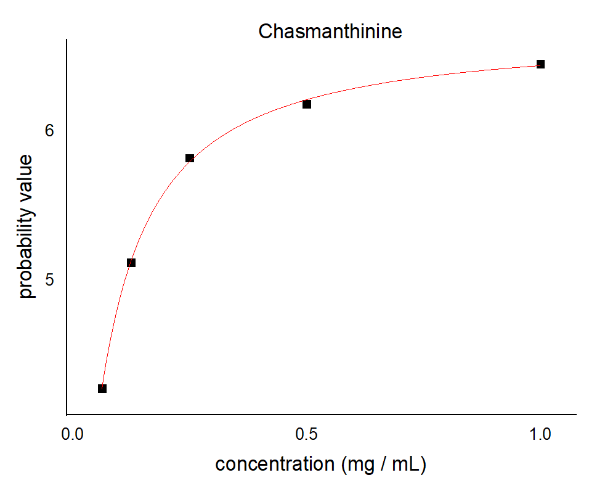

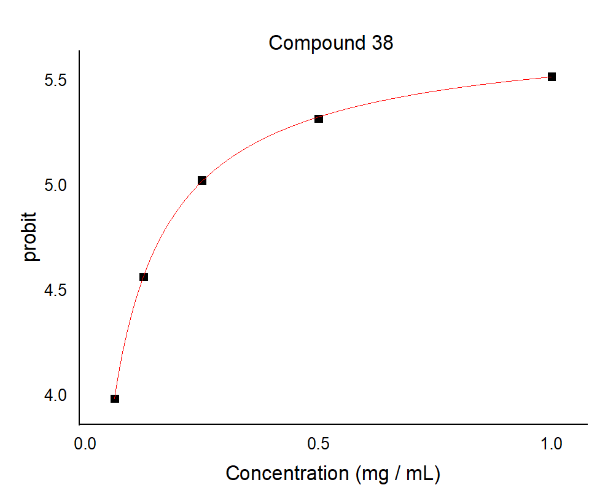

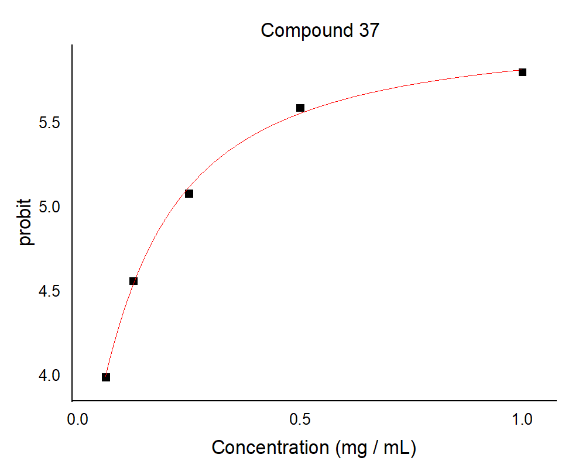


**Figure R8** Regression curve of Azadirachtin A in antifeedant activity

**Figure R7** Regression curve of Chasmanthinine in antifeedant activity

**Figure R6** Regression curve of compound 38 in antifeedant activity

**Figure R5** Regression curve of compound 37 in antifeedant activity

# Raw data on cytotoxicity of Sf9 cells

**Table R4** The inhibition rate (IR) of target compounds against Sf9 cells at 100 *μ*M.

| **Compds.** | **IR (%) ± SD** | **Compds.** | **IR (%) ± SD** |
| --- | --- | --- | --- |
| **1** | < 0 | **21** | 12.36 ± 7.80 |
| **2** | 5.04 ± 5.92 | **22** | 20.88 ± 3.39 |
| **3** | < 0 | **23** | 6.58 ± 2.79 |
| **4** | 42.40 ± 4.25 | **24** | 38.33 ± 3.16 |
| **5** | 19.86 ± 2.30 | **25** | 4.43 ± 2.90 |
| **6** | 15.45 ± 4.46 | **26** | 8.83 ± 7.69 |
| **7** | < 0 | **27** | 24.27 ± 1.60 |
| **8** | < 0 | **28** | 31.43 ± 2.13 |
| **9** | < 0 | **29** | < 0 |
| **10** | 94.88 ± 1.79 | **30** | 52.79 ± 1.42 |
| **11** | 5.24 ± 2.07 | **31** | 30.44 ± 4.60 |
| **12** | 8.12 ± 3.32 | **32** | 7.51 ± 2.08 |
| **13** | 5.16 ± 4.10 | **33** | < 0 |
| **14** | < 0 | **34** | 13.33 ± 3.37 |
| **15** | 1.30 ± 5.92 | **35** | 9.10 ± 6.71 |
| **16** | 6.67 ± 1.71 | **36** | < 0 |
| **17** | 18.64 ± 3.24 | **37** | 6.09 ± 6.29 |
| **18** | 1.64 ± 5.60 | **38** | 24.12 ± 1.38 |
| **19** | 30.46 ± 4.35 | **Azadirachtin A** | 98.02 ± 1.93 |
| **20** | < 0 |  |  |

The experiments were repeated six times.

**Figure R9** Inhibition rate (IR) of title compounds against Sf9 cells

**Table R5** Inhibition rate (IR) of compound **10** in concentration gradients against Sf9 cells

| **Concentration (*μ*mol/L)** | **IR (%) ± SD** |
| --- | --- |
| 3.00 | 17.50 ± 2.70 |
| 6.25 | 20.54 ± 2.21 |
| 12.50 | 46.55 ± 2.05 |
| 25.00 | 76.04 ± 1.45 |
| 50.00 | 90.91 ± 0.54 |
| 100.00 | 97.88 ± 1.42 |

The experiments were repeated six times.


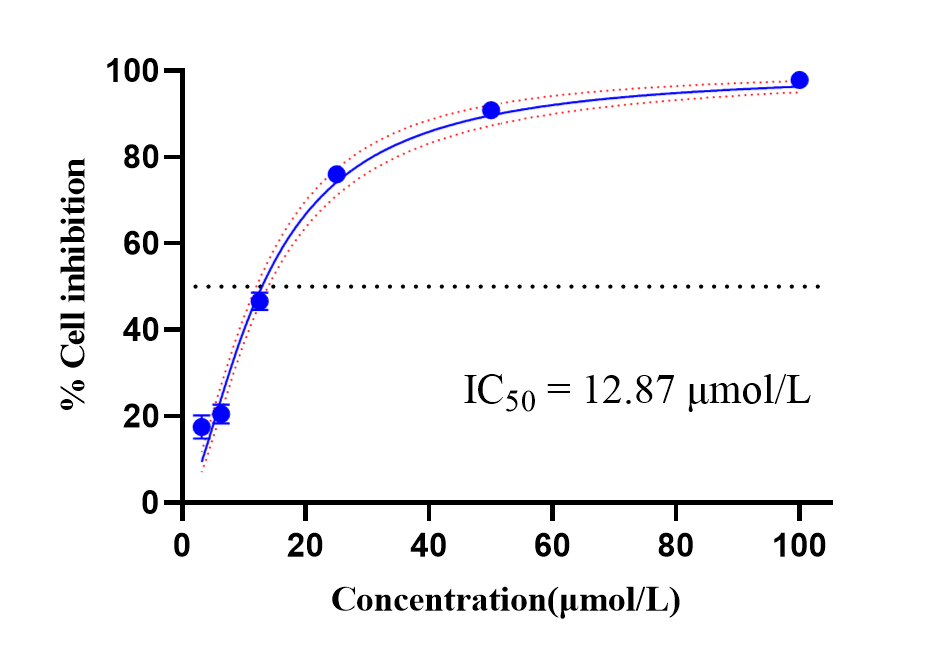


**Figure R10** Regression curve of compound **10** against Sf9 cells

**Table R6** Inhibition rate (IR) of compound **30** in concentration gradients against Sf9 cells

| **Concentration (*μ*mol/L)** | **IR (%) ± SD** |
| --- | --- |
| 50.00 | 6.94 ± 4.60 |
| 75.00 | 21.31 ± 1.26 |
| 100.00 | 32.18 ± 3.95 |
| 125.00 | 48.33 ± 2.22 |
| 150.00 | 62.40 ± 0.46 |
| 200.00 | 78.20 ± 0.13 |

The experiments were repeated six times.


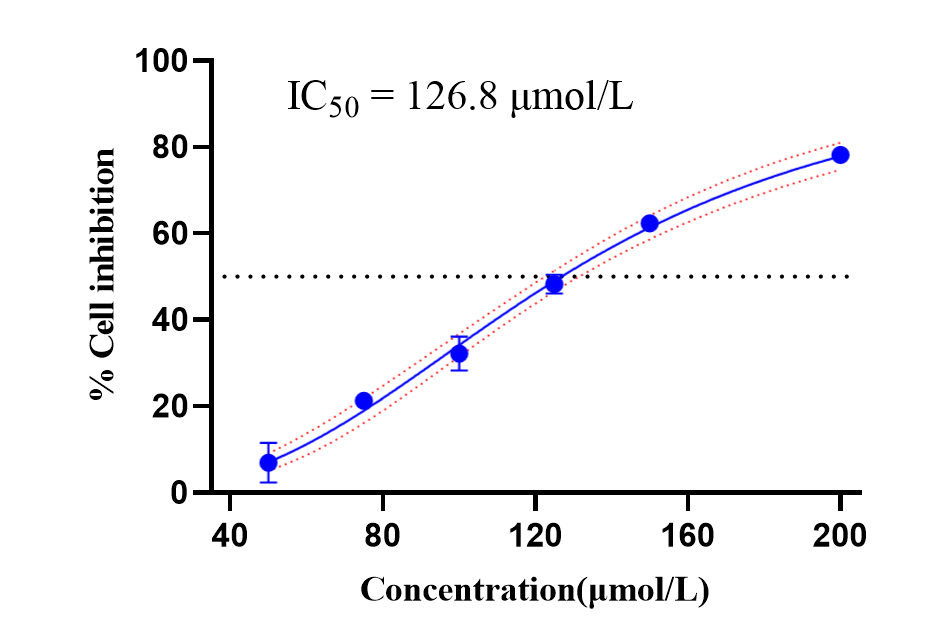


**Figure R11** Regression curve of compound **30** against Sf9 cells

# Raw data of contact toxicity against *S. exigua* (Hübner)

**Table R7** Mortality rate of compounds against *S. exigua* (Hübner) in contact toxicity

| **Compds.** | **Mortality rate (%) ± SD** | | | | | |
| --- | --- | --- | --- | --- | --- | --- |
|  | **0.625 mg/mL** | **1.250 mg/mL** | **2.500 mg/mL** | **5.000 mg/mL** | **10.000 mg/mL** | **20.000 mg/mL** |
| **10** | 8.89 ± 2.06 | 17.78 ± 4.89 | 32.22 ± 3.72 | 35.56 ± 5.16 | 58.89 ± 1.51 | - |
| **30** | 1.11 ± 0.27 | 5.56 ± 1.74 | 11.11 ± 3.29 | 22.22 ± 5.98 | 35.56 ± 4.68 | 56.07 ± 2.26 |
| **Cyhalothrin** | 11.11 ± 3.73 | 25.56 ± 4.76 | 46.67 ± 4.32 | 61.11 ± 3.53 | 92.22 ± 6.18 | - |

The experiments were repeated three times.

**Table R8** Lethal concentration 50% (LC_50_) and regression equation of contact toxicity against *S. exigua* (Hübner)

| **Compds.** | **Regression equation** | **R^2^** | **LC_50_ (mg /mL)** | **95% Confidence Interval** |
| --- | --- | --- | --- | --- |
| **10** | y = 2.69 / (1 + (x / 1.48) ^ 0.88) | 0.99991 | 5.79 | (5.08, 6.56) |
| **30** | y = 2.39 / (1 + (x / 2.83) ^ 1.04) | 0.98975 | 12.58 | (8.44, 18.23) |
| **Cyhalothrin** | y = 3.11 / (1 + (x / 3.53) ^ 0.99) | 0.99265 | 2.55 | (1.00, 6.29) |


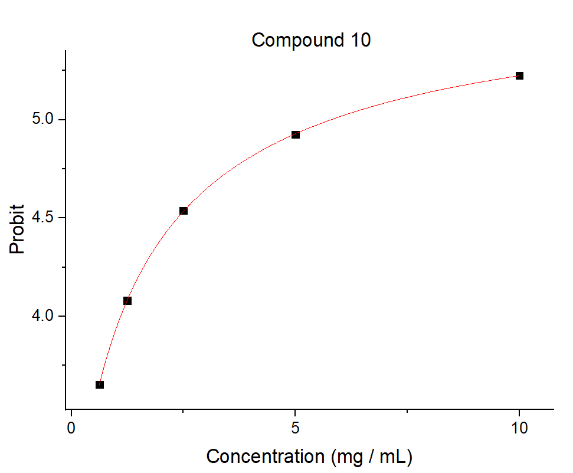

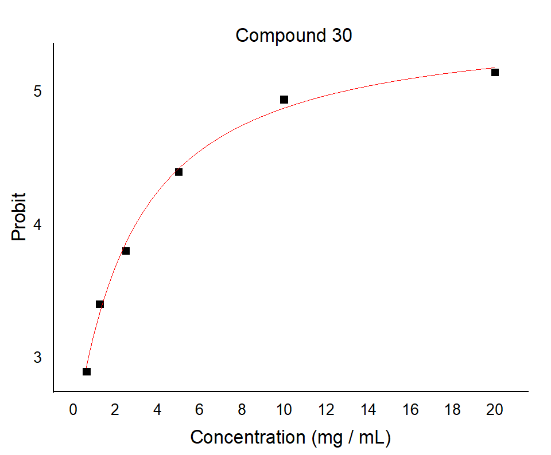
The experiments were repeated three times.
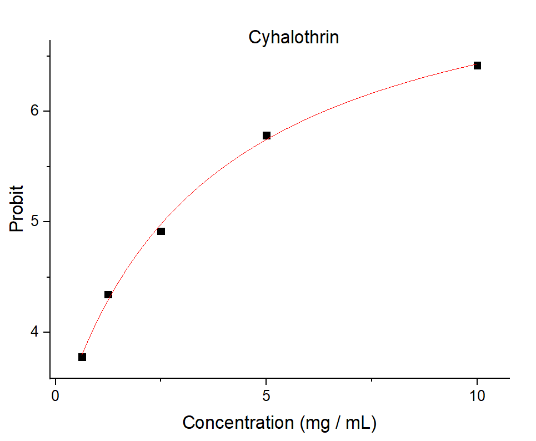


**Figure R14** Regression curve of Cyhalothrin in contact toxicity

**Figure R13** Regression curve of Compound 30 in contact toxicity

**Figure R12** Regression curve of Compound 10 in contact toxicity

# Raw data on inhibition rate of enzymes

**Table R9** Inhibition rate (IR) of enzymes of compound **33** in concentration of 0.50 mg/mL.

|  | **IR (%) ± SD** | | | |
| --- | --- | --- | --- | --- |
|  | **AChE** | **MFO** | **GST** | **CES** |
| **Compound 33** | 3.82 ± 0.01 | 84.23 ± 0.11 | 4.22 ± 0.18 | 90.23 ± 0.01 |
| **Azadirachtin A** | < 0 | < 0 | 71.24 ± 0.13 | 88.37 ± 0.01 |

The experiments were repeated three times. AChE: Acetylcholinesterase, MFO: Mixed function oxidase, GST: Glutathione S-transferases, CES: Carboxylesterase.
